# Detecting Differentially Variable MicroRNAs via Model-Based Clustering

**DOI:** 10.1155/2018/6591634

**Published:** 2018-07-12

**Authors:** Xuan Li, Yuejiao Fu, Xiaogang Wang, Dawn L. DeMeo, Kelan Tantisira, Scott T. Weiss, Weiliang Qiu

**Affiliations:** ^1^Department of Mathematics and Statistics, York University, Toronto, ON, Canada; ^2^Channing Division of Network Medicine, Brigham and Women's Hospital, Harvard Medical School, Boston, USA

## Abstract

Identifying differentially variable (DV) genomic probes is becoming a new approach to detect novel genomic risk factors for complex human diseases. The *F* test is the standard equal-variance test in statistics. For high-throughput genomic data, the probe-wise *F* test has been successfully used to detect biologically relevant DNA methylation marks that have different variances between two groups of subjects (e.g., cases versus controls). In addition to DNA methylation, microRNA (miRNA) is another important mechanism of epigenetics. However, to the best of our knowledge, no studies have identified DV miRNAs. In this article, we proposed a novel model-based clustering method to improve the power of the probe-wise *F* test to detect DV miRNAs. We imposed special structures on covariance matrices for each cluster of miRNAs based on the prior information about the relationship between variances in cases and controls and about the independence among them. Simulation studies showed that the proposed method seems promising in detecting DV probes. Based on two real datasets about human hepatocellular carcinoma (HCC), we identified 7 DV-only miRNAs (hsa-miR-1826, hsa-miR-191, hsa-miR-194-star, hsa-miR-222, hsa-miR-502-3p, hsa-miR-93, and hsa-miR-99b) using the proposed method, one (hsa-miR-1826) of which has not yet been reported to be related to HCC in the literature.

## 1. Introduction

Investigating the relationship between genomics and complex human diseases has greatly improved our understanding of the molecular mechanisms of, and the interplay of environmental factors and genomic factors to, the complex human diseases. High-throughput data from cutting-edge technologies have substantially facilitated the unbiased discovery of the genetic risk factors for many diseases. The standard approach to identify disease-associated genomic probes is to test if the mean level (e.g., DNA methylation) between cases and controls is significantly different. In addition to the mean, the variance is another important summary statistic. The larger the variance is, the more information the data could provide. However, the information about variance has not been directly used to detect disease-associated genomic probes until recent years.

Several groups of researchers have recently identified DNA methylation marks that have different variances between cases and controls [1–3]. They observed that (1) for differentially variable (DV) DNA methylation marks the variability in cases is usually higher than that in controls and (2) DV DNA methylation marks are biologically relevant. DNA methylation is an example of an epigenetic modification. Such modification leads to heritable changes via regulation of gene expression, without changing the genetic code. DNA methylation inhibits gene expression by adding a methyl group to the cytosine or adenine DNA nucleotides. Another example of an epigenetic modification is microRNAs (miRNAs) that are short noncoding 18–25-nucleotide-long RNA and negatively regulate mRNA translation [4, 5]. However, to the best of our knowledge, no studies have investigated differential variability for miRNAs. The main objective of this article is to develop statistical methods to detect DV miRNAs between cases and controls.

The *F* test is the classical method to test for equal variance between two groups of subjects, which evaluates whether the ratio of sample variances between two groups is significantly different from one. For high-throughput genomic data, such as DNA methylation data, the probe-wise *F* test could be used. That is, we first perform the *F* test for each probe to test for equal variances between cases and controls. We then calculate FDR-adjusted *p* value to control for multiple testing, where FDR stands for false discovery rate. If the FDR-adjusted *p* value < 0.05 for a DNA methylation mark, we then claim that this DNA methylation mark is differentially variable between cases and controls. The advantages of this probe-wise approach include flexibility (one model per probe) and easy implementation. However, DV probes might be governed by the same underlying mechanism. Statistically speaking, DV probes might follow the same distribution. Similarly, non-DV probes might also follow the same distribution. We hypothesize that these underlying distributions of variances could help us improve the power of the *F* test to detect DV probes.

A few methods have been proposed in the literature to borrow information across probes to detect differentially variable genomic probes. For example, Bar et al. [6] proposed a mixture-model approach for parallel detection of differential variances in genomic data analysis, by assuming that the ratio of sample variances between two groups for a given probe is drawn from a three-component mixture. Bar et al. [7] introduced a bivariate model (N3) to account for both differential expression and differential variation in high-throughput data analysis, by assuming that both means and variances follow three-component mixture distributions. Bar and Schifano [8] proposed a unified three-component mixture model, the L_2_N model, that can be used to detect either differential expression (mean) or differential variation, by modeling the differences of means and variances (dispersions) between two groups of samples. In the L_2_N model, one log-normal component is used to fit underexpressed (dispersed) probes, one log-normal component is used to fit overexpressed (dispersed) probes, and one normal component is used to fit nondifferentially expressed (dispersed) probes. These models characterize the distributions of the summary statistics (e.g., mean, variance, or difference of means), instead of the observed expression levels.

In this article, we propose a mixture of three-component multivariate normal distributions to fit the expression levels of miRNAs to identify DV miRNAs between cases and controls.

## 2. Method

### 2.1. Model

We assume that miRNAs belong to one and only one of the following three clusters: (1) miRNAs having higher variances in cases than in controls (denoted as the OV cluster), (2) miRNAs having equal variances between cases and controls (denoted as the EV cluster), and (3) miRNAs having smaller variances in cases (denoted as the UV cluster). We followed Qiu et al. [9] to directly model the marginal distributions of miRNAs in the 3 clusters. In this article, we modified Qiu et al.'s marginal model [9] to allow the detection of DV probes. We assume that (1) data have been normalized to remove the effects of confounding factors, such as chip effect and batch effect, and (2) data have been transformed so that the distributions of miRNA expressions are close to normal distributions.

For a given miRNA, we denote *X_i_* as the preprocessed expression for the *i*th subject, *i* = 1,…, *m*, where *m* = *m*_*c*_ + *m*_*n*_, *m*_*c*_ is the number of cases, and *m*_*n*_ is the number of controls. For the *k*th cluster (*k* = 1, 2, or 3), we assume that the expressions of the *m*_*c*_ cases are identically distributed with mean *μ*_*kc*_ and variance *σ*_*kc*_^2^. We assume that the expressions of the *m*_*n*_ controls are identically distributed with mean *μ*_*kn*_ and variance *σ*_*kn*_^2^. According to Qiu et al. [9], *X*_*i*_'s are marginally correlated with correlation *ρ*_*kc*_ for cases and *ρ*_*kn*_ for controls. We also assume that (1) cases and controls are independent, and (2) the *m* × 1 random vector (*X*_1_,…, *X_m_*)*^T^* follows a multivariate normal distribution. For the OV cluster, we require that *σ*_1*c*_^2^ > *σ*_1*n*_^2^. For the UV cluster, we require that *σ*_3*c*_^2^ < *σ*_3*n*_^2^. For the EV cluster, we require that *σ*_2*c*_^2^ = *σ*_2*n*_^2^. We allow the means and correlations to be different between cases and controls in the EV cluster.

We used the EM algorithm [10] to estimate the model parameters *μ*_*kc*_, *σ*_*kc*_^2^, *μ*_*kn*_, and *σ*_*kn*_^2^. The posterior probability *p*_*gk*_ = *Pr*(*g*th miRNA in *k*th cluster | *x*) = *π*_1_ *f*_1_(*x*)/[*π*_1_ *f*_1_(*x*) + *π*_2_ *f*_2_(*x*) + *π*_3_ *f*_3_(*x*)] is used to assign the *g*th miRNA to one of the 3 clusters, where *f*_*k*_(*x*) is the density function of the multivariate normal distribution for the *k*th cluster. If *p*_*g*1_ is the largest posterior probability among *p*_*g*1_, *p*_*g*2_, and *p*_*g*3_, then the *g*th miRNA will be assigned to the 1st cluster (i.e., OV cluster). The supplementary document gives the details about the model and the corresponding parameter estimation procedure.

### 2.2. Real Datasets

We downloaded two miRNA datasets from NIH's Gene Expression Omnibus (GEO) [11]: GSE67138 (https://www.ncbi.nlm.nih.gov/geo/query/acc.cgi?acc=GSE67138) and GSE67139 (https://www.ncbi.nlm.nih.gov/geo/query/acc.cgi?acc=GSE67139). Both datasets are from the same project that aims at detecting miRNAs differentially expressed between human hepatocellular carcinoma (HCC) tumor tissues with and without vascular invasion. GSE67138 is the first batch containing 57 samples (34 invasive tumor tissues and 23 noninvasive tumor tissues), while GSE67139 is the second batch containing 120 samples (60 invasive tumor tissues and 60 noninvasive tumor tissues). The expression levels of miRNAs in both GEO datasets were measured by using Affymetrix Multispecies miRNA-1 Array (GPL8786). Both datasets contain 847 miRNAs.

We checked the data quality by visualizing the plot (Figure A1) of percentiles across arrays and the scatterplot (Figure A2) of the first two principal components. Both plots indicate that the two datasets have been cleaned and have good quality (i.e., no apparent outlying miRNAs, outlying arrays, or technical batch effects). Hence, we directly used the two datasets in the further analyses. Since GSE67139 has a larger sample size than GSE61738, we regarded GSE67139 as the discovery set and GSE67138 as the validation set.

### 2.3. Simulation

We conducted 4 sets of simulation studies. In the first set (denoted as SimI), we generated miRNA data from the proposed marginal mixture model, where estimated model parameters for GSE67139 (i.e., the discovery set) are used as the true values of the model parameters (*π*_1_ = 0.31, *π*_2_ = 0.58, *π*_3_ = 0.11, *μ*_1*c*_ = −0.14, *σ*_1*c*_^2^ = 1.49, *ρ*_1*c*_ = 0.08, *μ*_1*n*_ = 0.14, *σ*_1*n*_^2^ = 0.45, *ρ*_1*n*_ = 0.32, *μ*_2*c*_ = 0.03, *σ*_2*c*_^2^ = 1.01, *ρ*_2*c*_ = 0.04, *μ*_2*n*_ = −0.03, *σ*_2*n*_^2^ = 1.01, *ρ*_2*n*_ = 0.11, *μ*_3*c*_ = 0.13, *σ*_3*c*_^2^ = 0.28, *ρ*_3*c*_ = 0.04, *μ*_3*n*_ = −0.13, *σ*_3*n*_^2^ = 1.69, *ρ*_1*n*_ = −0.01). We generated 100 datasets, each of which has 1000 miRNAs for 50 cases and 50 controls. Thirty one percent (310) of the 1000 miRNAs are in the OV cluster. Eleven percent (110) of the miRNAs are in the UV cluster. The remaining 58% (580) miRNAs are in the EV cluster.

In the second set (denoted as SimII), we generated miRNA data from a mixture of 3 multivariate *t* distribution with the same mean vectors and covariance matrices as those in SimI and with 3 degrees of freedom. SimII is used to evaluate the performance of the proposed method when the normality assumption for any one of the three clusters (OV, EV, and UV) is violated.

In the third set (denoted as SimIII) of the simulation studies, we generated miRNA data from the same model as that in SimI, except that the marginal correlation within-subject groups were set to zero (*ρ*_*kc*_ = 0 and *ρ*_*kn*_ = 0). SimIII is used to evaluate the performance of the proposed method when there are no marginal correlations.

In the fourth set (denoted as SimIV) of the simulation studies, we generated miRNA data from the same model as that in SimII, except that the marginal correlations within-subject groups were set to zero (*ρ*_*kc*_ = 0 and *ρ*_*kn*_ = 0). SimIV is used to evaluate the performance of the proposed method when there are no marginal correlations and when the normality assumption for any one of the three clusters (OV, EV, and UV) is violated.

### 2.4. Statistical Analysis

We compared the proposed method (denoted as gs) with sixteen existing differential-variance detecting methods by using both the real datasets and the simulated datasets. The ten equal variance tests are (1) the *F* test (denoted as F), (2) Ahn and Wang's score test [12] (denoted as AW), (3) Phipson and Oshlack's AD test [13] (denoted as PO.AD), (4) Phipson and Oshlack's SQ test [13] (denoted as PO.SQ), (5) Levene's test [14] (denoted as L), (6) Brown and Forsythe's test [15] (denoted as BF), (7) trimmed-mean-based Levene's test [15] (denoted as Ltrim), (8) improved AW test based on Levene's test [16] (denoted as iL), (9) improved AW test based on the BF test [16] (denoted as iBF), and (10) improved AW test based on the trimmed-mean-based Levene's test [16] (denoted as iTrim). The remaining six methods are based on Bar et al.'s [7] N3 model and Bar and Schifano's [8] L_2_N model. Both N3 and L_2_N models have been implemented in the R package DVX [8]. For both N3 and L_2_N, DVX outputs raw *p* values, *q* values, and posterior probabilities *p*_*gk*_ that the probe *g* belongs to cluster *k* given its expression profile and estimated model parameters, *k* = 1, 2, 3. Hence, for both N3 and L_2_N, we used three methods to assign probes to two clusters: DV probes and non-DV probes. The first method is based on the *q* value. If a miRNA has a *q* value < 0.05, we claim it is differentially variable; otherwise, we claim it is nondifferentially variable. The second method is based on the false discovery rate- (FDR-) adjusted *p* value. If a miRNA has an FDR-adjusted *p* value < 0.05, we claim it is differentially variable; otherwise, we claim it is nondifferentially variable. The third method is based on the posterior probabilities. We assign a miRNA to cluster *k*∗ if the posterior probability *p*_*gk*∗_ is the largest among the 3 posterior probabilities, *p*_*g*1_, *p*_*g*2_, and *p*_*g*3_. We denote the 3-miRNA assignment methods as N3.q (L_2_N.q), N3.f (L_2_N.f), and N3 (L_2_N), respectively.

In real data analysis, we followed Qiu et al.'s [9] data preprocessing steps. That is, we first performed the same Box-Cox transformation for each expression level, and then for each miRNA, we performed mean centering and scaling operations so that the mean expression level is 0 and the variance is 1. We then applied the 17 methods (the gs method and the 16 existing methods) to the discovery set (GSE67139) to detect DV miRNAs between invasive tumors and noninvasive tumors. For the 10 probe-wise tests (F, AW, PO.AD, PO.SQ, L, BF, Ltrim, iL, iBF, and iTrim), we obtained FDR-adjusted *p* values. If a miRNA has an FDR-adjusted *p* value < 0.05, we claim that this miRNA has significantly different variances between invasive tumors and noninvasive tumors. We then applied the same procedure to the validation set (GSE67138). We claim that a miRNA is a validated DV miRNA (1) if the miRNA is DV in both discovery and validation sets and (2) if the sign of the difference (*s*_*c*_^2^ − *s*_*n*_^2^) is the same in both datasets, where *s*_*c*_^2^ and *s*_*n*_^2^ are sample variances for cases and controls, respectively. We next calculated the proportion of the validated DV miRNAs (i.e., validation rate) pValid = *n*_12_/*n*_1_, where *n*_1_ is the number of DV miRNAs in the discovery set (GSE67139) and *n*_12_ is the number of significant DV miRNAs sharing the same difference direction of variances in both data sets. To estimate the variation of the validation rate pValid, we obtained the 100 bootstrap validation rates based on 100 bootstrap discovery and validation sets. We then test if the median bootstrap validation rate of the gs method is the same as that of each of the other 16 methods by two-sided Wilcoxon signed rank tests.

For the validated DV miRNAs detected by the gs method, we also checked if they are validated differentially expressed (DE) miRNAs by using R Bioconductor package *limma* [17]. A miRNA is a validated DE miRNA if the FDR-adjusted *p* value for testing equal mean expression between cases and controls is <0.05 in both the discovery and validation sets and if the sign of the mean difference ${\bar{x}}_{c}-{\bar{x}}_{n}$ is the same in both discovery and validation sets, where ${\bar{x}}_{c}$ and ${\bar{x}}_{n}$ are the sample means of the cases and controls, respectively. Denote *S*_DVonly_ as the set of miRNAs that are validated DV, but not validated DE. Denote *S*_DEonly_ as the set of miRNAs that are validated DE, but not validated DV. Denote *S*_both_ as the set of miRNAs that are both validated DE and validated DV.

We applied the miRSystem [18] to predict the target genes of miRNAs in each of the 3 sets: *S*_DVonly_, *S*_DEonly_, and *S*_both_. The miRSystem also provides the enriched KEGG pathways for the predicted target genes.

For simulated datasets, we calculated the magnitude of agreement between the true cluster memberships of miRNAs and the detected cluster memberships by each of the 17 methods by using the Jaccard index [9, 19]. The maximum value of the Jaccard index is one, indicating perfect agreement. The minimum value of the Jaccard index is zero, indicating that the agreement is by chance. We also evaluate the performances using false positive rate (FPR) (i.e., the proportion of detected DV probes among the true non-DV probes) and false negative rate (FNR) (i.e., the proportion of detected non-DV probes among the true DV probes). The smaller the FPR (FNR) is, the better the performance is.

## 3. Result

**Figure 1:**
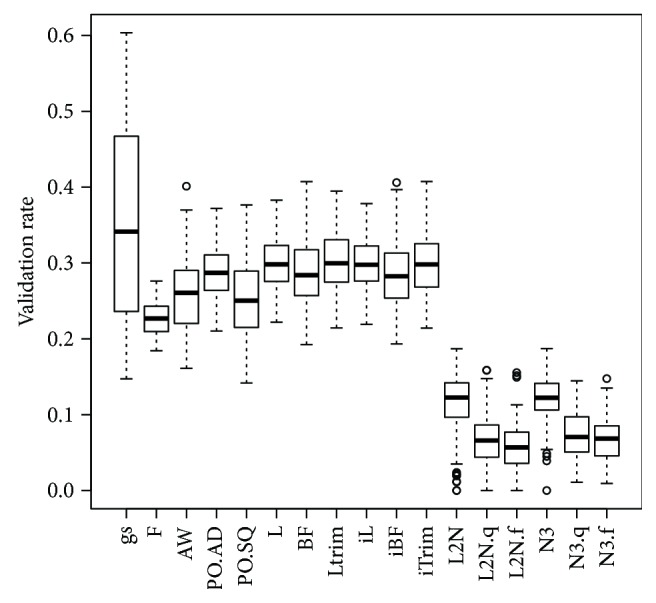
Boxplots of validation rates based on 100 bootstrap samples of the discovery set (GSE67139) and the validation set (GSE67138).

For the real data analyses, the numbers of the DV miRNAs in the discovery set (GSE67139), and the numbers and proportions of the validated DV miRNAs are shown in Table 1. The gs method detected 358 DV probes based on the discovery set (GSE67139), 67 of which are validated in the validation set (GSE67138). Among the 67 validated DV miRNAs, 66 miRNAs are OV and only one miRNA is UV. The proportion of the validated DV miRNAs is 0.19 for the gs method, which is higher than those of the N3 and L_2_N methods but lower than those of the 10 probe-wise tests. However, the gs method had the highest median bootstrap validation rate among all 17 methods (Figure 1). For all the 17 methods, the number (nValid.OV) of the validated OV miRNAs is much larger than the number (nValid.UV) of the validated UV miRNAs. This observation is consistent with that observed by other researchers using DNA methylation data [3].

We got 392 DE miRNAs based on the discovery set (GSE67139), among which 217 DE miRNAs were validated. There are only 7 miRNAs in *S*_DVonly_ (hsa-miR-1826, hsa-miR-191, hsa-miR-194-star, hsa-miR-222, hsa-miR-502-3p, hsa-miR-93, and hsa-miR-99b), the parallel boxplots of which are shown in Figure A3. *S*_DEonly_ contains 157 miRNAs (Table A1), the parallel boxplots of which are shown in Figure A4. *S*_both_ contains 60 miRNAs (Table A2), the parallel boxplots of which are shown in Figure A5.

Based on the miRSystem analysis, there are 1639 genes (Table A3) targeted by the 7 miRNAs in *S*_DVonly_, 8141 targeted genes (Table A4) for the 157 miRNAs in *S*_DEonly_, and 6893 targeted genes (Table A5) for the 60 miRNAs in *S*_both_. The 1639 genes targeted by the 7 miRNAs in *S*_DVonly_ are significantly enriched (raw *p* value < 0.05) in 6 KEGG pathways (calcium signaling pathway, salivary secretion, amyotrophic lateral sclerosis (ALS), MAPK signaling pathway, PPAR signaling pathway, and Alzheimer's disease) (Table A6). The 8141 genes targeted by the 157 miRNAs in *S*_DEonly_ are significantly enriched in only one KEGG pathway (antigen processing and presentation) with raw *p* value = 2.70*E* − 2 (Table A7). The 6893 genes targeted by the 60 miRNAs in *S*_both_ are enriched in two KEGG pathways (O-glycan biosynthesis and glycine serine and threonine metabolism) (Table A8).

**Figure 2:**
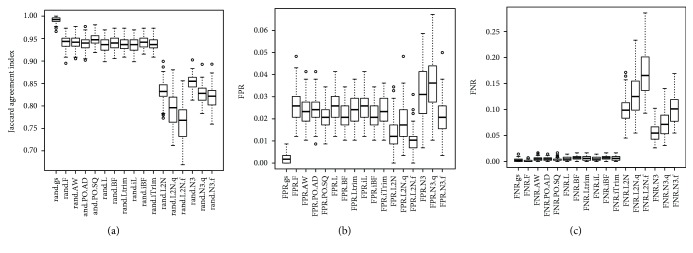
The boxplots of the 100 estimated Jaccard indices, FPR, and FNR based on the 100 simulated datasets in SimI (generating data from a mixture of multivariate normal distributions with nonzero marginal correlations). The closer the Jaccard index is to one, the better the performance of the method is. The closer the FPR (FNR) is to zero, the better the performance of the method is.

**Figure 3:**
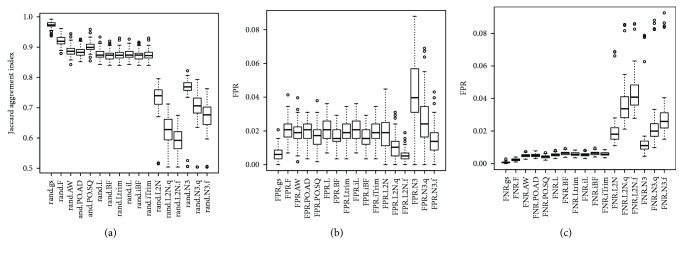
The boxplots of the 100 estimated Jaccard indices based on the 100 simulated datasets in SimIII (generating data from a mixture of multivariate normal distributions with zero marginal correlations). The closer the Jaccard index is to one, the better the performance of the method is. The closer the FPR (FNR) is to zero, the better the performance of the method is.

For the first and the third simulation studies (SimI and SimIII) where data were generated from a mixture of multivariate normal distributions, the values of the Jaccard index obtained by the gs method are close to one (the perfect agreement). Figures 2 and 3 showed that the boxplots of the Jaccard index obtained by the gs method are higher than boxplots obtained by the other 16 methods. The small *p* values in Table A9 showed that the gs method had significantly higher values of the Jaccard index than the 16 existing equal-variance tests. Figures 2 and 3 and Tables A10 and A11 also showed that the gs method had significantly smaller FPR and FNR than the other 16 methods, except that the gs method had significantly higher median FNR in SimI (Figure 2).

**Figure 4:**
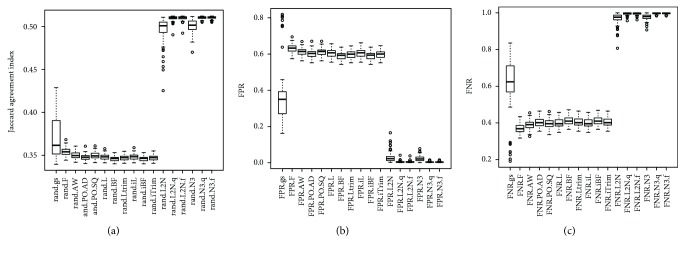
The boxplots of the 100 estimated Jaccard indices based on the 100 simulated datasets in SimII (generating data from a mixture of multivariate *t* distributions with nonzero marginal correlations). The closer the Jaccard index is to one, the better the performance of the method is. The closer the FPR (FNR) is to zero, the better the performance of the method is.

**Figure 5:**
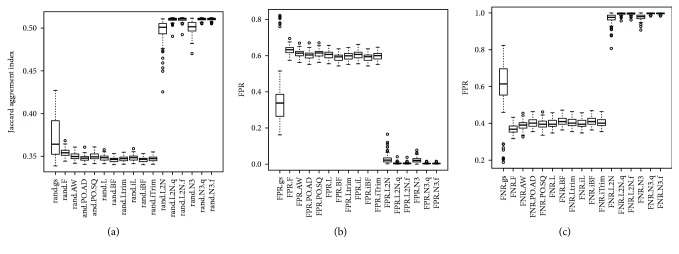
The boxplots of the 100 estimated Jaccard indices based on the 100 simulated datasets in SimIV (generating data from a mixture of multivariate *t* distributions with zero marginal correlations). The closer the Jaccard index is to one, the better the performance of the method is. The closer the FPR (FNR) is to zero, the better the performance of the method is.

Figures 4 and 5 and Table A9 showed that for SimII and SimIV where data was generated from a mixture of multivariate *t* distributions, the N3 and L_2_N methods had the highest Jaccard index values (median Jaccard index > 0.50), while the remaining 11 methods had a median Jaccard index < 0.4. The gs method still had much higher values of the Jaccard index than the 10 probe-wise tests (F, AW, PO.AD, PO.SQ, L, BF, Ltrim, iL, iBF, and iTrim). Figures 4 and 5 and Tables A10 and A11 also showed that the gs method had a lower FPR than the 10 probe-wise tests, but had higher FNR than these 10 probe-wise tests. Although the N3 and L2N methods had very low FPR, they had FNR close to one.

Tables A9–A12 showed the *p* values of two-sided Wilcoxon signed rank tests to evaluate if the median measures (Jaccard index, FPR, FNR, and bootstrap validation rate) are the same as those of each of the other 16 methods, respectively. All *p* values are smaller than 0.05, indicating that all the differences are statistically significant.

## 4. Discussion

In this article, we proposed a novel model-based clustering method (the gs method) to detect miRNAs having different variances between cases and controls. The proposed method is different from probe-wised equal-variance tests in that it does not involve hypothesis testing. The real data analysis showed that the gs method had a larger median bootstrap validation rate than the 16 existing equal-variance detecting methods. The four simulation studies showed that the gs method outperformed the 16 existing equal-variance detection methods if the miRNA data follow a mixture of multivariate normal distributions. If the data were generated from other distributions, such as a mixture of multivariate *t* distributions, the gs method had a lower FPR and a higher FNR than the 10 probe-wise tests. Since controlling FPR is more important than controlling FNR, the gs method is promising in the genomic data analysis.

Several model-based clustering algorithms have been proposed to detect DV genomic probes in the literature, such as Bar et al.'s [7] N3 methods, and Bar and Schifano's [8] L_2_N methods. The N3 methods and L2N methods do not seem to work as well as the gs method under the simulation scenarios in this article. This is probably partly due to the gs method that directly models the observed expression levels, while the N3 and L_2_N methods model the summary statistics (e.g., mean, variance, or difference of means). Using summary statistics might cause the loss of information. Moreover, the N3 and L_2_N methods applied a couple of approximations to derive the marginal densities, while approximations might cause deviations from true marginal densities.

In the simulation studies, the proposed method outperformed the 10 probe-wised tests, including the classic *F* test that has been reported to outperform other equal-variance tests when the normality assumption is held [20, 21]. The reason why the gs method performed better than the *F* test in SimI and SimIII, where the normality assumption for any one of the three clusters (OV, EV, and UV) is held, is that the gs method could borrow information across miRNAs (i.e., the estimation of the model parameters uses the information provided by all the miRNAs).

The gs method had lower FPR than the 10 probe-wised equal-variance tests in SimII and SimIV, where the data were generated from a mixture of multivariate *t* distribution (i.e., the normality assumption for any one of the three clusters (OV, EV, and UV) is violated) (Figure A6). However, the gs method had higher FNR than the 10 probe-wised tests in SimII and SimIV. The results of SimII and SimIV also suggest that the large variation of the validation rate in the real data analyses (Figure 1) may be due to the violation of the underlying assumption that the expression levels are from the mixture of multivariate normal distributions.

Figure A7 showed that the distributions of the original real datasets are quite different from a mixture of normal distributions. Note that in genomic data analysis, majority probes are supposed to be nondifferentially expressed. Hence, we can use a histogram and QQ plot to roughly check if data are from a mixture of normal distributions. We followed Qiu et al.'s [9]data preprocessing steps. That is, we first performed the same Box-Cox transformation to each expression level, and then for each miRNA, we performed mean-centering and scaling operations so that the mean expression level is 0 and the variance is 1. Figure A7 showed that even after the Box-Cox transformation and scaling, the data distributions are still quite different from normal distributions. Further investigation is warranted to develop more robust model-based clustering methods.

In the real data analysis, the gs method detected 67 validated DV miRNAs (66 OV and 1 UV), seven of which are DV only. The 7 DV-only miRNAs (hsa-miR-1826, hsa-miR-191, hsa-miR-194-star, hsa-miR-222, hsa-miR-502-3p, hsa-miR-93, and hsa-miR-99b) were targeted to 1639 genes based on the miRSystem analysis. Except for hsa-miR-1826, all DV-only miRNAs have been associated with HCC. Elyakim et al. [22] showed that miR-191 is a candidate oncogene target for hepatocellular carcinoma therapy. Law and Wong [23] reported the association of miR-194 with the metastatic behavior of HCC. Murakami et al [24] reported that miR-222 is increased in poorly versus moderately versus well-differentiated hepatomas. Jin et al. [25] reported that miR-502-3p suppressed cell proliferation, migration, and invasion in HCC by targeting SET. Li et al. [26] confirmed that the miR-106b-25 cluster, which miR-93 belongs to, is overexpressed in HCC. Morishita et al. [27] found that miR-99b is upregulated in HBV-infected HCC cells.

The 1639 genes, which are targeted by the 7 DV-only miRNAs, are enriched in 6 KEGG pathways (calcium signaling pathway, salivary secretion, amyotrophic lateral sclerosis (ALS), MAPK signaling pathway, PPAR signaling pathway, and Alzheimer's disease). All these 6 pathways have been linked to HCC in the literature. For example, Huang et al. [28] reported that increased mitochondrial fission induced cytosolic calcium signaling in HCC cells. Chen et al. [29] reported that in a mice study, DNA methylation marks that are differentially methylated between livers with HCC and livers without HCC are enriched in the salivary secretion pathway. Seol et al.'s [30] results suggest that Riluzole, an amyotrophic lateral sclerosis (ALS) drug, has an anticancer effect on HCC. Feng et al. [31] reported that cantharidic acid inhibits HCC cell proliferation by inducing cell apoptosis through the p38 MAPK signaling pathway. Nwosu et al. [32] reported that downregulated genes (HCC versus non-HCC) were enriched in the PPAR signaling pathway based on each of the 8 HCC datasets downloaded from the Gene Expression Omnibus (GEO). Jin et al. [33] reported that kynurenine 3-monooxygenase (KMO), an enzyme playing a critical role in Huntington's and Alzheimer's diseases, exhibits tumor-promoting effects towards HCC. Hence, DV-only miRNAs are biologically relevant to HCC.

There are no overlaps among the enriched pathways for the 3 sets of miRNAs in the real data analysis: *S*_DVonly_ (the set of miRNAs that are validated DV, but not validated DE), *S*_DEonly_ (the set of miRNAs that are validated DE, but not validated DV), and *S*_both_ (the set of miRNAs that are both validated DE and validated DV). This indicates that DV-only miRNAs might provide additional information about the molecular mechanisms of HCC than that provided by DE miRNAs.

In summary, the proposed gs method assumes expression levels from the mixture of multivariate normal distributions. The proposed gs method seems promising to detect differential variability based on our simulation studies. In the future, we will improve it into a robust version against the violation of the normality assumption on the component distributions.

## Figures and Tables

**Table 1:** Information about the validated DV miRNAs.

Method	nSig	n.OV	n.UV	nValid	nValid.OV	nValid.UV	pValid
gs	358	262	96	67	66	1	0.19
F	472	349	123	99	96	3	0.21
AW	141	136	0	33	33	0	0.23
PO.AD	202	186	0	68	68	0	0.34
PO.SQ	141	136	5	32	32	0	0.23
L	201	185	16	72	70	2	0.36
BF	175	164	11	54	53	1	0.31
Ltrim	181	168	0	58	56	2	0.32
iL	199	183	16	70	68	2	0.35
iBF	174	163	11	53	52	1	0.30
iTrim	181	168	13	57	55	2	0.31
L2N	225	121	104	30	29	1	0.13
L2N.q	173	69	104	17	16	1	0.10
L2N.f	157	60	97	16	15	1	0.10
N3	247	141	106	34	33	1	0.14
N3.q	202	96	106	25	24	1	0.12
N3.f	178	74	104	18	17	1	0.10

nSig: the number of the DV miRNAs detected in the discovery set (GSE67139); n.OV: the number of OV miRNAs detected in the discovery set; n.UV: the number of UV miRNAs detected in the discovery set; nValid: the number of validated DV miRNAs; nValid.OV: the number of validated OV miRNAs; nValid.UV: the number of validated UV miRNAs; pValid = nValid/nSig.

## Data Availability

The authors downloaded two microRNA datasets from NIH's Gene Expression Omnibus (GEO) [8]: GSE67138 (https://www.ncbi.nlm.nih.gov/geo/query/acc.cgi?acc=GSE67138) and GSE67139 (https://www.ncbi.nlm.nih.gov/geo/query/acc.cgi?acc=GSE67139).

## Supplementary Materials

Supplementary Materials Details about the gs model and its parameter estimation via the EM algorithm. Supplemental Table A1: the list of the 157 DE-only miRNAs. Supplemental Table A2: the list of the 60 DV-and-DE miRNAs. Supplemental Table A3: the list of the 1639 genes targeted by the 7 miRNAs in *S*_DVonly_. Supplemental Table A4: the list of the 8141 genes targeted by the 157 miRNAs in *S*_DEonly_. Supplemental Table A5: the list of the 6893 genes targeted by the 60 miRNAs in *S*_both_. Supplemental Table A6: the list of the 6 KEGG pathways enriched by the 1639 genes targeted by the 7 miRNAs in *S*_DVonly_. Supplemental Table A7: the list of the 1 KEGG pathway enriched by the 8141 genes targeted by the 157 miRNAs in *S*_DEonly_. Supplemental Table A8: the list of the 2 KEGG pathways enriched by the 6893 genes targeted by the 60 miRNAs in *S*_both_. Supplemental Table A9: *p* values of two-sided Wilcoxon signed rank tests to test if the median Jaccard index obtained by the gs method is the same as that obtained by each of the other 16 methods. Supplemental Table A10: *p* values of two-sided Wilcoxon signed rank tests to test if the median FPR obtained by the gs method is the same as that obtained by each of the other 16 methods. Supplemental Table A11: *p* values of two-sided Wilcoxon signed rank tests to test if the median FNR obtained by the gs method is the same as that obtained by each of the other 16 methods. Supplemental Table A12: *p* values of two-sided Wilcoxon signed rank tests to test if the median proportion of validation obtained by the gs method is the same as that obtained by each of the other 16 methods based on 100 bootstrap samples. Figure A1: the plot of percentiles of log2 expression levels across arrays. Left panel: GSE67138. Right panel: GSE67139. Figure A2: the plot of the first and second principal components. Left panel: GSE67138. Right panel: GSE67139. Supplemental Figure A3: parallel boxplots of the 7 validated DV-only miRNAs. Left panel: GSE67138. Right panel: GSE67139. Supplemental Figure A4: parallel boxplots of the 157 validated DE-only miRNAs. Left panel: GSE67138. Right panel: GSE67139. Supplemental Figure A5: parallel boxplots of the 60 validated DV-and-DE miRNAs. Left panel: GSE67138. Right panel: GSE67139. Supplemental Figure A6: histograms and QQ plots for a simulated dataset in each of the 4 simulation scenarios. Supplemental Figure A7: histograms and QQ plots for the two real datasets. Note that in genomic data analysis, majority probes are supposed to be nondifferentially expressed. Hence, we can use a histogram and QQ plot to roughly check if data are from a mixture of normal distributions.

## References

[1] Hansen K. D., Timp W., Bravo H. C. (2011). Increased methylation variation in epigenetic domains across cancer types. *Nature Genetics*.

[2] Jaffe A. E., Feinberg A. P., Irizarry R. A., Leek J. T. (2012). Significance analysis and statistical dissection of variably methylated regions. *Biostatistics*.

[3] Teschendorff A. E., Widschwendter M. (2012). Differential variability improves the identification of cancer risk markers in DNA methylation studies profiling precursor cancer lesions. *Bioinformatics*.

[4] Hayes J., Peruzzi P. P., Lawler S. (2014). MicroRNAs in cancer: biomarkers, functions and therapy. *Trends in Molecular Medicine*.

[5] Simonson B., Das S. (2015). MicroRNA therapeutics: the next magic bullet?. *Mini Reviews in Medicinal Chemistry*.

[6] Bar H. Y., Booth J. G., Wells M. T. (2012). A mixture-model approach for parallel testing for unequal variances. *Statistical Applications in Genetics and Molecular Biology*.

[7] Bar H. Y., Booth J. G., Wells M. T. (2014). A bivariate model for simultaneous testing in bioinformatics data. *Journal of the American Statistical Association*.

[8] Bar H., Schifano E. D. (2018). *Differential Variation and Expression Analysis*.

[9] Qiu W., He W., Wang X., Lazarus R. (2008). A marginal mixture model for selecting differentially expressed genes across two types of tissue samples. *The International Journal of Biostatistics*.

[10] Dempster A., Laird N., Rubin D. (1977). Maximum likelihood from incomplete data via the EM algorithm. *Journal of the Royal Statistical Society, Series B*.

[11] Edgar R., Domrachev M., Lash A. E. (2002). Gene Expression Omnibus: NCBI gene expression and hybridization array data repository. *Nucleic Acids Research*.

[12] Ahn S., Wang T. (2013). A powerful statistical method for identifying differentially methylated markers in complex diseases. *Pacific Symposium on Biocomputing*.

[13] Phipson B., Oshlack A. (2014). DiffVar: a new method for detecting differential variability with application to methylation in cancer and aging. *Genome Biology*.

[14] Levene H., Olkin I. (1960). Robust tests for equality of variances. *Contributions to Probability and Statistics: Essays in Honor of Harold Hotelling*.

[15] Brown M. B., Forsythe A. B. (1974). Robust tests for the equality of variances. *Journal of the American Statistical Association*.

[16] Qiu W., Li X., Morrow J. (2017). New score tests for equality of variances in the application of DNA methylation data analysis. *Insights in Genetics and Genomics*.

[17] Ritchie M. E., Phipson B., Wu D. (2015). limma powers differential expression analyses for RNA-sequencing and microarray studies. *Nucleic Acids Research*.

[18] Lu T. P., Lee C. Y., Tsai M. H. (2012). miRSystem: an integrated system for characterizing enriched functions and pathways of microRNA targets. *PLoS One*.

[19] Jaccard P. (1912). The distribution of the flora in the alpine zone. *New Phytologist*.

[20] Conover W. J., Johnson M. E., Johnson M. M. (1981). A comparative study of tests for homogeneity of variances, with applications to the outer continental shelf bidding data. *Technometrics*.

[21] Li X., Qiu W., Morrow J. (2015). A comparative study of tests for homogeneity of variances with application to DNA methylation data. *PLoS One*.

[22] Elyakim E., Sitbon E., Faerman A. (2010). hsa-miR-191 is a candidate oncogene target for hepatocellular carcinoma therapy. *Cancer Research*.

[23] Law P. T.-Y., Wong N. (2011). Emerging roles of microRNA in the intracellular signaling networks of hepatocellular carcinoma. *Journal of Gastroenterology and Hepatology*.

[24] Murakami Y., Yasuda T., Saigo K. (2006). Comprehensive analysis of microRNA expression patterns in hepatocellular carcinoma and non-tumorous tissues. *Oncogene*.

[25] Jin H., Yu M., Lin Y. (2016). MiR-502-3P suppresses cell proliferation, migration, and invasion in hepatocellular carcinoma by targeting SET. *OncoTargets and Therapy*.

[26] Li Y., Tan W., Neo T. W. L. (2009). Role of the *miR-106b-25* microRNA cluster in hepatocellular carcinoma. *Cancer Science*.

[27] Morishita A., Iwama H., Fujihara S. (2016). MicroRNA profiles in various hepatocellular carcinoma cell lines. *Oncology Letters*.

[28] Huang Q., Cao H., Zhan L. (2017). Mitochondrial fission forms a positive feedback loop with cytosolic calcium signaling pathway to promote autophagy in hepatocellular carcinoma cells. *Cancer Letters*.

[29] Chen H., Cai W., Chu E. S. H. (2017). Hepatic cyclooxygenase-2 overexpression induced spontaneous hepatocellular carcinoma formation in mice. *Oncogene*.

[30] Seol H. S., Lee S. E., Song J. S. (2016). Glutamate release inhibitor, Riluzole, inhibited proliferation of human hepatocellular carcinoma cells by elevated ROS production. *Cancer Letters*.

[31] Feng I. C., Hsieh M. J., Chen P. N. (2018). Cantharidic acid induces apoptosis through the p38 MAPK signaling pathway in human hepatocellular carcinoma. *Environmental Toxicology*.

[32] Nwosu Z. C., Megger D. A., Hammad S. (2017). Identification of the consistently altered metabolic targets in human hepatocellular carcinoma. *Cellular and Molecular Gastroenterology and Hepatology*.

[33] Jin H., Zhang Y., You H. (2015). Prognostic significance of kynurenine 3-monooxygenase and effects on proliferation, migration, and invasion of human hepatocellular carcinoma. *Scientific Reports*.

