# Supplementary material for: Detecting Differentially Variable MicroRNAs via Model-Based Clustering
Source: Int J Genomics. 2018 Jul 12;2018:6591634. doi: 10.1155/2018/6591634 (PMC6079429; doi:10.1155/2018/6591634)
Supplement: Supplementary Materials — Details about the gs model and its parameter estimation via the EM algorithm. Supplemental Table A1: the list of the 157 DE-only miRNAs. Supplemental Table A2: the list of the 60 DV-and-DE miRNAs. Supplemental Table A3: the list of the 1639 genes targeted by the 7 miRNAs in S DVonly. Supplemental Table A4: the list of the 8141 genes targeted by the 157 miRNAs in S DEonly. Supplemental Table A5: the list of the 6893 genes targeted by the 60 miRNAs in S both. Supplemental Table A6: the list of the 6 KEGG pathways enriched by the 1639 genes targeted by the 7 miRNAs in S DVonly. Supplemental Table A7: the list of the 1 KEGG pathway enriched by the 8141 genes targeted by the 157 miRNAs in S DEonly. Supplemental Table A8: the list of the 2 KEGG pathways enriched by the 6893 genes targeted by the 60 miRNAs in S both. Supplemental Table A9: p values of two-sided Wilcoxon signed rank tests to test if the median Jaccard index obtained by the gs method is the same as that obtained by each of the other 16 methods. Supplemental Table A10: p values of two-sided Wilcoxon signed rank tests to test if the median FPR obtained by the gs method is the same as that obtained by each of the other 16 methods. Supplemental Table A11: p values of two-sided Wilcoxon signed rank tests to test if the median FNR obtained by the gs method is the same as that obtained by each of the other 16 methods. Supplemental Table A12: p values of two-sided Wilcoxon signed rank tests to test if the median proportion of validation obtained by the gs method is the same as that obtained by each of the other 16 methods based on 100 bootstrap samples. Figure A1: the plot of percentiles of log2 expression levels across arrays. Left panel: GSE67138. Right panel: GSE67139. Figure A2: the plot of the first and second principal components. Left panel: GSE67138. Right panel: GSE67139. Supplemental Figure A3: parallel boxplots of the 7 validated DV-only miRNAs. Left panel: GSE67138. Right panel: GSE67139. Supplemental Fi [file 6591634.f1.zip › FigA4_IJG_2364377.pdf]

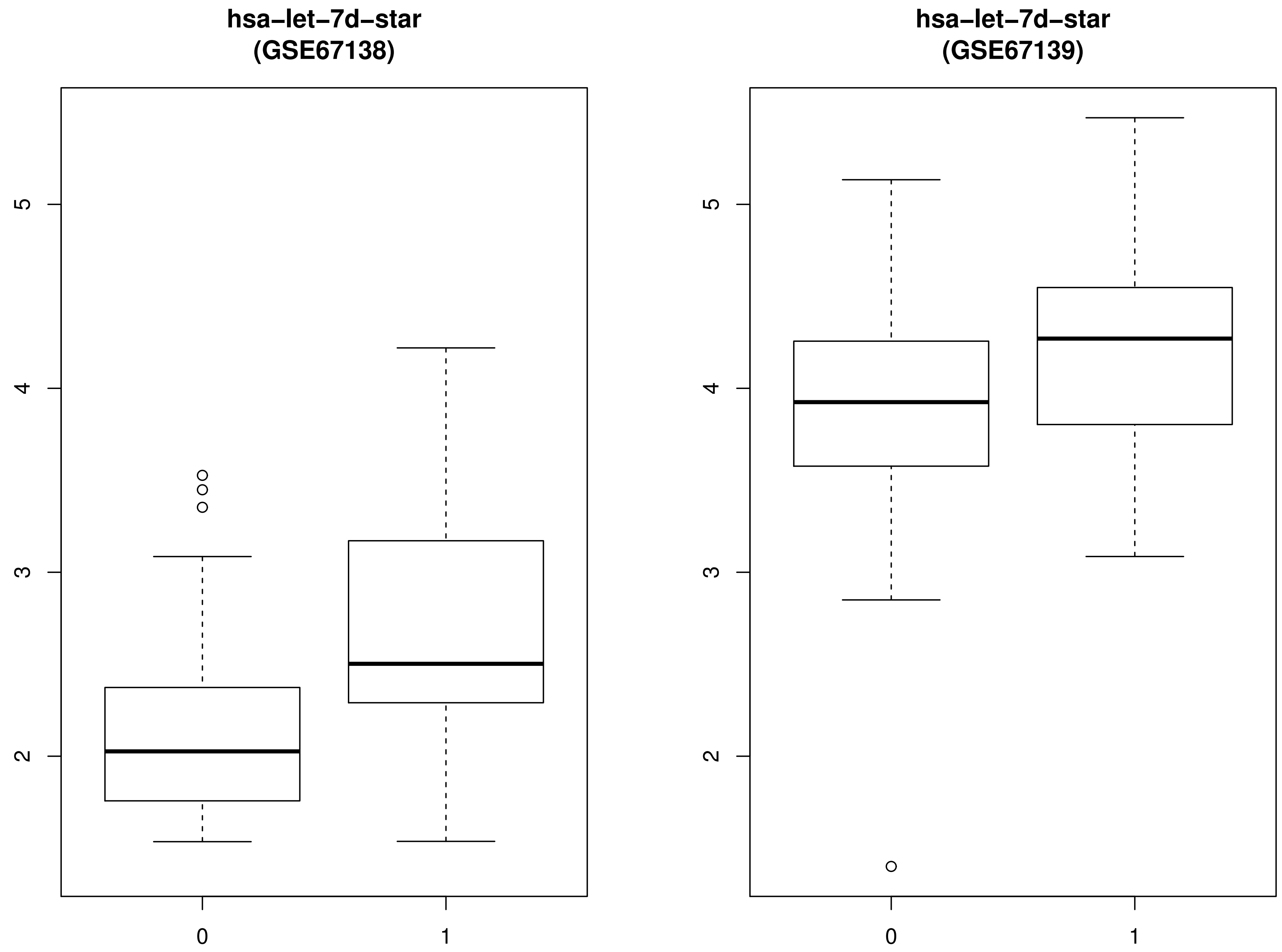
hsa−let−7d−star
(GSE67138)
hsa−let−7d−star
(GSE67139)
2
3
4
5
0
1
2
3
4
5
0
1

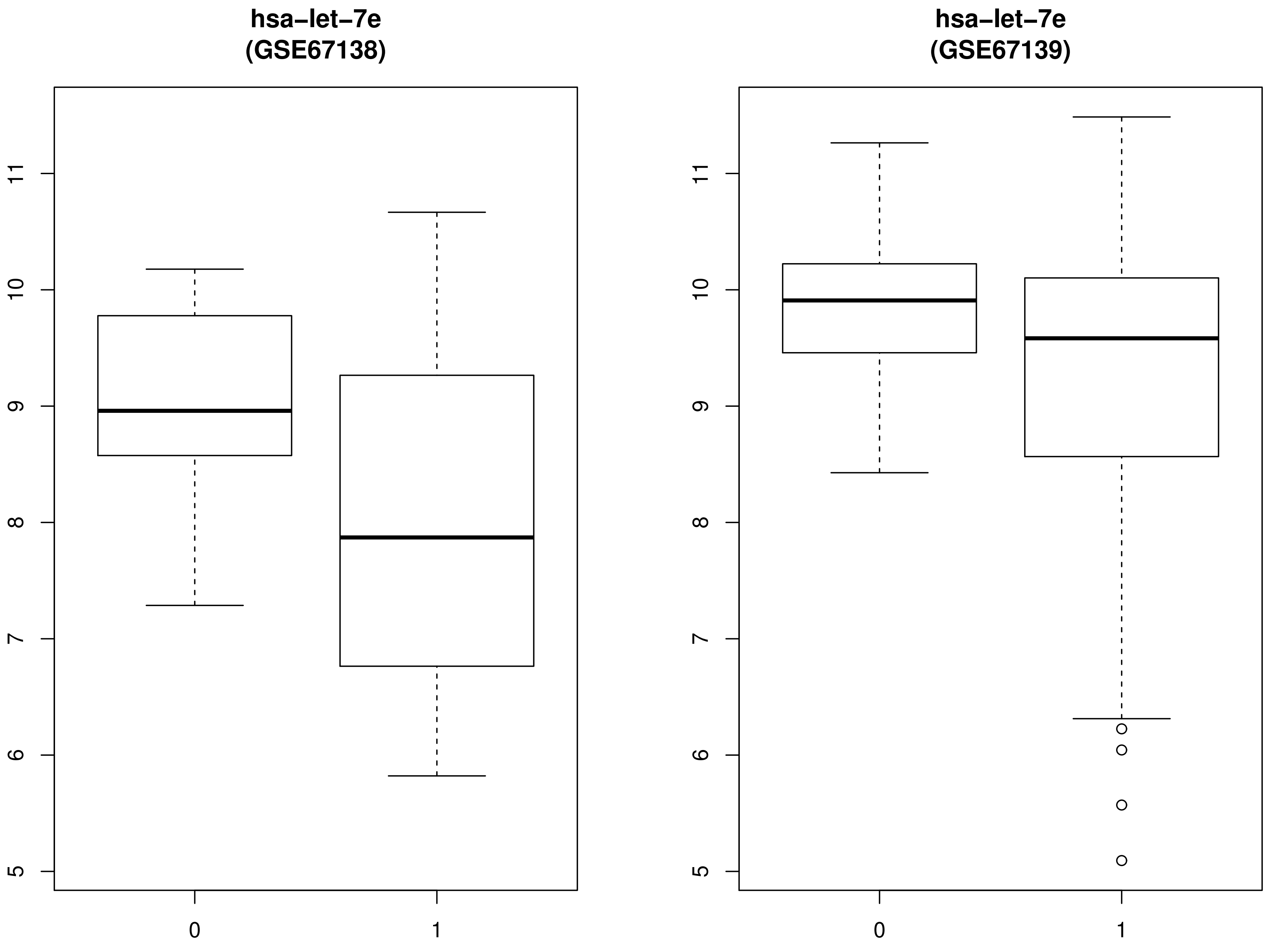
hsa−let−7e
(GSE67138)
hsa−let−7e
(GSE67139)
5
6
7
8
9
10
11
0
1

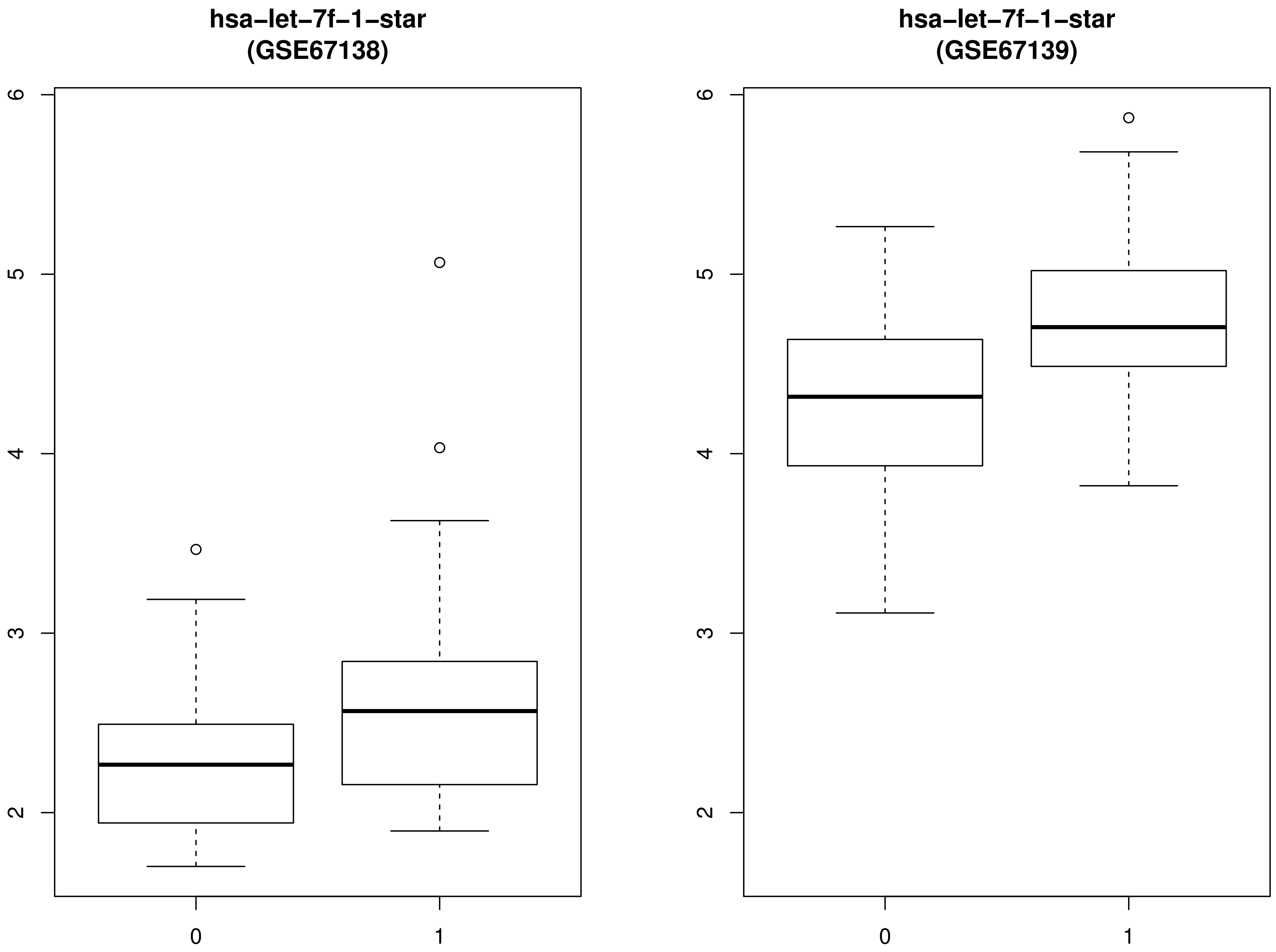
hsa−let−7f−1−star
(GSE67138)
hsa−let−7f−1−star
(GSE67139)
2
3
4
5
6
0
1

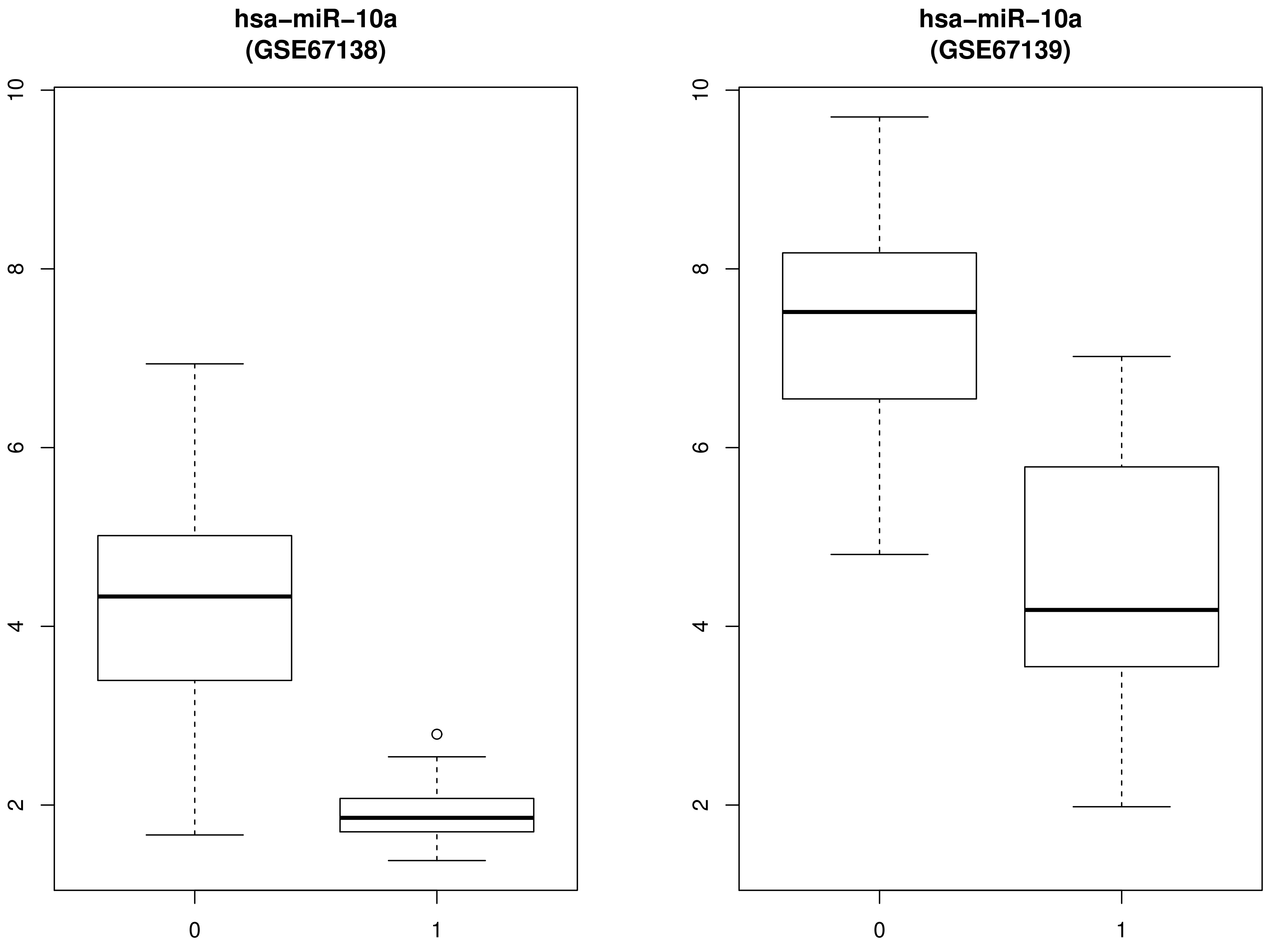
hsa−miR−10a
(GSE67138)
hsa−miR−10a
(GSE67139)
10
8
6
4
2
0
1

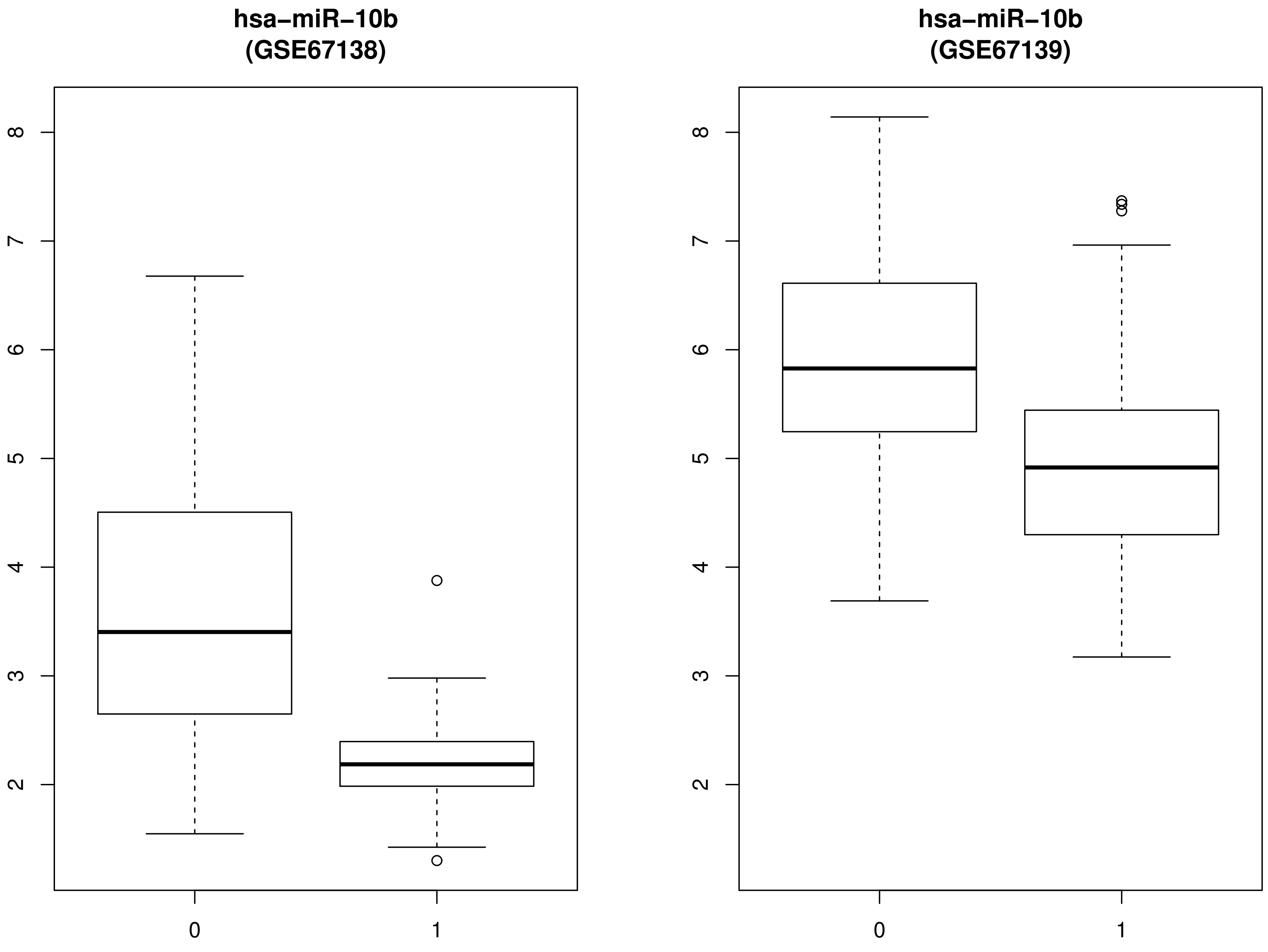

hsa−miR−10b
(GSE67138)
hsa−miR−10b
(GSE67139)
2
3
4
5
6
7
8
0
1

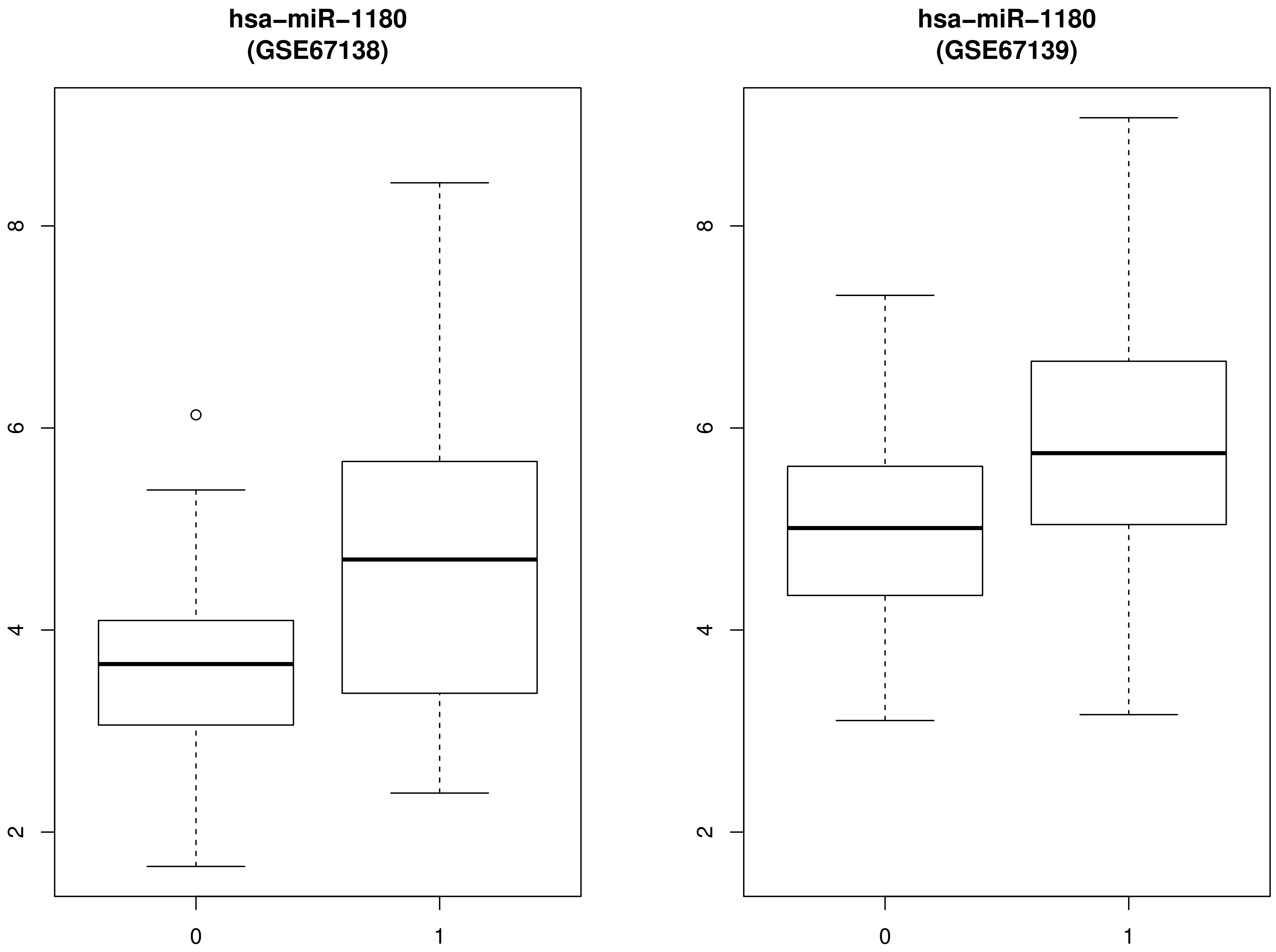
hsa−miR−1180
(GSE67138)
hsa−miR−1180
(GSE67139)
2
4
6
8
0
1
2
4
6
8
0
1

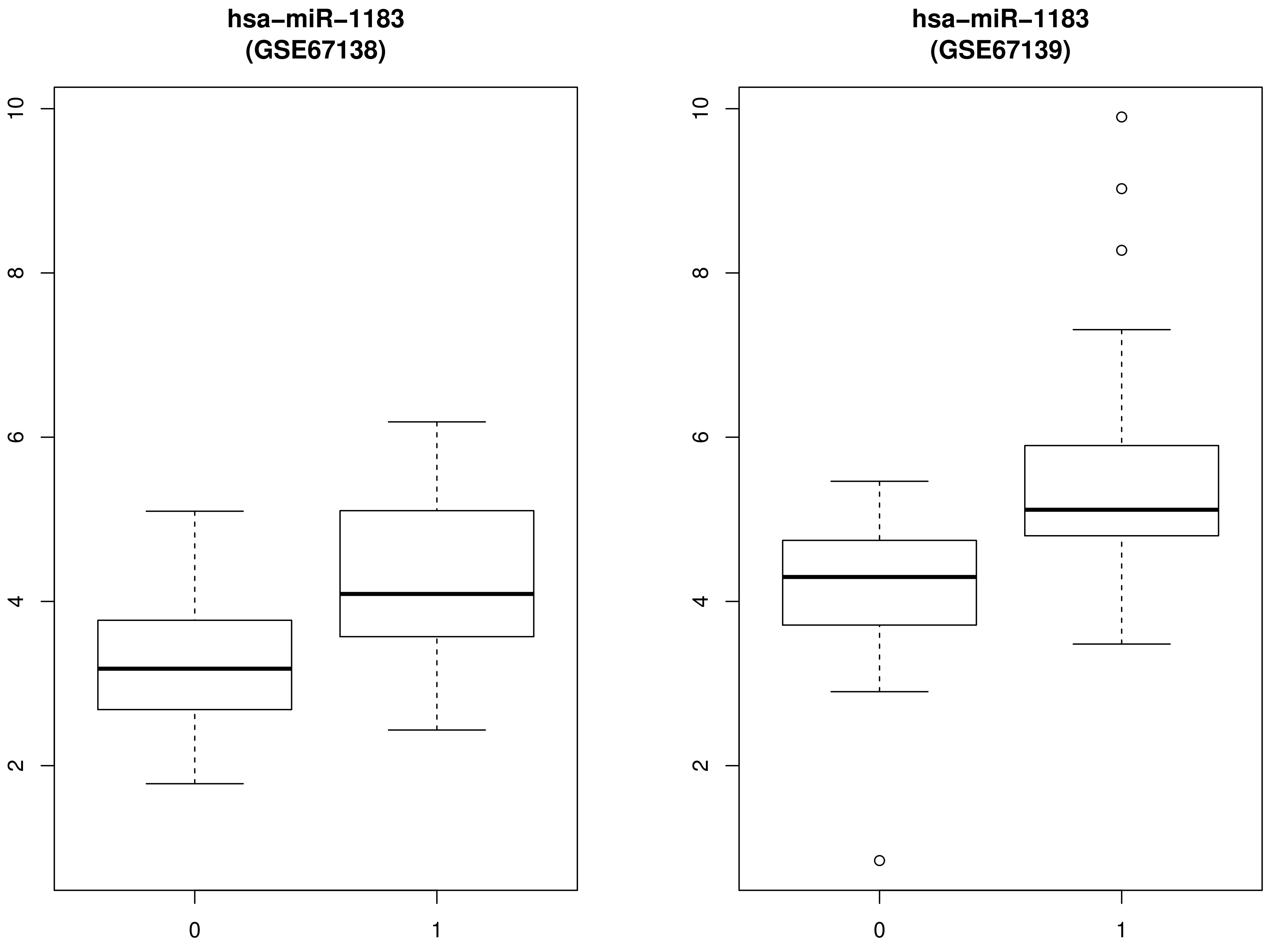
hsa−miR−1183
(GSE67138)
hsa−miR−1183
(GSE67139)
10
8
6
4
2
0
1

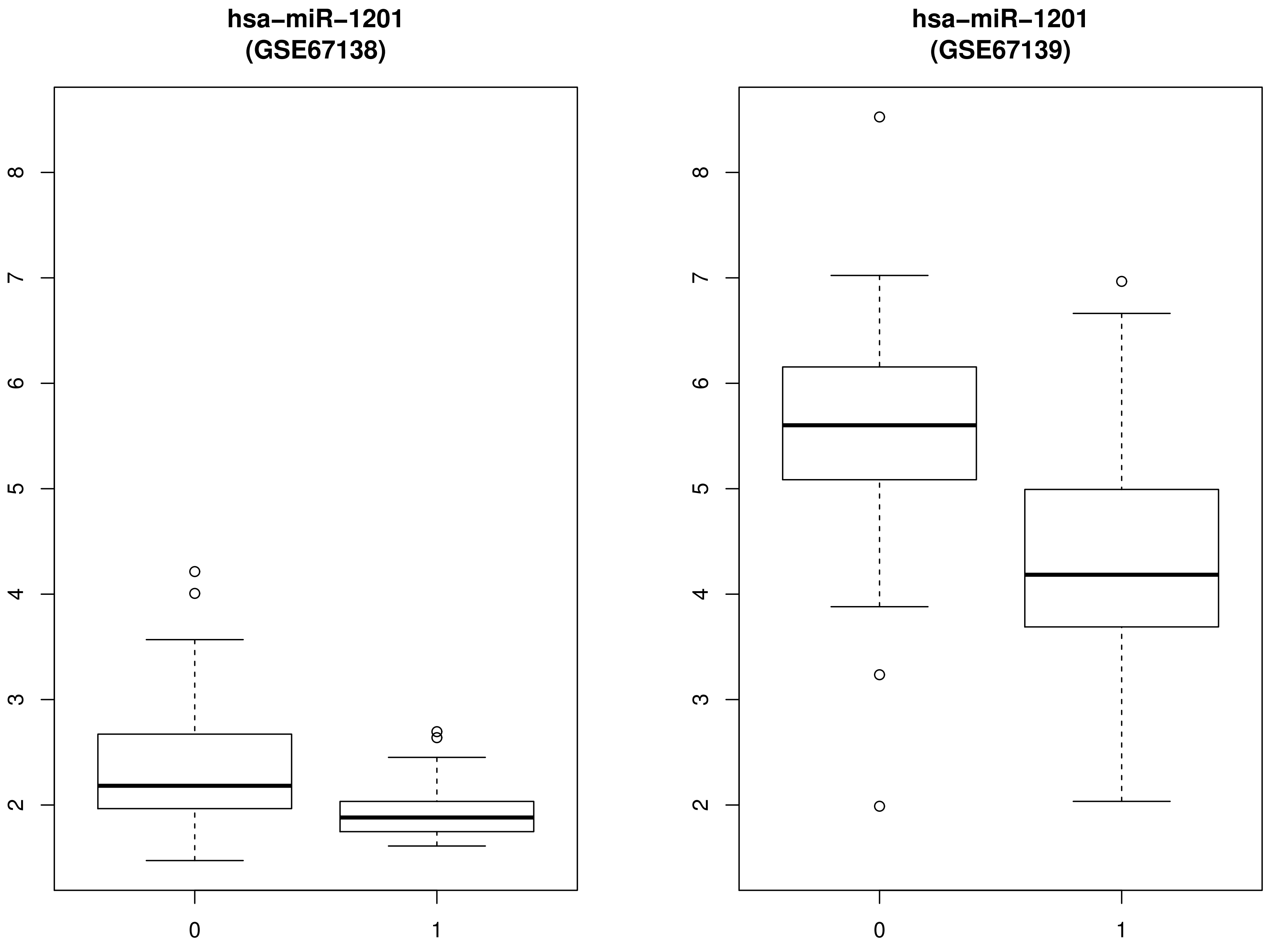
hsa−miR−1201
(GSE67138)
hsa−miR−1201
(GSE67139)
2
3
4
5
6
7
8
0
1

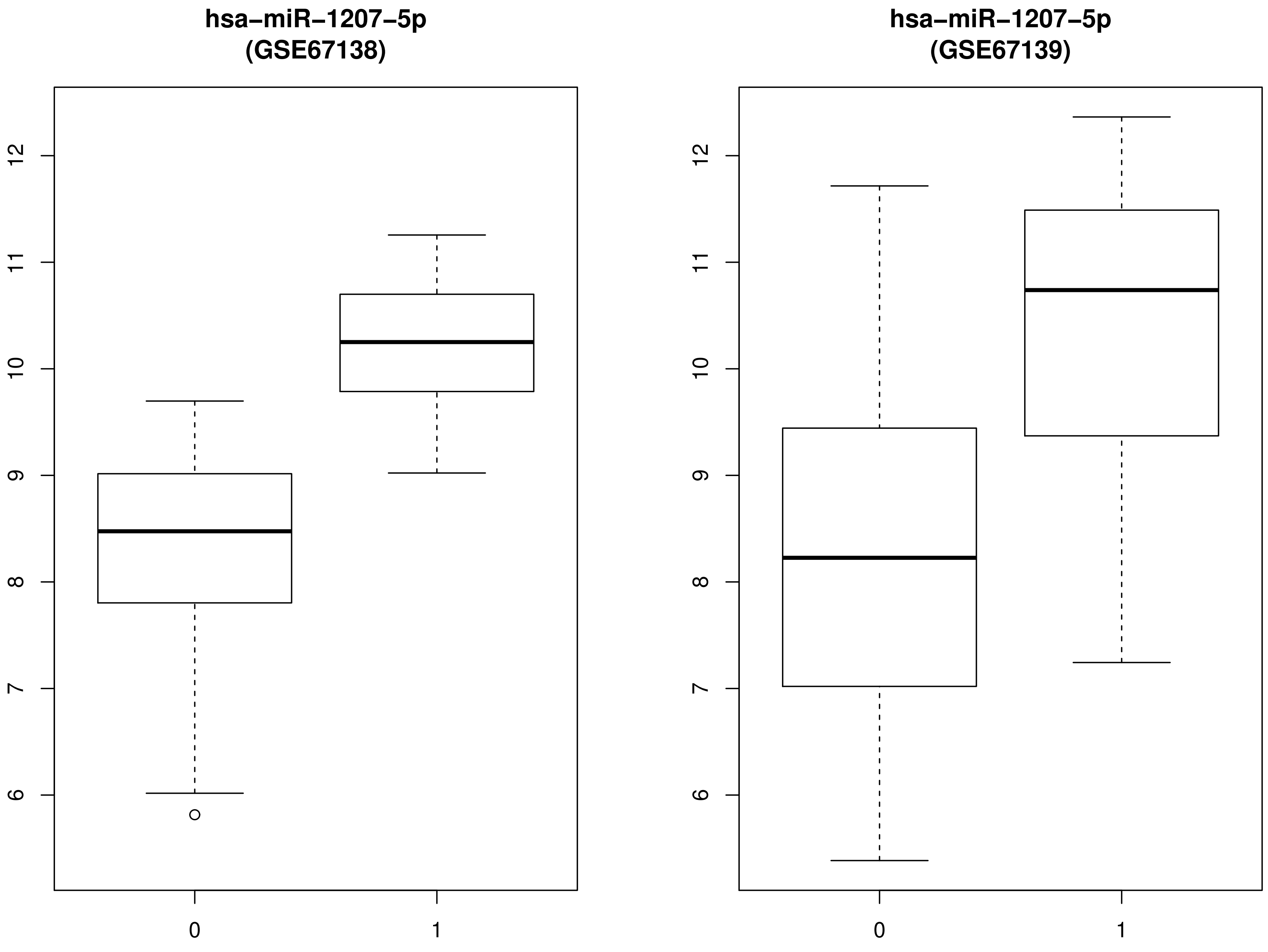
hsa−miR−1207−5p
(GSE67138)
12
11
10
9
8
7
6
0
1
hsa−miR−1207−5p
(GSE67139)
12
11
10
9
8
7
6
0
1

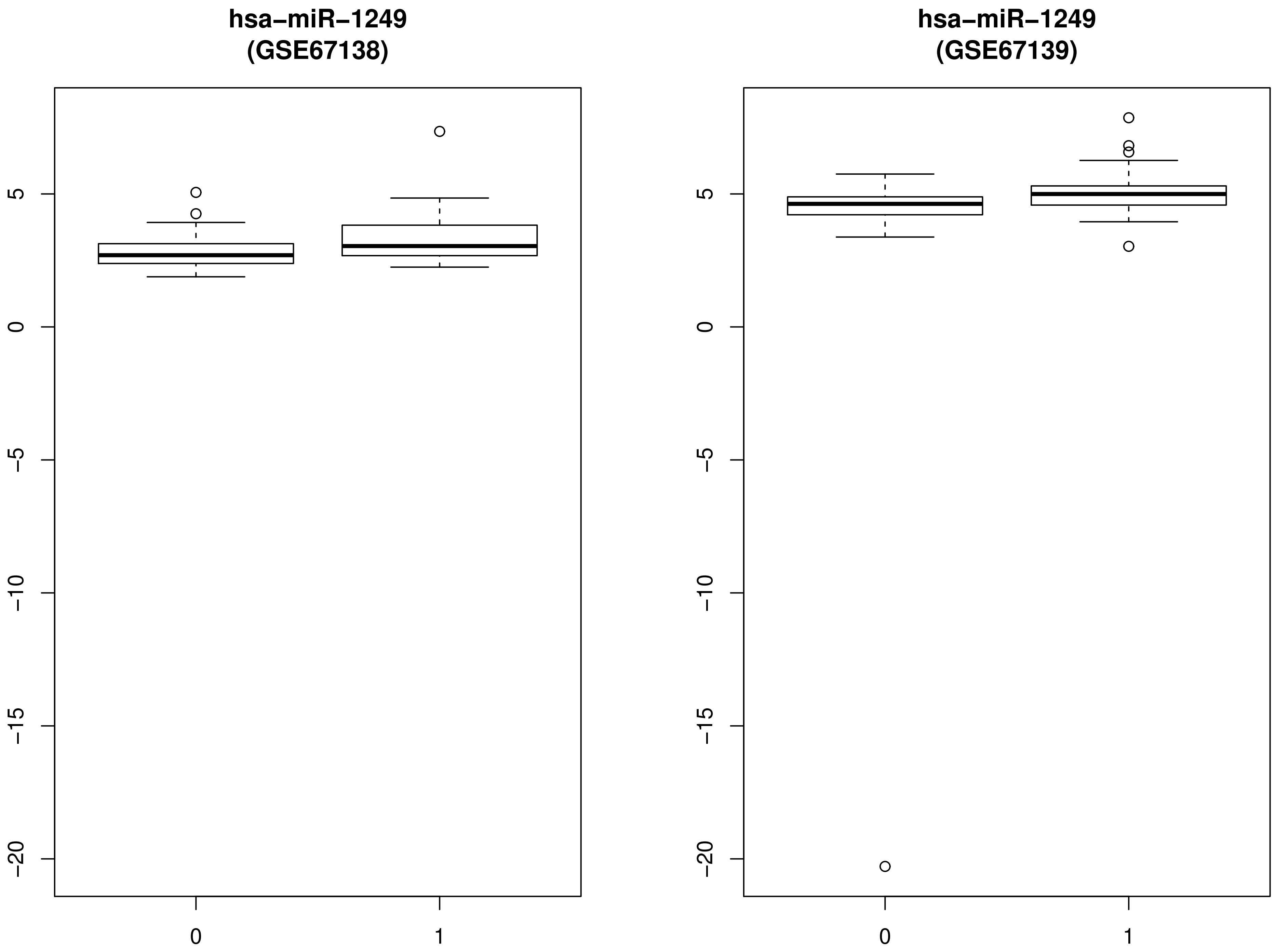
hsa−miR−1249
(GSE67138)
hsa−miR−1249
(GSE67139)
5
0
−5
−10
−15
−20
0
1

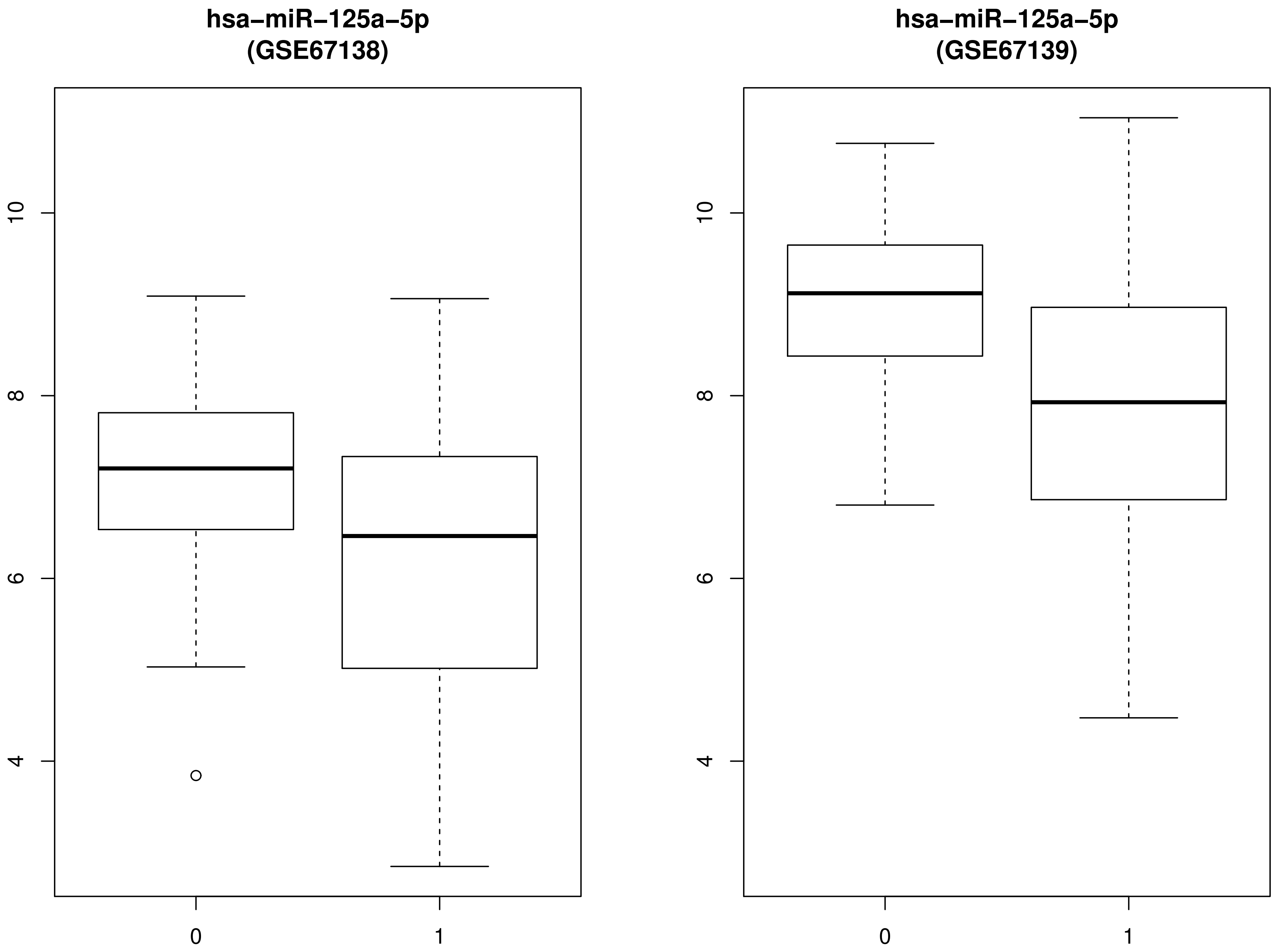
hsa−miR−125a−5p
(GSE67138)
hsa−miR−125a−5p
(GSE67139)
4
6
8
10
0
1

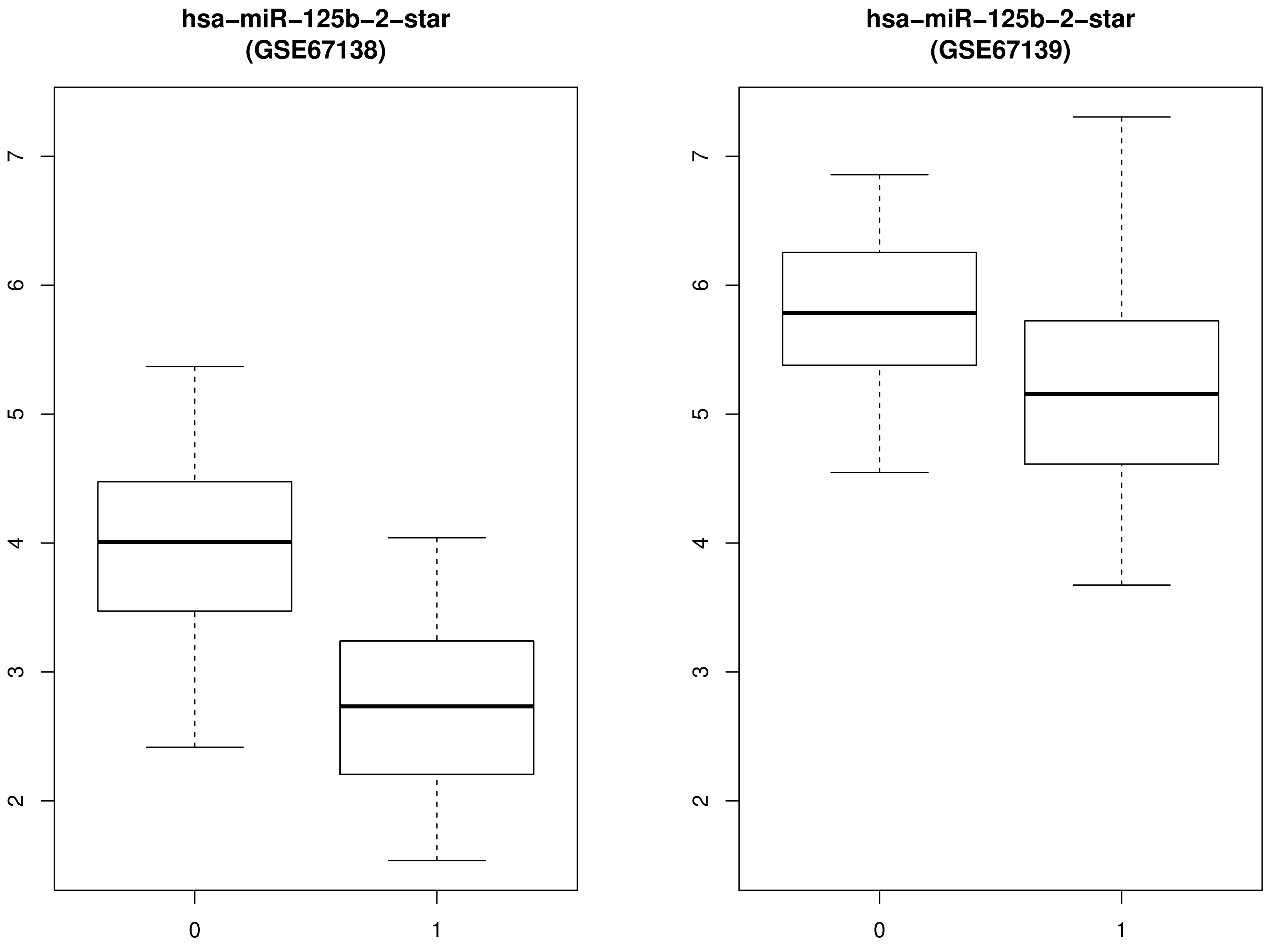
hsa−miR−125b−2−star
(GSE67138)
hsa−miR−125b−2−star
(GSE67139)
2
3
4
5
6
7
0
1

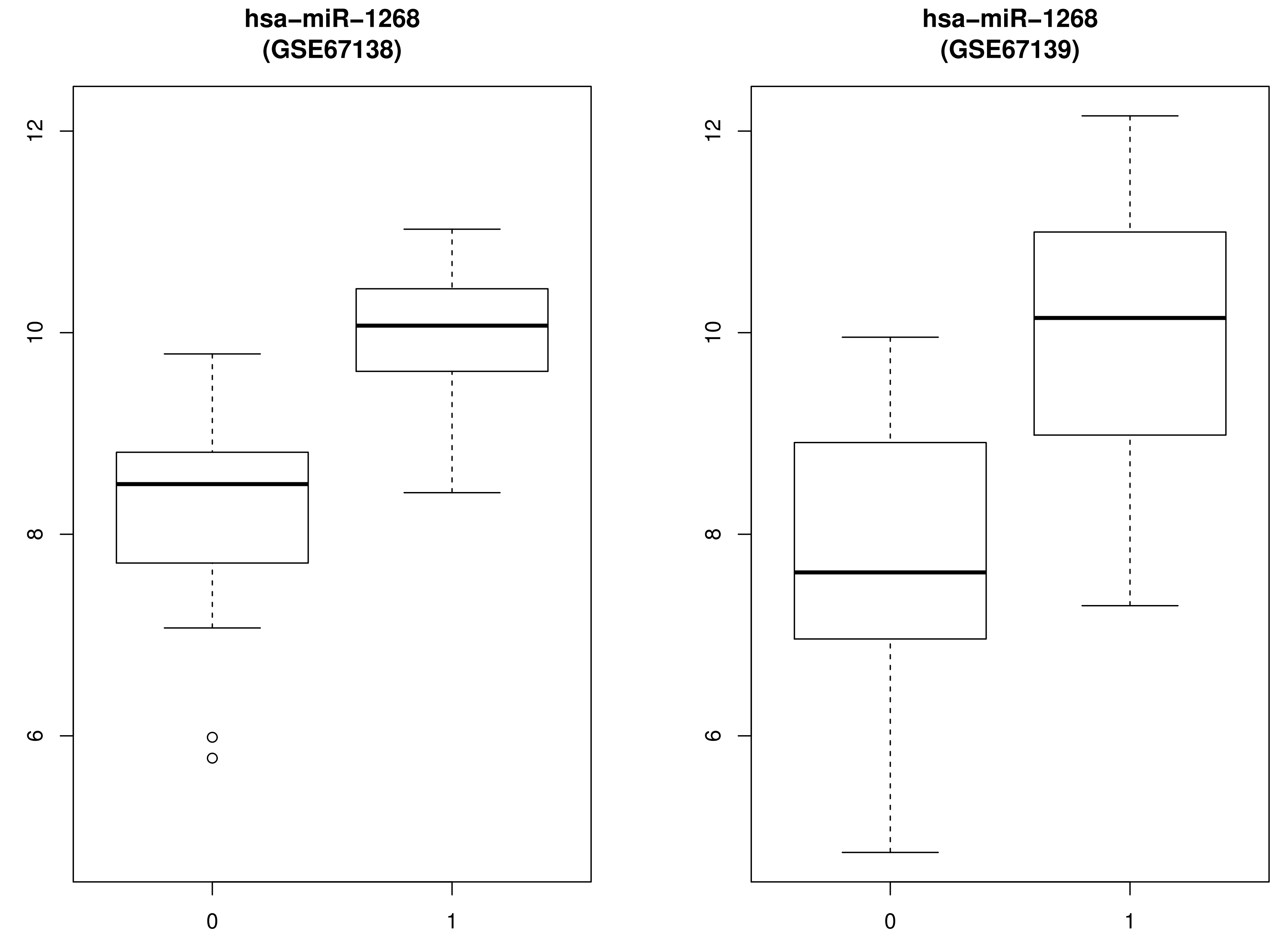
hsa−miR−1268
(GSE67138)
hsa−miR−1268
(GSE67139)
12
10
8
6
0
1
12
10
8
6
0
1

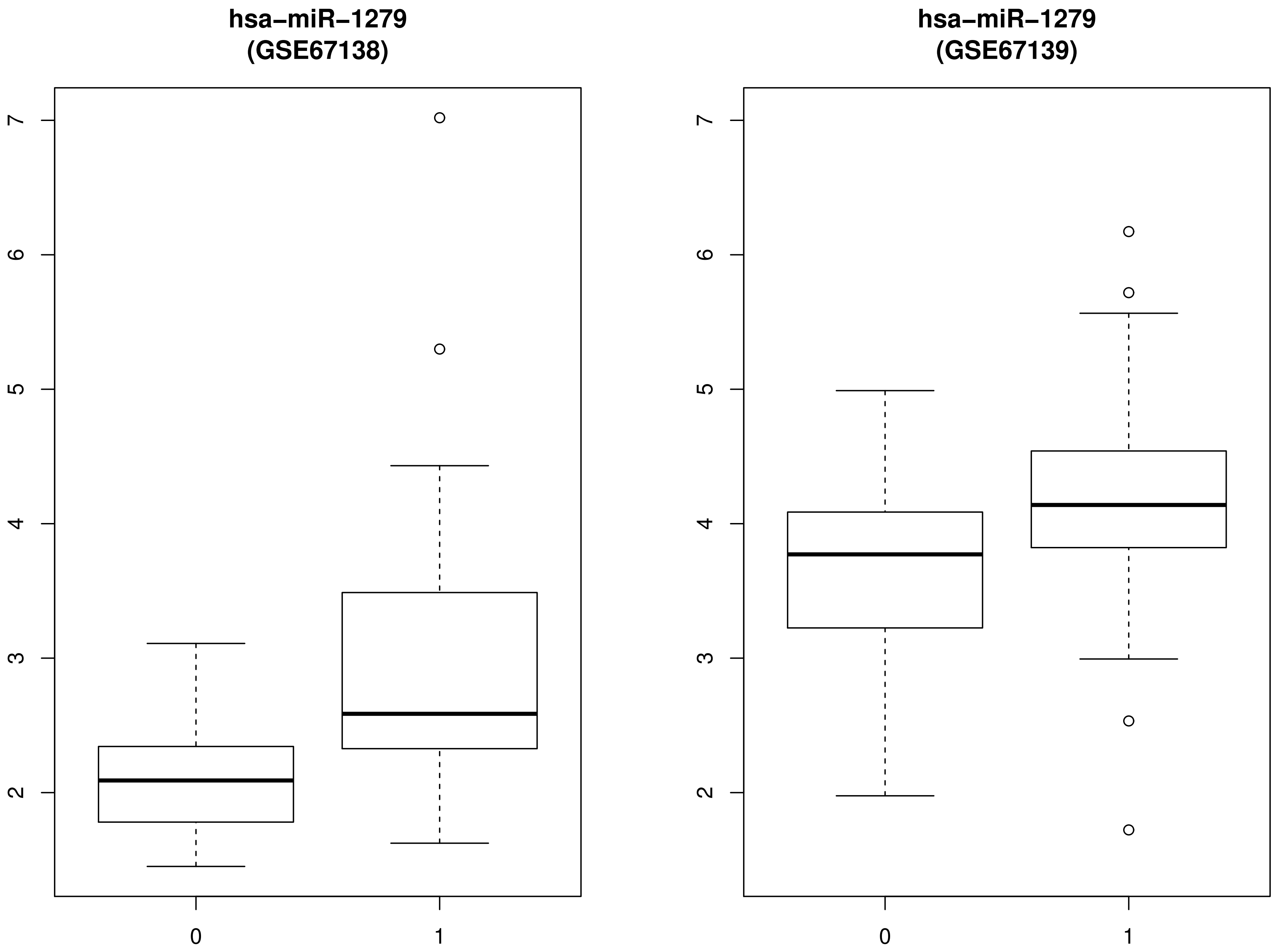

hsa−miR−1279
(GSE67138)
7
6
5
4
3
2
0
1
hsa−miR−1279
(GSE67139)
7
6
5
4
3
2
0
1

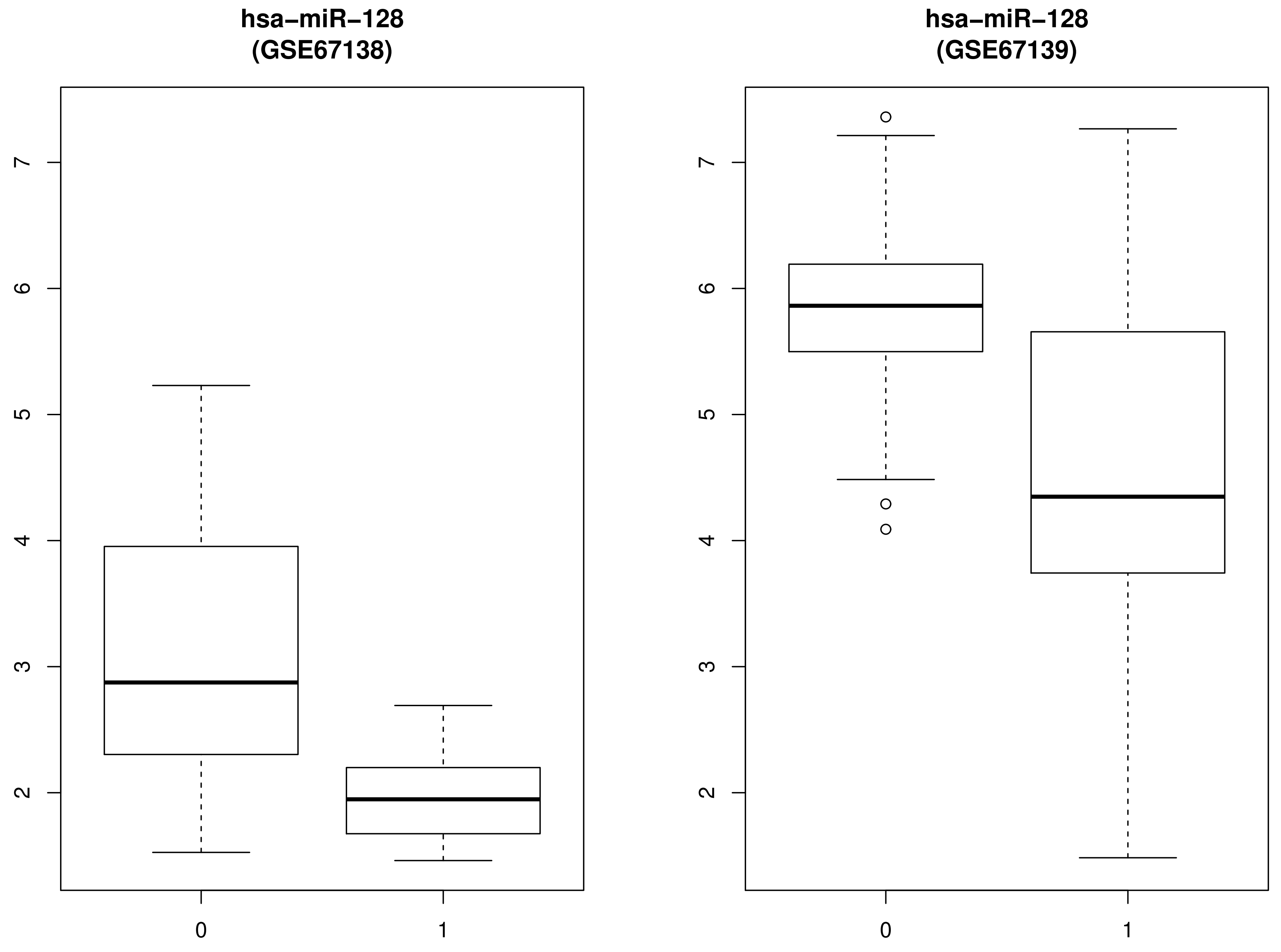
hsa−miR−128
(GSE67138)
hsa−miR−128
(GSE67139)
2
3
4
5
6
7
0
1

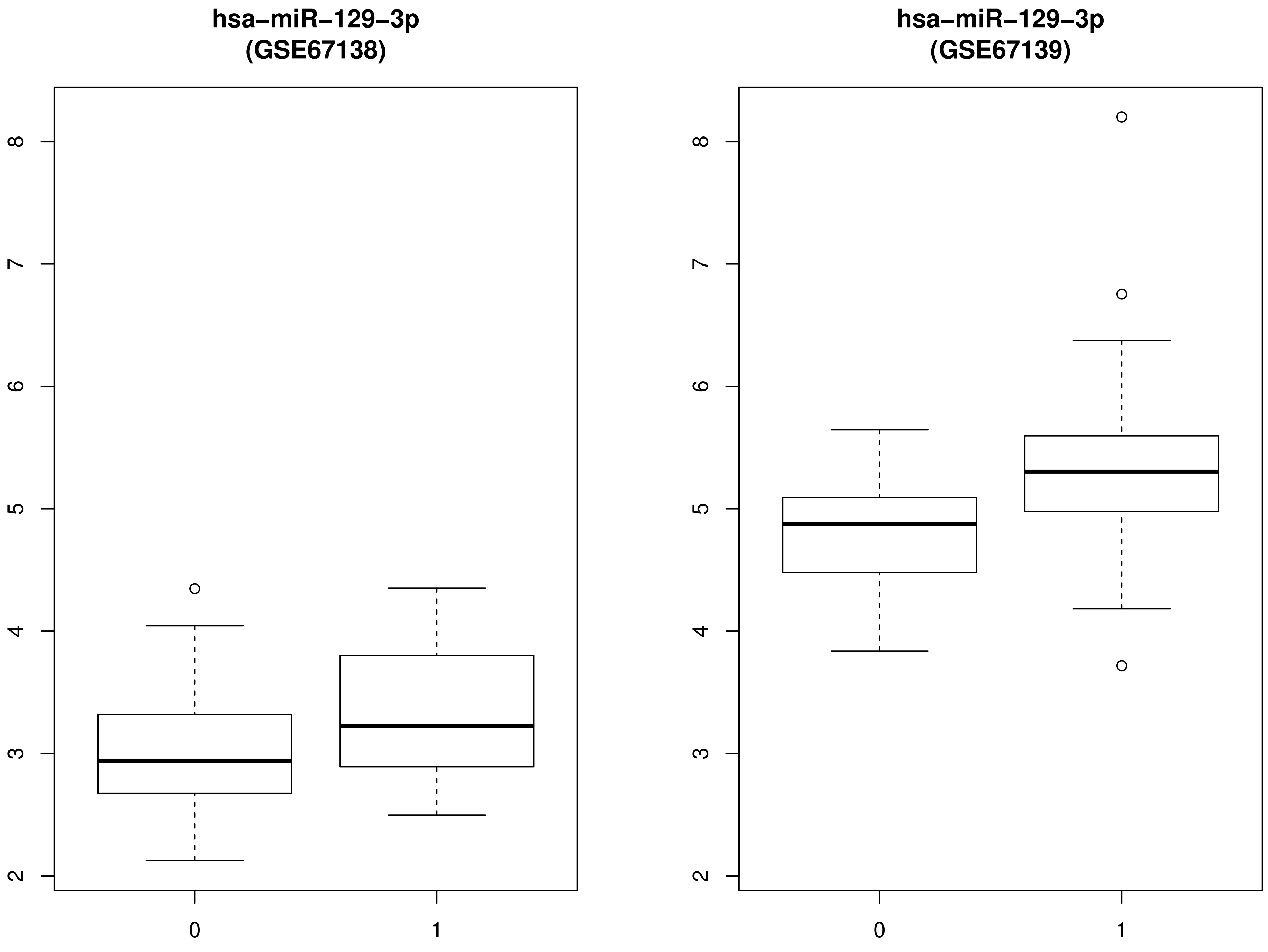
hsa−miR−129−3p
(GSE67138)
hsa−miR−129−3p
(GSE67139)
2
3
4
5
6
7
8
0
1

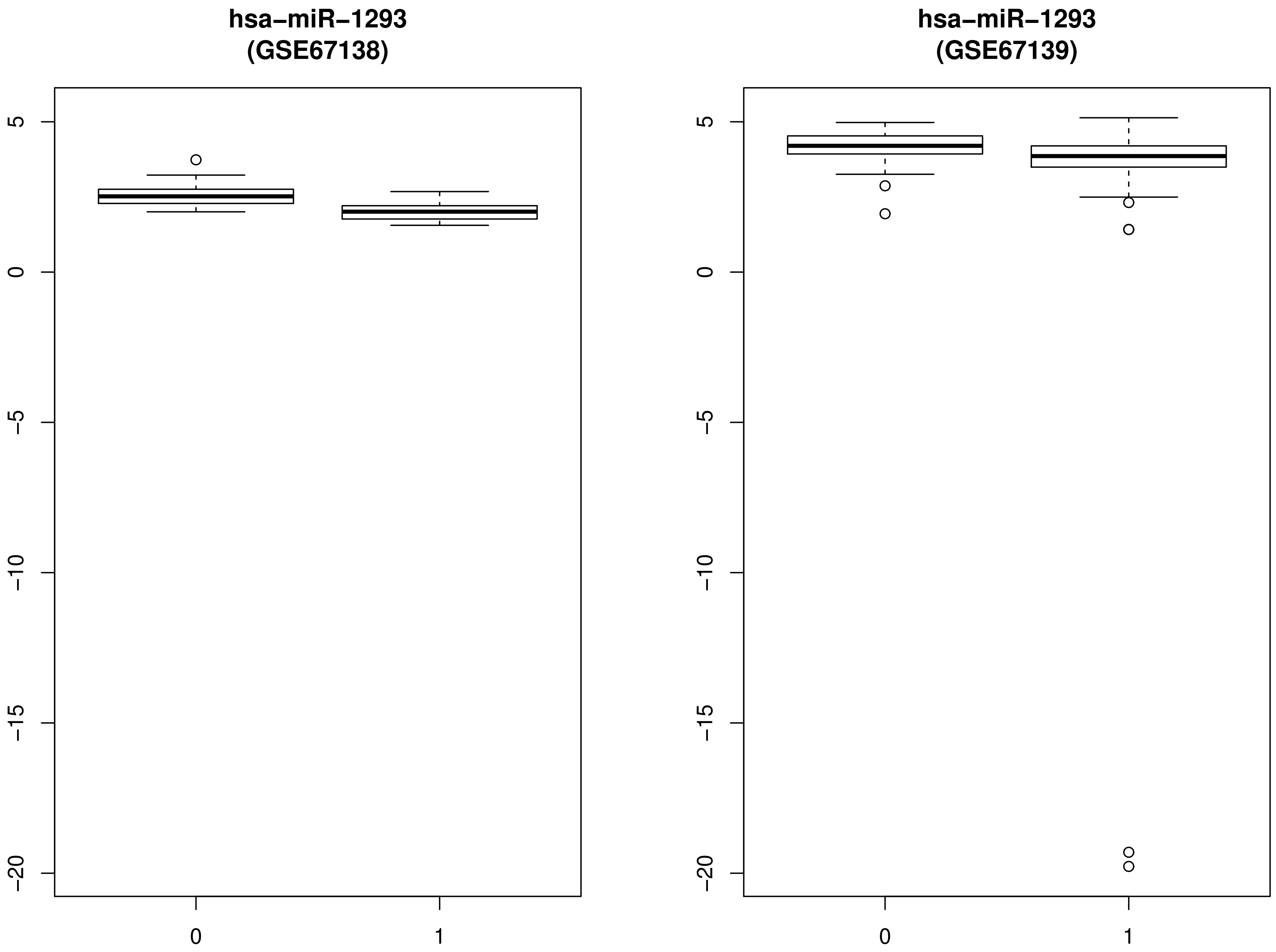
hsa−miR−1293
(GSE67138)
5
0
−5
−10
−15
−20
0
1
hsa−miR−1293
(GSE67139)
5
0
−5
−10
−15
−20
0
1

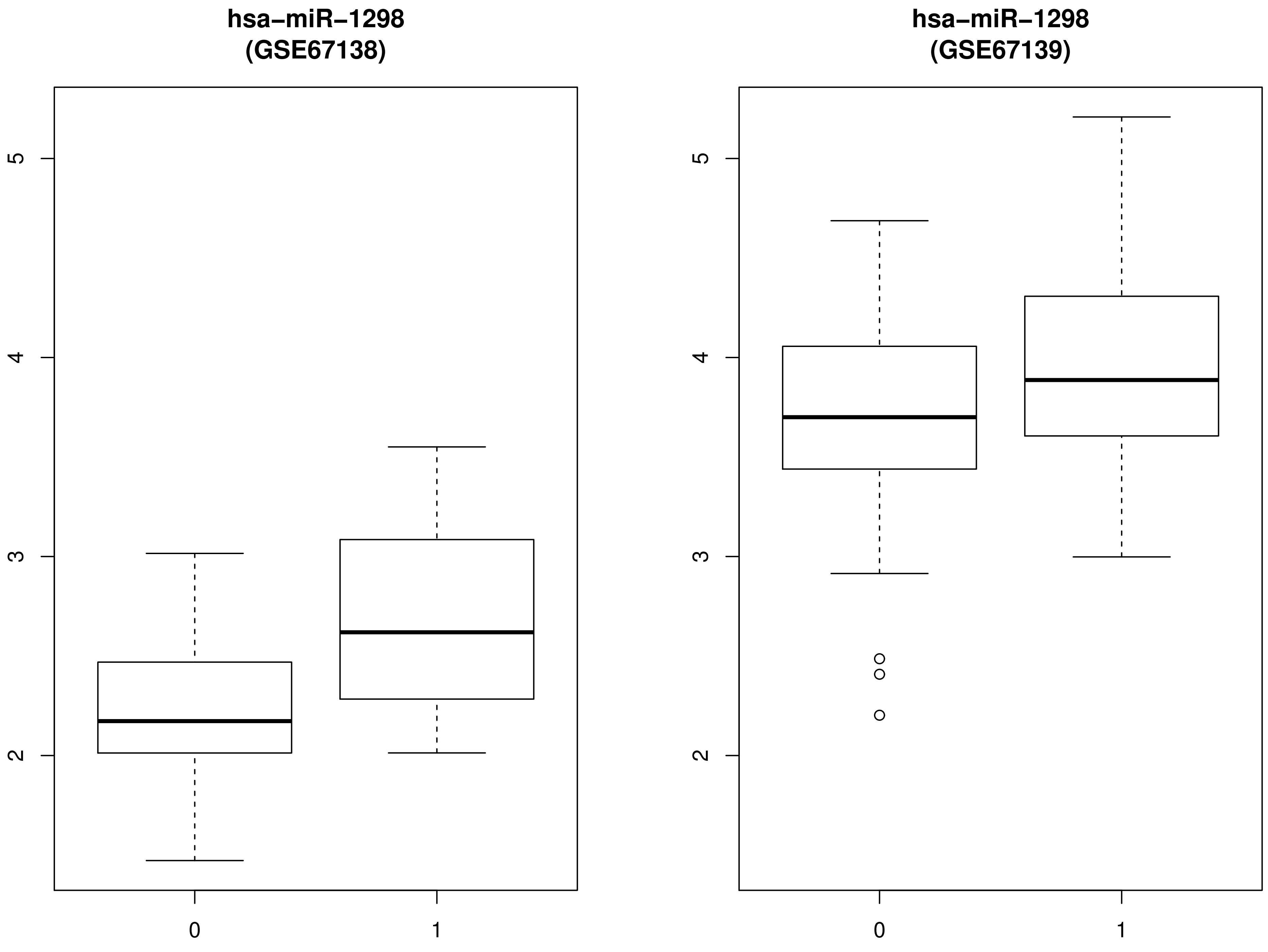
hsa−miR−1298
(GSE67138)
5
4
3
2
0
1
hsa−miR−1298
(GSE67139)
5
4
3
2
0
1

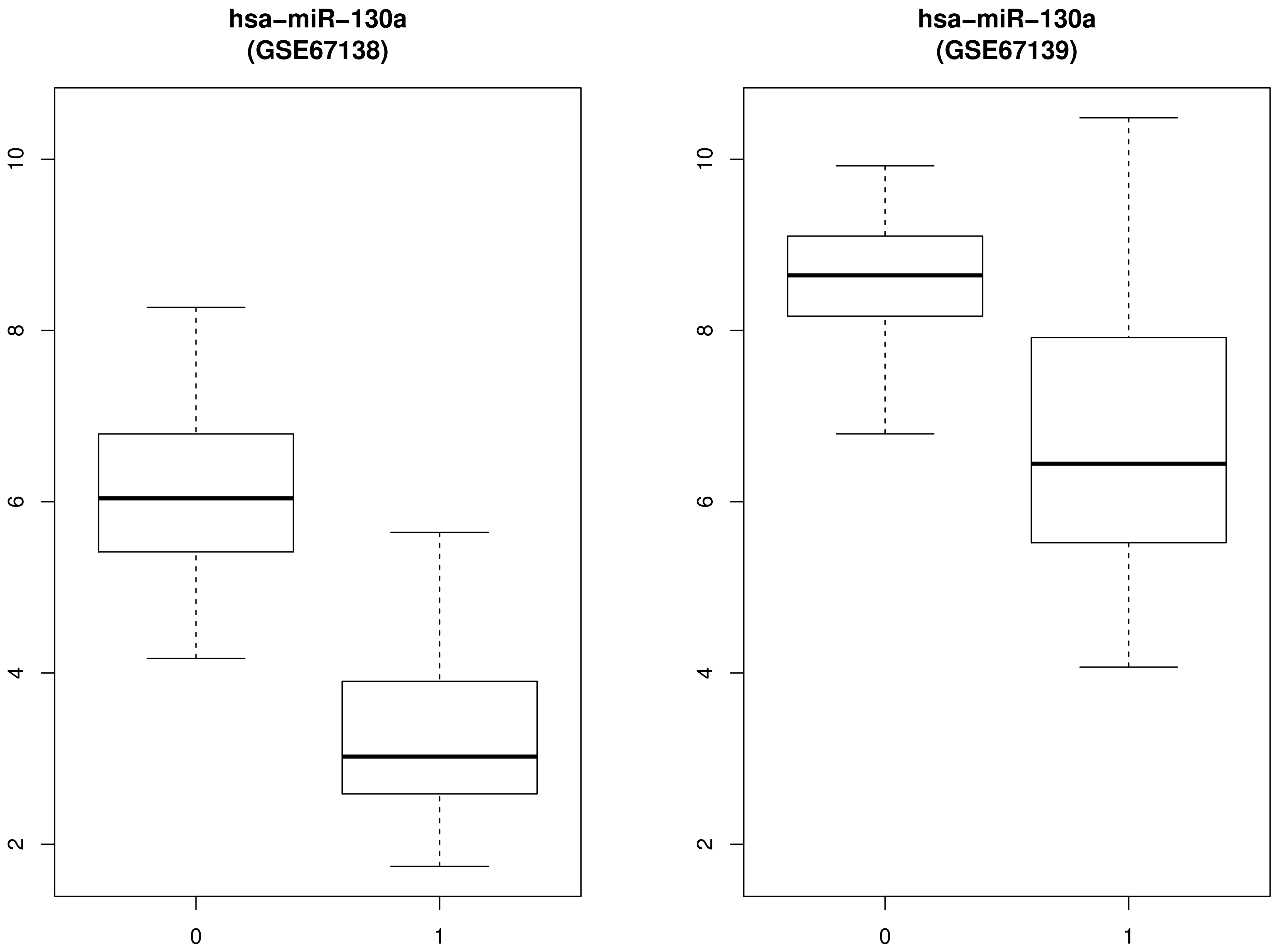
hsa−miR−130a
(GSE67138)
hsa−miR−130a
(GSE67139)
2
4
6
8
10
0
1

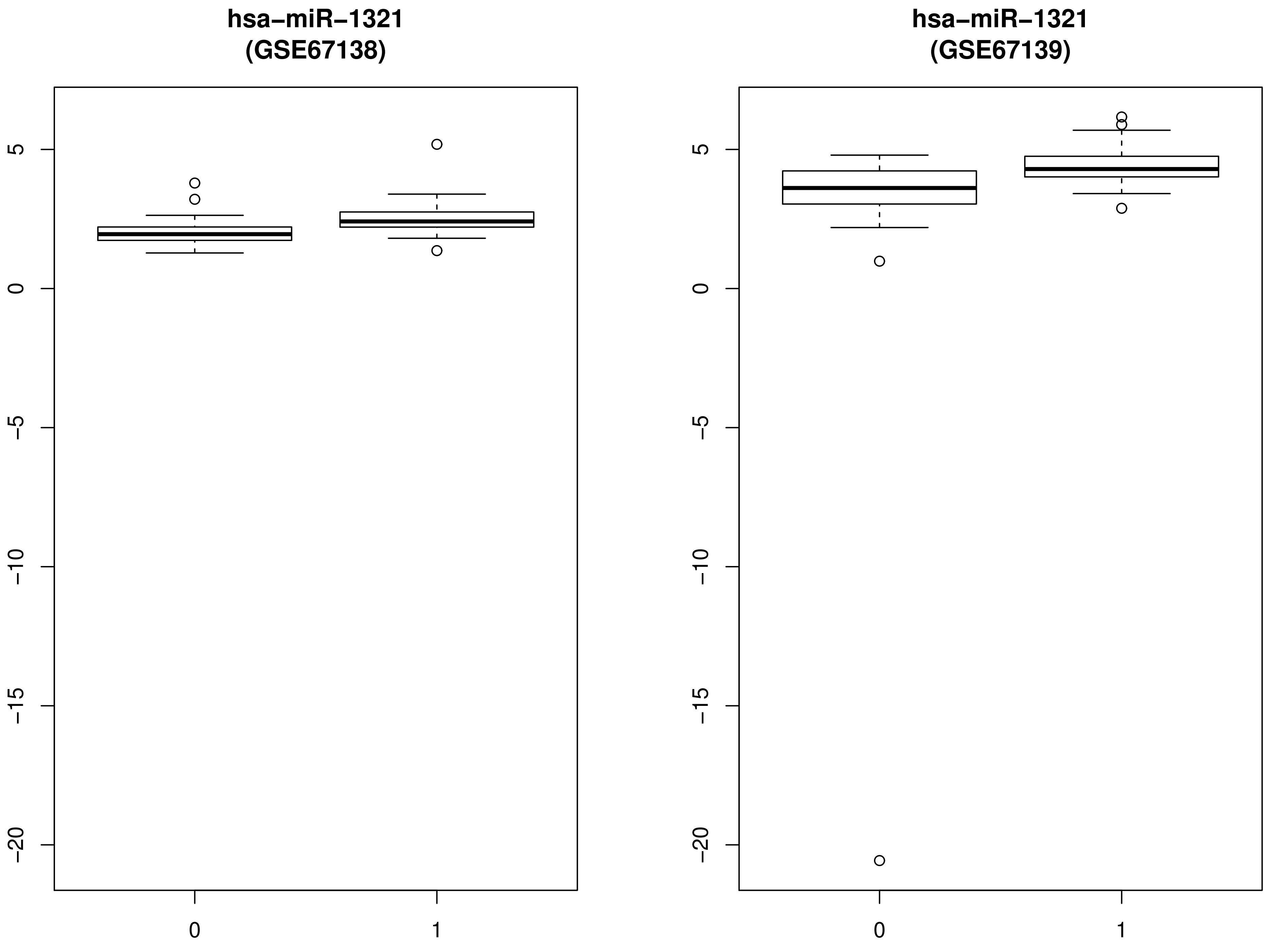
hsa−miR−1321
(GSE67138)
hsa−miR−1321
(GSE67139)
5
0
−5
−10
−15
−20
0
1
5
0
−5
−10
−15
−20
0
1

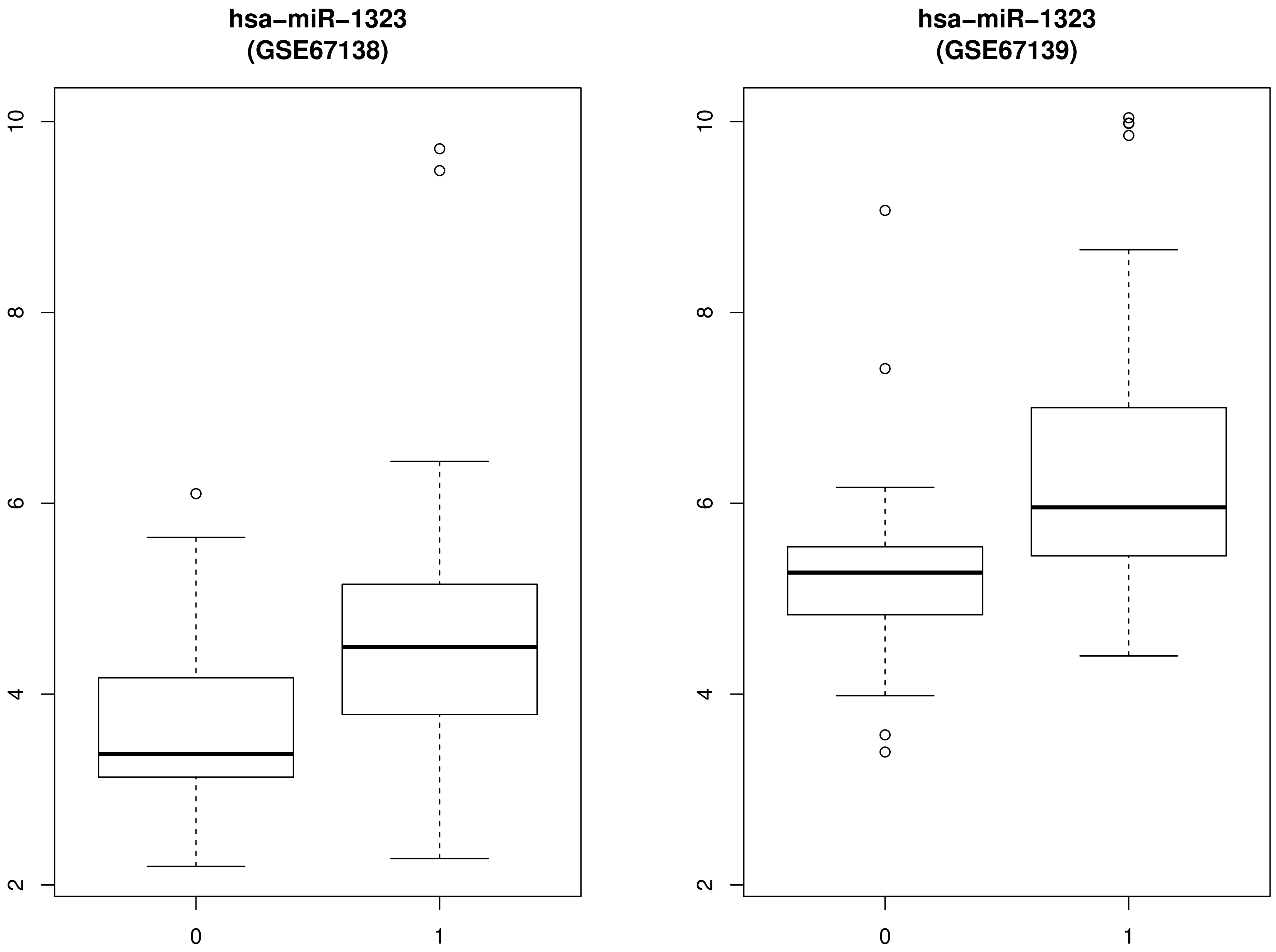
hsa-miR-1323
(GSE67138)
hsa-miR-1323
(GSE67139)
2
4
6
8
10
0
1

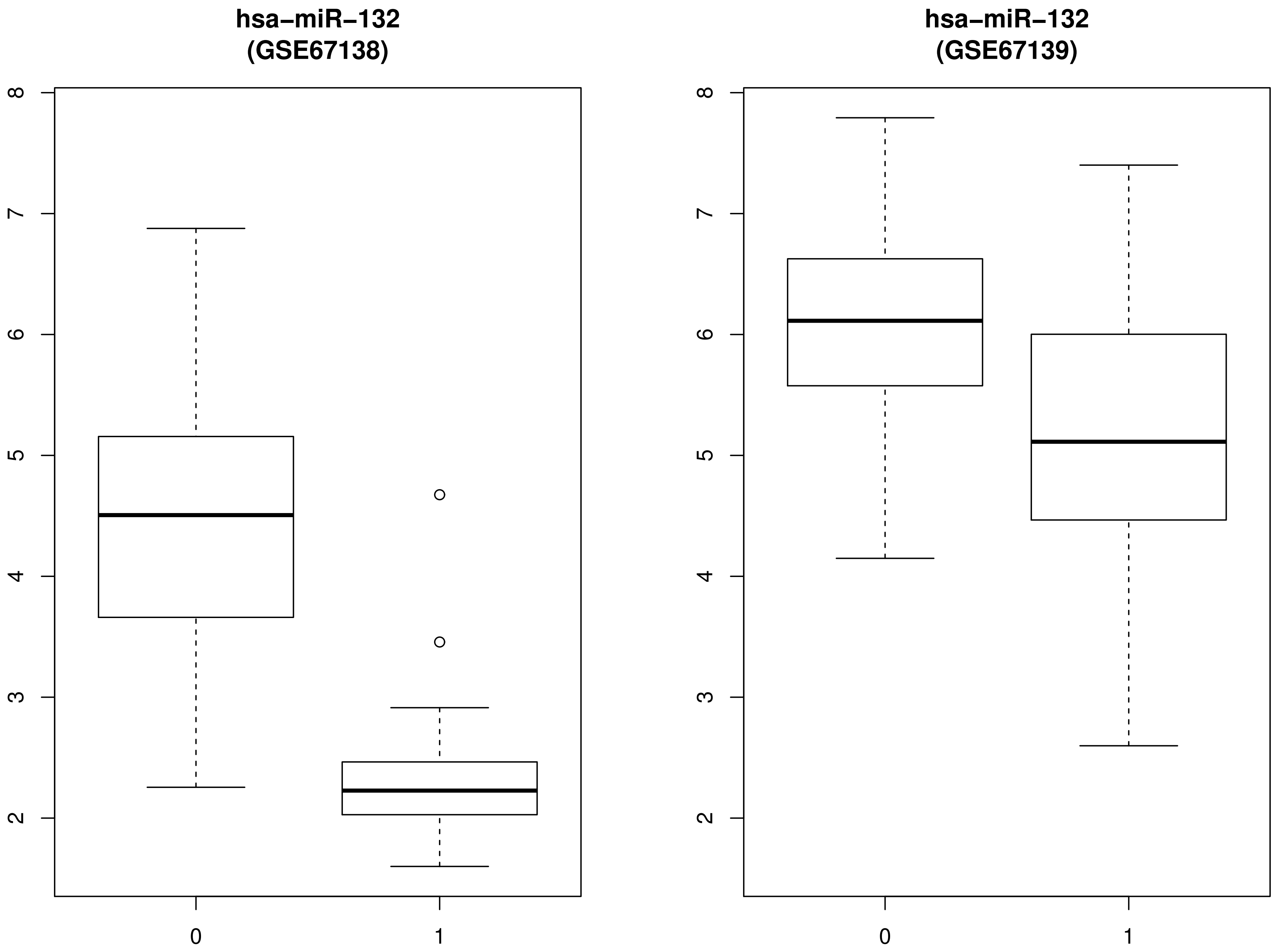
hsa−miR−132
(GSE67138)
hsa−miR−132
(GSE67139)
2
3
4
5
6
7
8
0
1

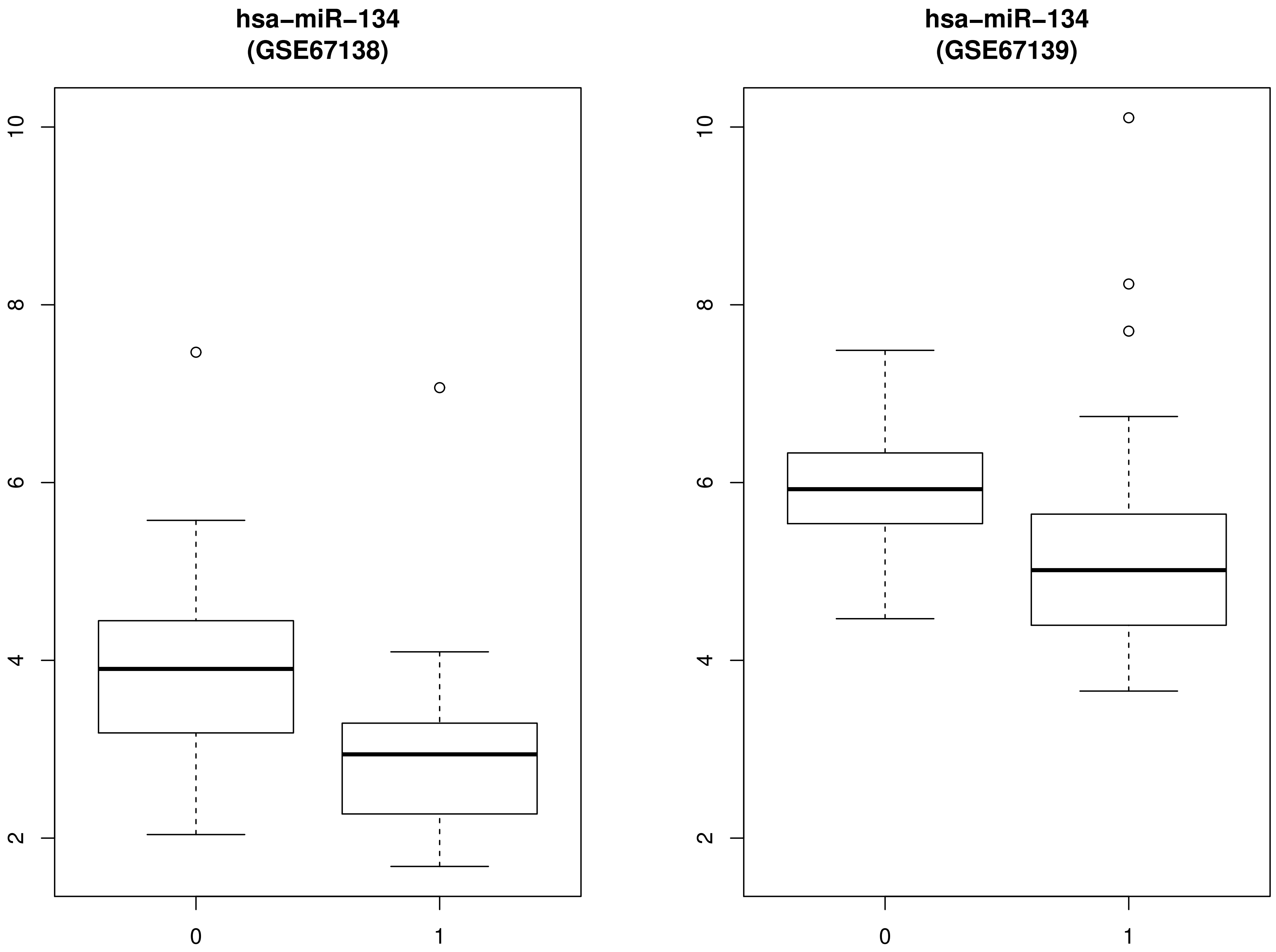
hsa−miR−134
(GSE67138)
hsa−miR−134
(GSE67139)
2
4
6
8
10
0
1

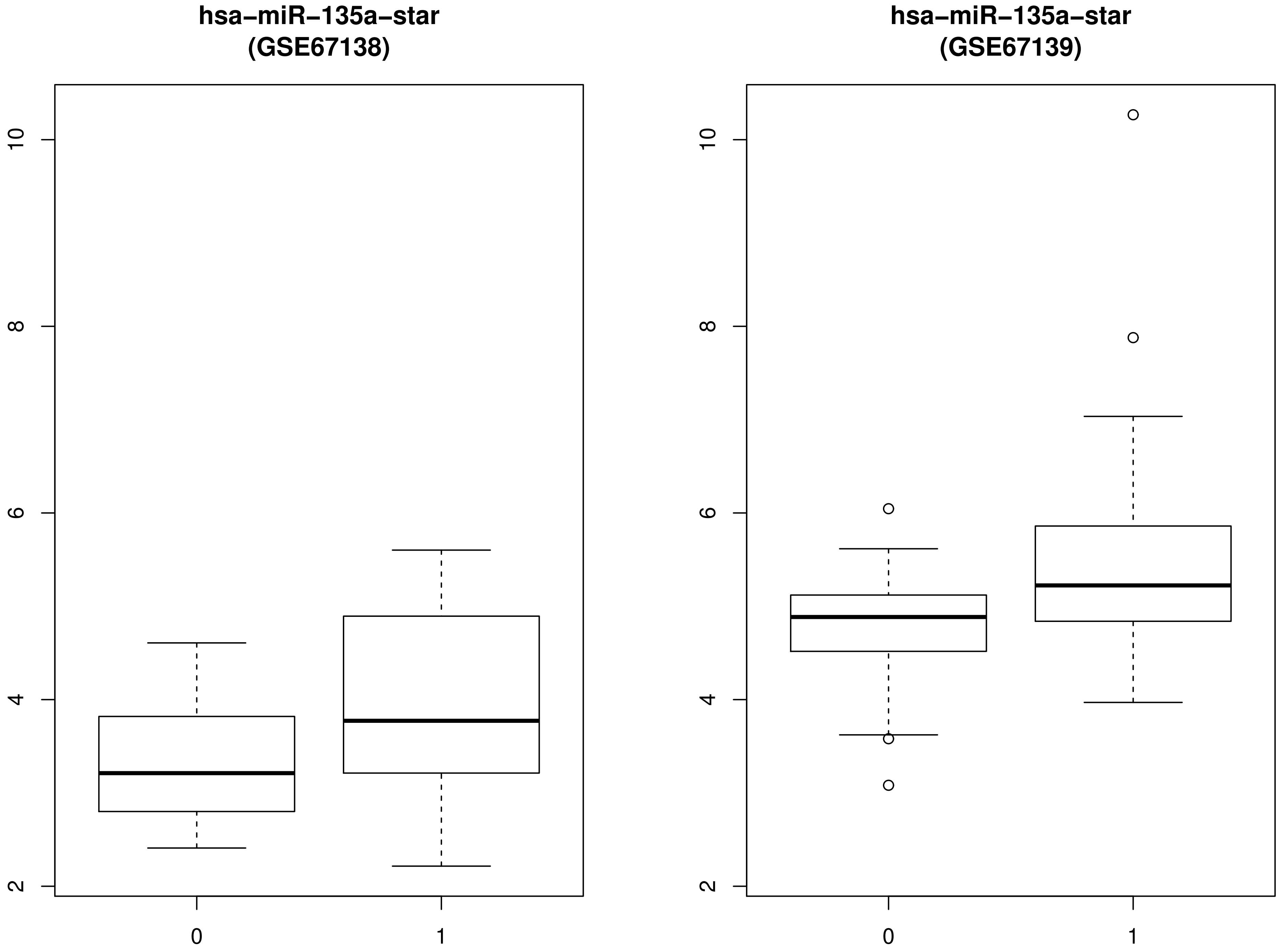

hsa−miR−135a−star
(GSE67138)
hsa−miR−135a−star
(GSE67139)
2
4
6
8
10
0
1

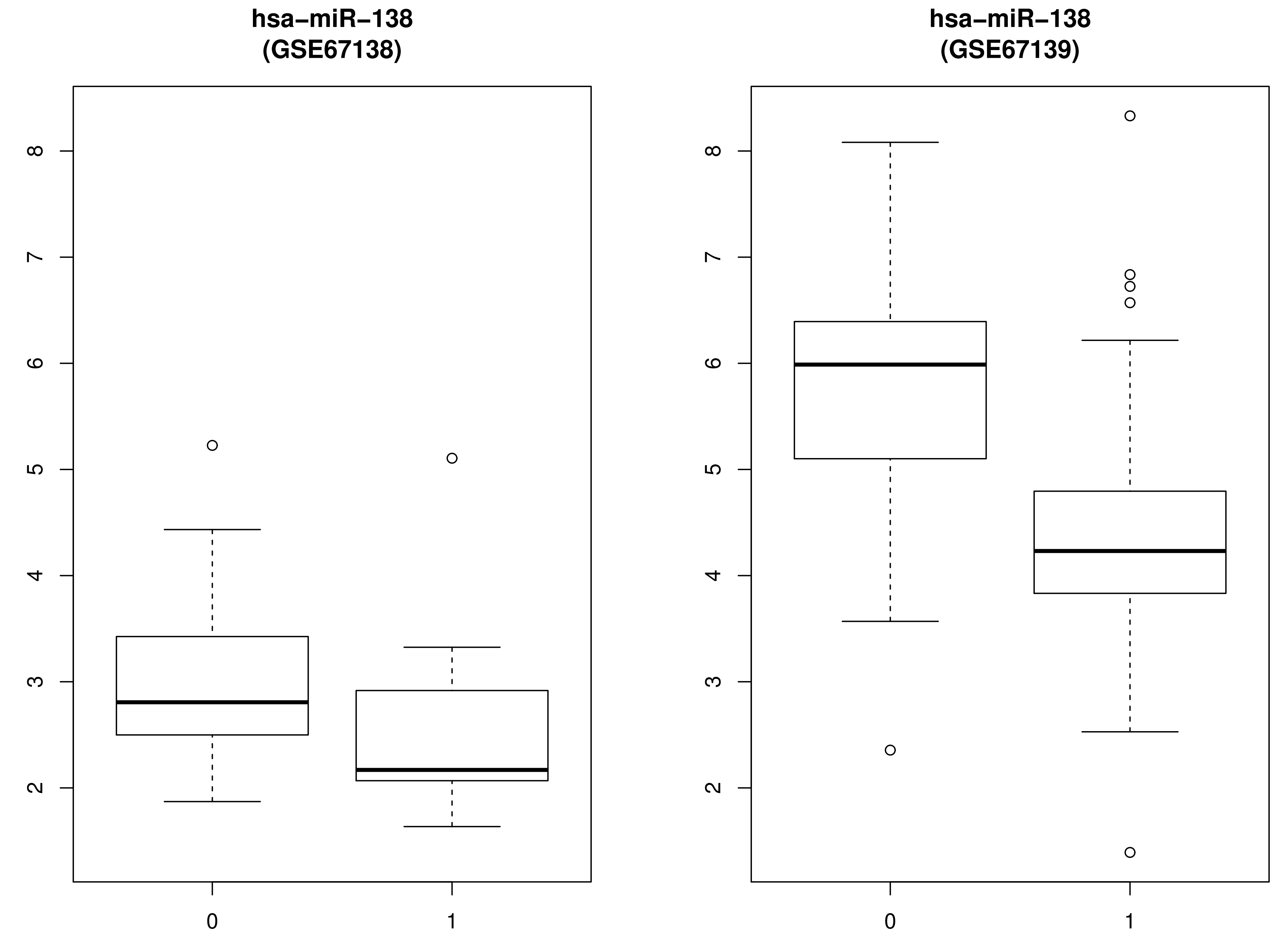
hsa–miR–138
(GSE67138)
hsa–miR–138
(GSE67139)
2
3
4
5
6
7
8
0
1

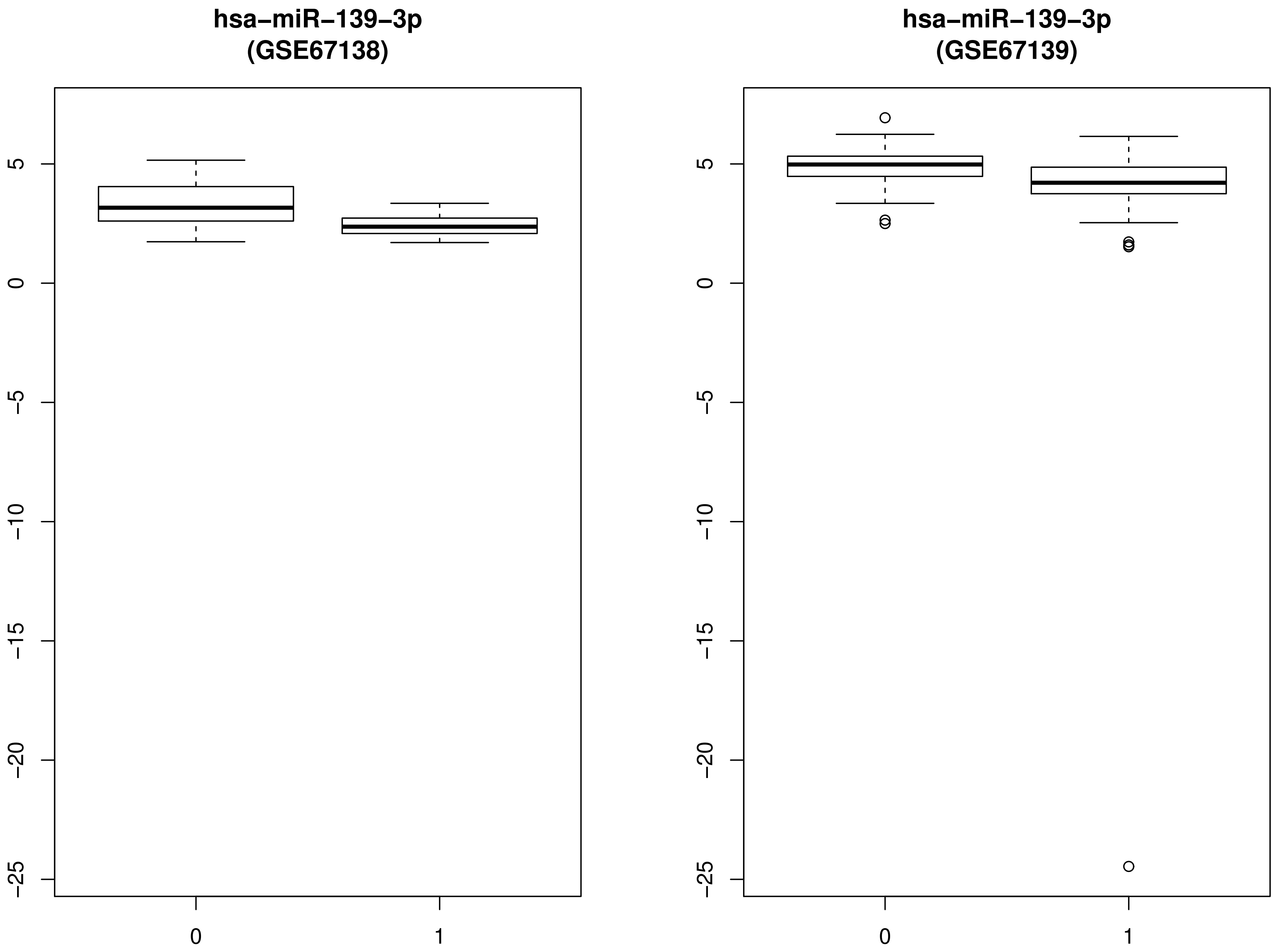

hsa−miR−139−3p
(GSE67138)
hsa−miR−139−3p
(GSE67139)
5
0
−5
−10
−15
−20
−25
0
1

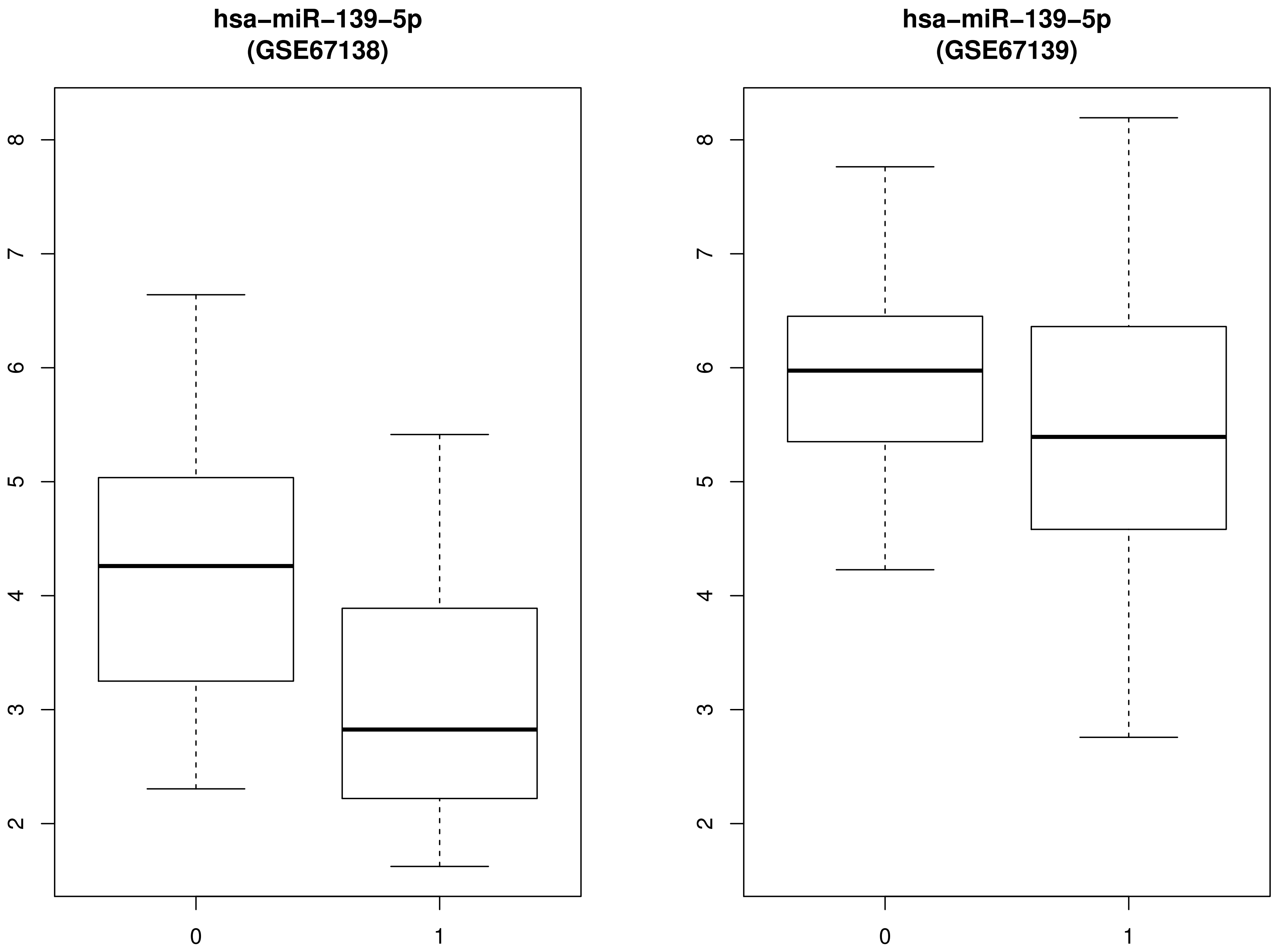
hsa−miR−139−5p
(GSE67138)
hsa−miR−139−5p
(GSE67139)
2
3
4
5
6
7
8
0
1

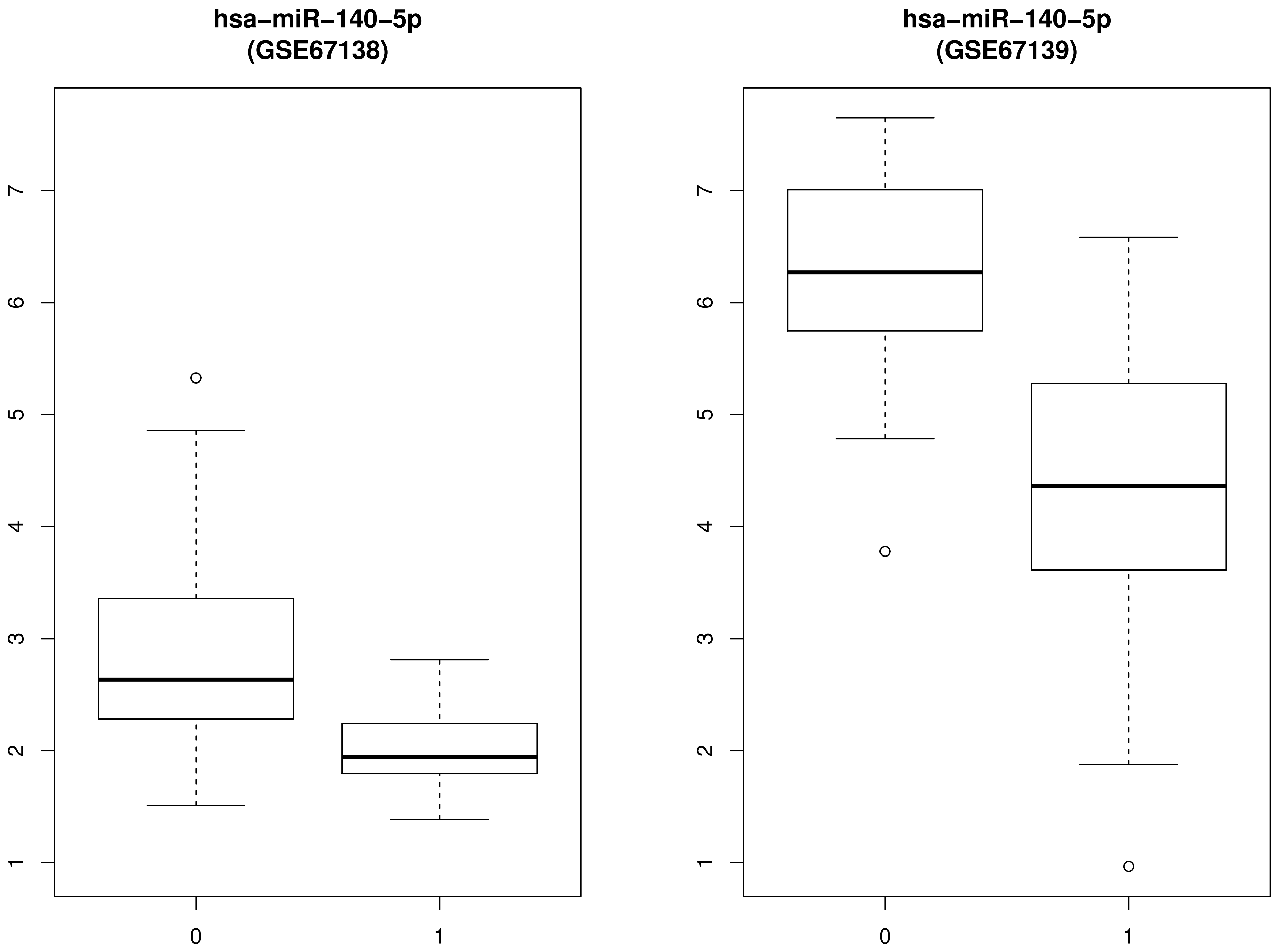
hsa−miR−140−5p
(GSE67138)
hsa−miR−140−5p
(GSE67139)
1
2
3
4
5
6
7
0
1

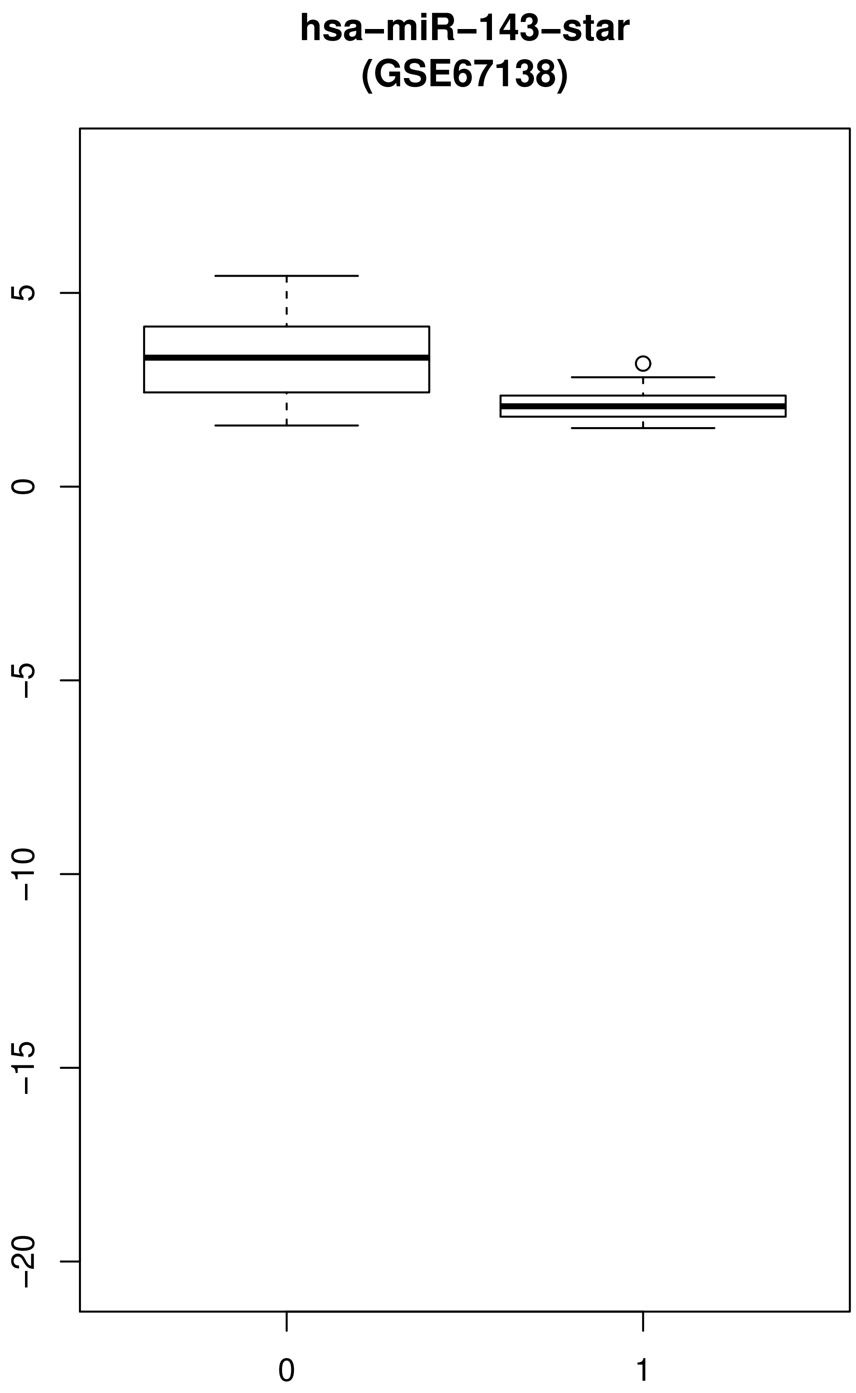
hsa−miR−143−star
(GSE67138)
5
0
−5
−10
−15
−20
0
1
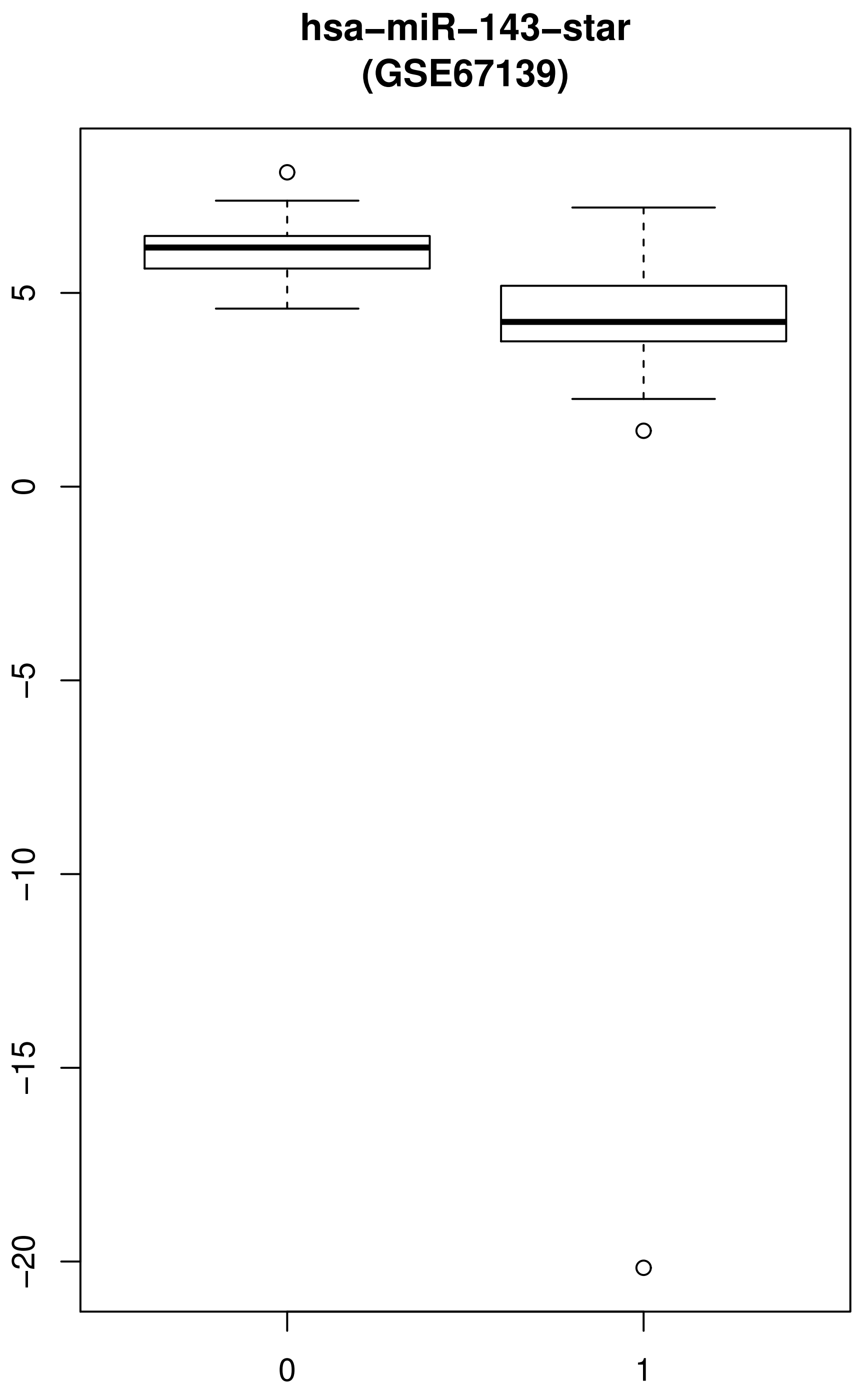
hsa−miR−143−star
(GSE67139)
5
0
−5
−10
−15
−20
0
1

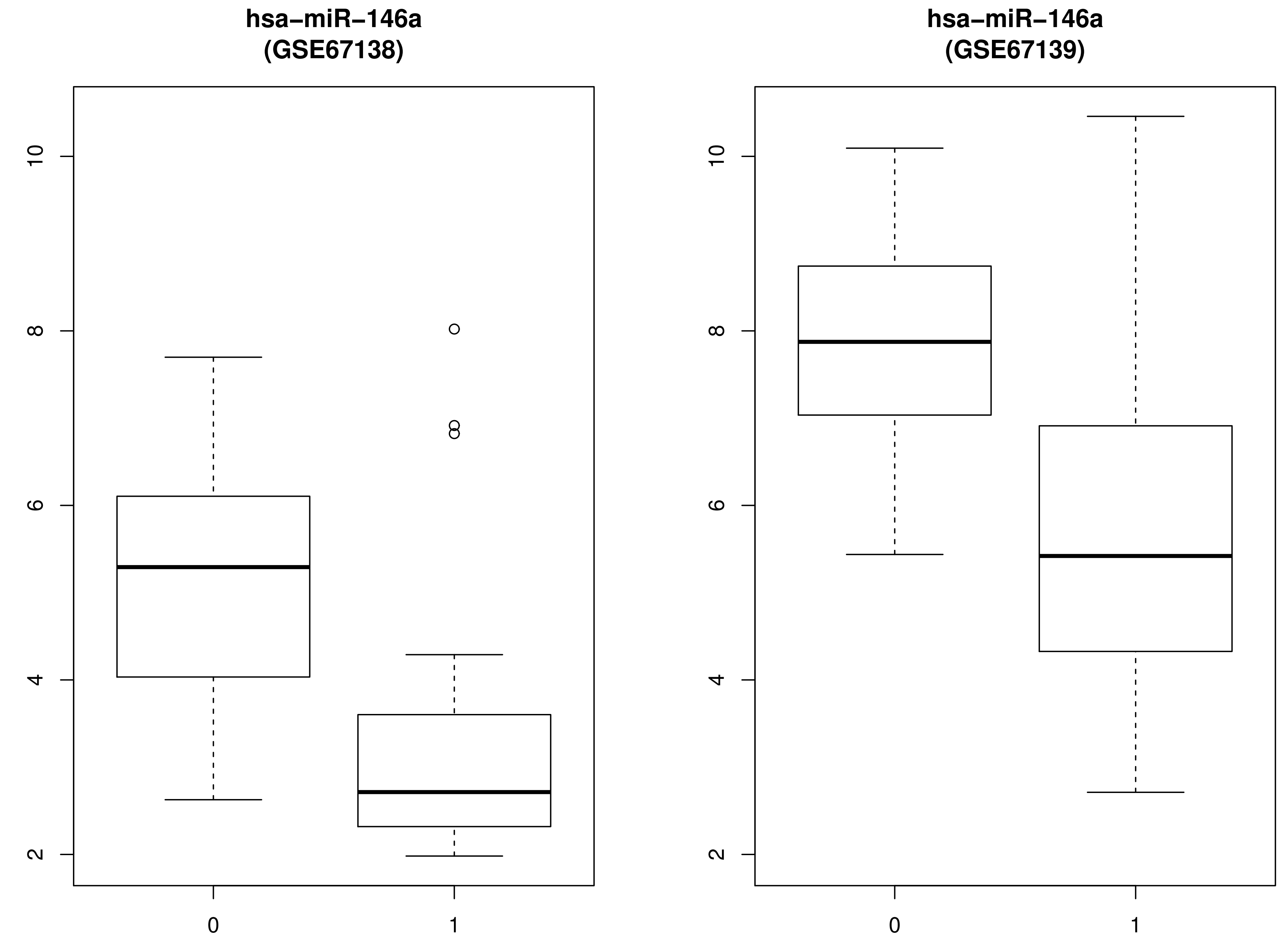
hsa−miR−146a
(GSE67138)
hsa−miR−146a
(GSE67139)
2
4
6
8
10
0
1

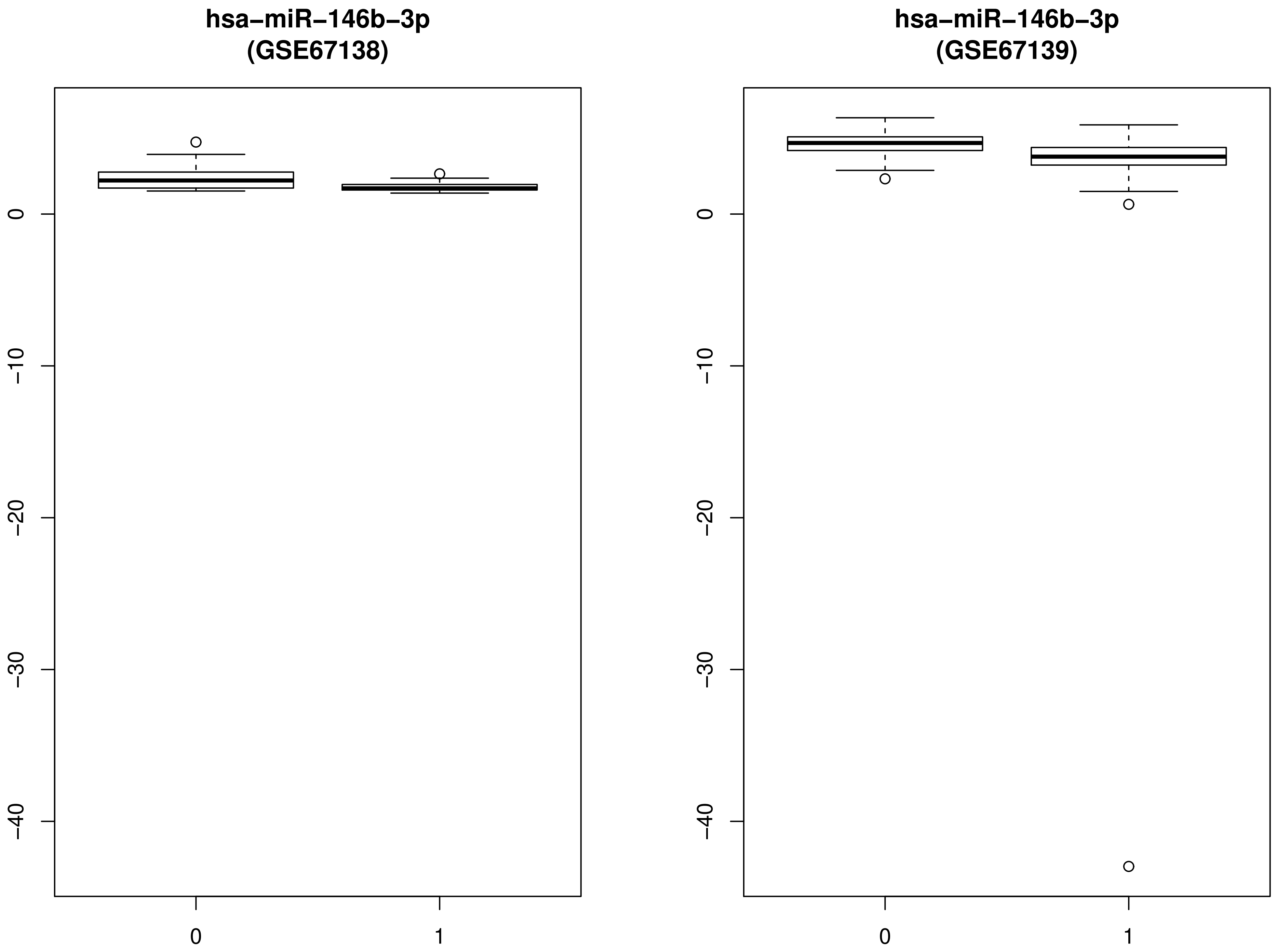
hsa−miR−146b−3p
(GSE67138)
0
−10
−20
−30
−40
0
1
hsa−miR−146b−3p
(GSE67139)
0
−10
−20
−30
−40
0
1

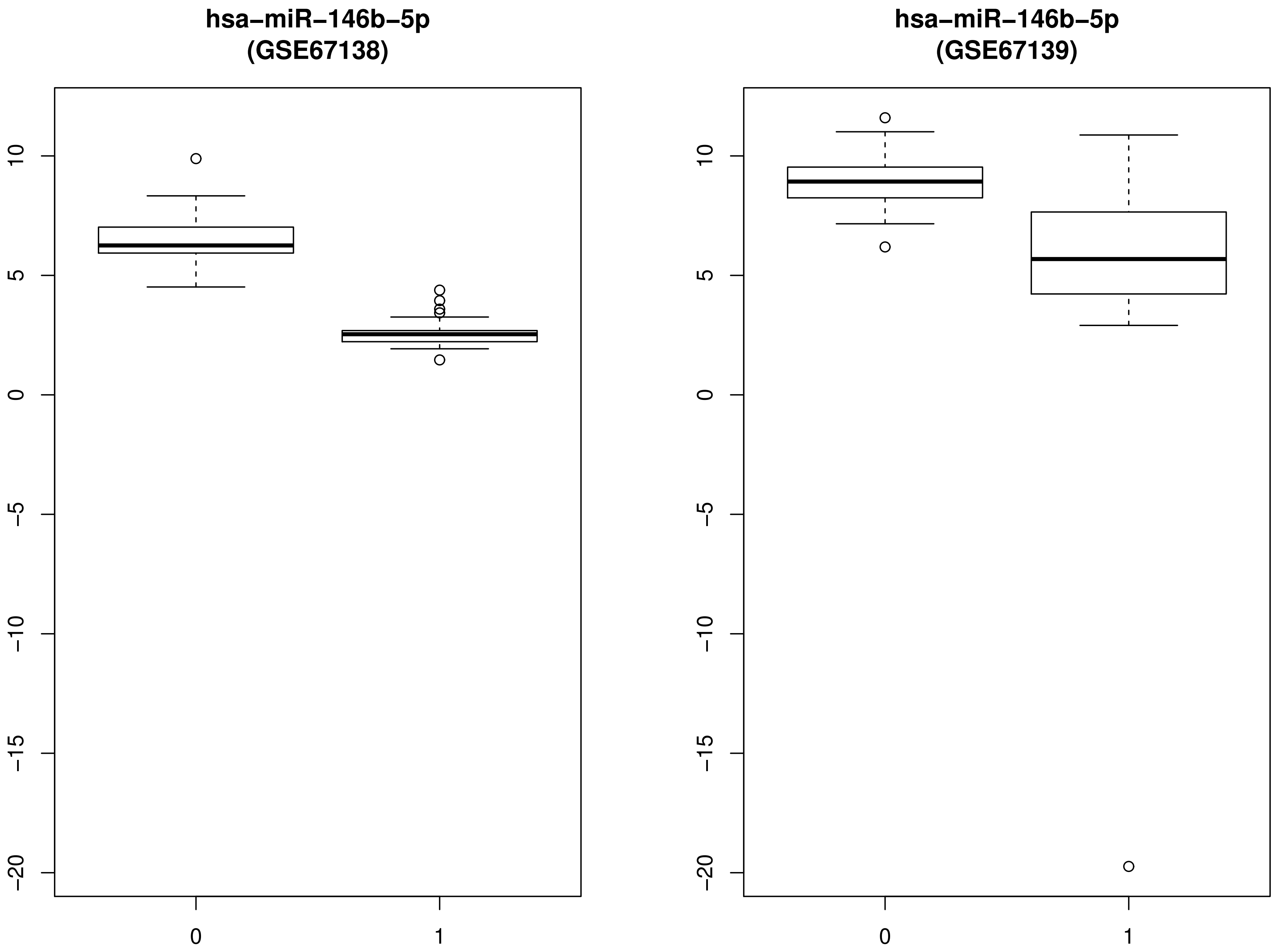

hsa−miR−146b−5p
(GSE67138)
hsa−miR−146b−5p
(GSE67139)
10
5
0
−5
−10
−15
−20
0
1

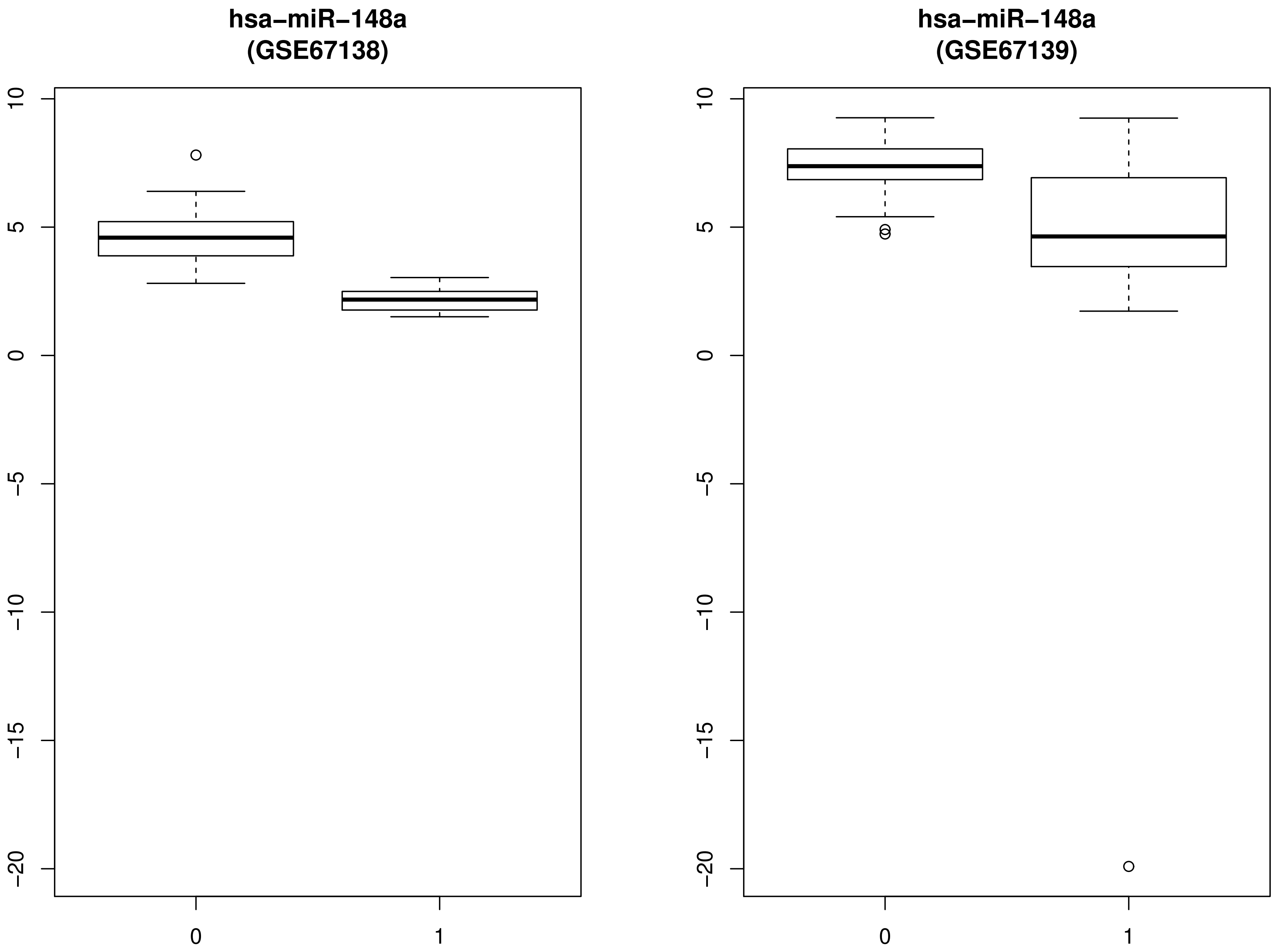
hsa−miR−148a
(GSE67138)
hsa−miR−148a
(GSE67139)
10
5
0
−5
−10
−15
−20
0
1

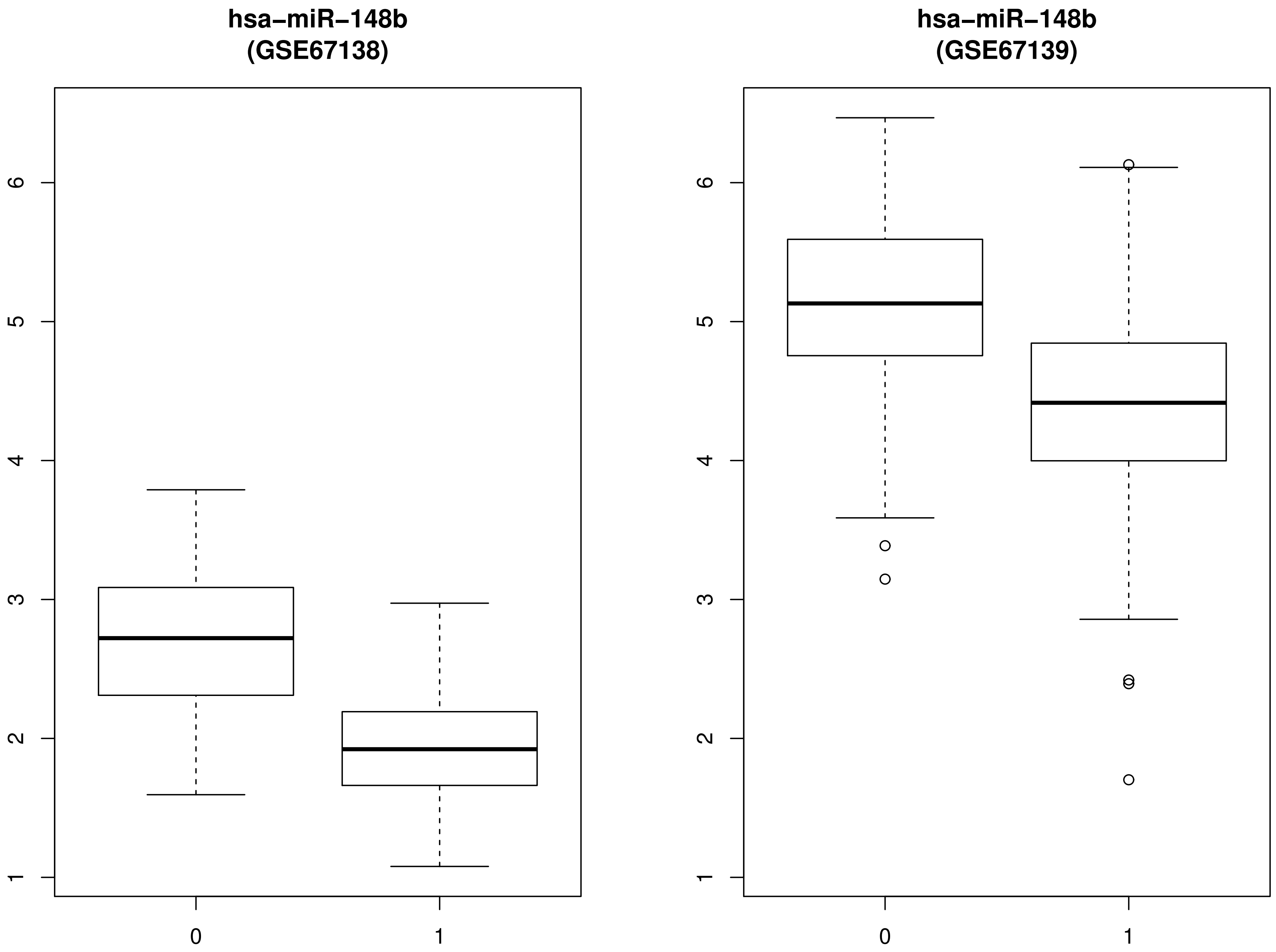
hsa−miR−148b
(GSE67138)
hsa−miR−148b
(GSE67139)
1
2
3
4
5
6
0
1

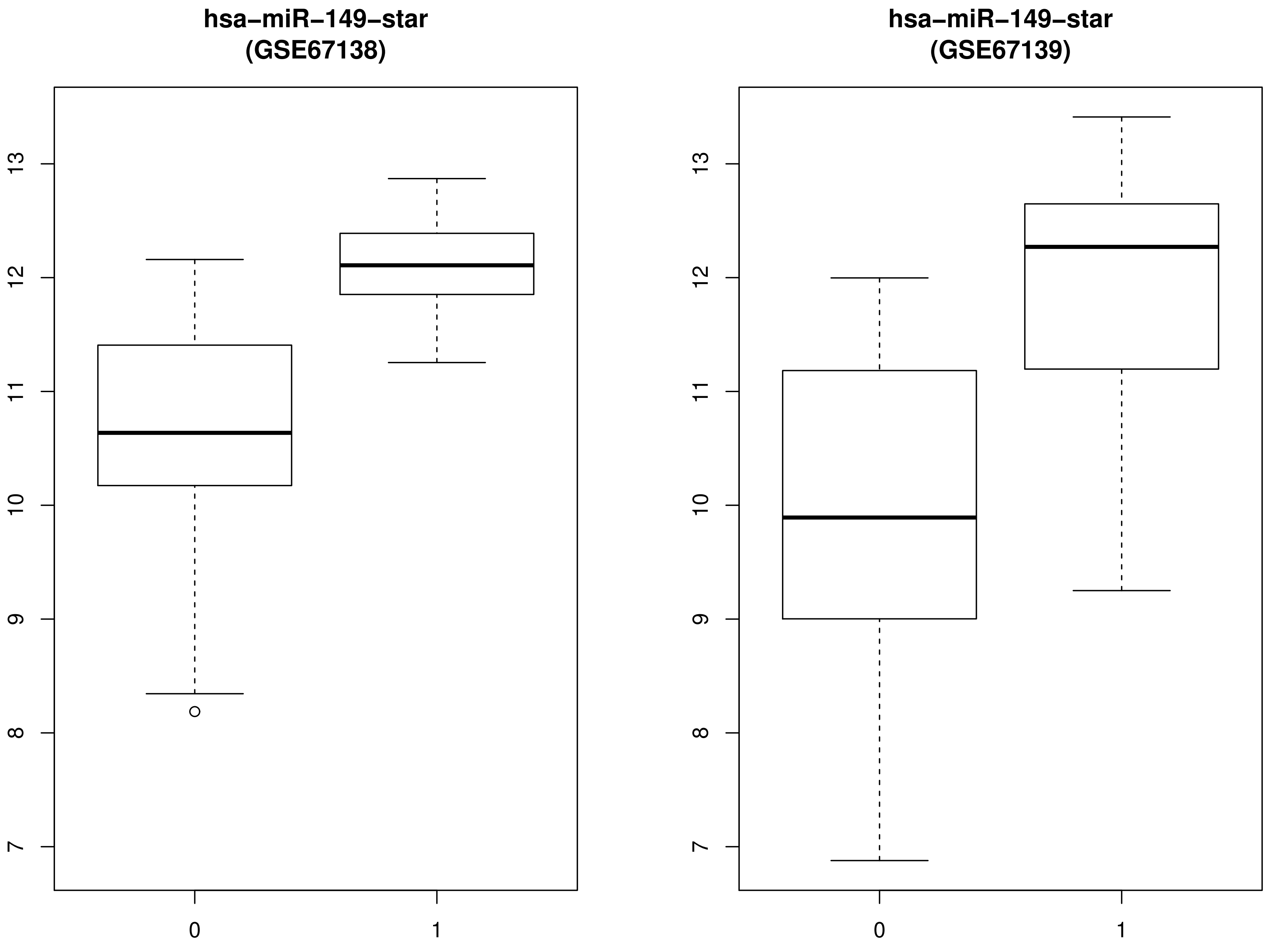

hsa−miR−149−star
(GSE67138)
hsa−miR−149−star
(GSE67139)
7
8
9
10
11
12
13
0
1

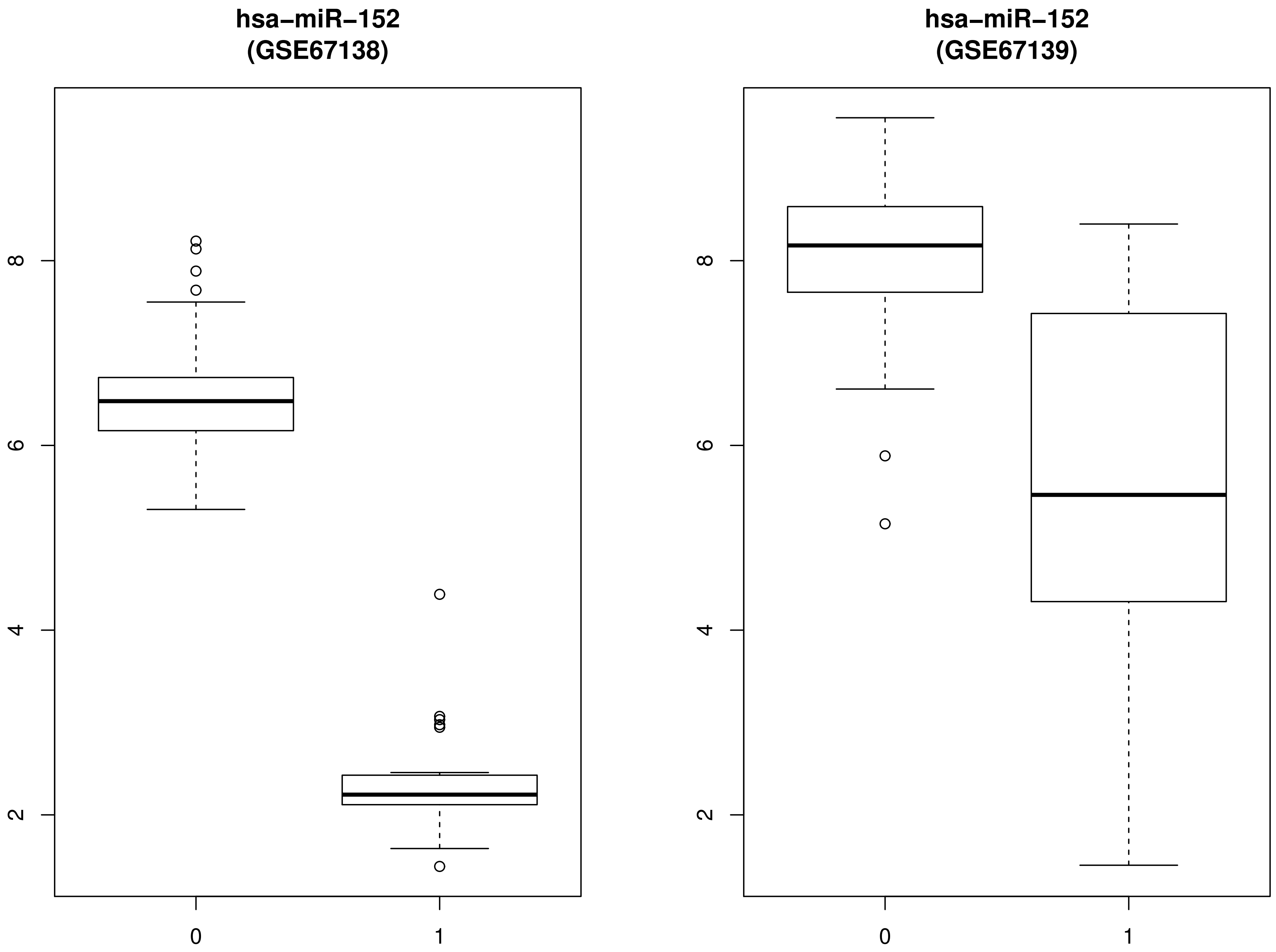

hsa−miR−152
(GSE67138)
8
6
4
2
0
1
hsa−miR−152
(GSE67139)
8
6
4
2
0
1

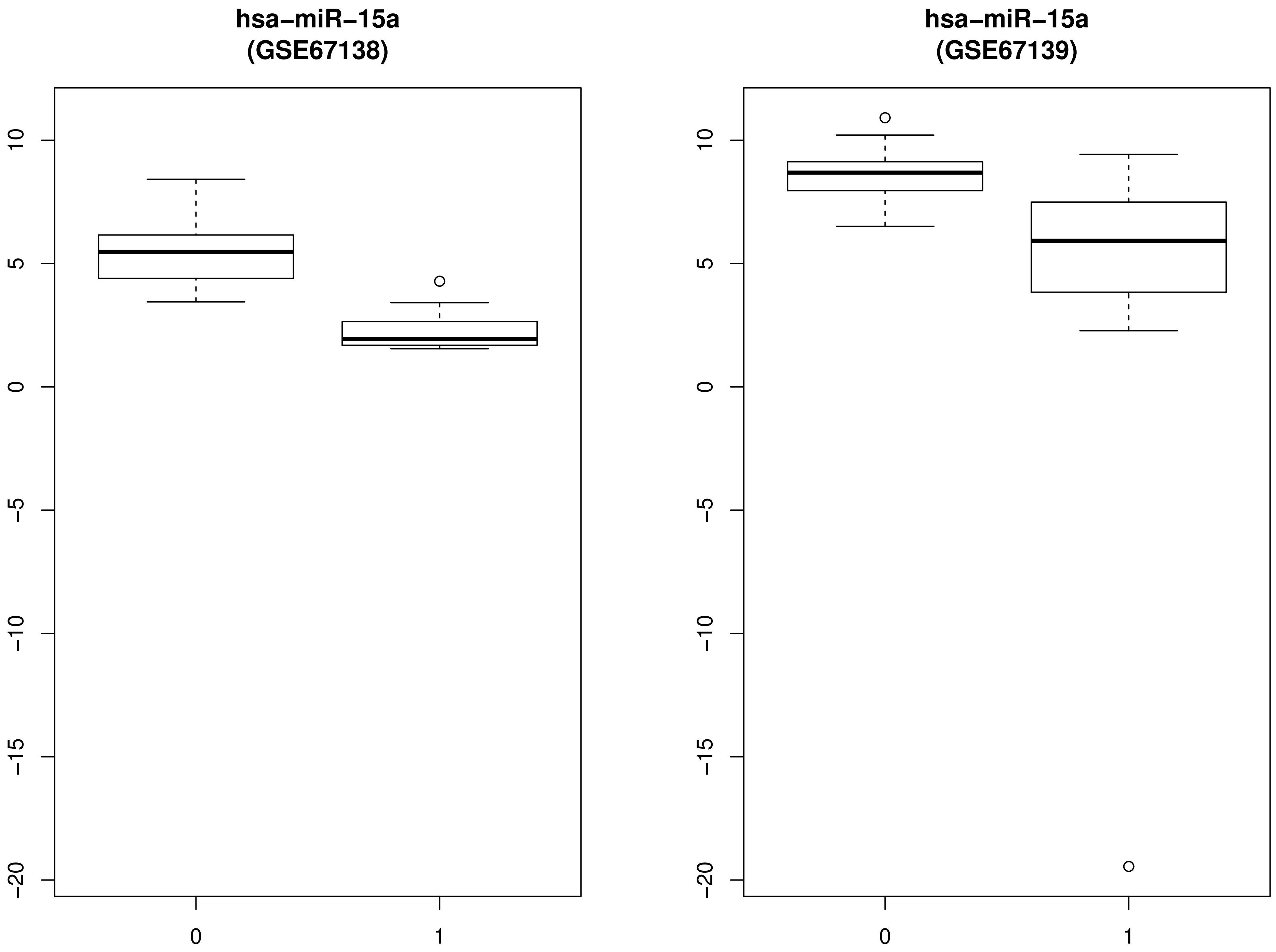
hsa−miR−15a
(GSE67138)
hsa−miR−15a
(GSE67139)
10
5
0
−5
−10
−15
−20
0
1

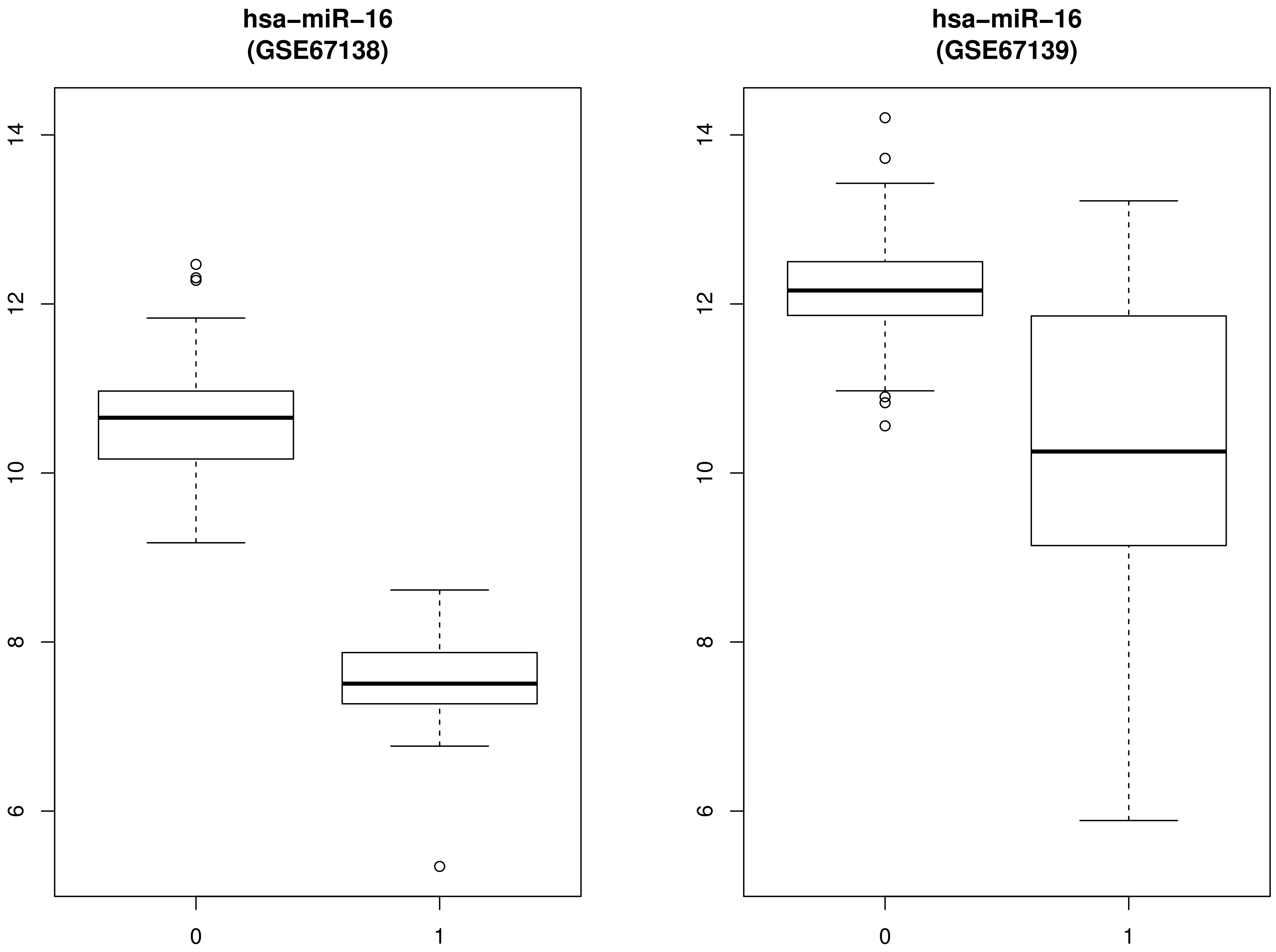
hsa−miR−16
(GSE67138)
hsa−miR−16
(GSE67139)
14
12
10
8
6
0
1

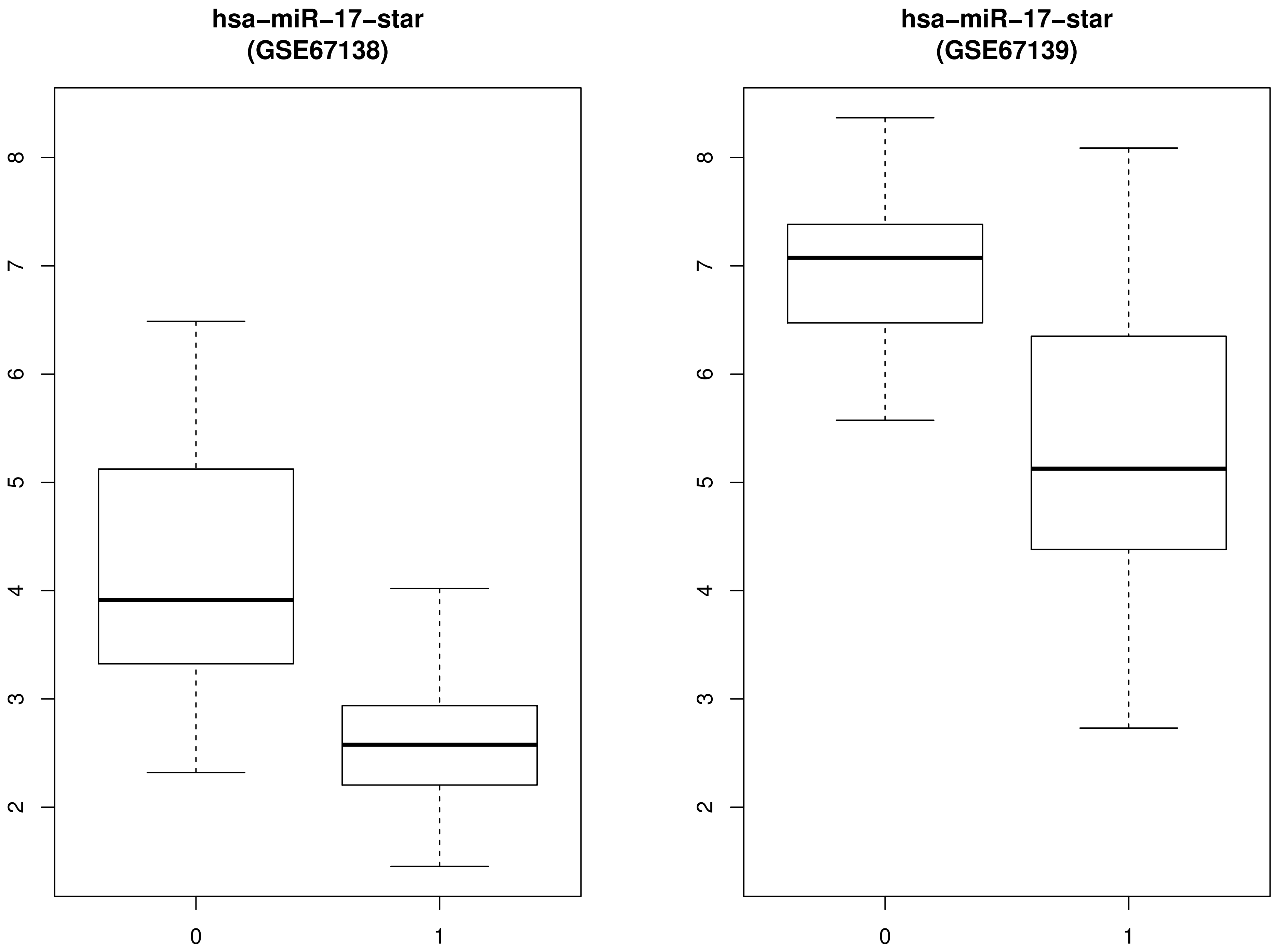

hsa−miR−17−star
(GSE67138)
hsa−miR−17−star
(GSE67139)
2
3
4
5
6
7
8
0
1

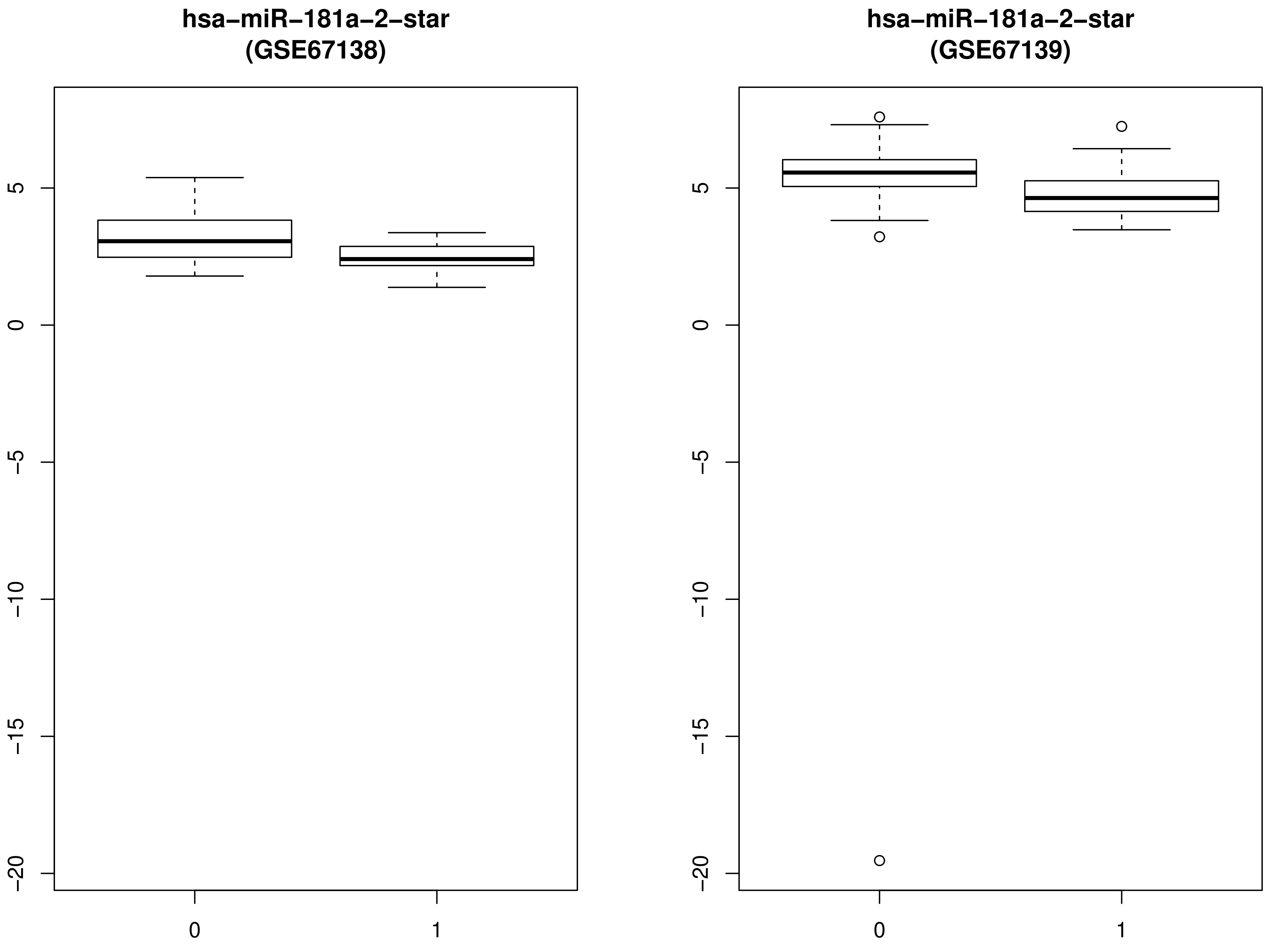

hsa−miR−181a−2−star
(GSE67138)
hsa−miR−181a−2−star
(GSE67139)
5
0
−5
−10
−15
−20
0
1

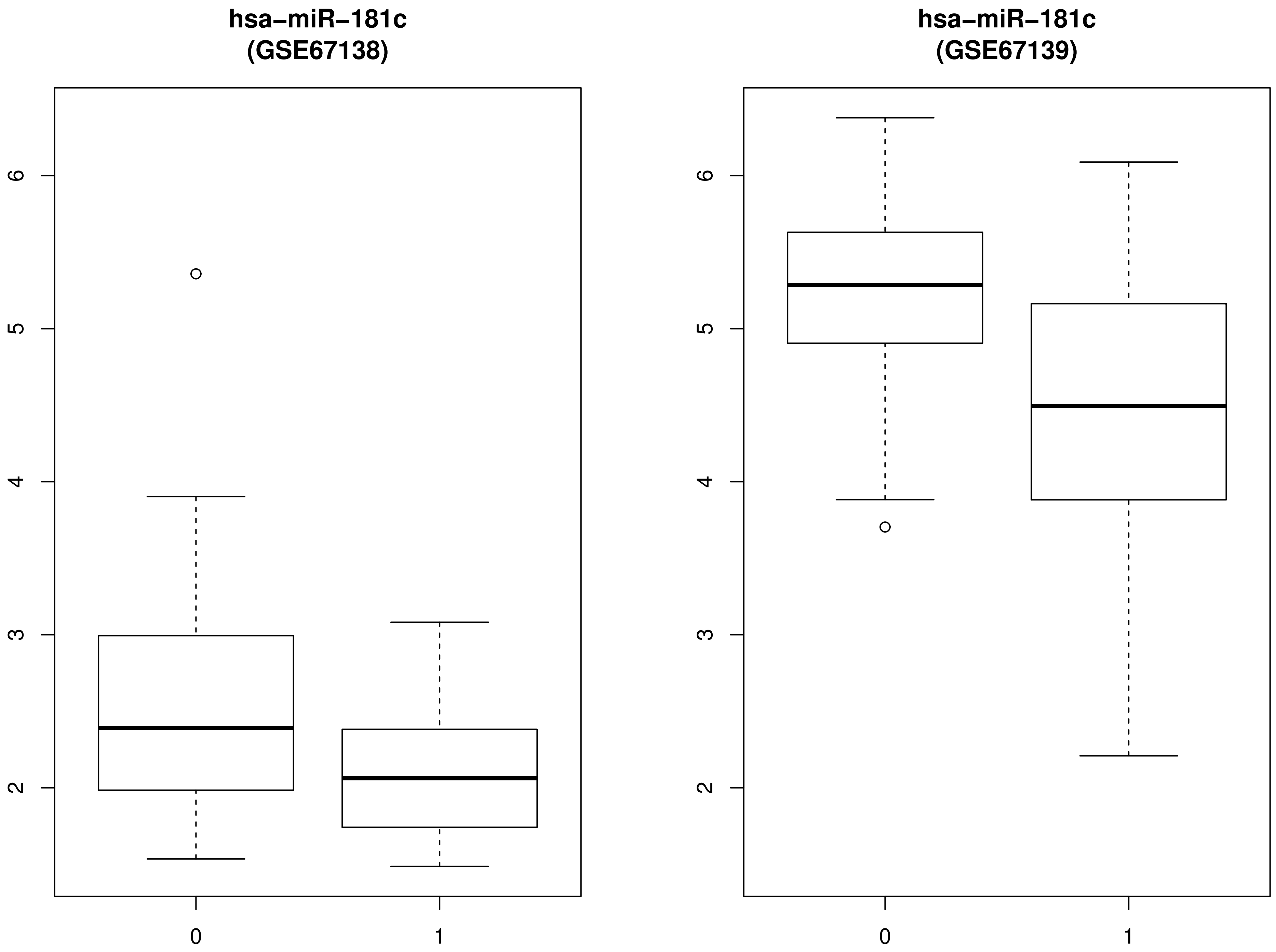

hsa−miR−181c
(GSE67138)
6
5
4
3
2
0
1
hsa−miR−181c
(GSE67139)
6
5
4
3
2
0
1

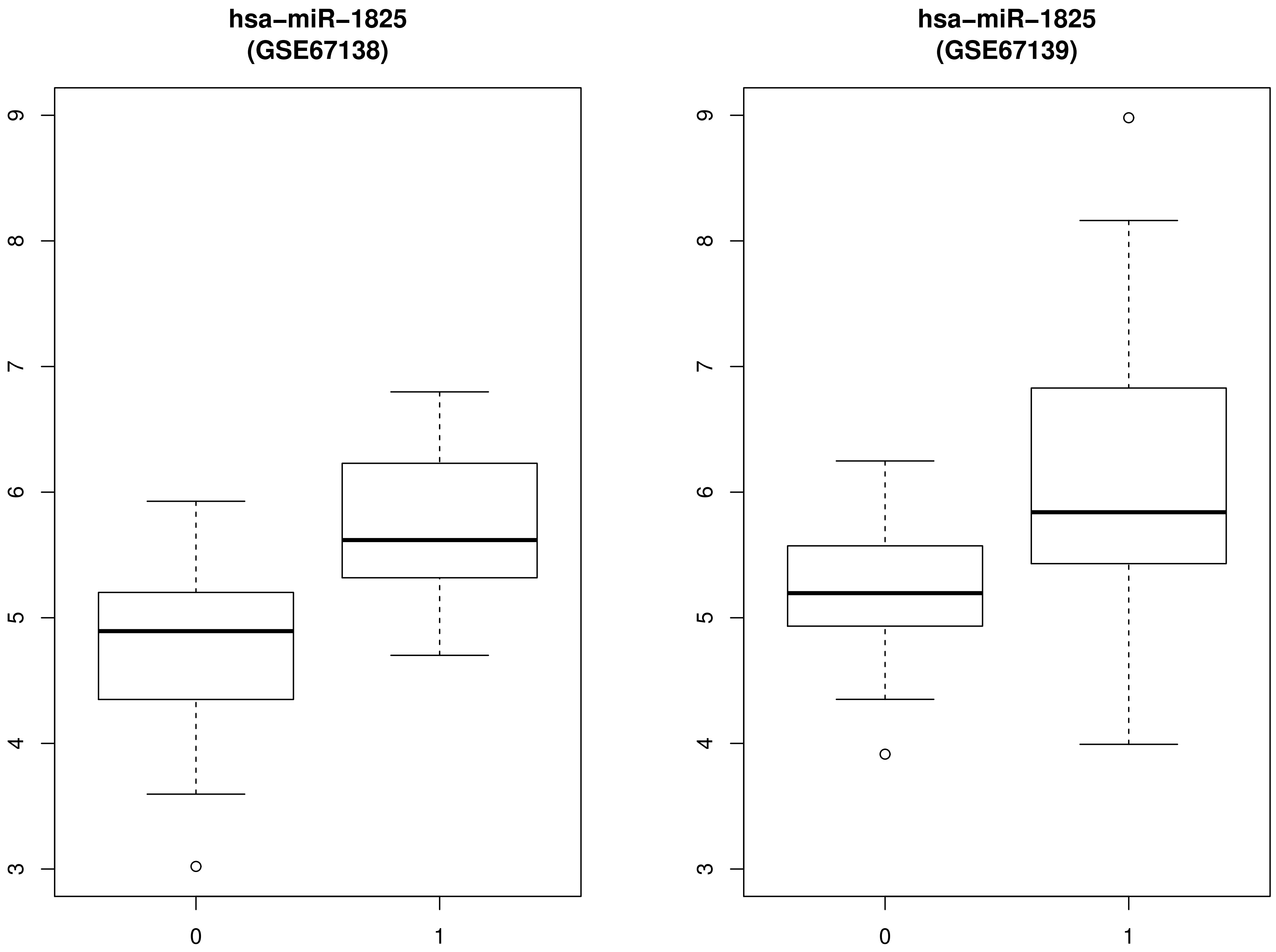

hsa−miR−1825
(GSE67138)
hsa−miR−1825
(GSE67139)
3
4
5
6
7
8
9
0
1

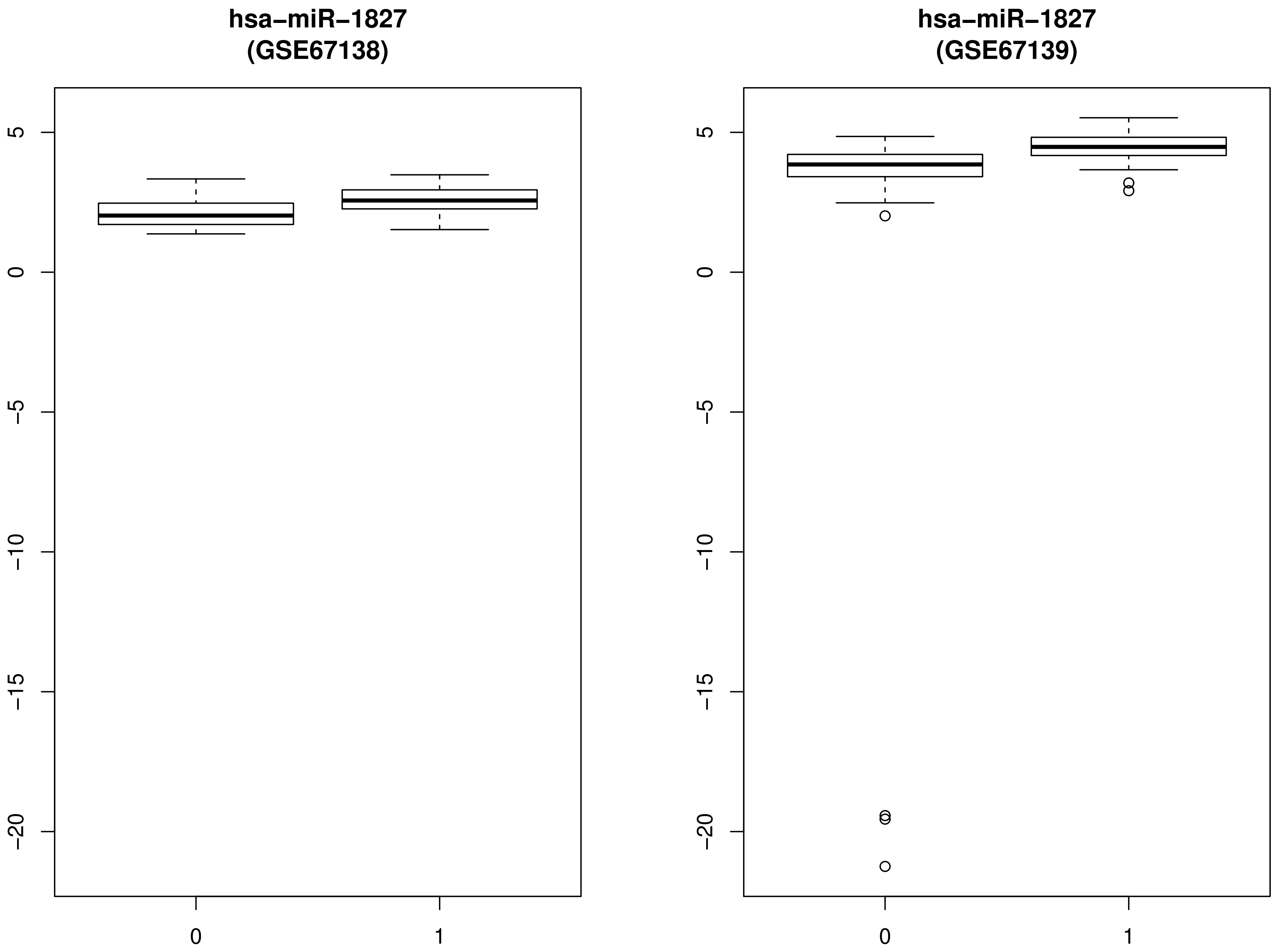
hsa−miR−1827
(GSE67138)
hsa−miR−1827
(GSE67139)
5
0
−5
−10
−15
−20
0
1

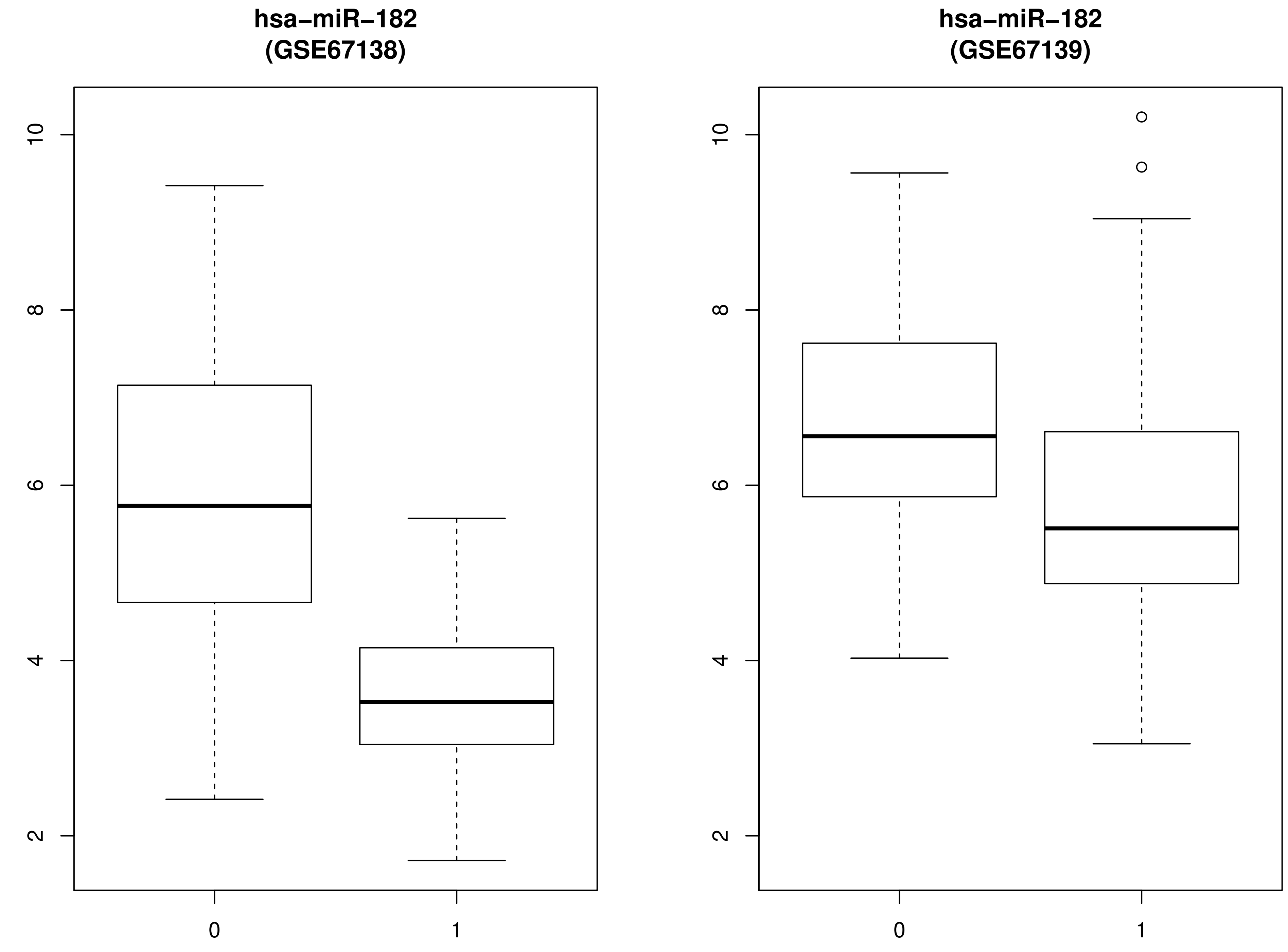

hsa−miR−182
(GSE67138)
hsa−miR−182
(GSE67139)
10
8
6
4
2
0
1

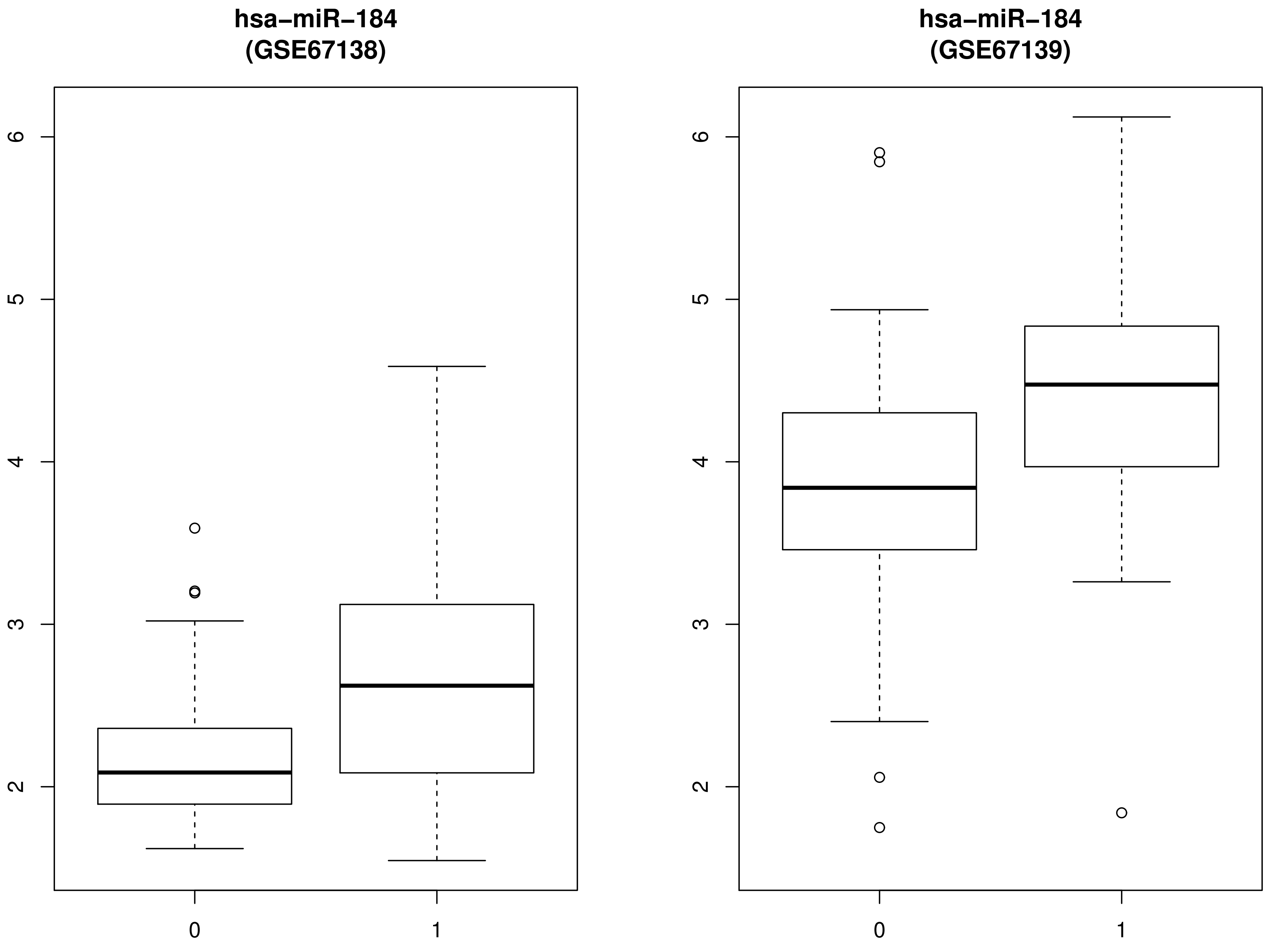
hsa−miR−184
(GSE67138)
hsa−miR−184
(GSE67139)
6
5
4
3
2
0
1

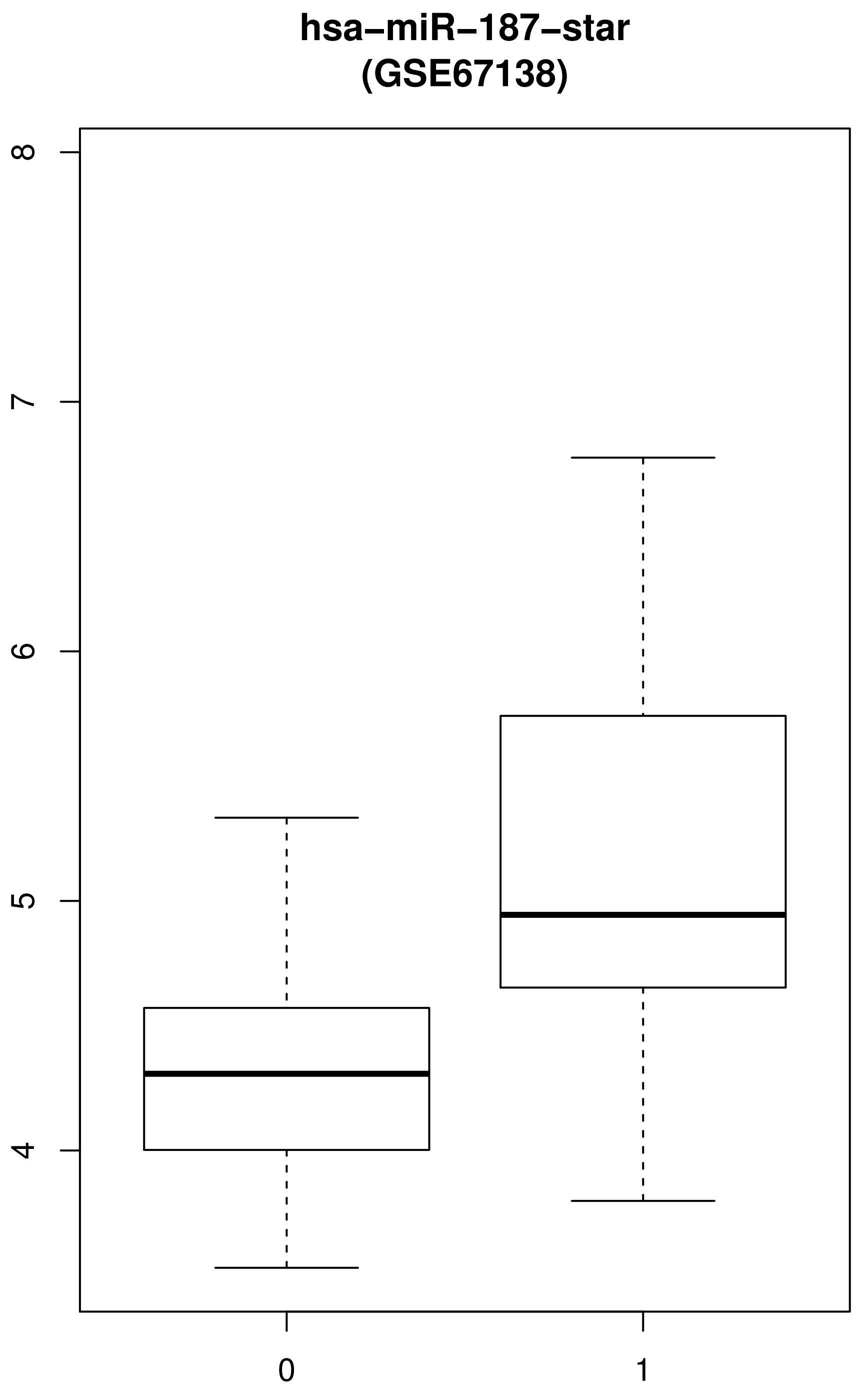
hsa−miR−187−star
(GSE67138)
8
7
6
5
4
0
1
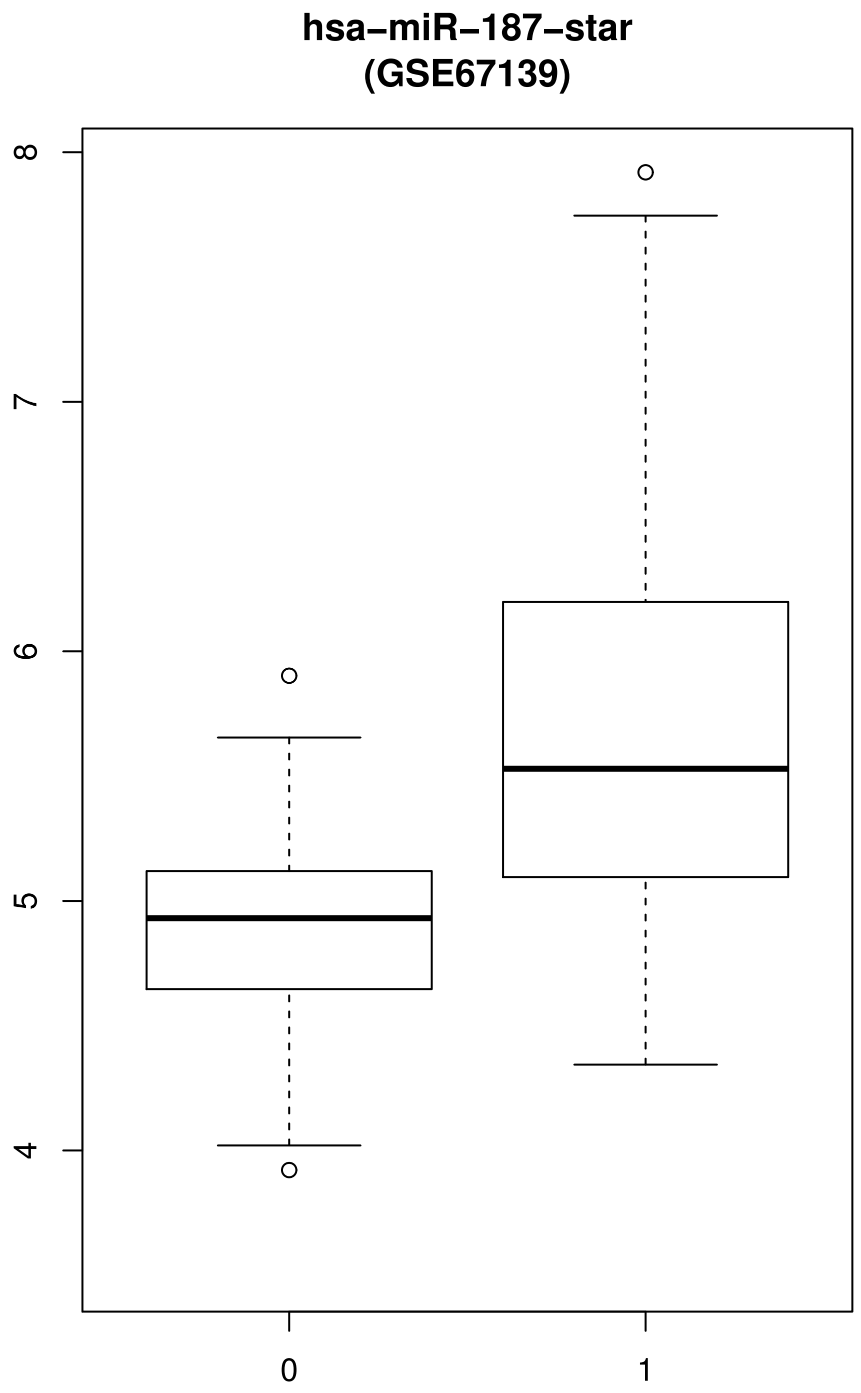
hsa−miR−187−star
(GSE67139)
8
7
6
5
4
0
1

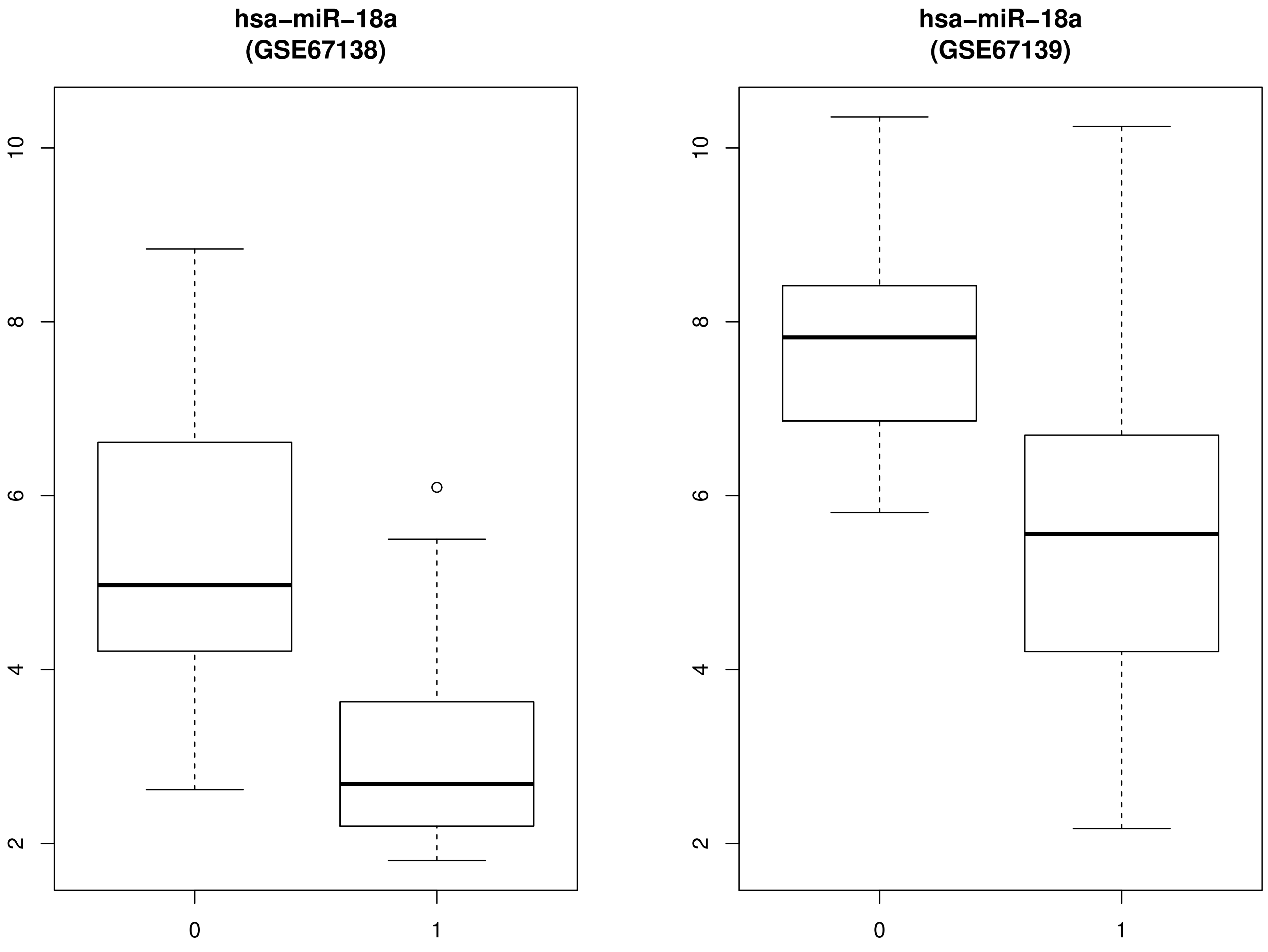
hsa−miR−18a
(GSE67138)
hsa−miR−18a
(GSE67139)
2
4
6
8
10
0
1

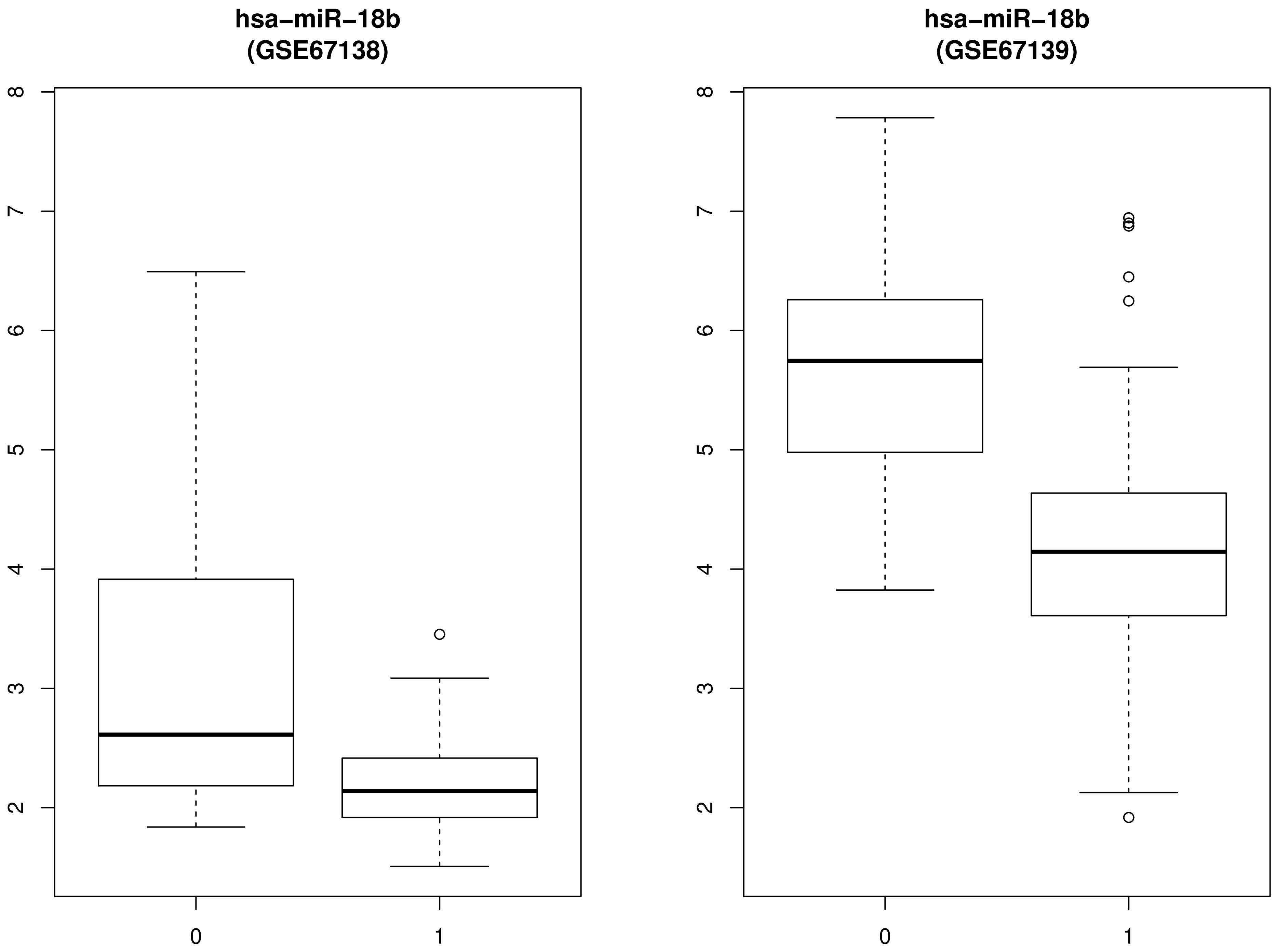
hsa−miR−18b
(GSE67138)
hsa−miR−18b
(GSE67139)
2
3
4
5
6
7
8
0
1

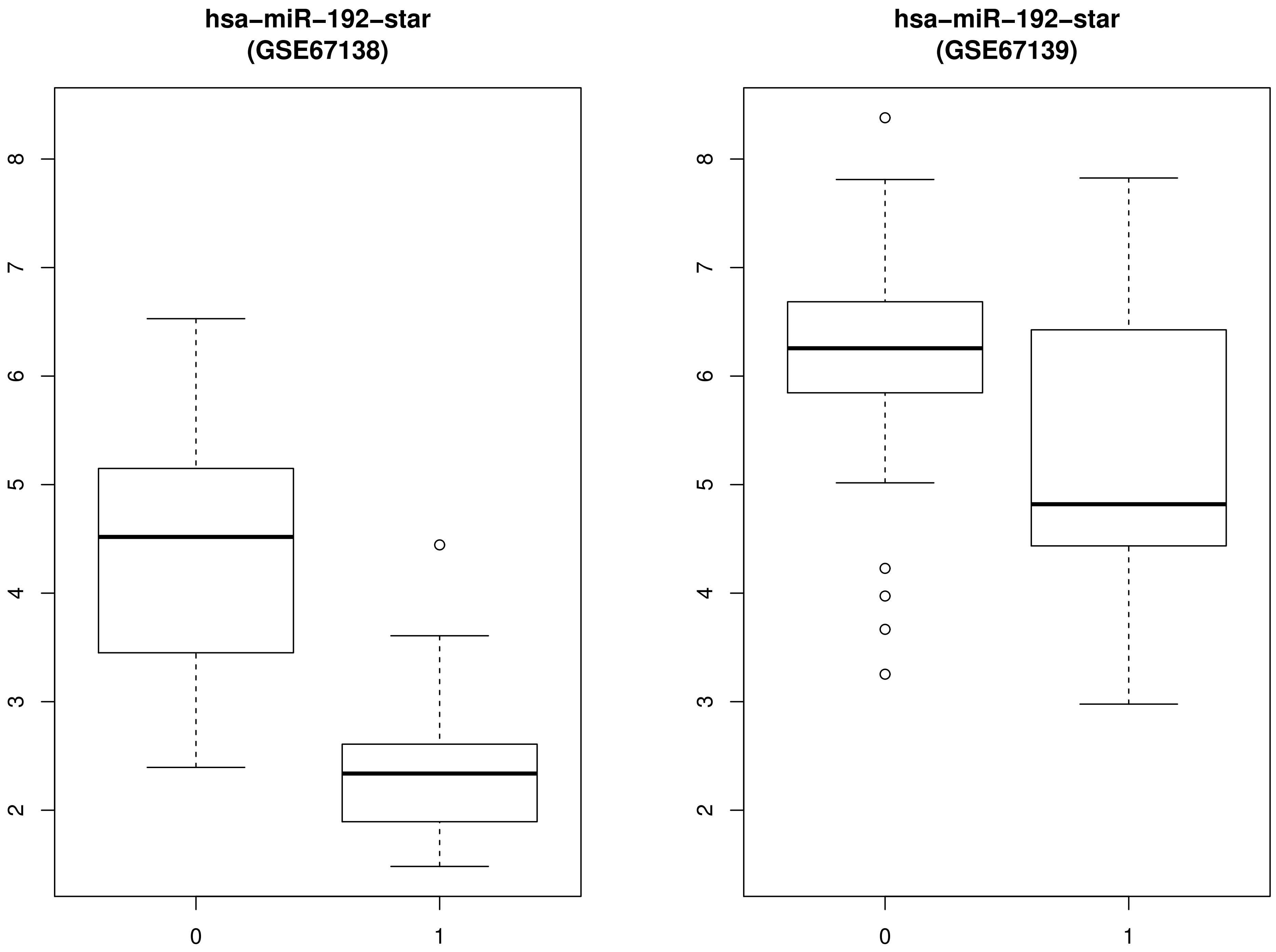
hsa−miR−192−star
(GSE67138)
hsa−miR−192−star
(GSE67139)
2
3
4
5
6
7
8
0
1

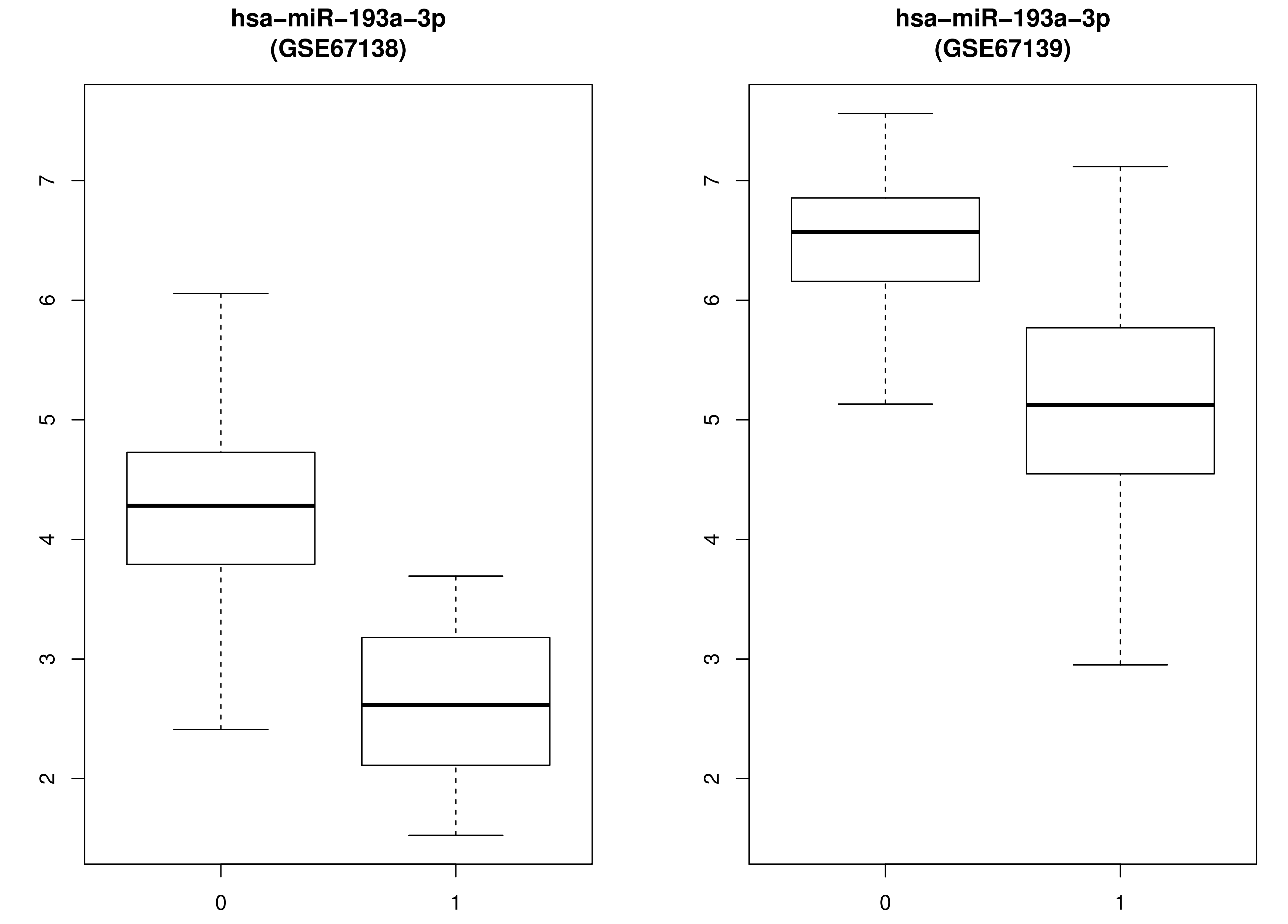
hsa–miR–193a–3p
(GSE67138)
hsa–miR–193a–3p
(GSE67139)
2
3
4
5
6
7
0
1

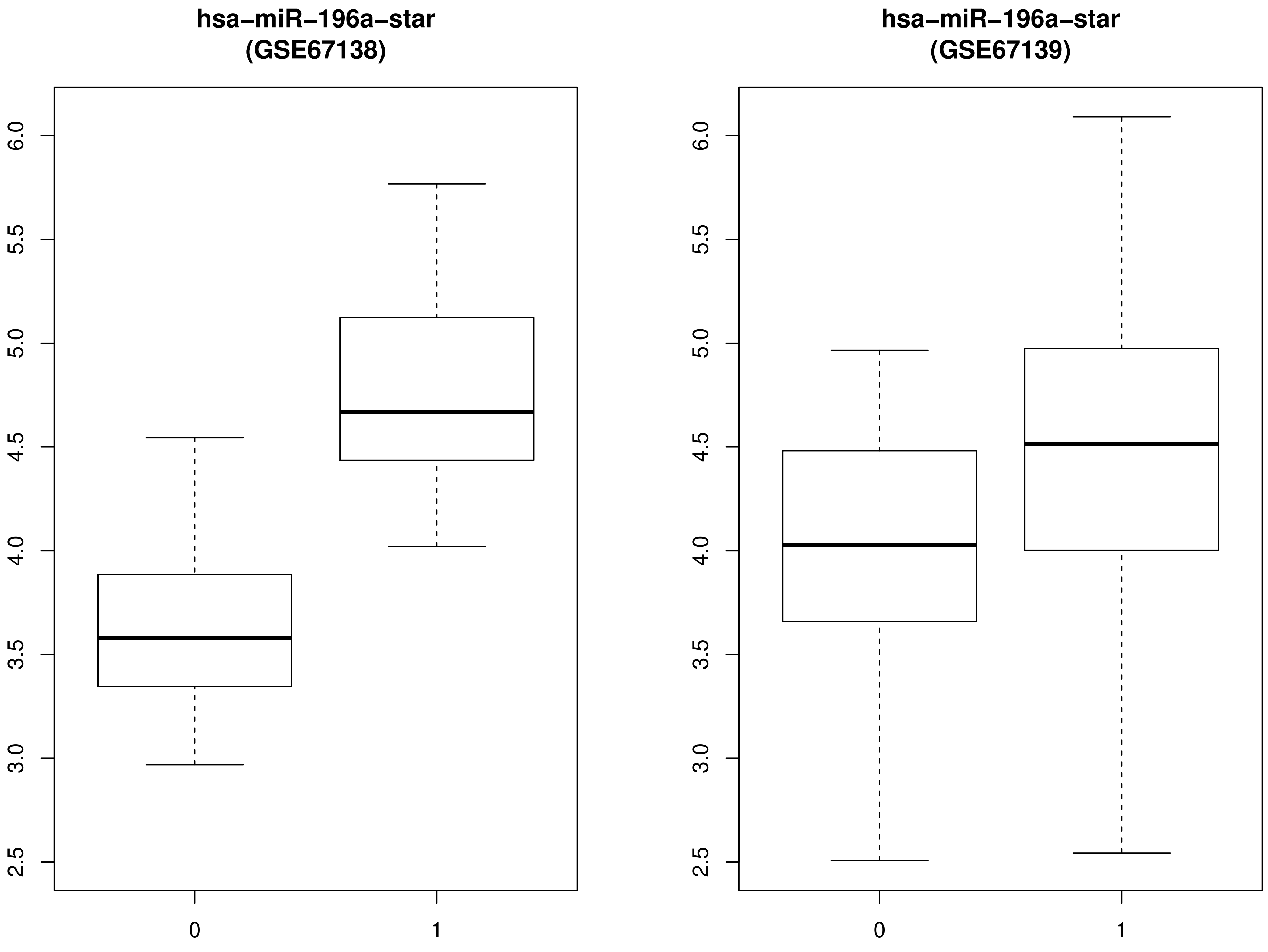
hsa−miR−196a−star
(GSE67138)
hsa−miR−196a−star
(GSE67139)
2.5
3.0
3.5
4.0
4.5
5.0
5.5
6.0
0
1

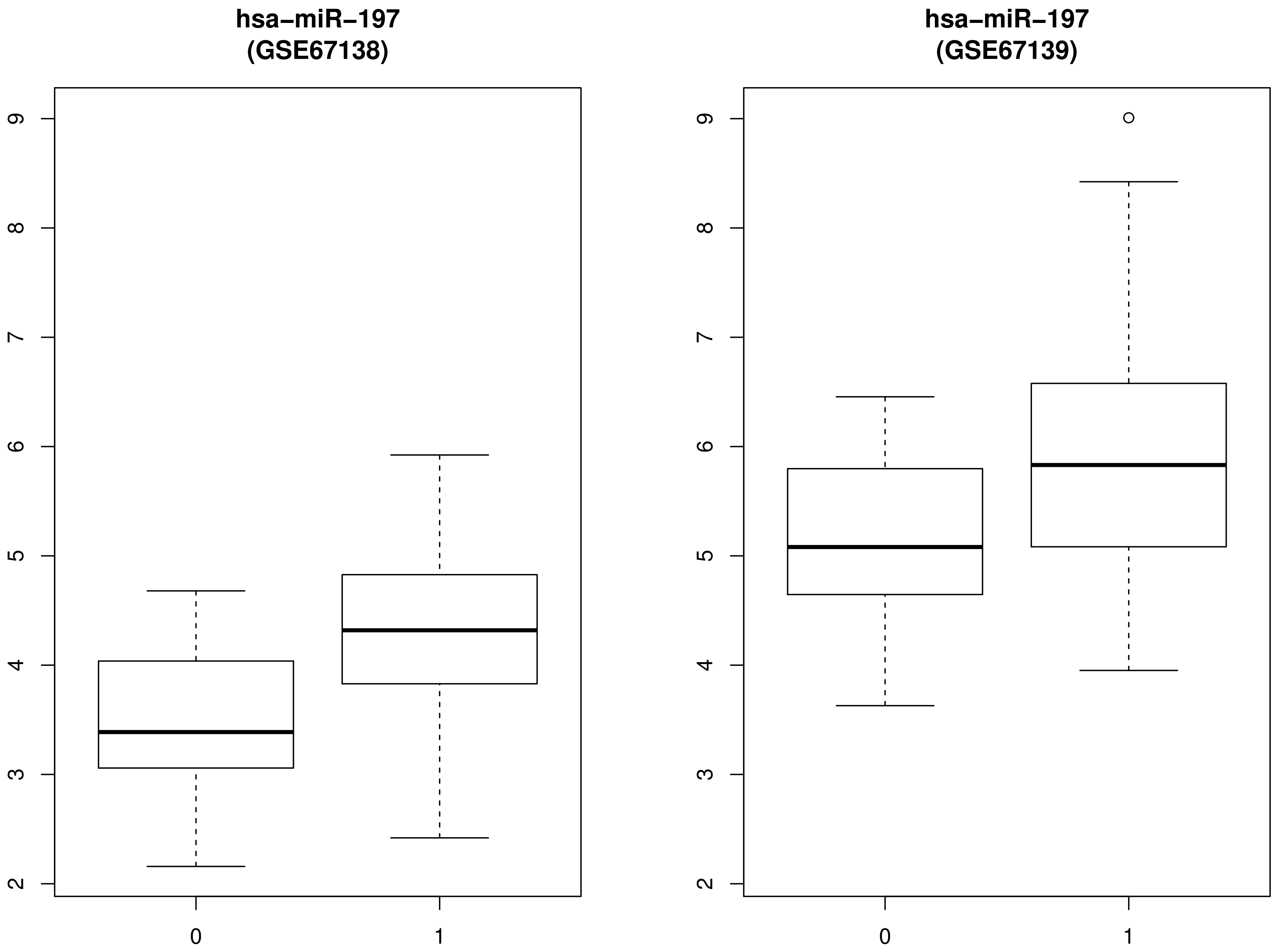
hsa−miR−197
(GSE67138)
hsa−miR−197
(GSE67139)
2
3
4
5
6
7
8
9
0
1

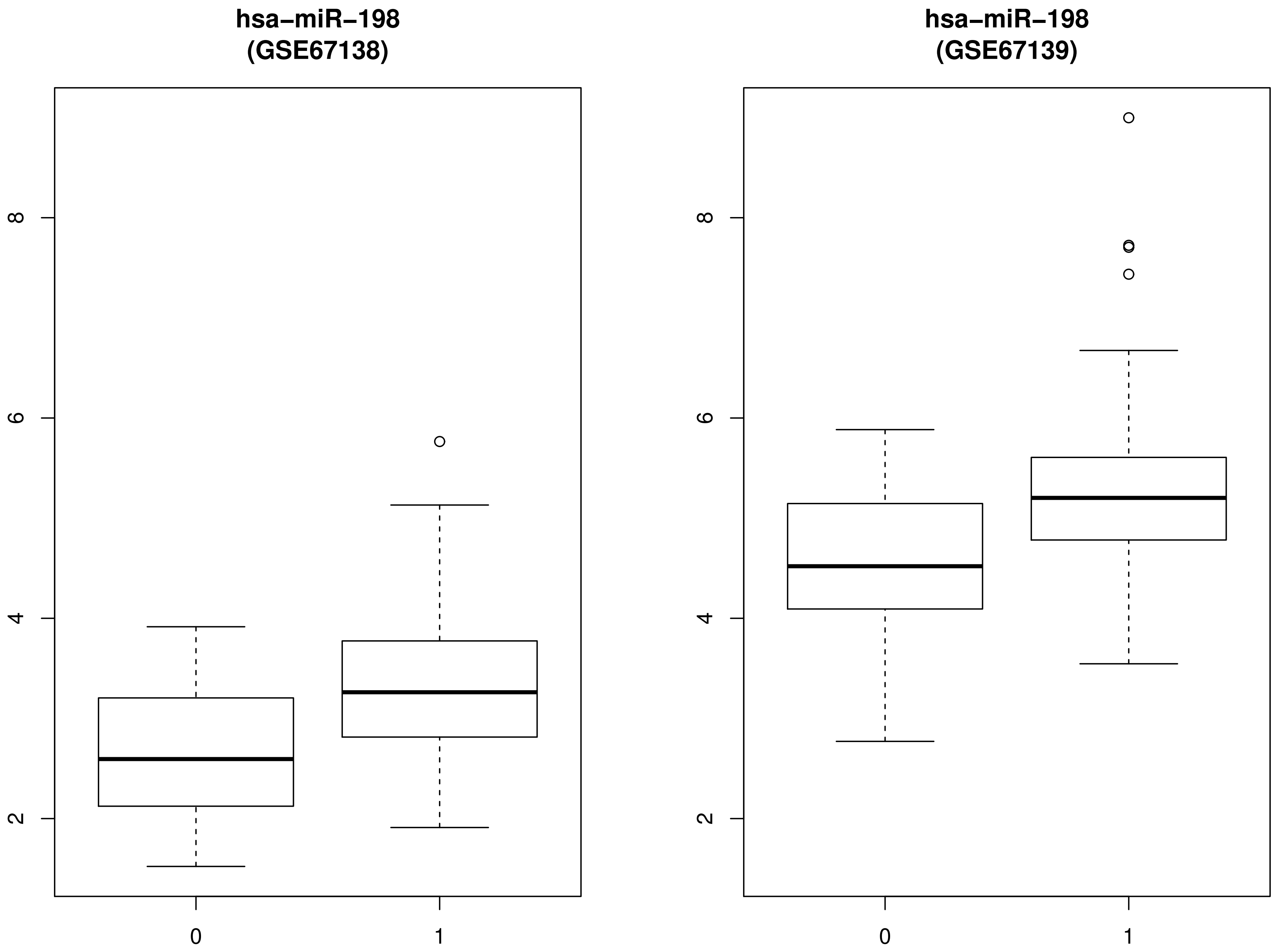
hsa−miR−198
(GSE67138)
hsa−miR−198
(GSE67139)
2
4
6
8
0
1

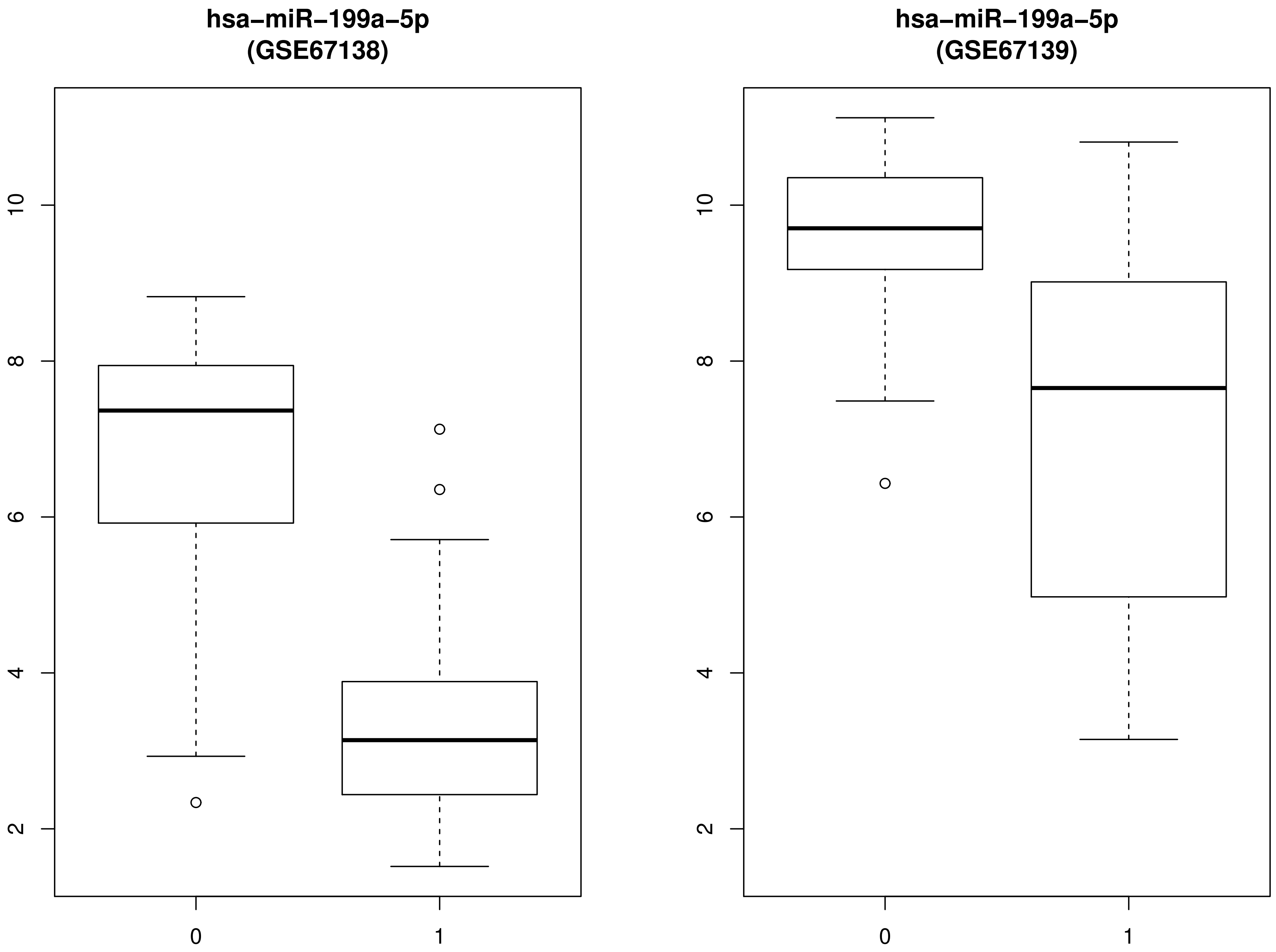

hsa−miR−199a−5p
(GSE67138)
hsa−miR−199a−5p
(GSE67139)
2
4
6
8
10
0
1

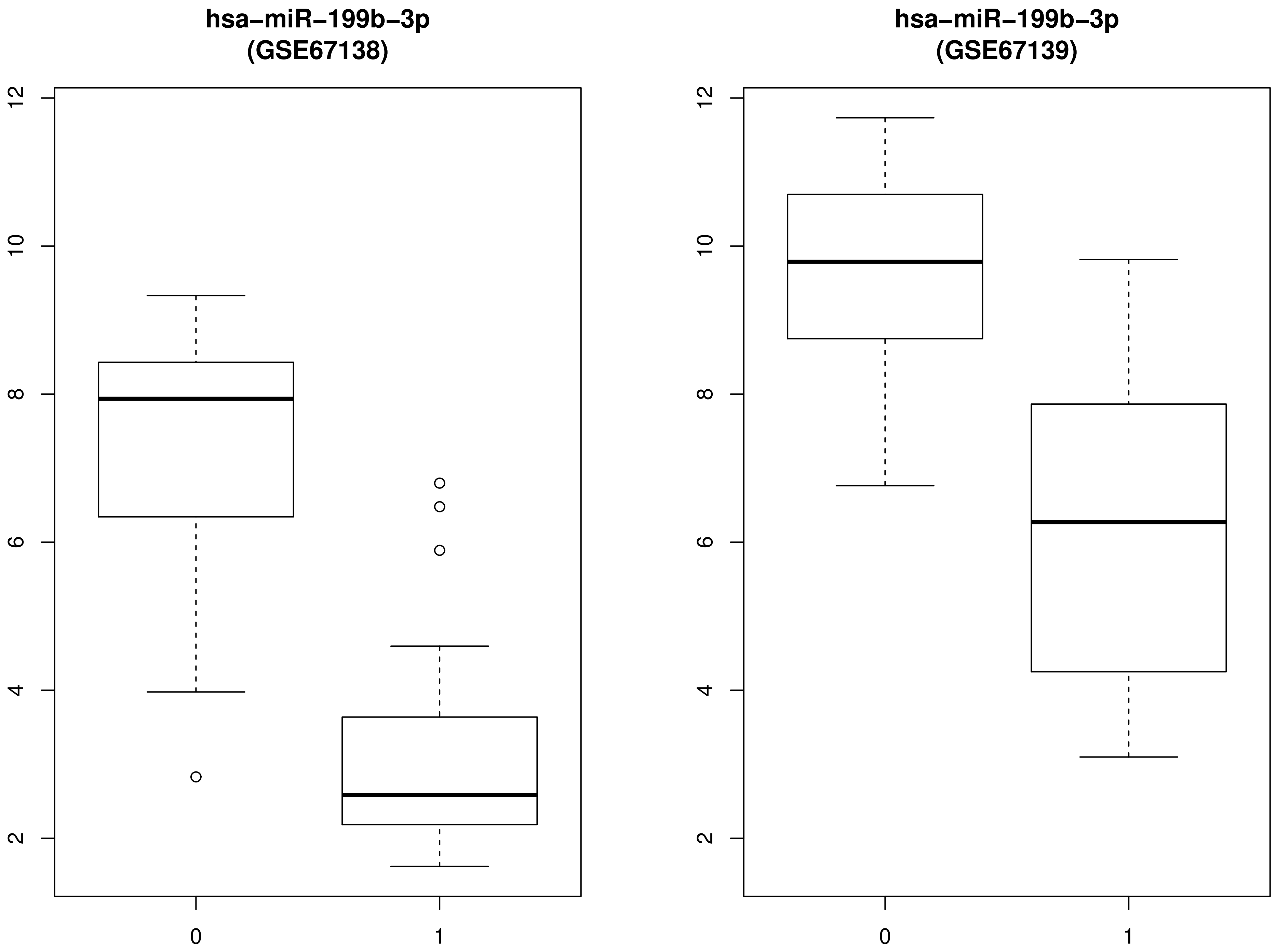

hsa−miR−199b−3p
(GSE67138)
hsa−miR−199b−3p
(GSE67139)
2
4
6
8
10
12
0
1

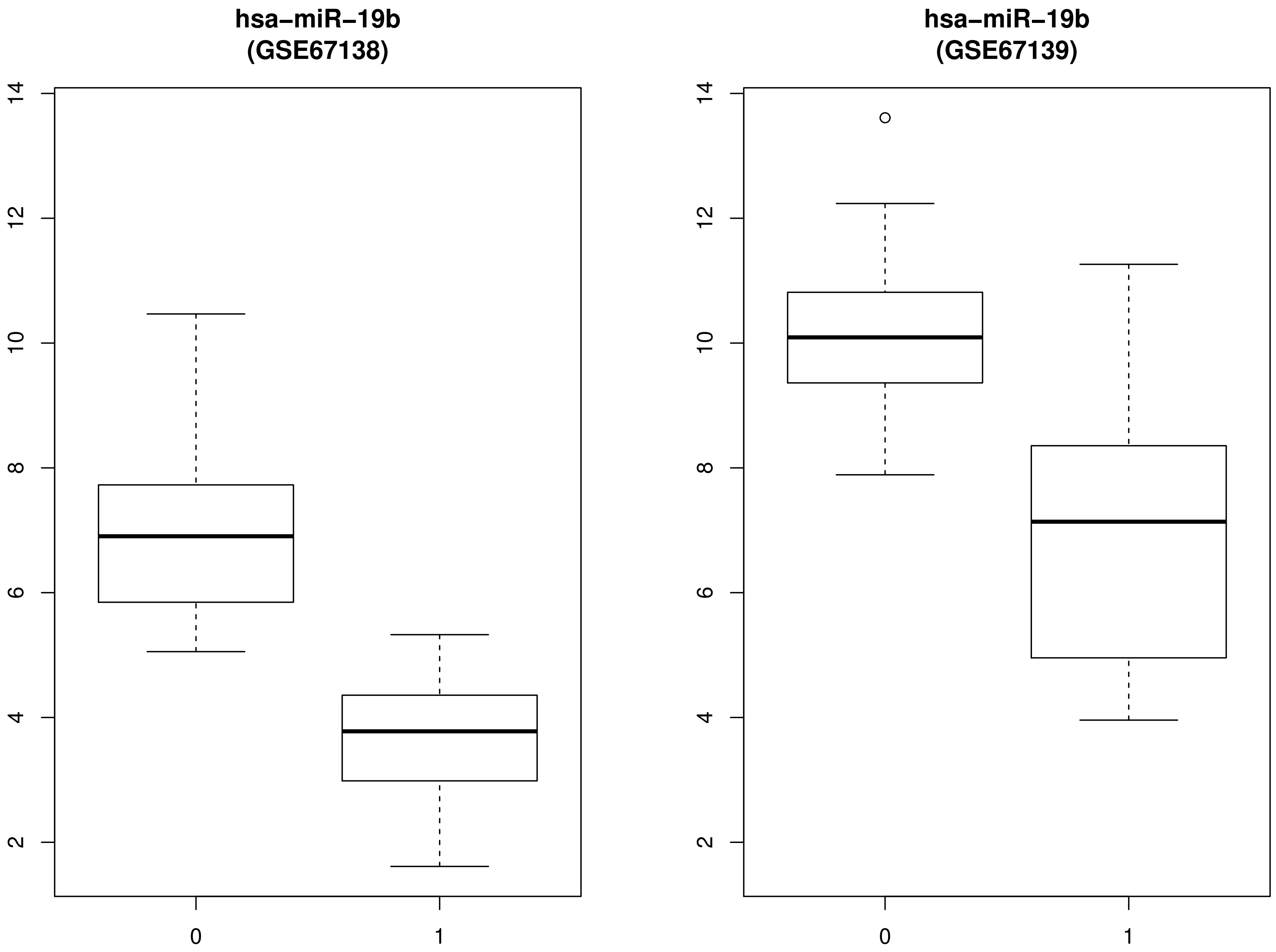

hsa−miR−19b
(GSE67138)
hsa−miR−19b
(GSE67139)
14
12
10
8
6
4
2
0
1

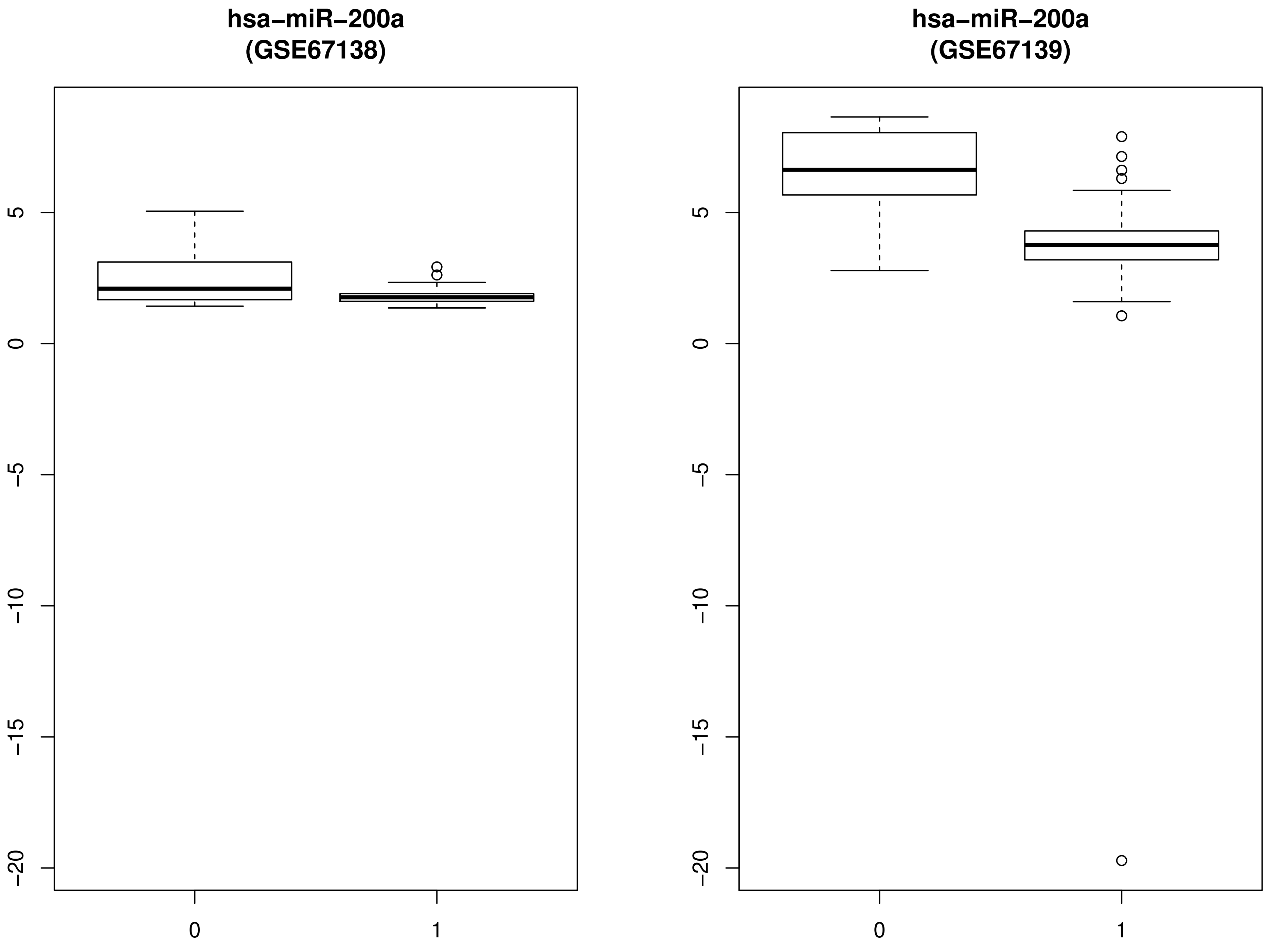
hsa−miR−200a
(GSE67138)
hsa−miR−200a
(GSE67139)
5
0
−5
−10
−15
−20
0
1

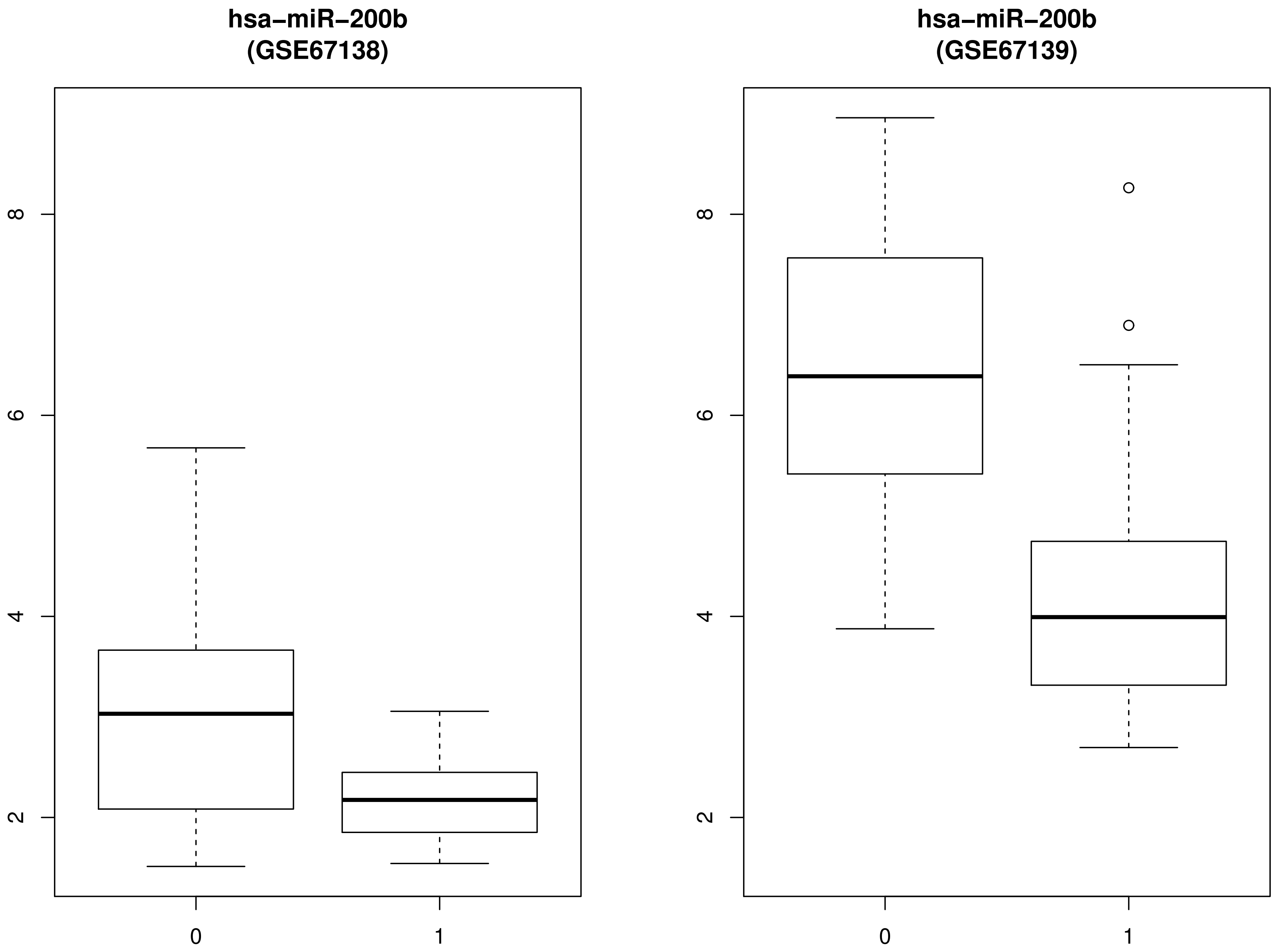
hsa−miR−200b
(GSE67138)
hsa−miR−200b
(GSE67139)
2
4
6
8
0
1

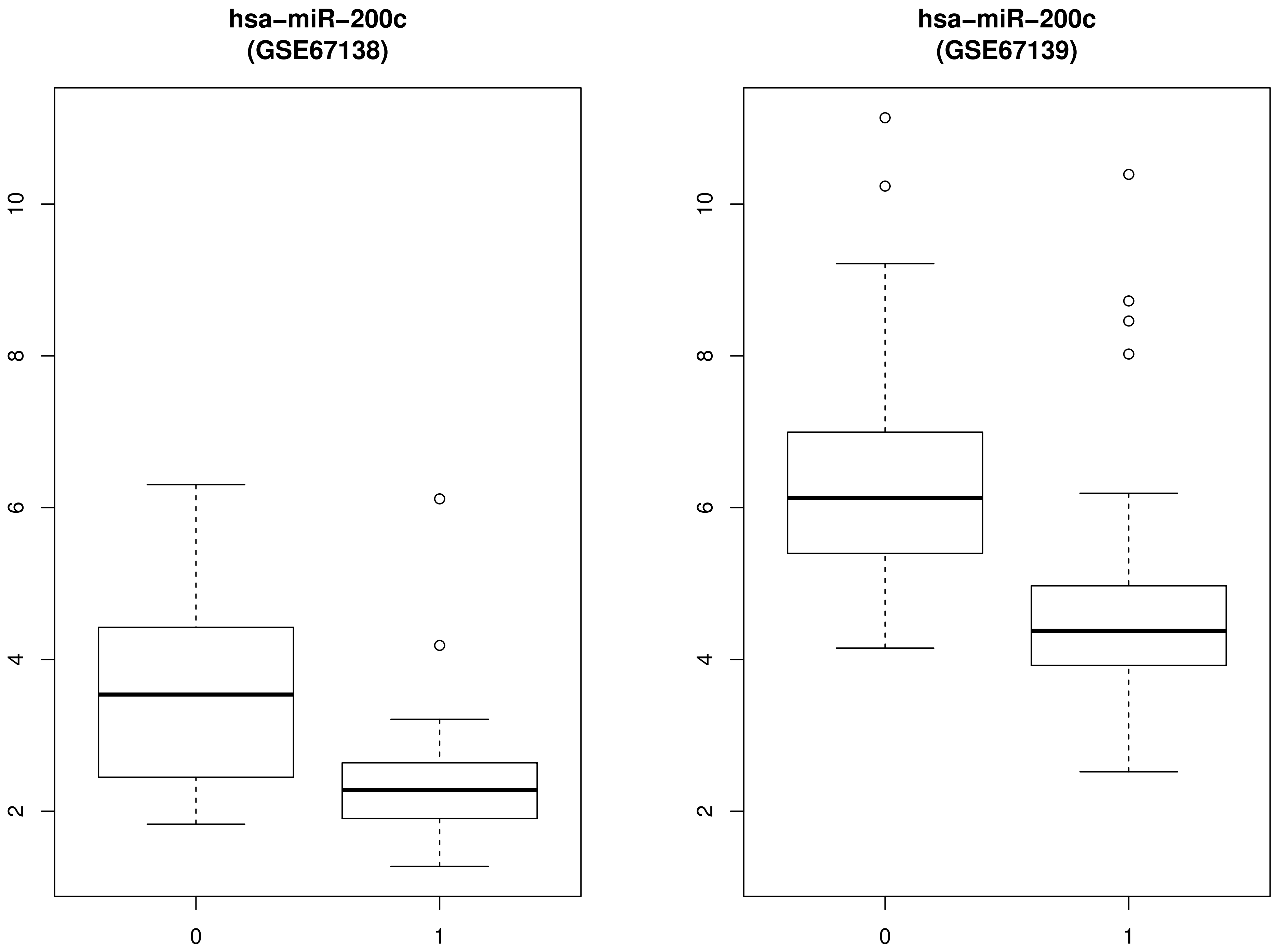
hsa−miR−200c
(GSE67138)
hsa−miR−200c
(GSE67139)
2
4
6
8
10
0
1

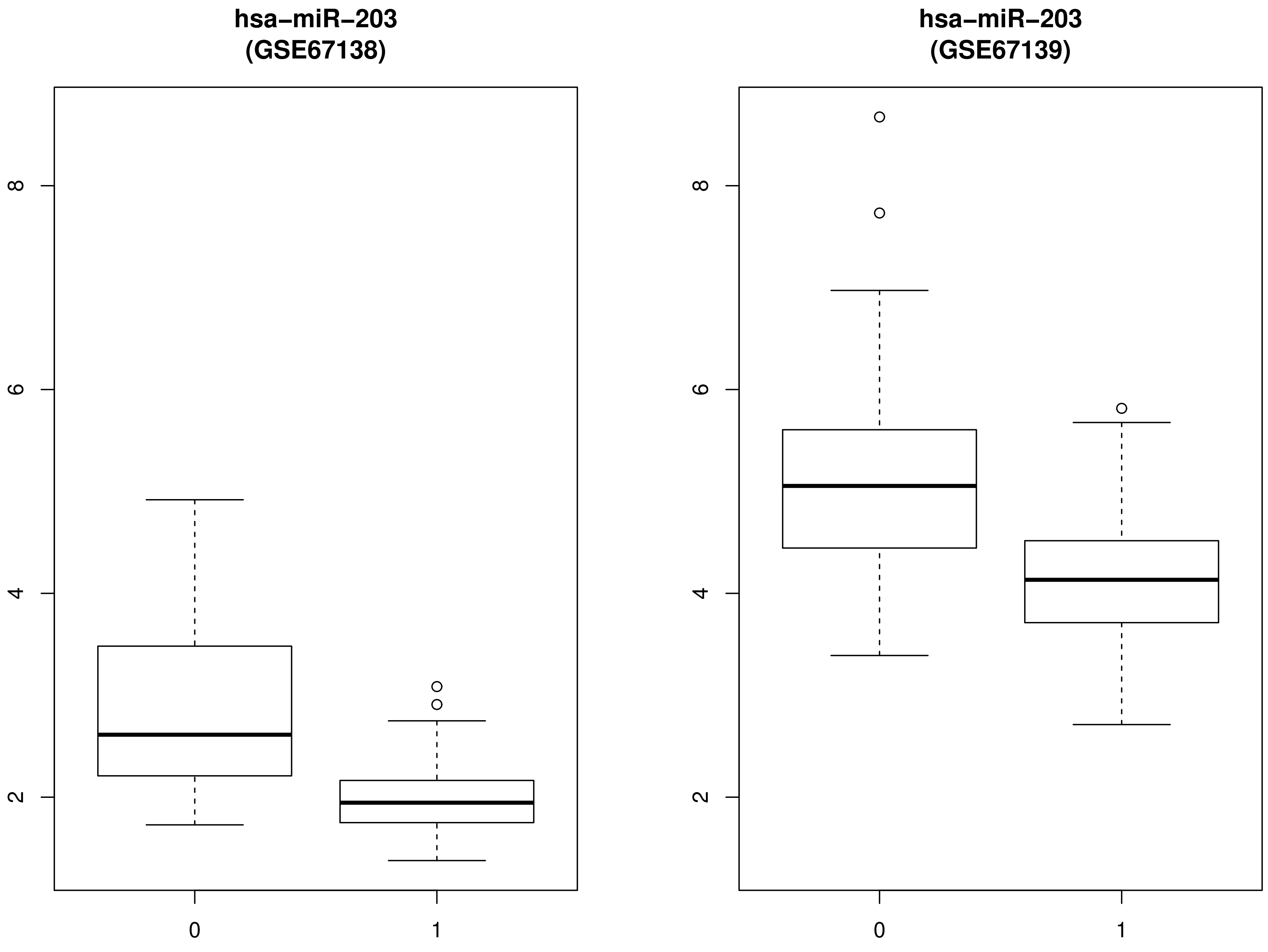
hsa−miR−203
(GSE67138)
hsa−miR−203
(GSE67139)
8
6
4
2
0
1

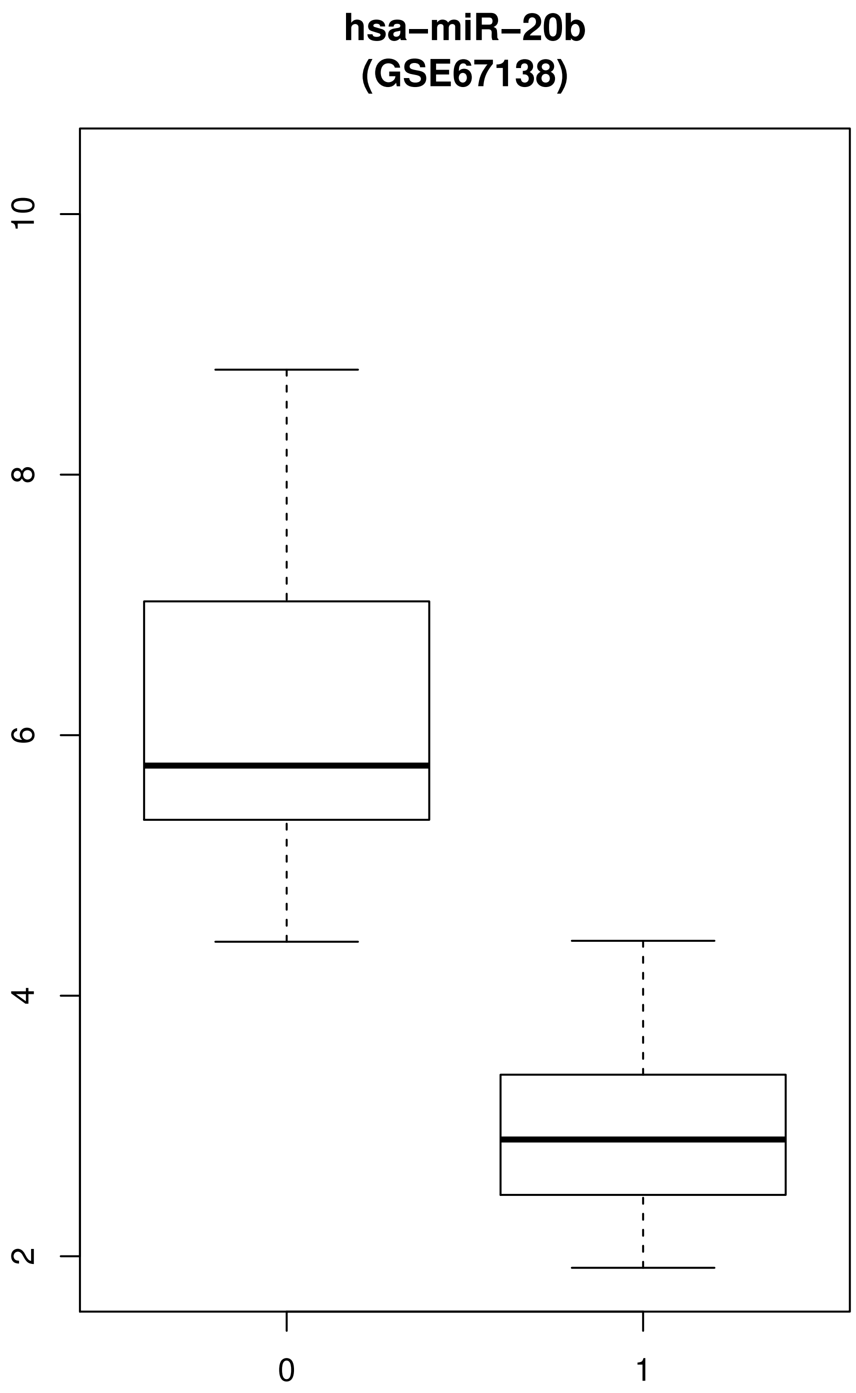
hsa−miR−20b
(GSE67138)
10
8
6
4
2
0
1

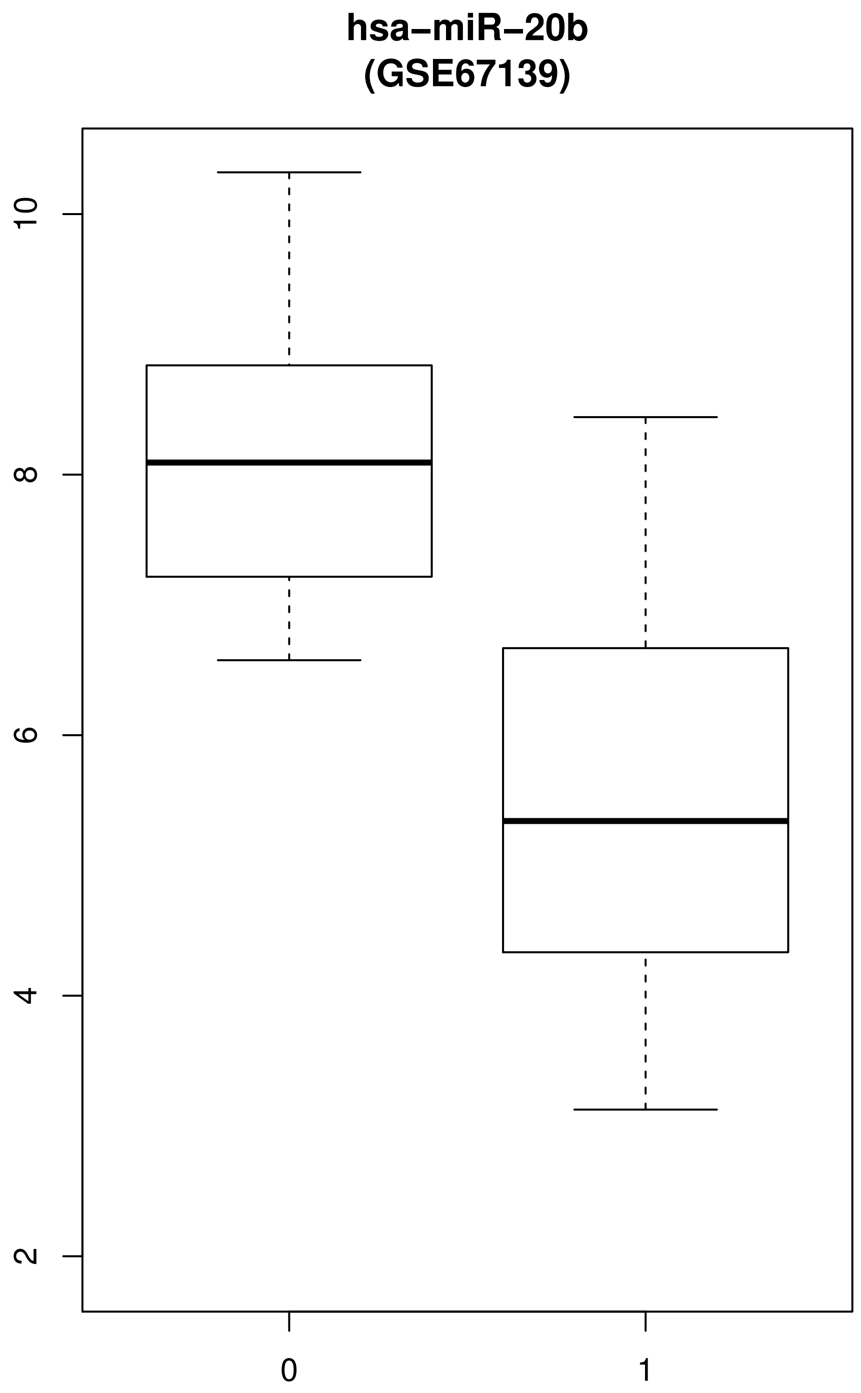
hsa−miR−20b
(GSE67139)
10
8
6
4
2
0
1

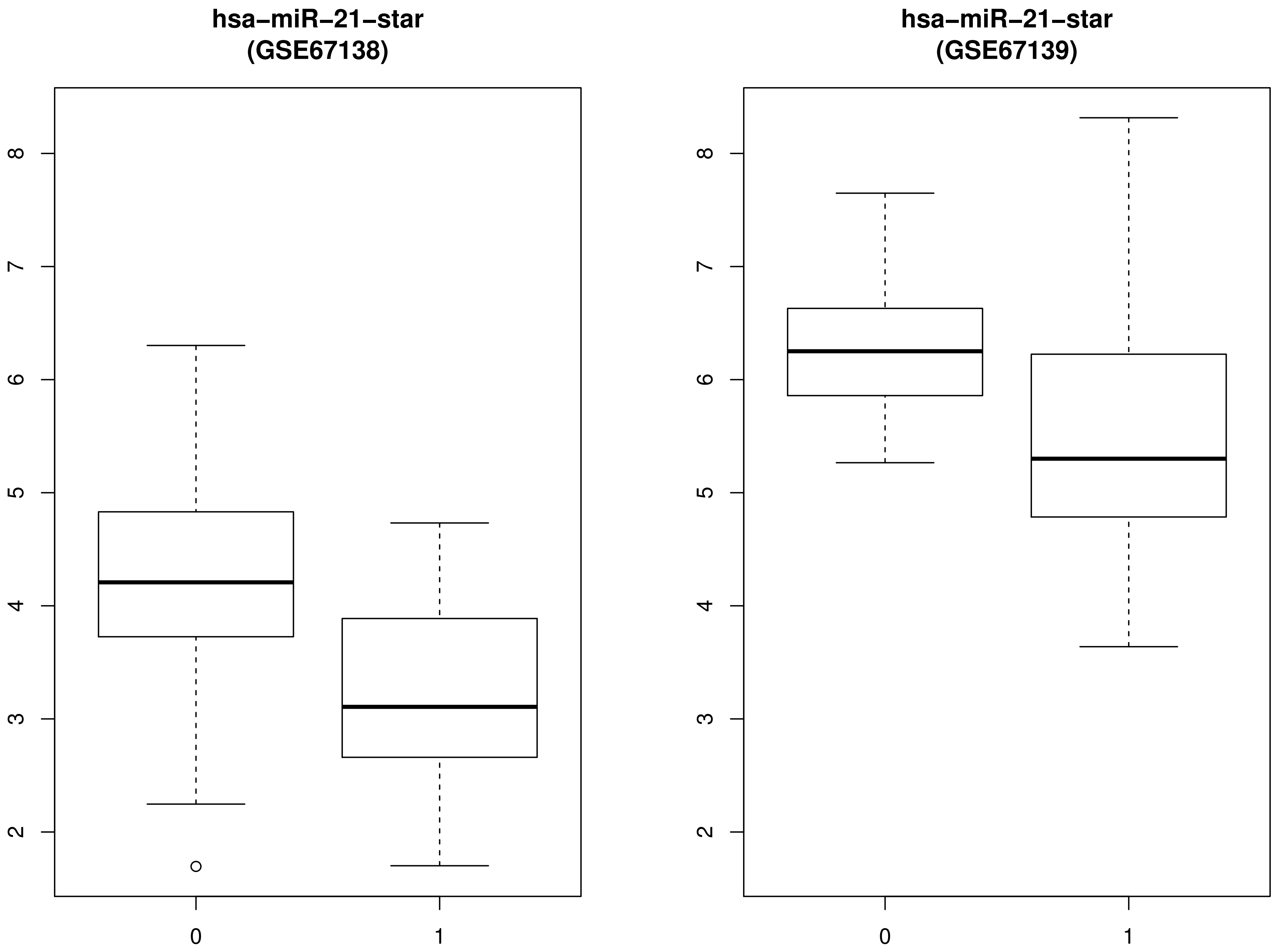

hsa−miR−21−star
(GSE67138)
hsa−miR−21−star
(GSE67139)
2
3
4
5
6
7
8
0
1

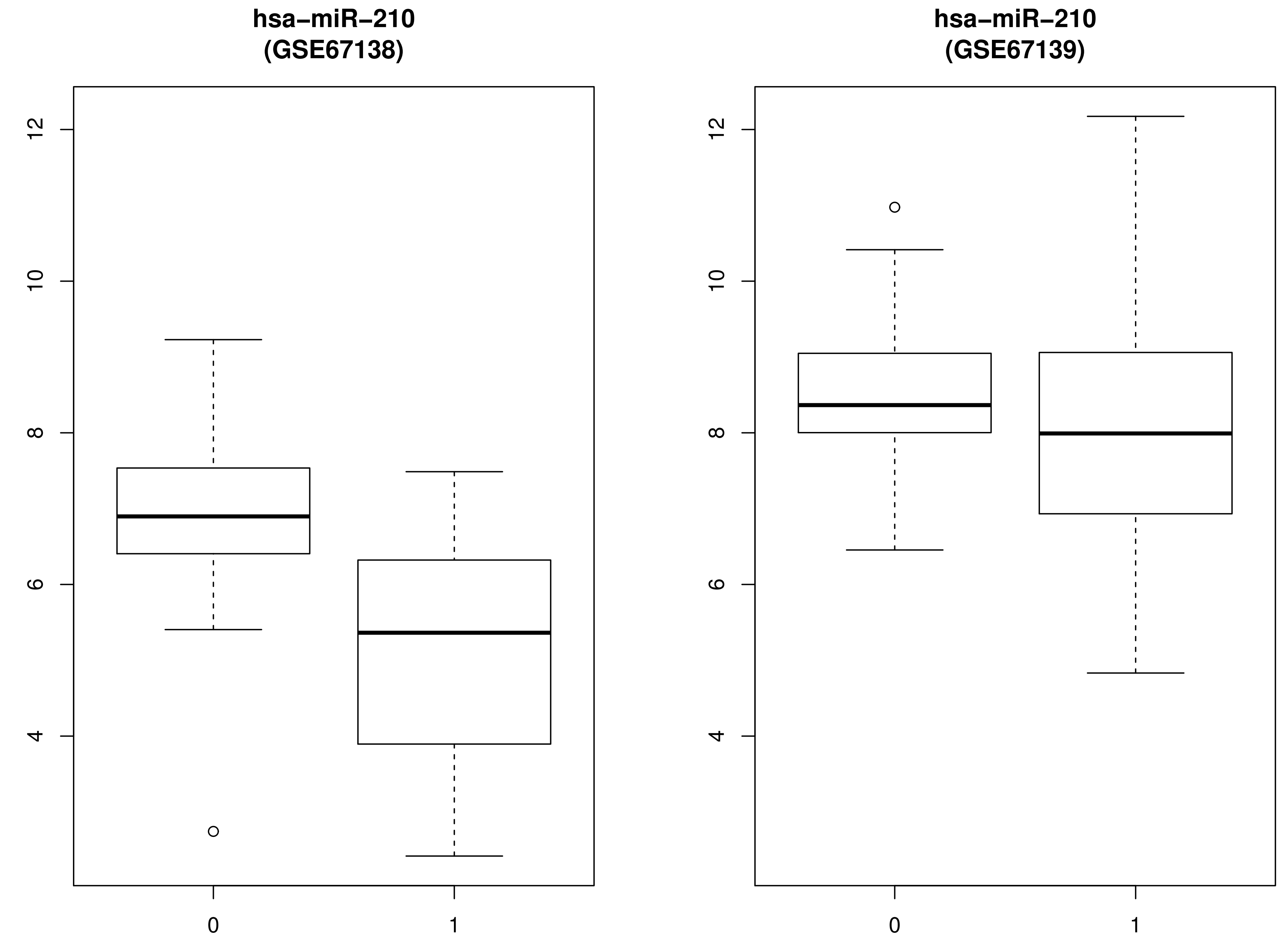
hsa−miR−210
(GSE67138)
hsa−miR−210
(GSE67139)
12
10
8
6
4
0
1

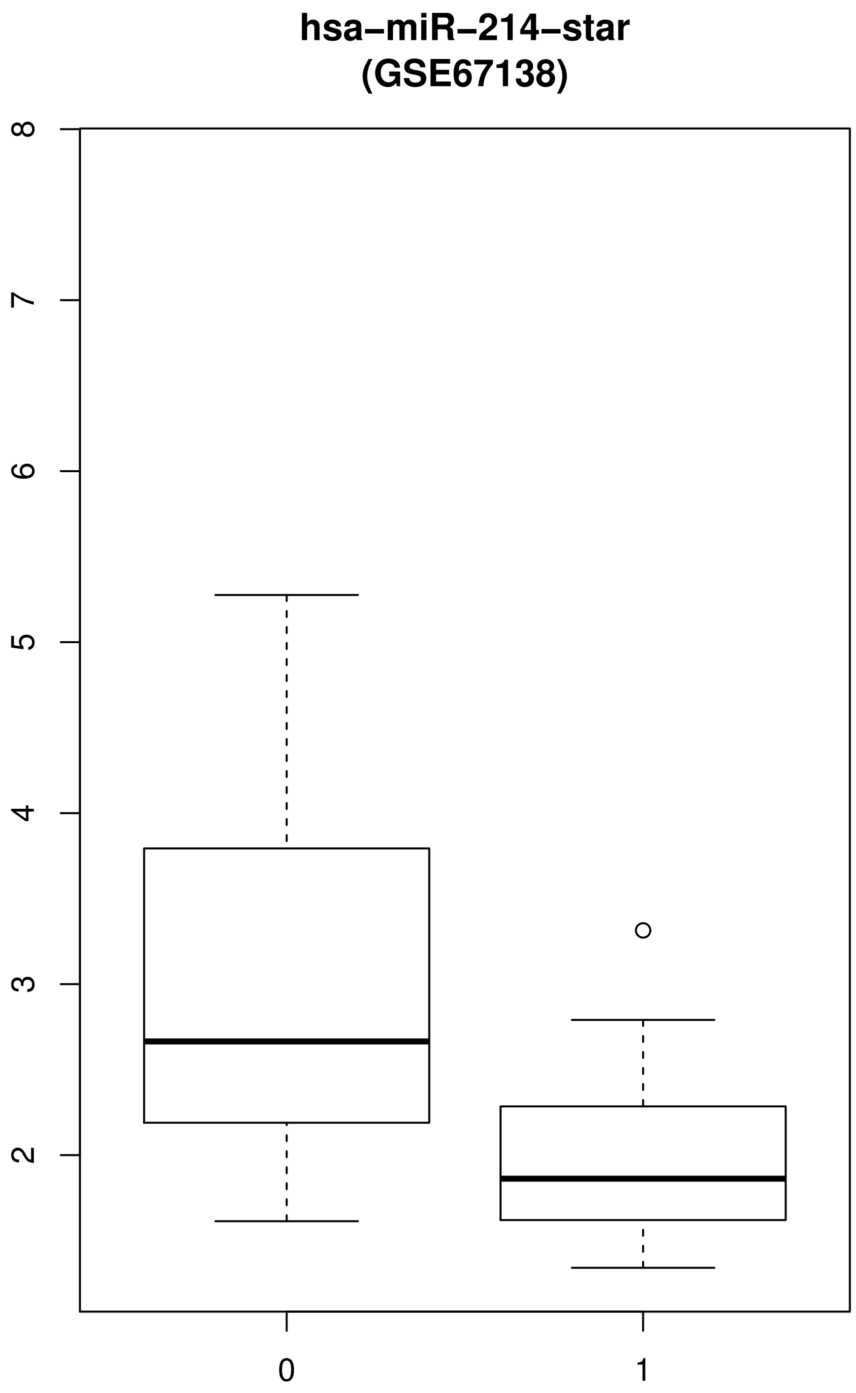

hsa−miR−214−star
(GSE67138)
8
7
6
5
4
3
2
0
1

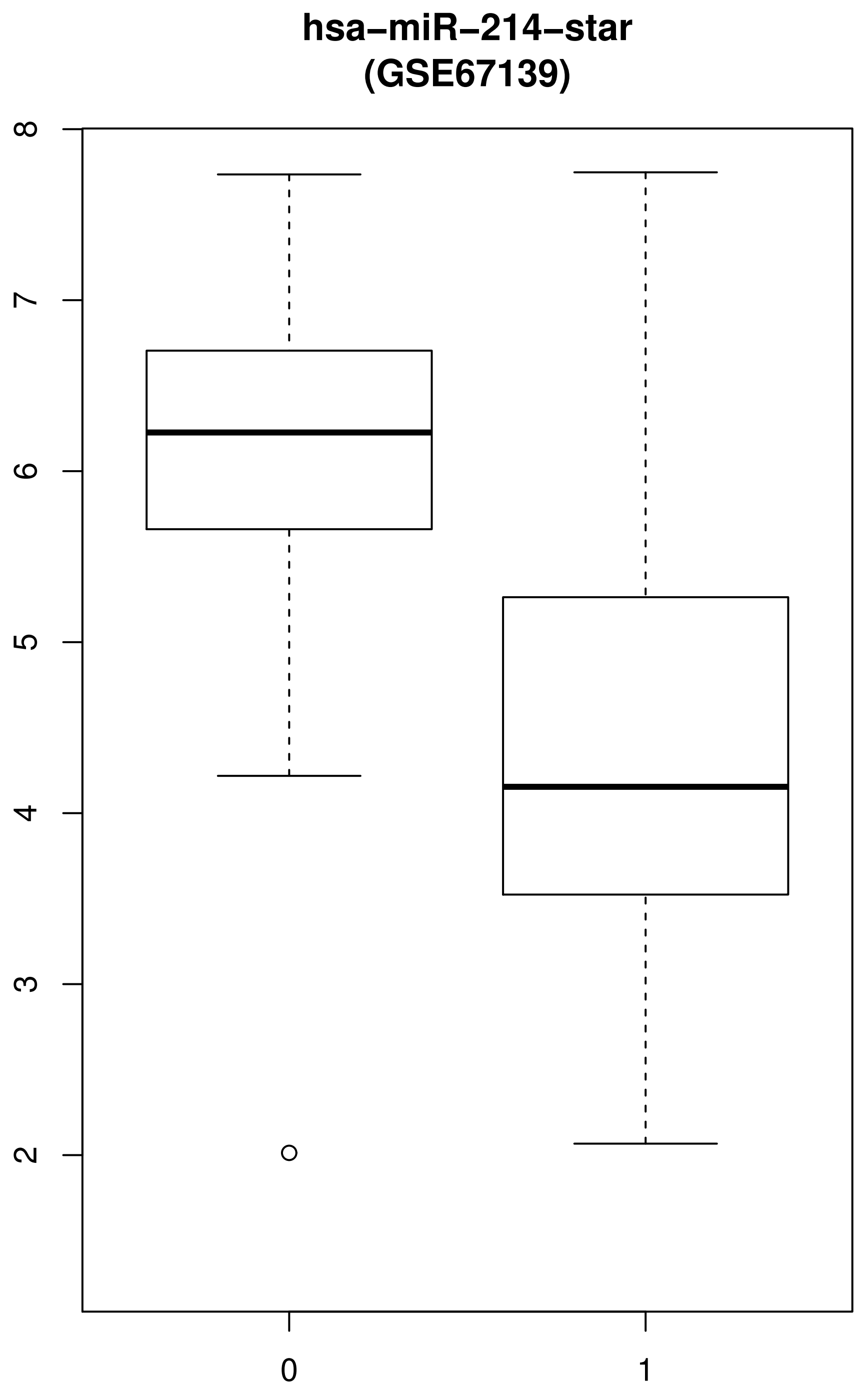

hsa−miR−214−star
(GSE67139)
8
7
6
5
4
3
2
0
1

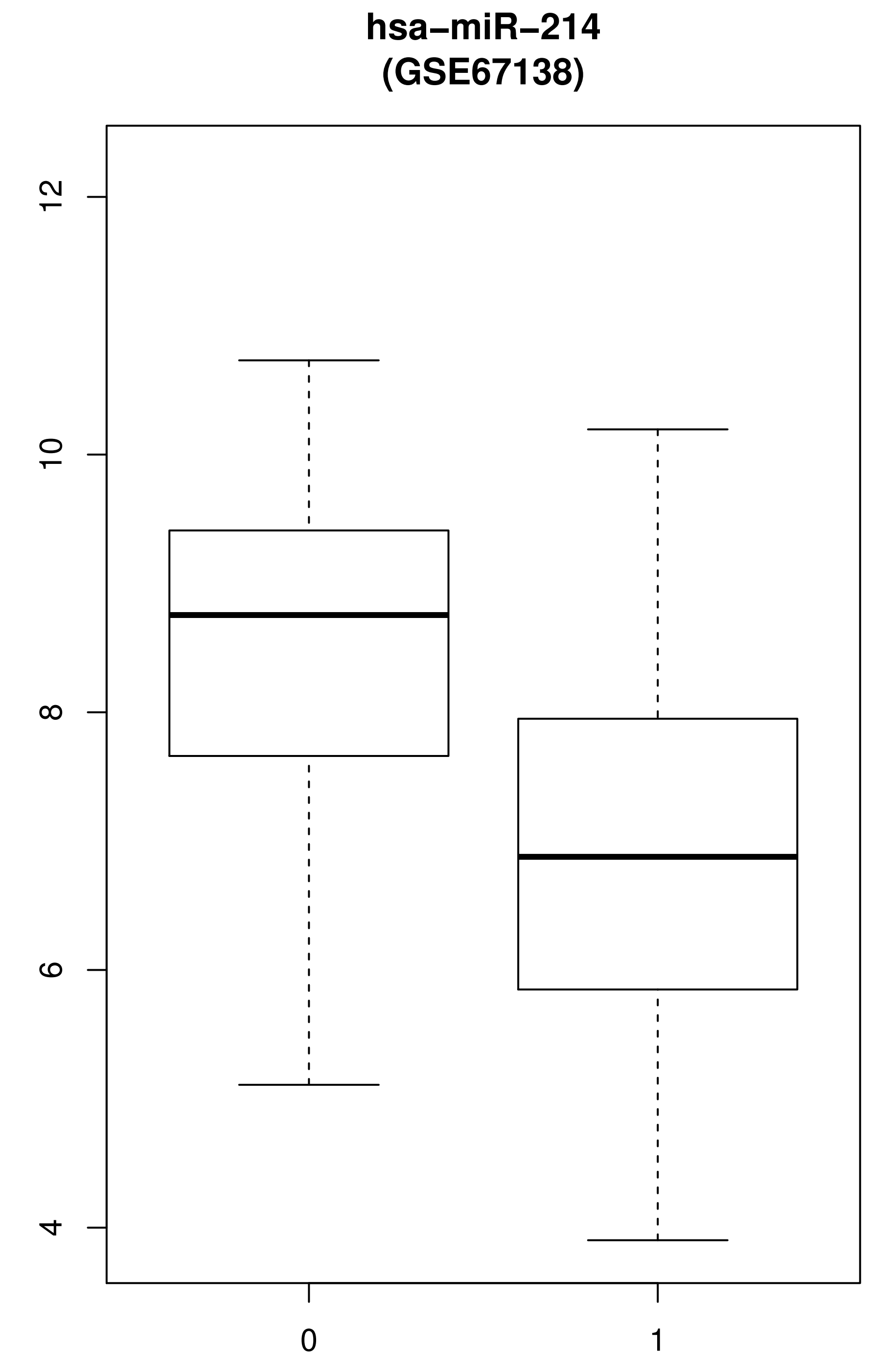

hsa−miR−214
(GSE67138)
12
10
8
6
4
0
1

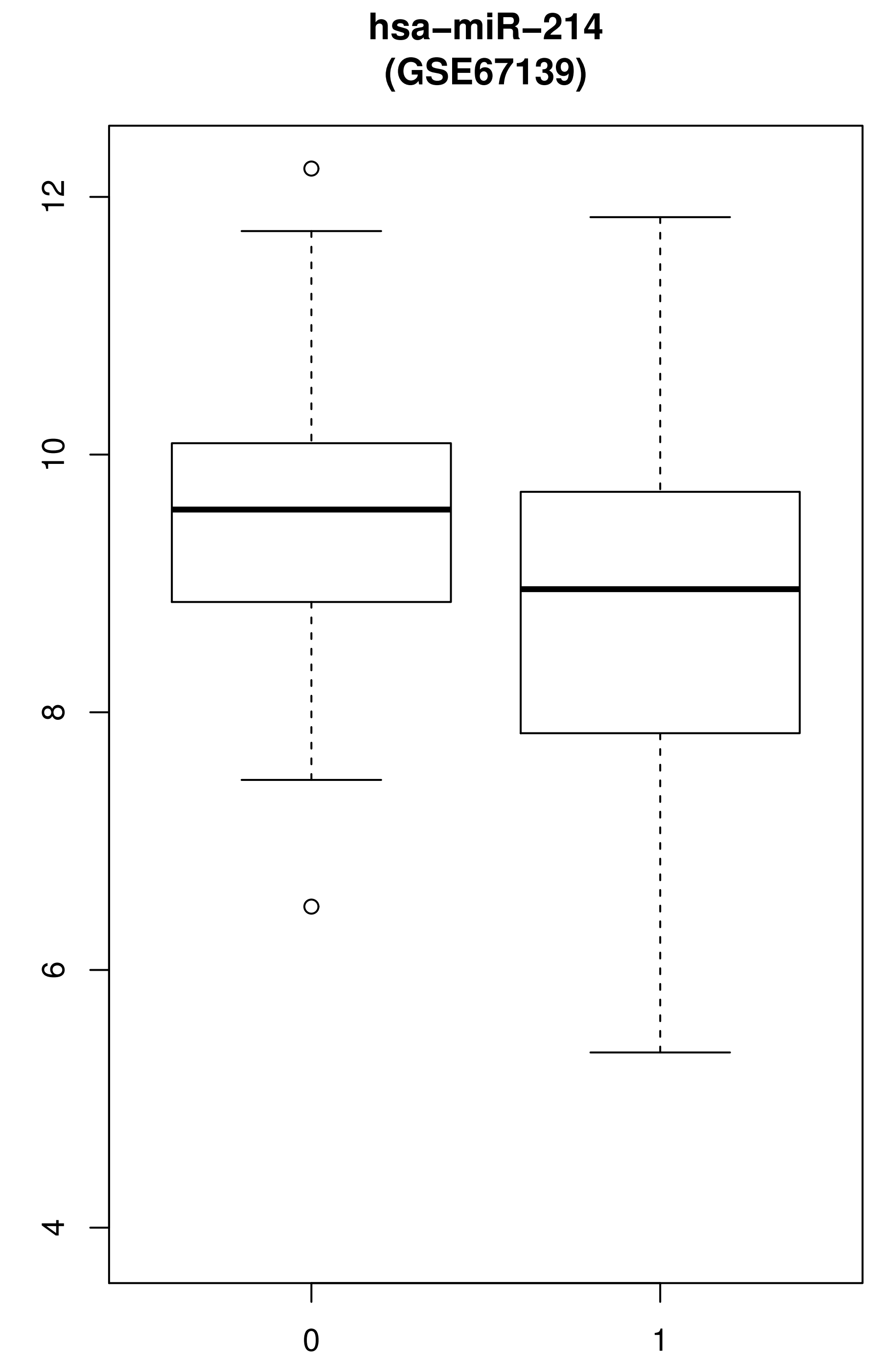

hsa−miR−214
(GSE67139)
12
10
8
6
4
0
1

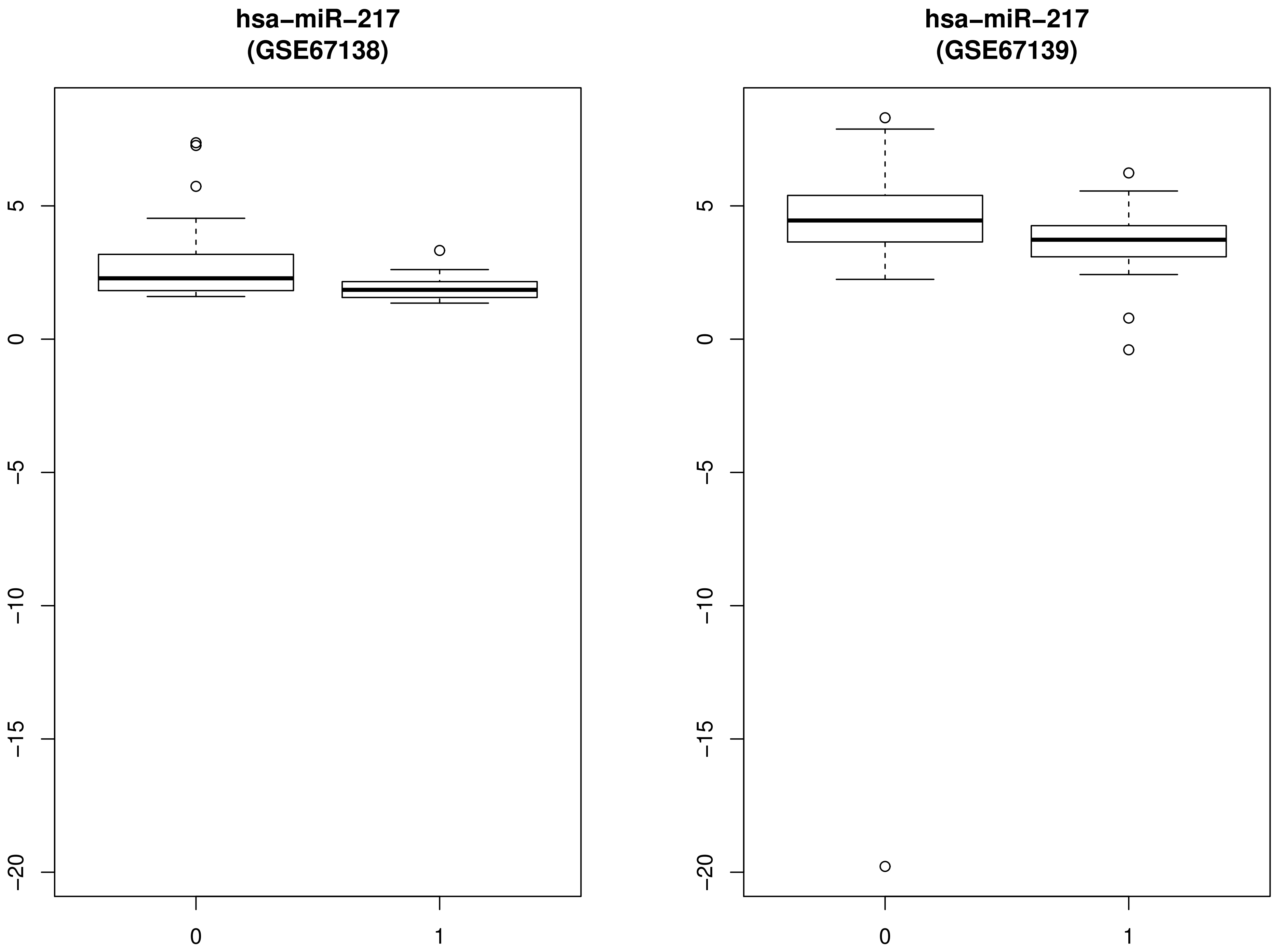
hsa−miR−217
(GSE67138)
hsa−miR−217
(GSE67139)
5
0
−5
−10
−15
−20
0
1

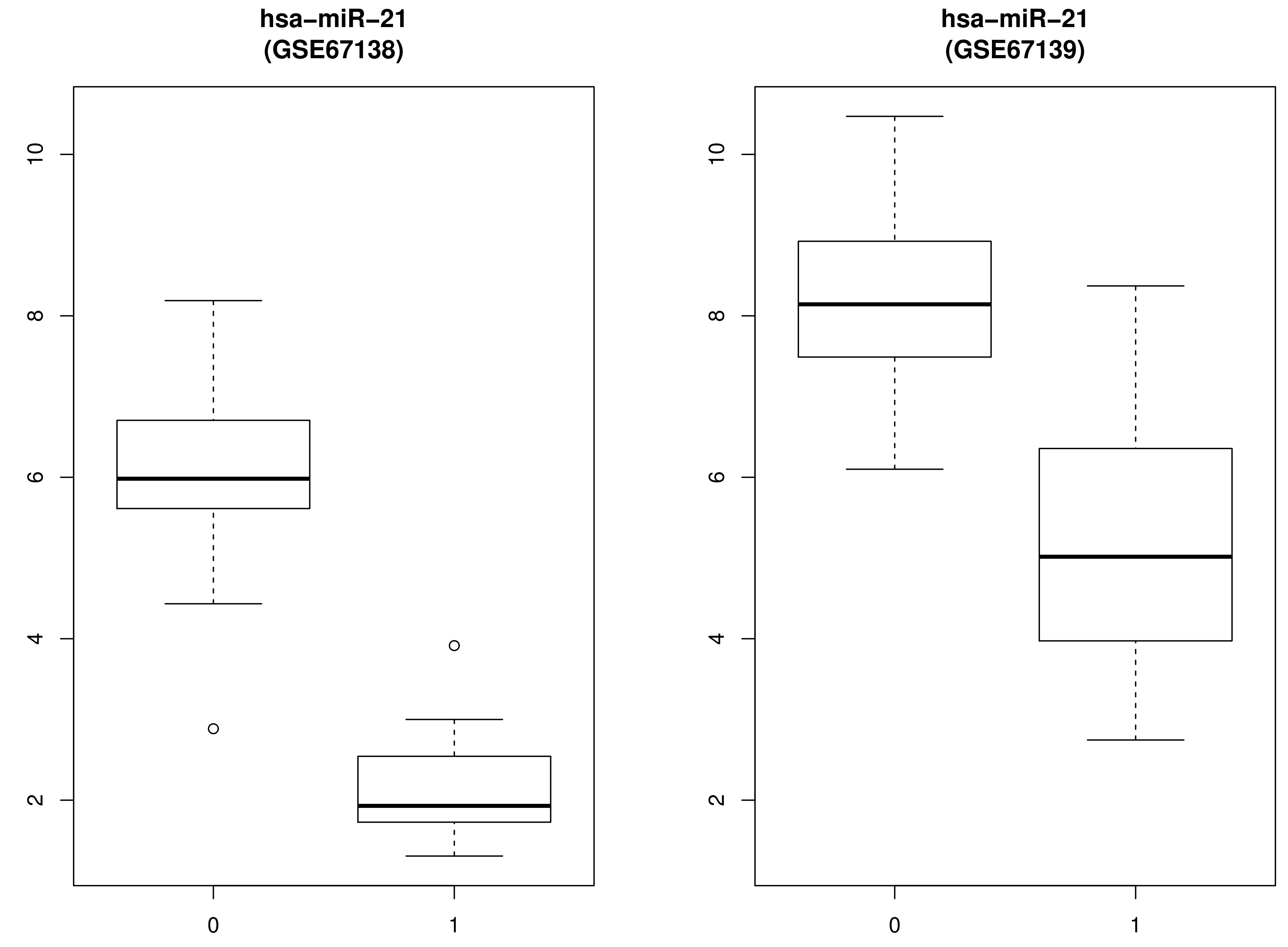
hsa−miR−21
(GSE67138)
hsa−miR−21
(GSE67139)
2
4
6
8
10
0
1
2
4
6
8
10
0
1

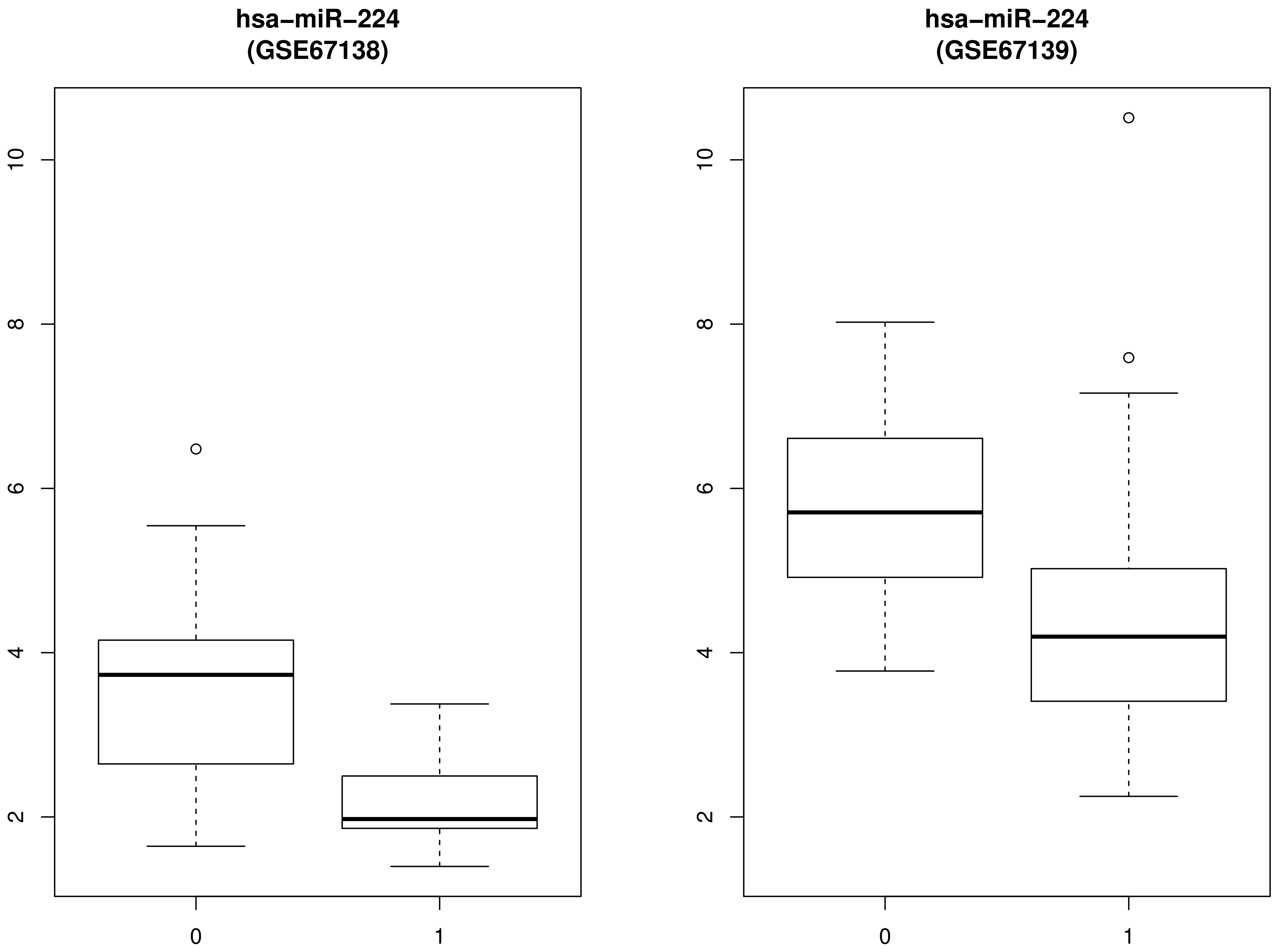
hsa−miR−224
(GSE67138)
hsa−miR−224
(GSE67139)
2
4
6
8
10
0
1
2
4
6
8
10
0
1

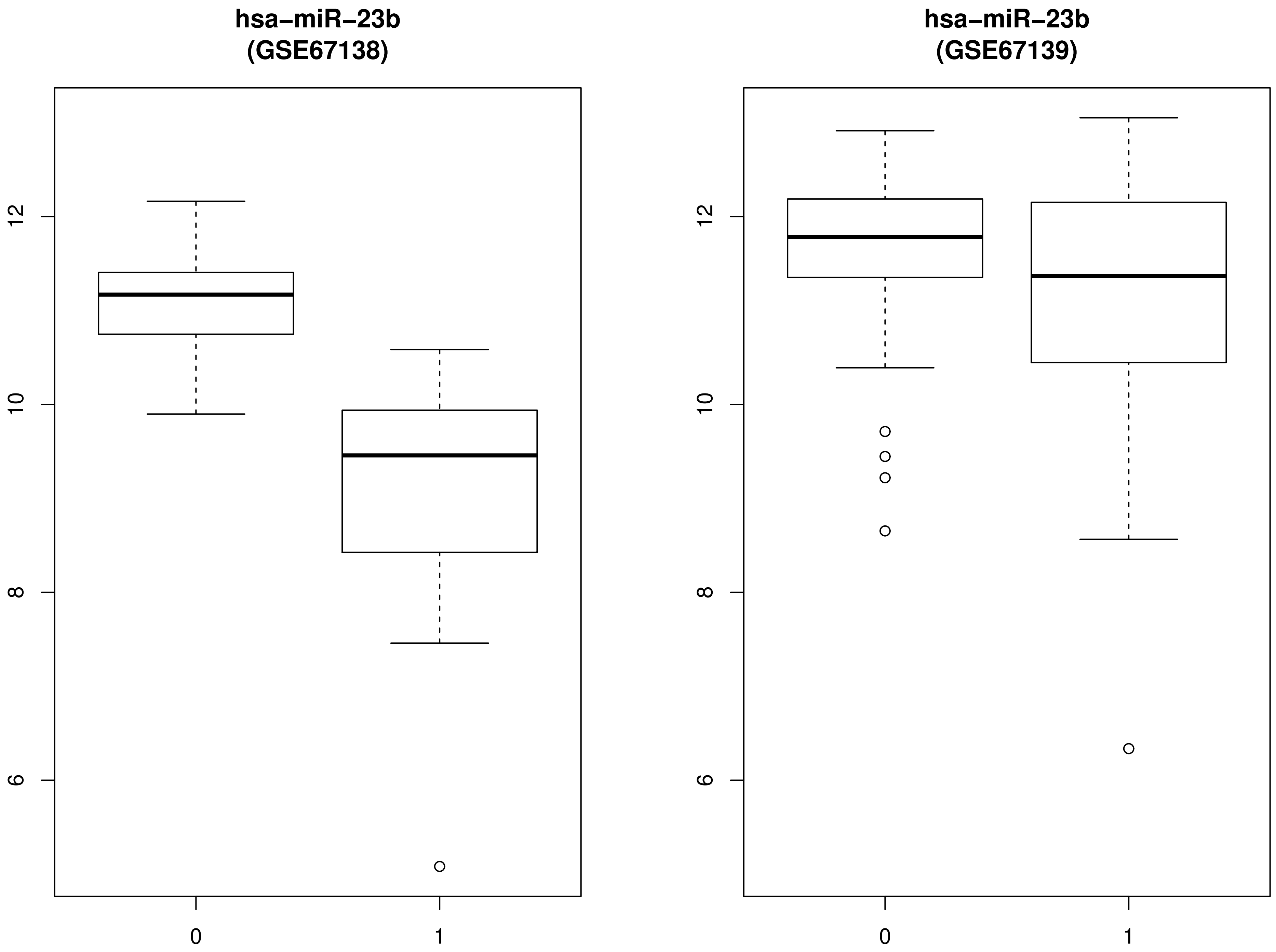
hsa−miR−23b
(GSE67138)
hsa−miR−23b
(GSE67139)
12
10
8
6
0
1

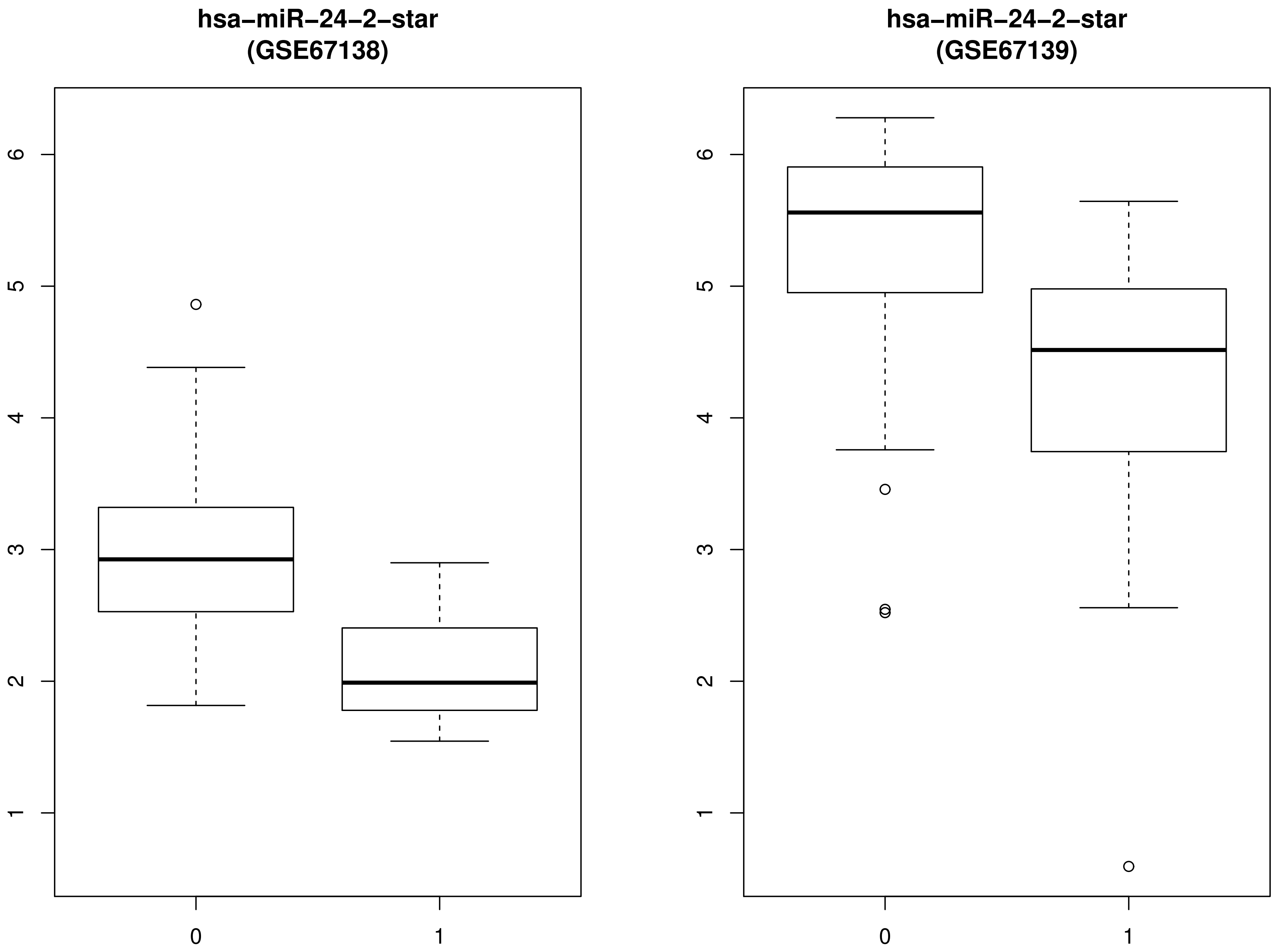
hsa−miR−24−2−star
(GSE67138)
hsa−miR−24−2−star
(GSE67139)
1
2
3
4
5
6
0
1

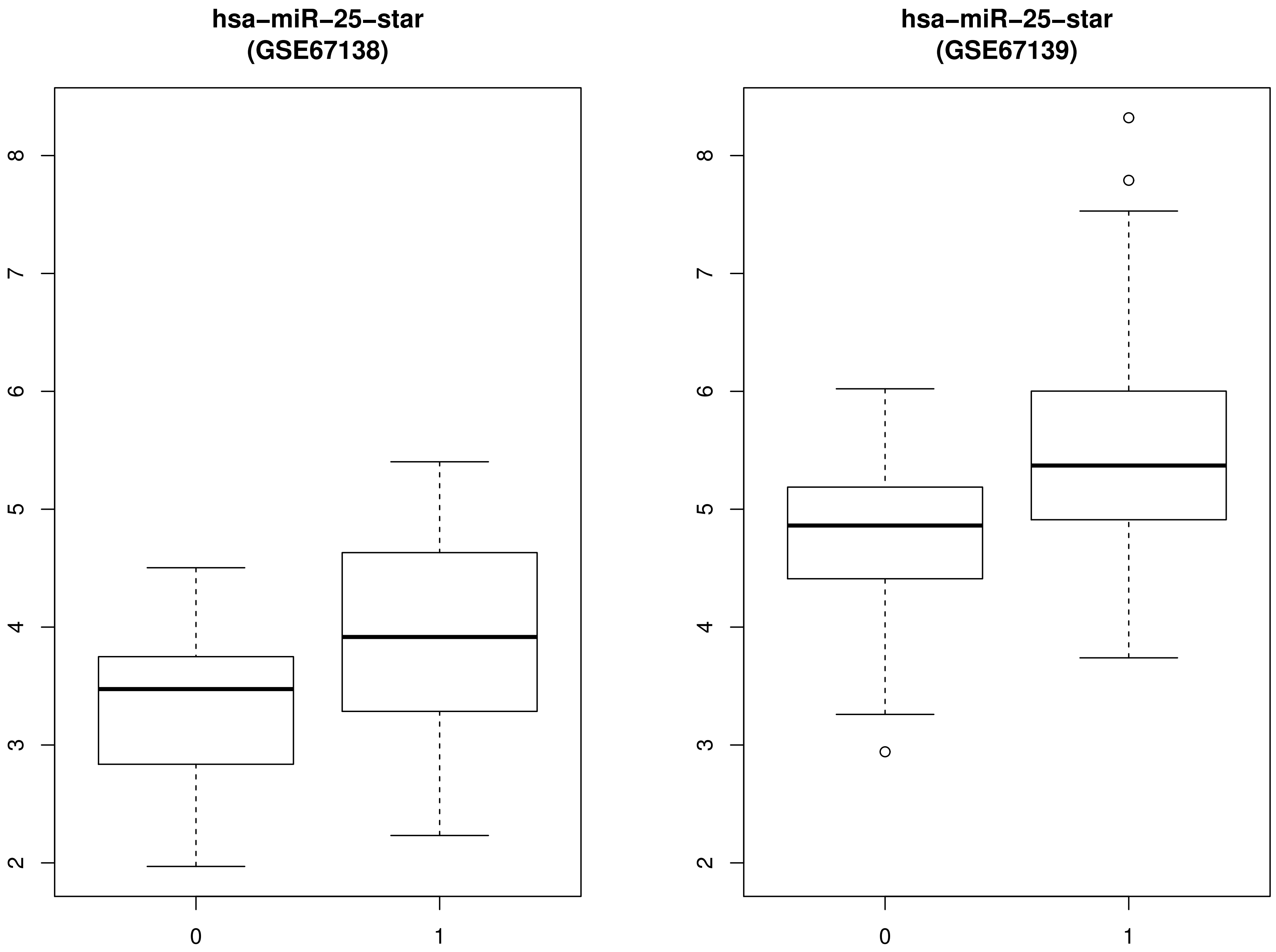
hsa-miR-25-star
(GSE67138)
hsa-miR-25-star
(GSE67139)
2
3
4
5
6
7
8
0
1

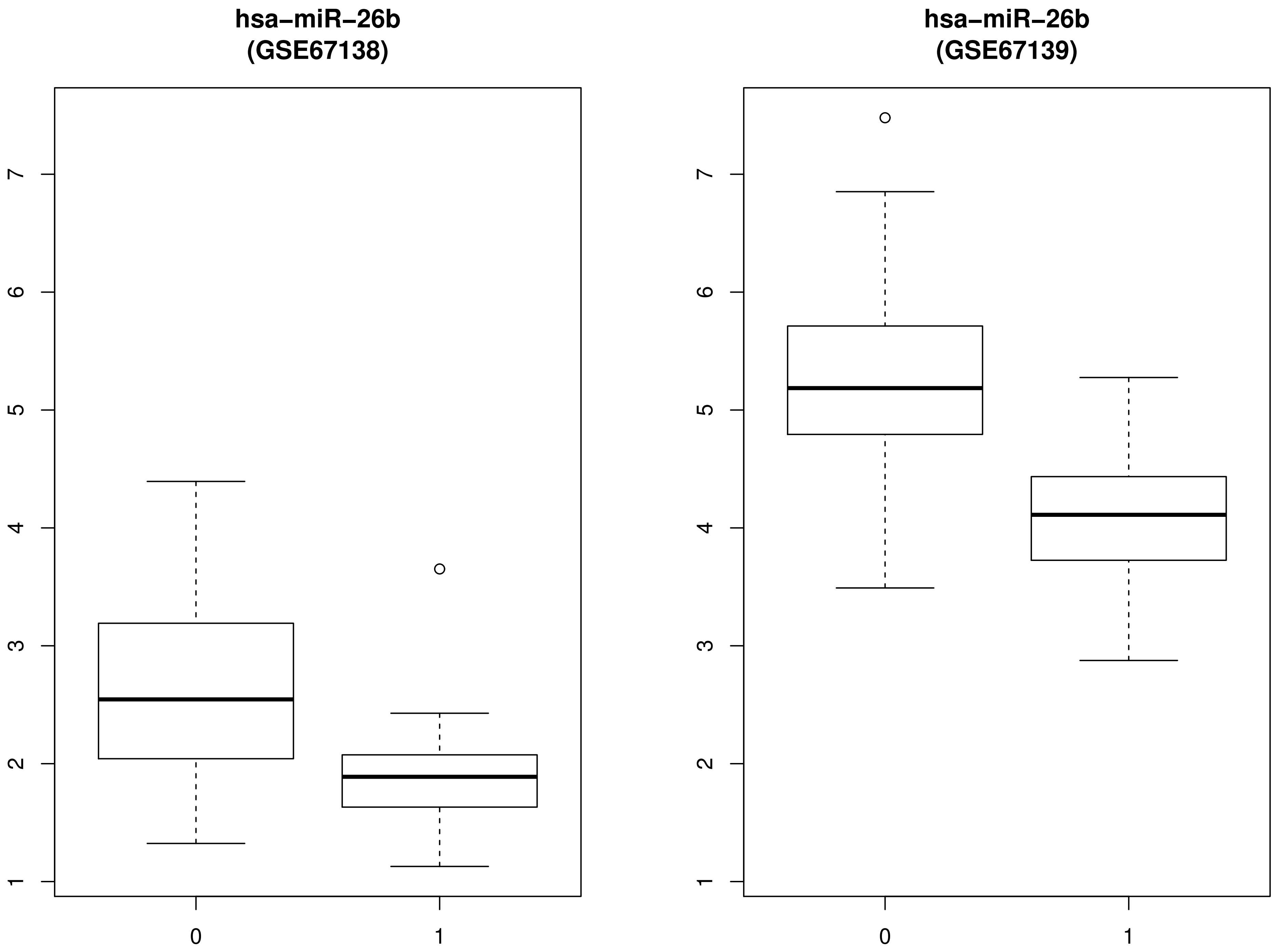
hsa−miR−26b
(GSE67138)
hsa−miR−26b
(GSE67139)
1
2
3
4
5
6
7
0
1

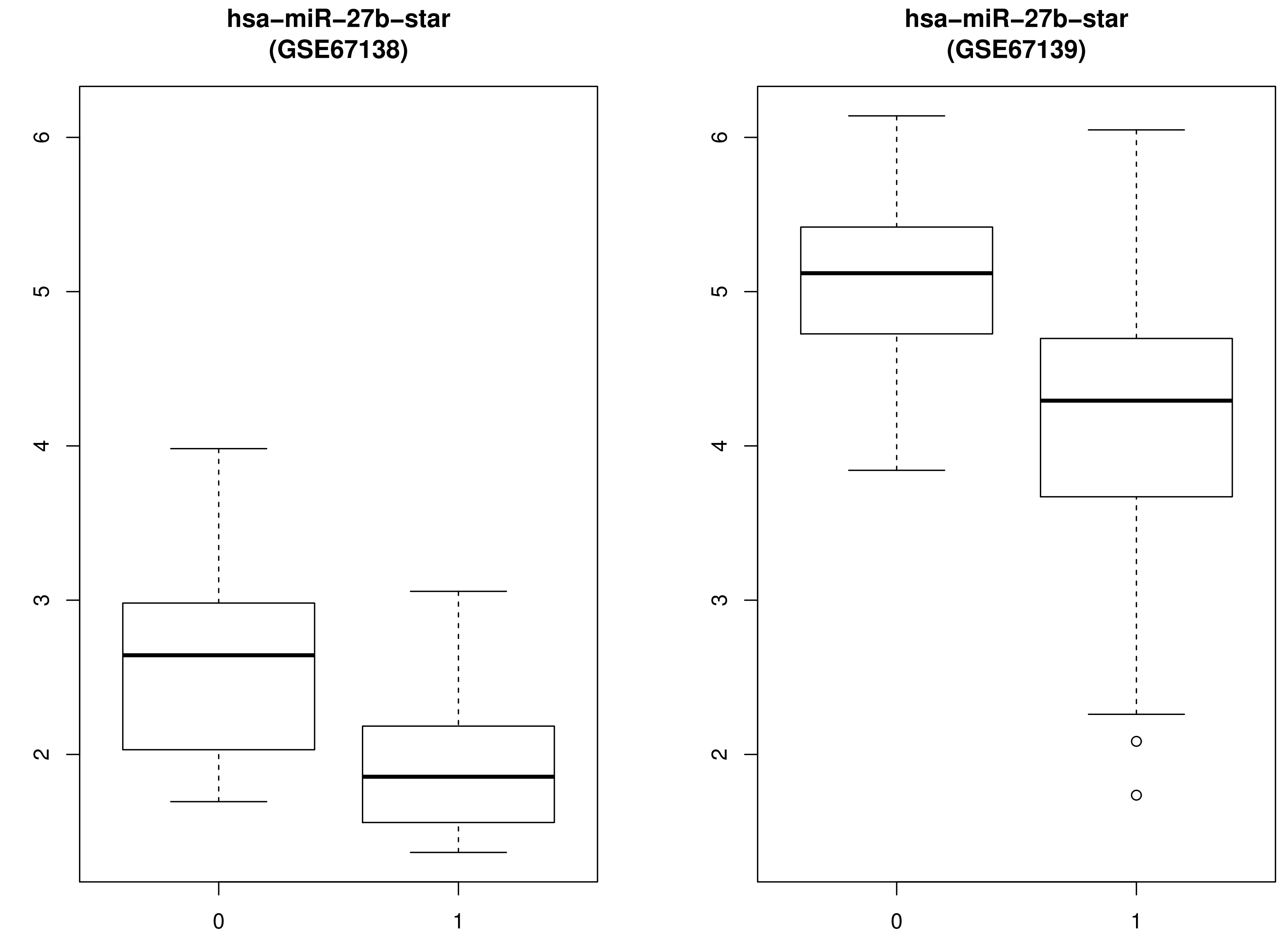
hsa−miR−27b−star
(GSE67138)
hsa−miR−27b−star
(GSE67139)
2
3
4
5
6
0
1

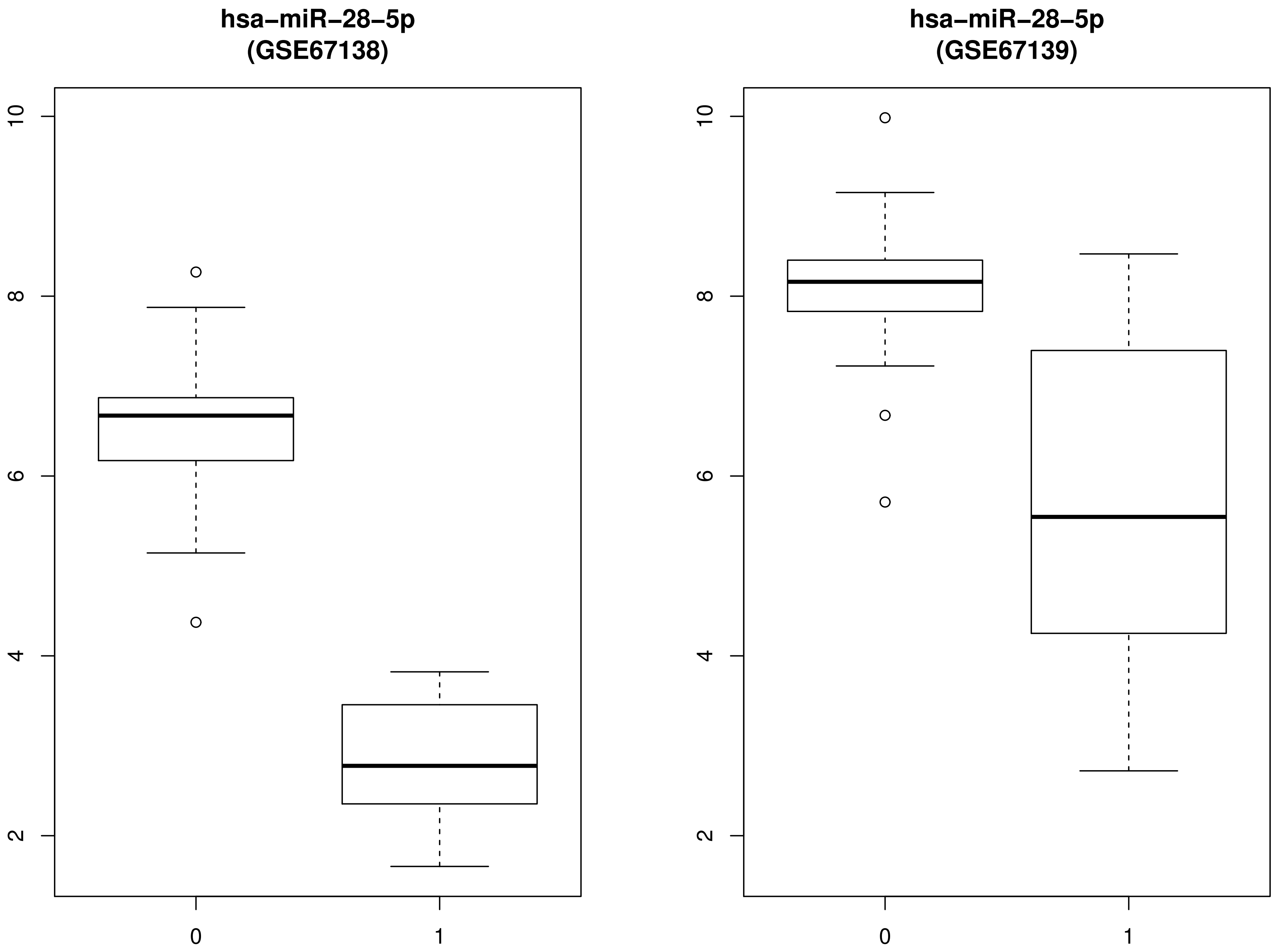
hsa−miR−28−5p
(GSE67138)
hsa−miR−28−5p
(GSE67139)
2
4
6
8
10
0
1

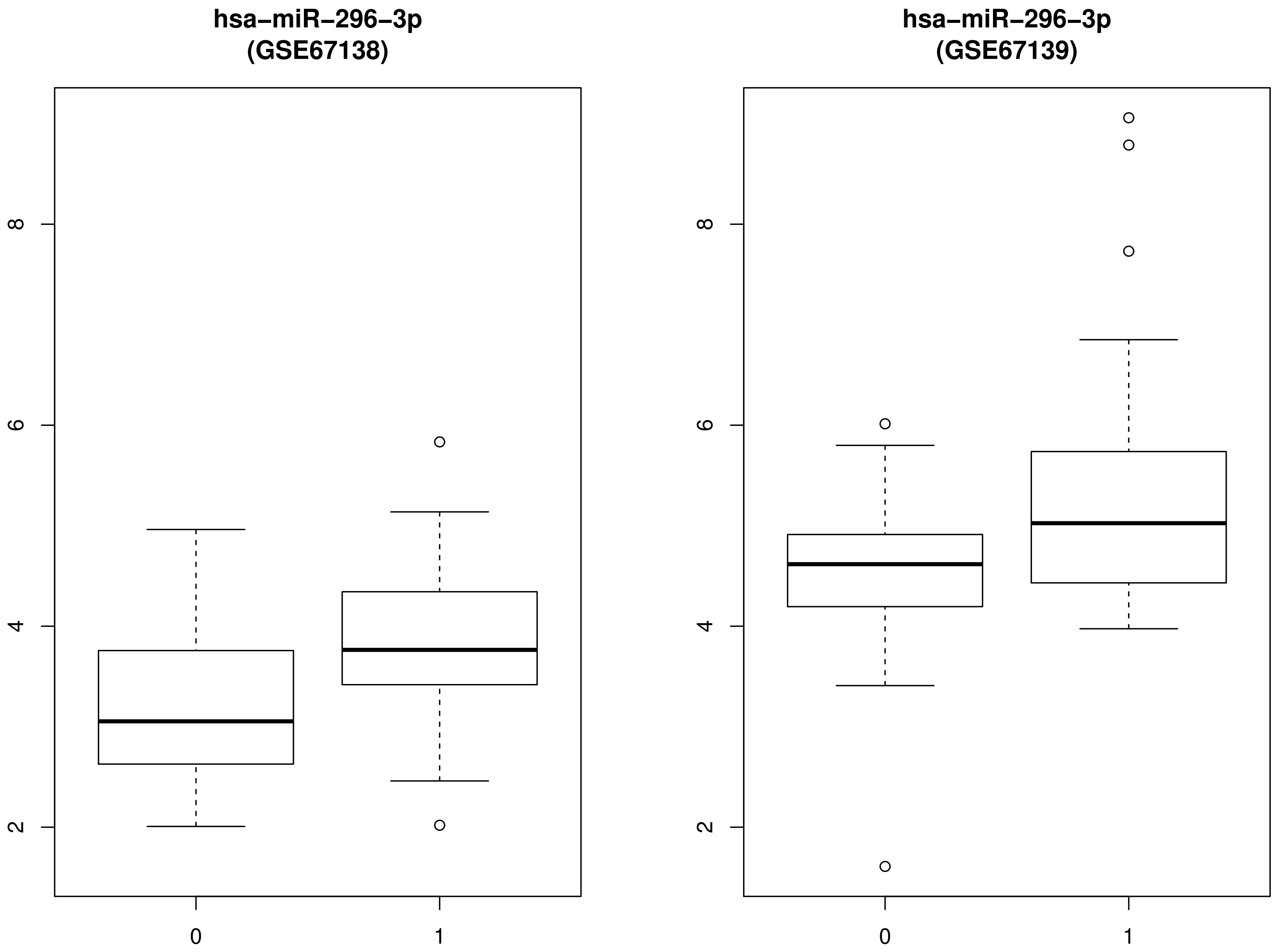
hsa−miR−296−3p
(GSE67138)
hsa−miR−296−3p
(GSE67139)
2
4
6
8
0
1

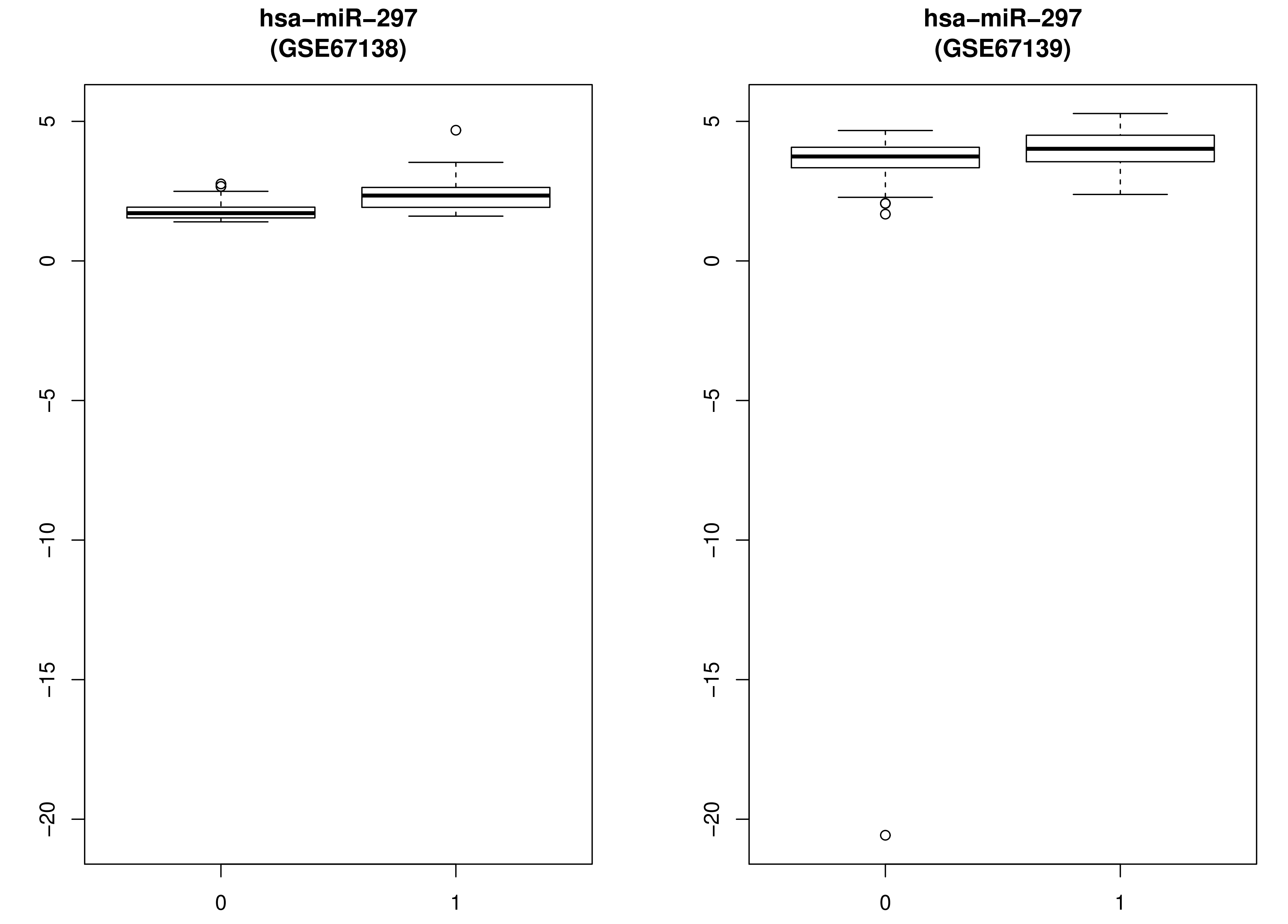

hsa−miR−297
(GSE67138)
hsa−miR−297
(GSE67139)
5
0
−5
−10
−15
−20
0
1

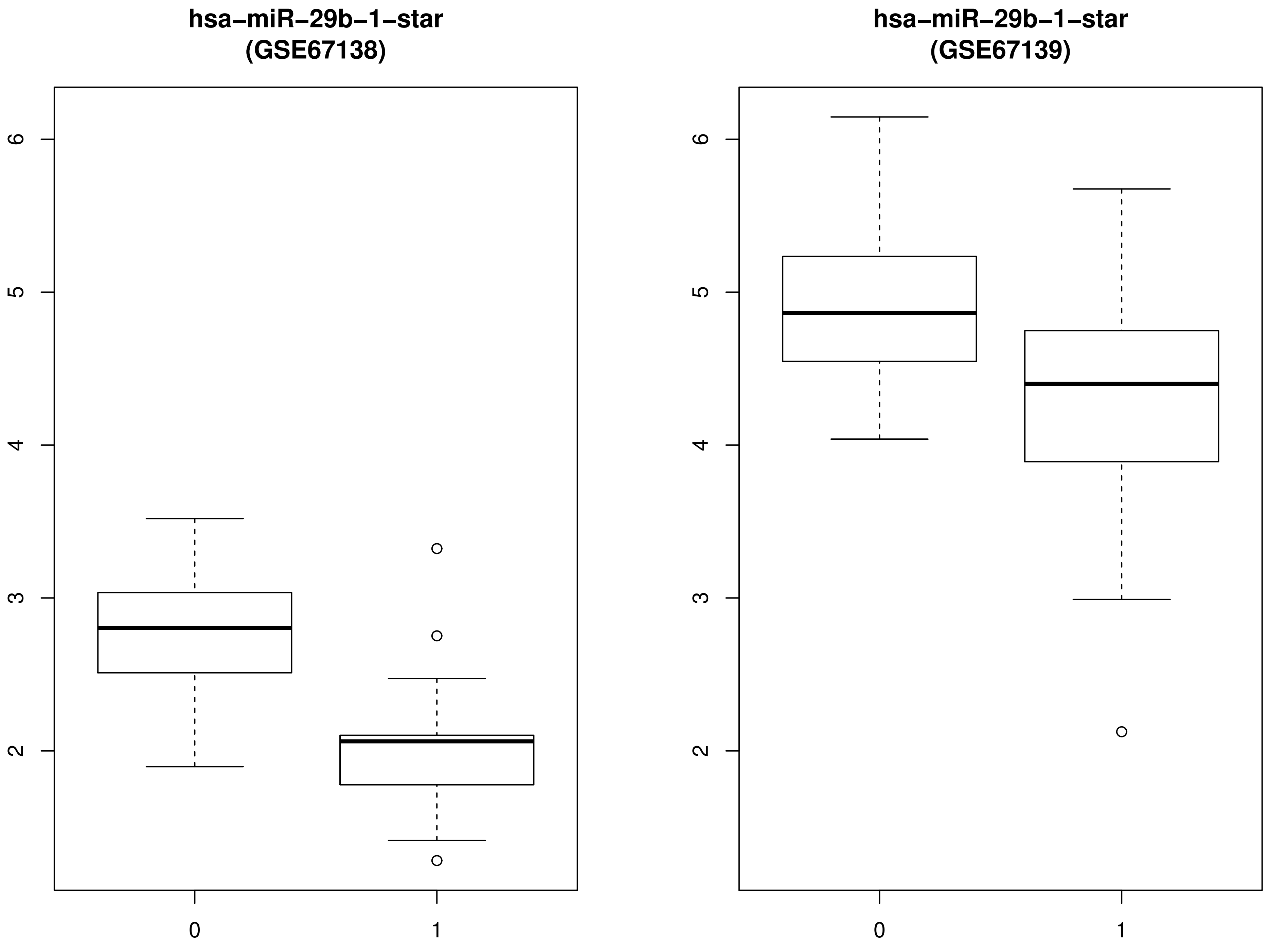

hsa−miR−29b−1−star
(GSE67138)
hsa−miR−29b−1−star
(GSE67139)
2
3
4
5
6
0
1

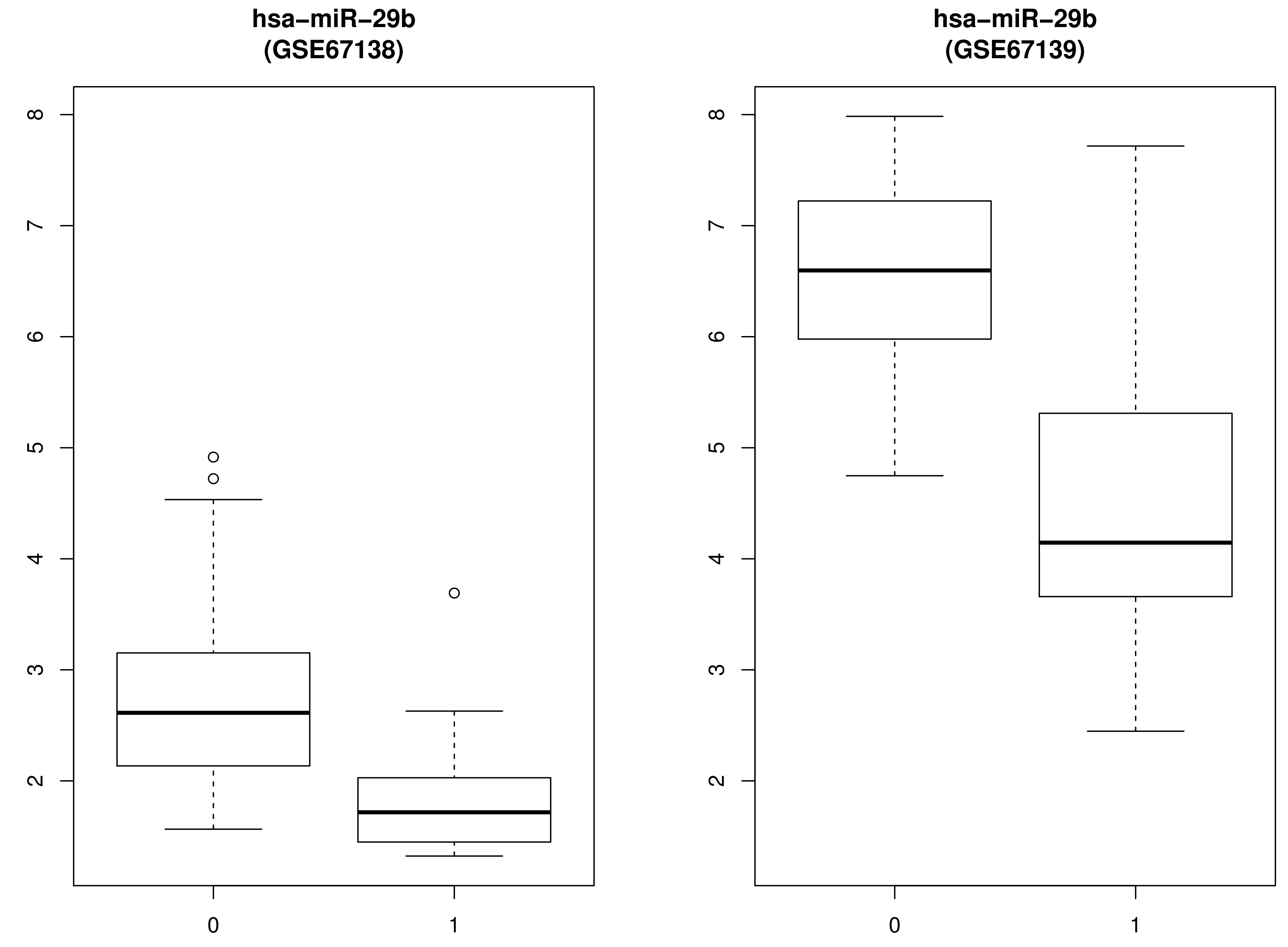
hsa−miR−29b
(GSE67138)
hsa−miR−29b
(GSE67139)
2
3
4
5
6
7
8
0
1

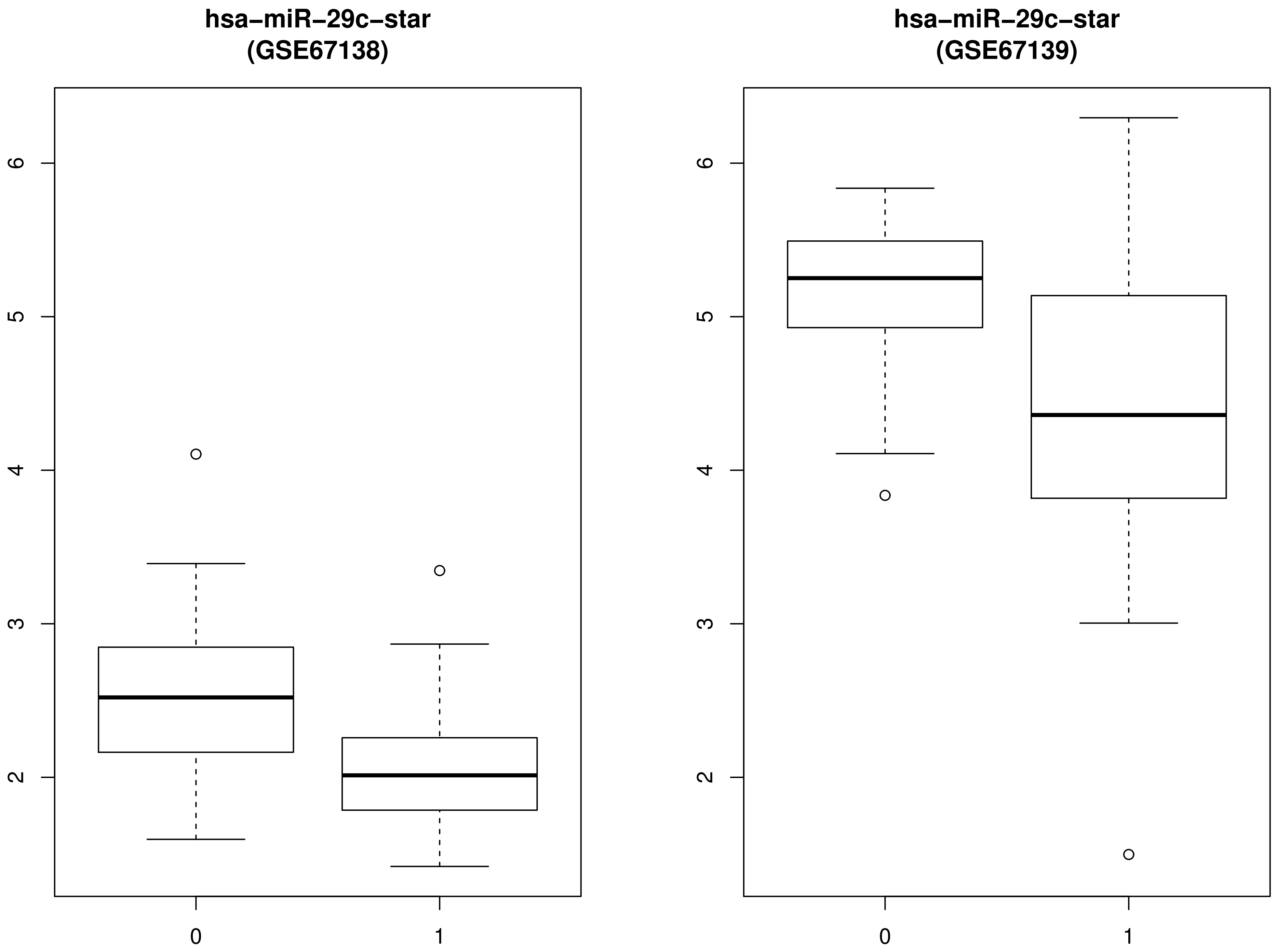
hsa−miR−29c−star
(GSE67138)
hsa−miR−29c−star
(GSE67139)
2
3
4
5
6
0
1

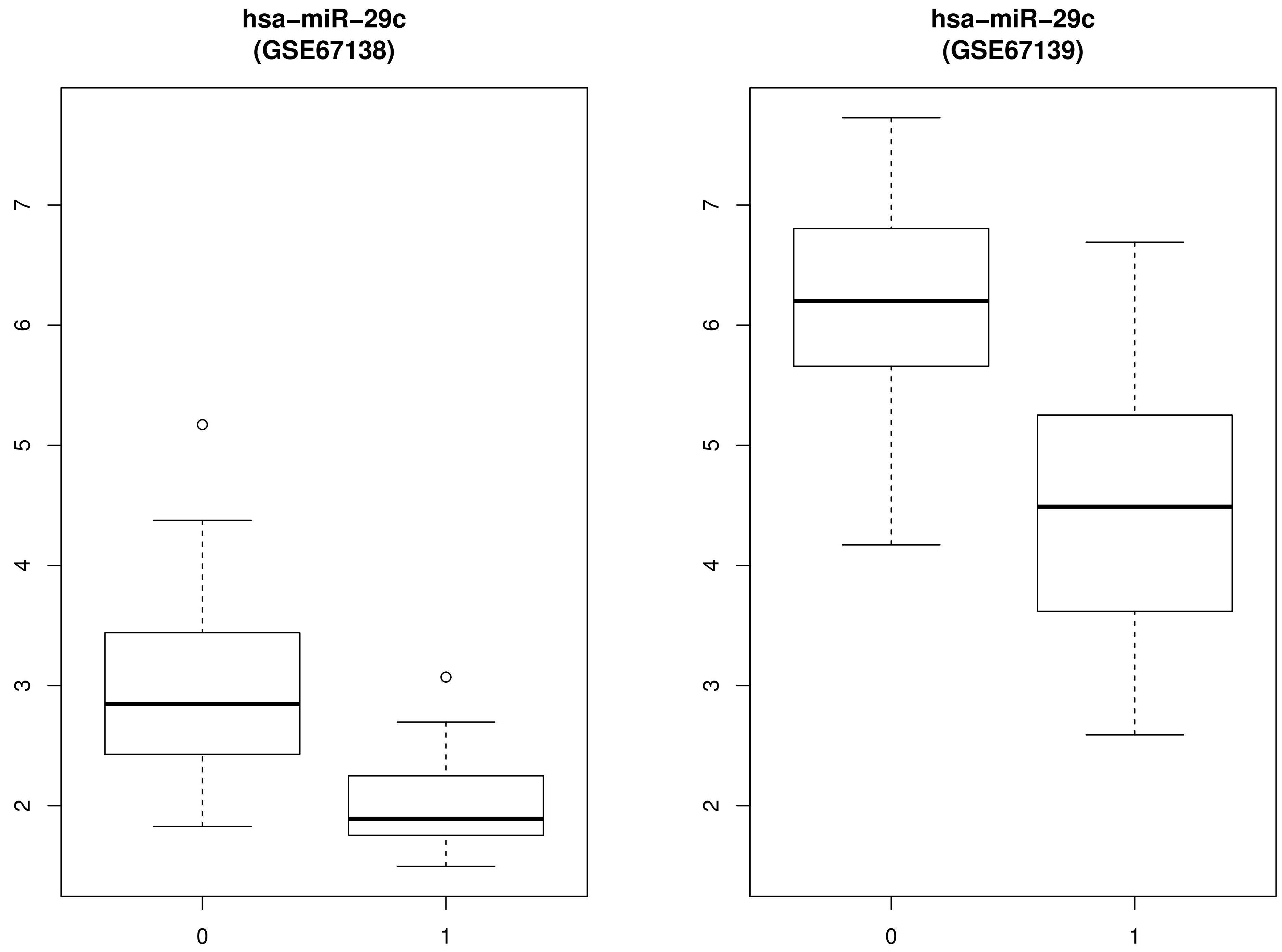
hsa−miR−29c
(GSE67138)
hsa−miR−29c
(GSE67139)
2
3
4
5
6
7
0
1

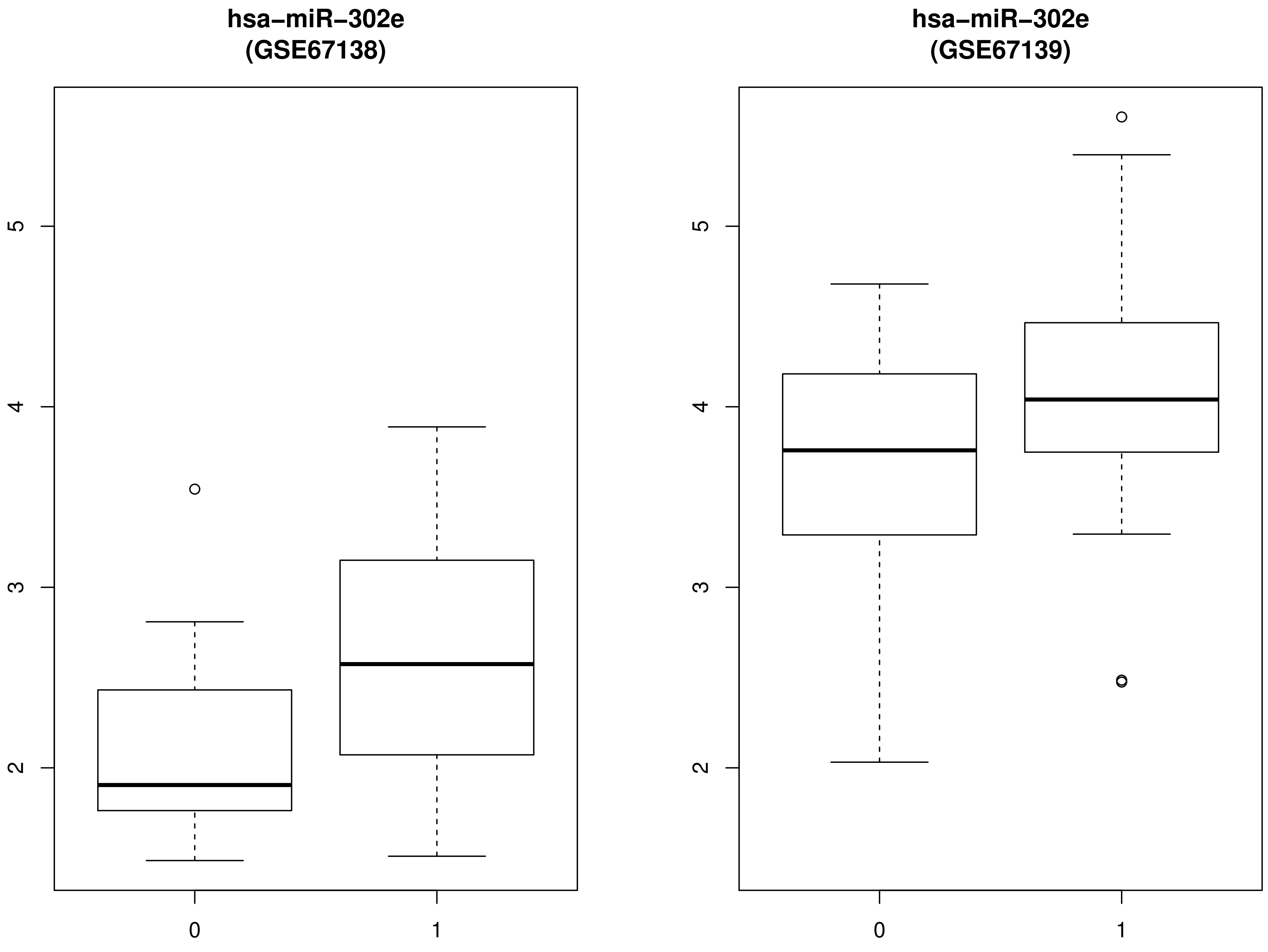
hsa−miR−302e
(GSE67138)
hsa−miR−302e
(GSE67139)
2
3
4
5
0
1
2
3
4
5
0
1

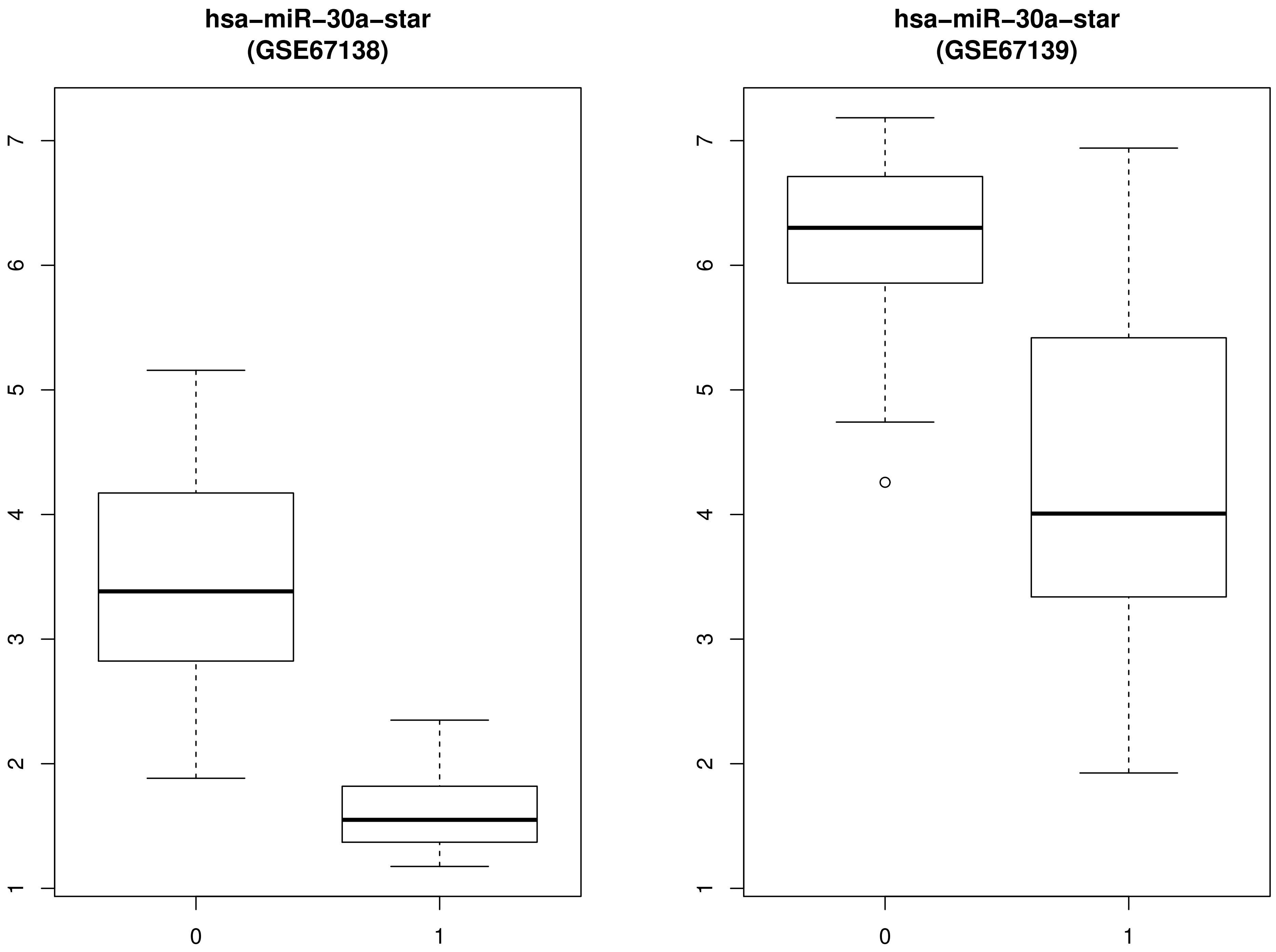
hsa−miR−30a−star
(GSE67138)
hsa−miR−30a−star
(GSE67139)
1
2
3
4
5
6
7
0
1

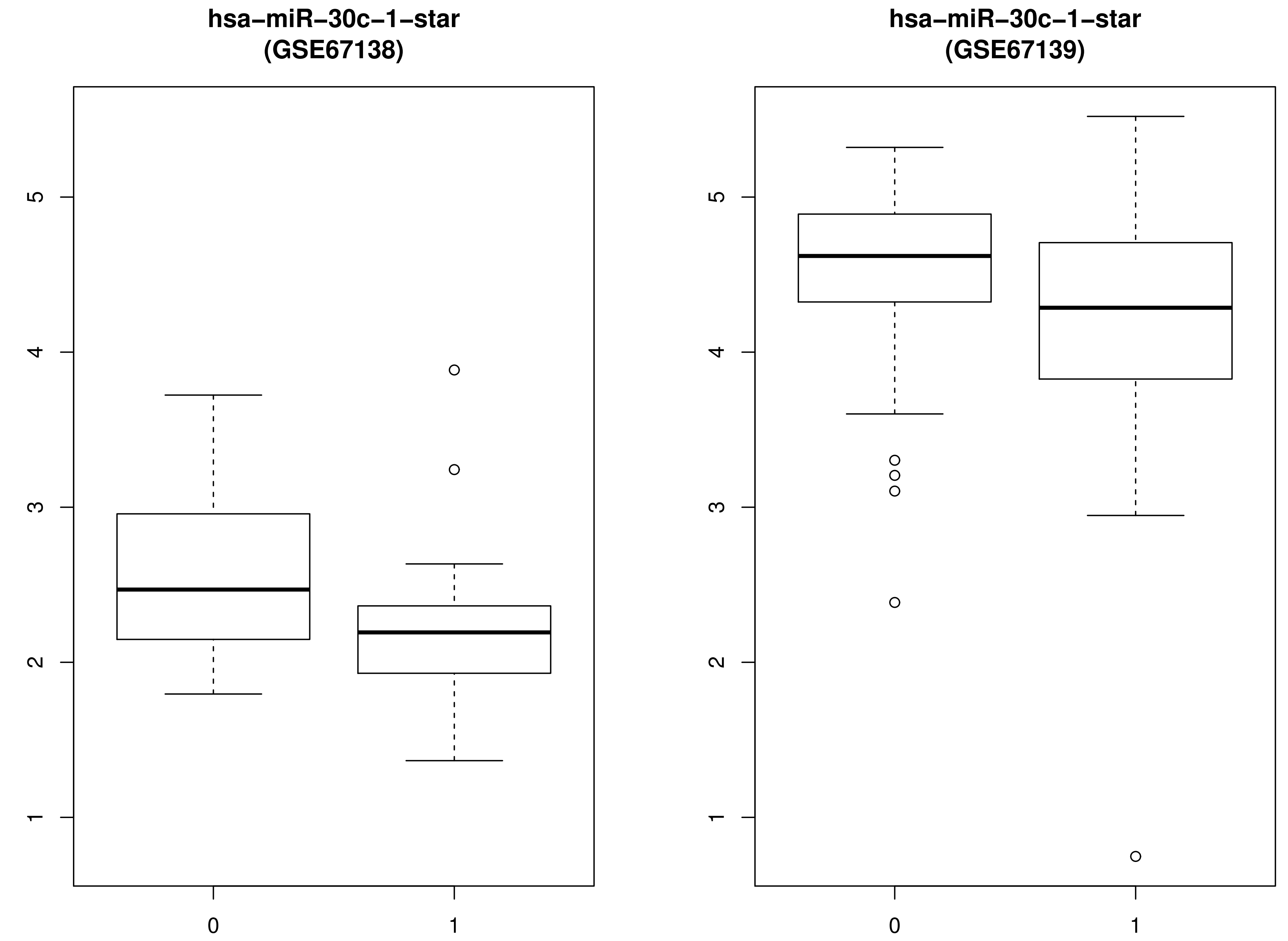
hsa−miR−30c−1−star
(GSE67138)
hsa−miR−30c−1−star
(GSE67139)
1
2
3
4
5
0
1

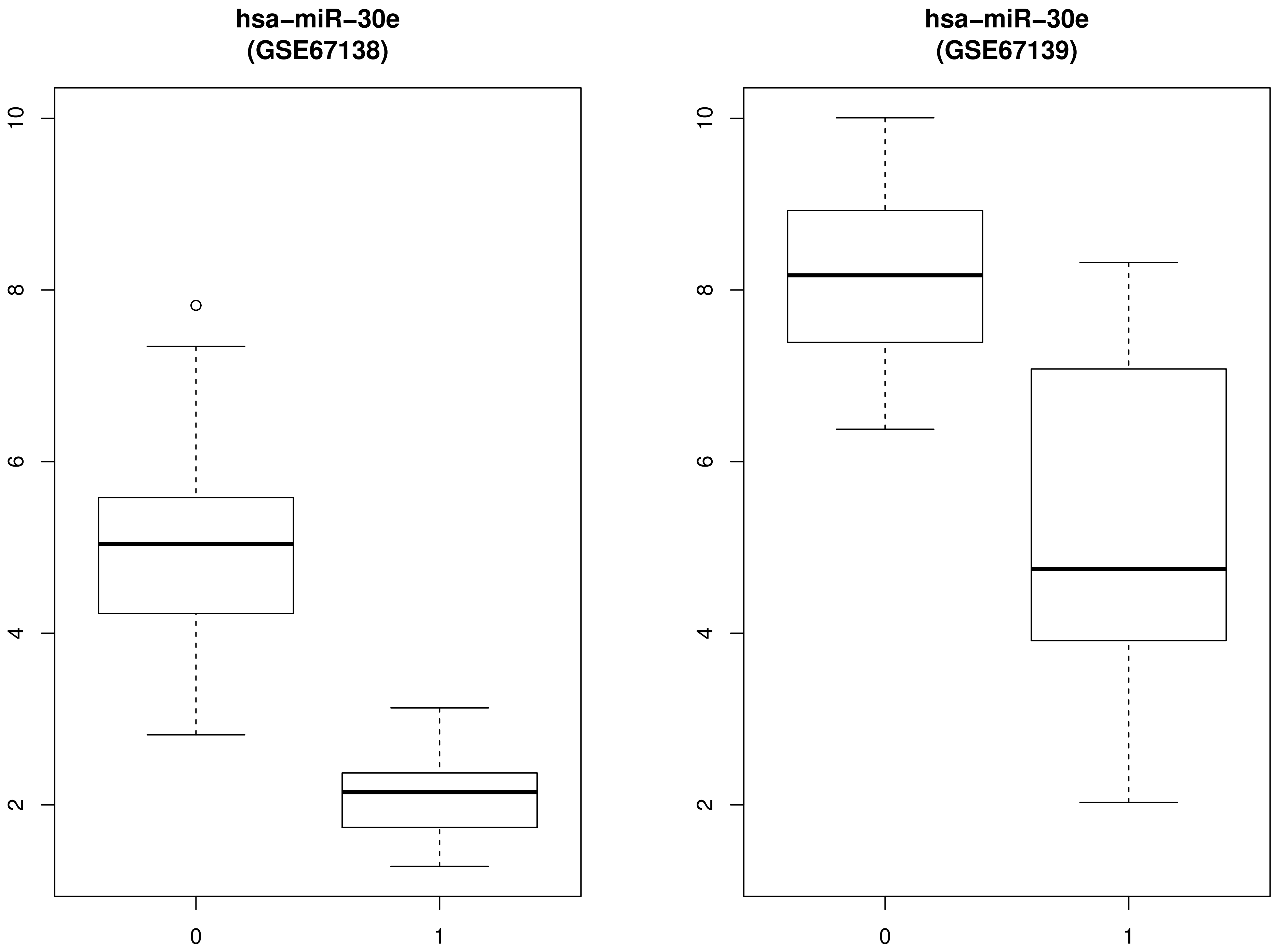

hsa−miR−30e
(GSE67138)
hsa−miR−30e
(GSE67139)
10
8
6
4
2
0
1

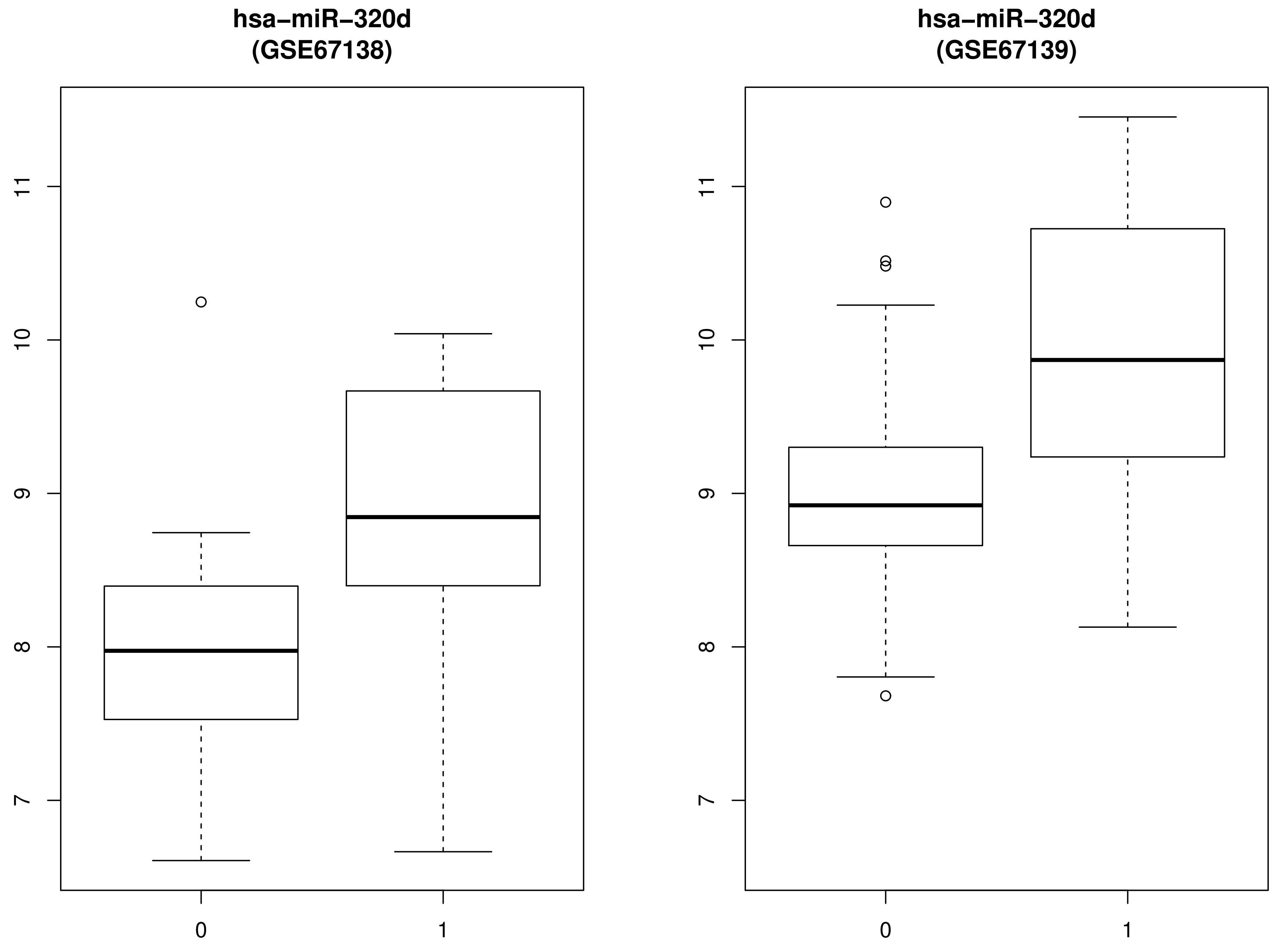
hsa−miR−320d
(GSE67138)
hsa−miR−320d
(GSE67139)
7
8
9
10
11
0
1

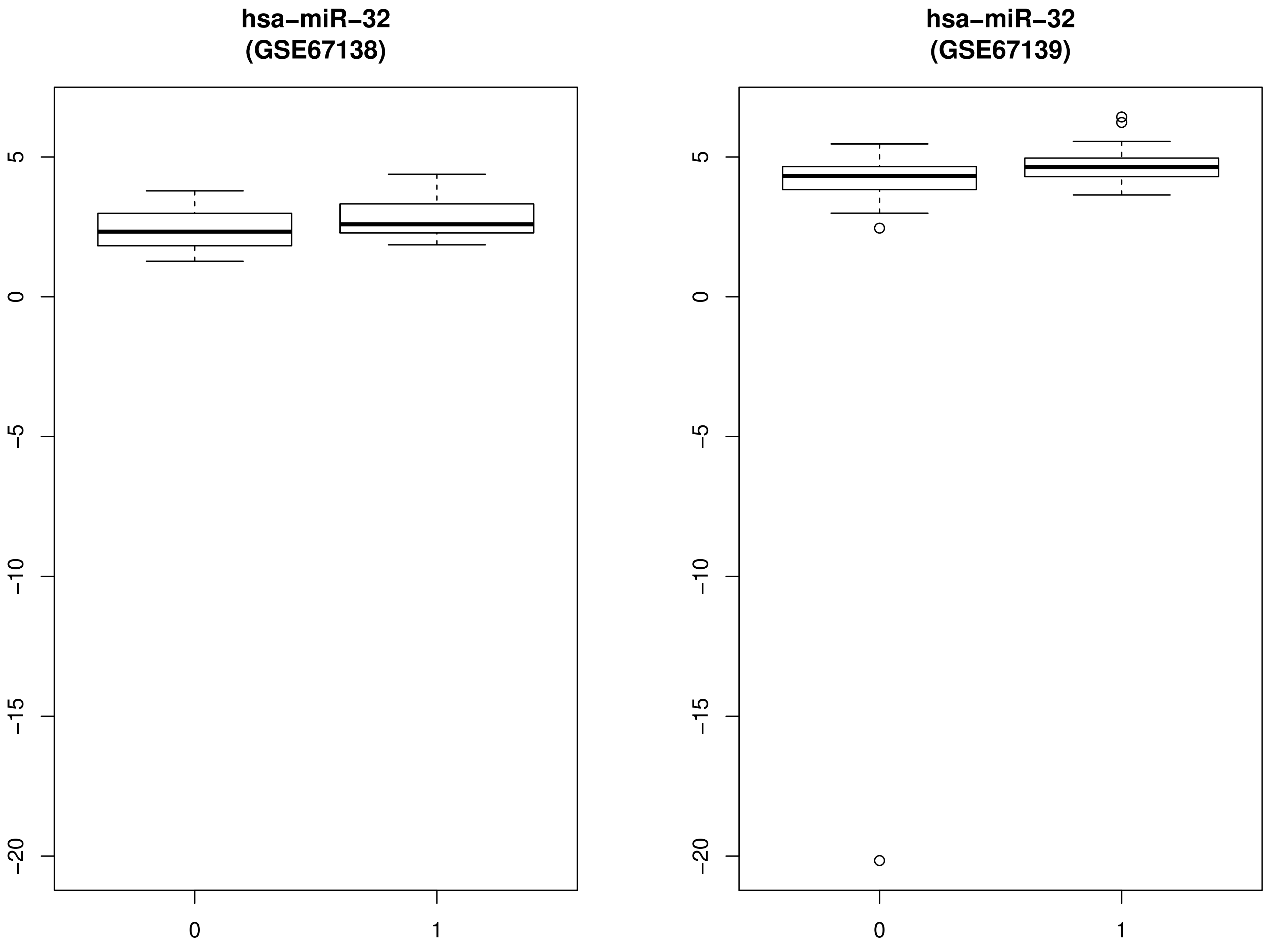
hsa−miR−32
(GSE67138)
hsa−miR−32
(GSE67139)
5
0
−5
−10
−15
−20
0
1

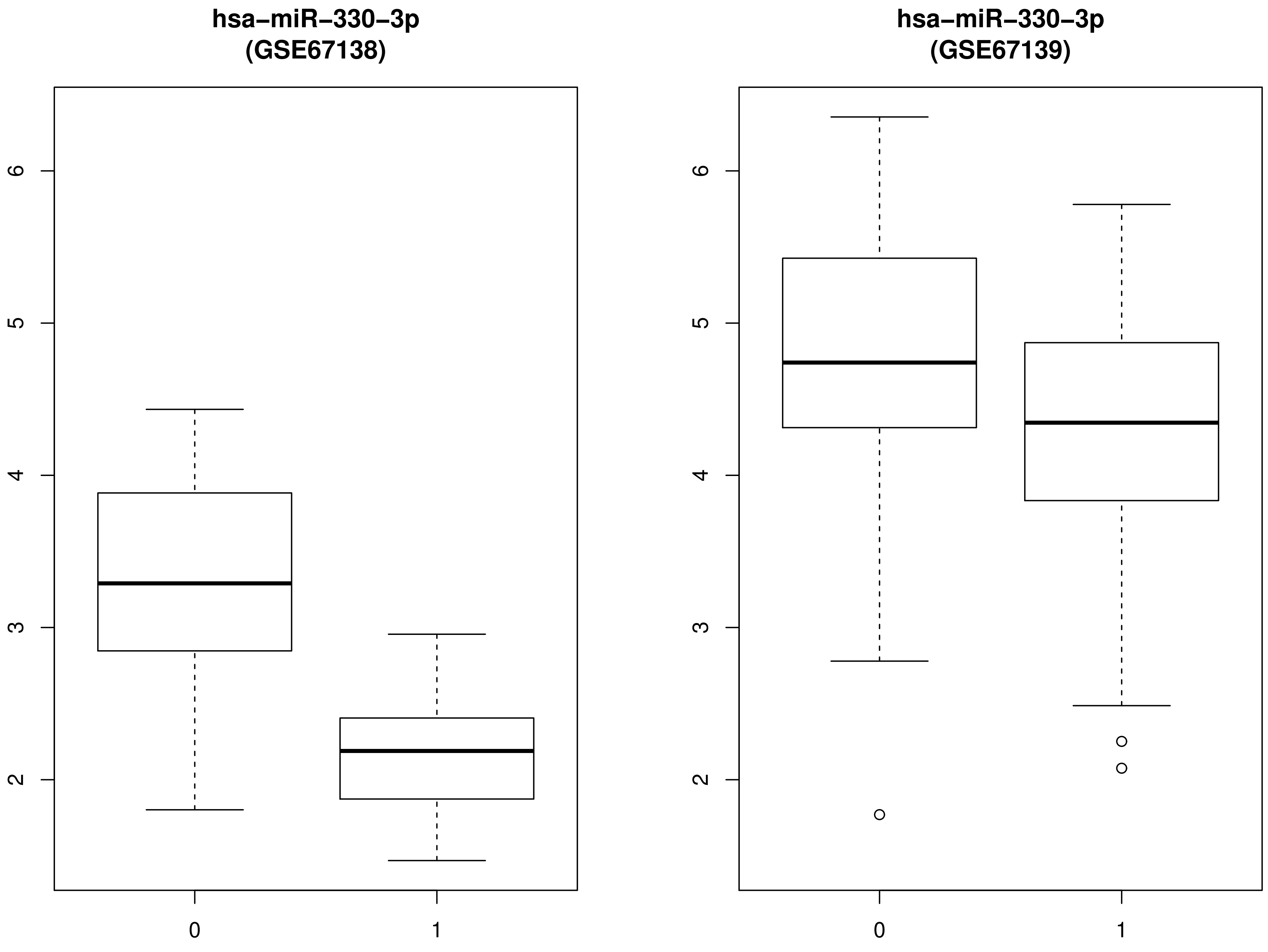
hsa−miR−330−3p
(GSE67138)
hsa−miR−330−3p
(GSE67139)
6
5
4
3
2
0
1
6
5
4
3
2
0
1

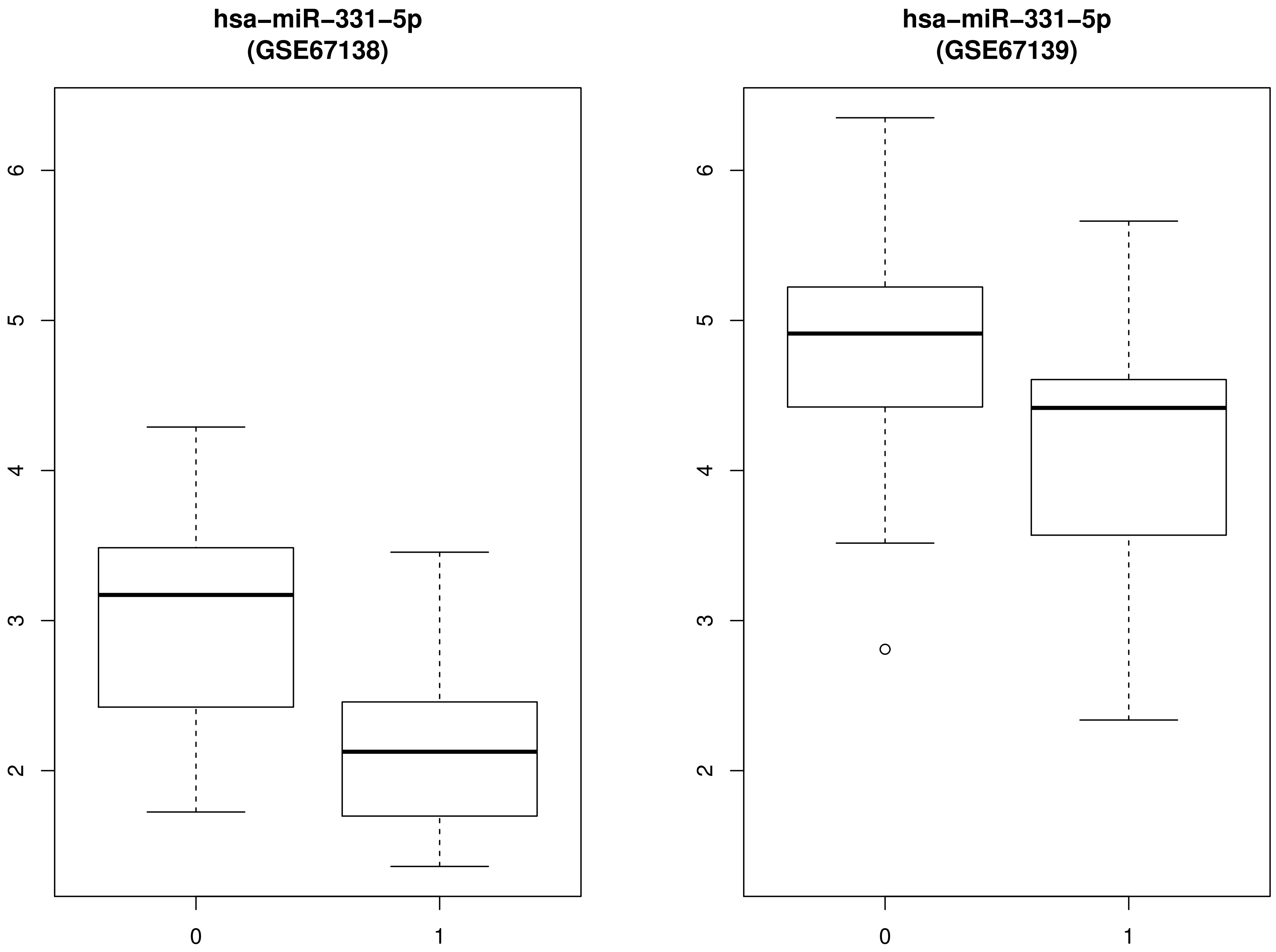
hsa−miR−331−5p
(GSE67138)
hsa−miR−331−5p
(GSE67139)
2
3
4
5
6
0
1

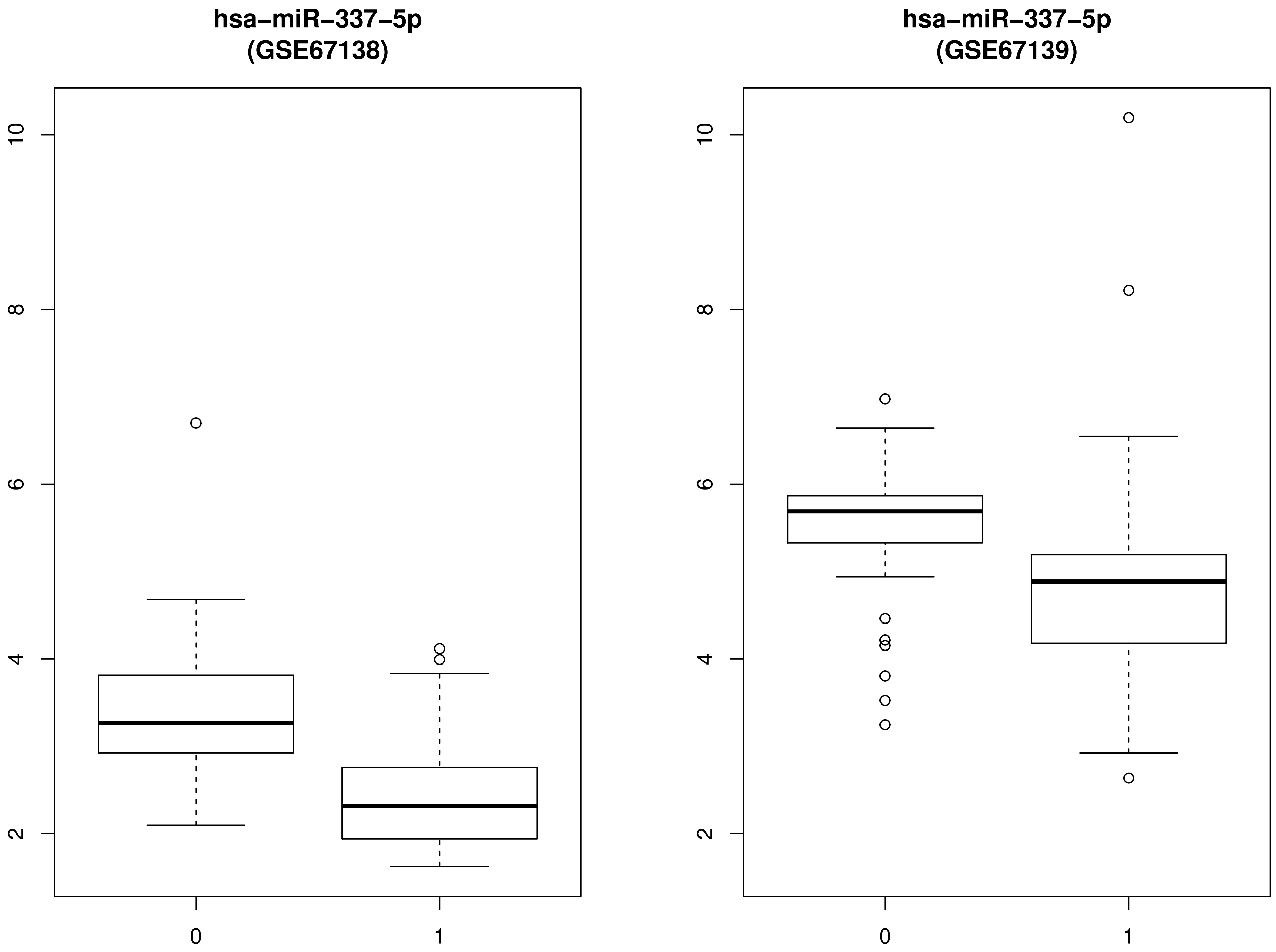
hsa−miR−337−5p
(GSE67138)
hsa−miR−337−5p
(GSE67139)
10
8
6
4
2
0
1

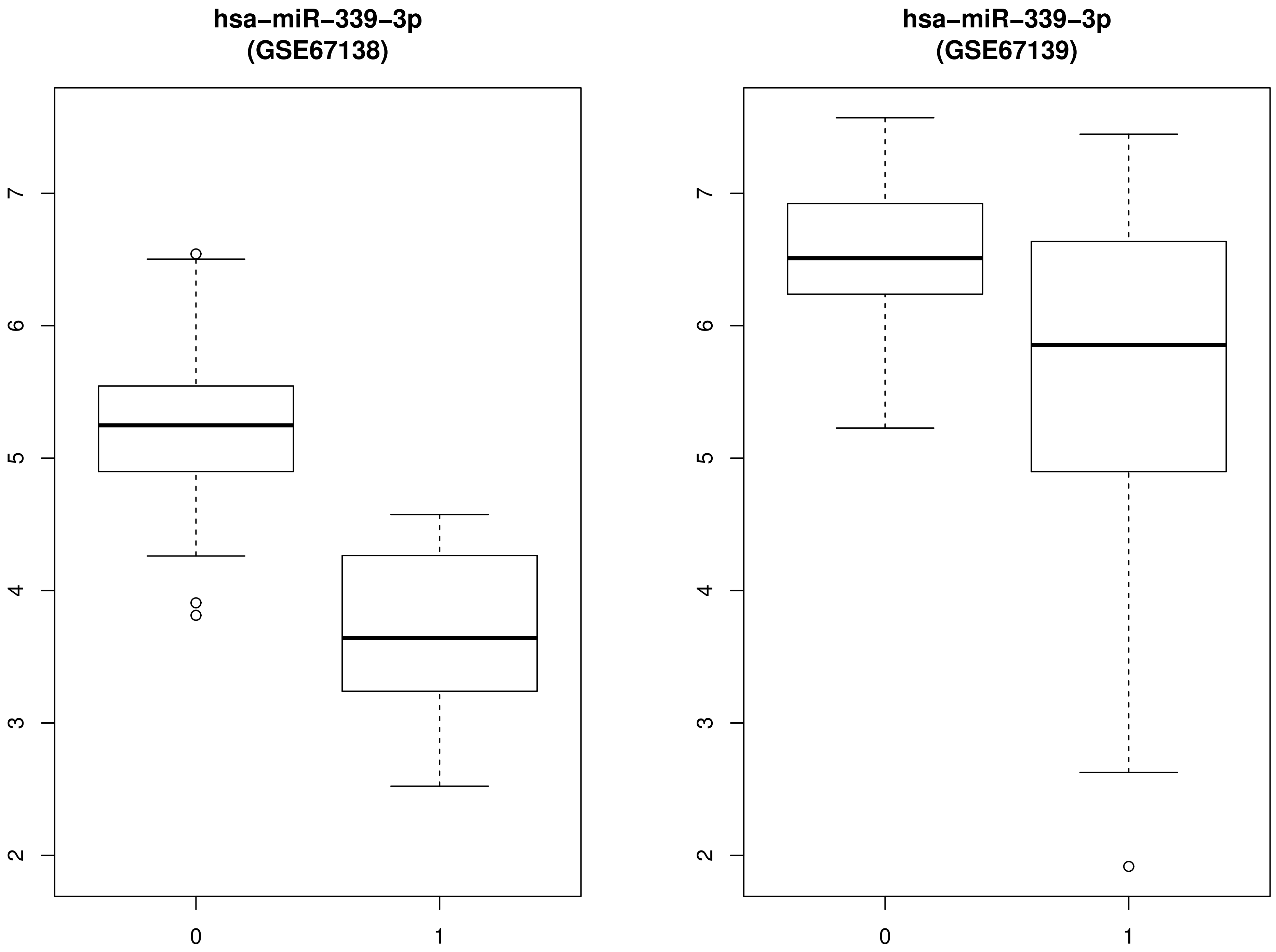
hsa−miR−339−3p
(GSE67138)
hsa−miR−339−3p
(GSE67139)
2
3
4
5
6
7
0
1

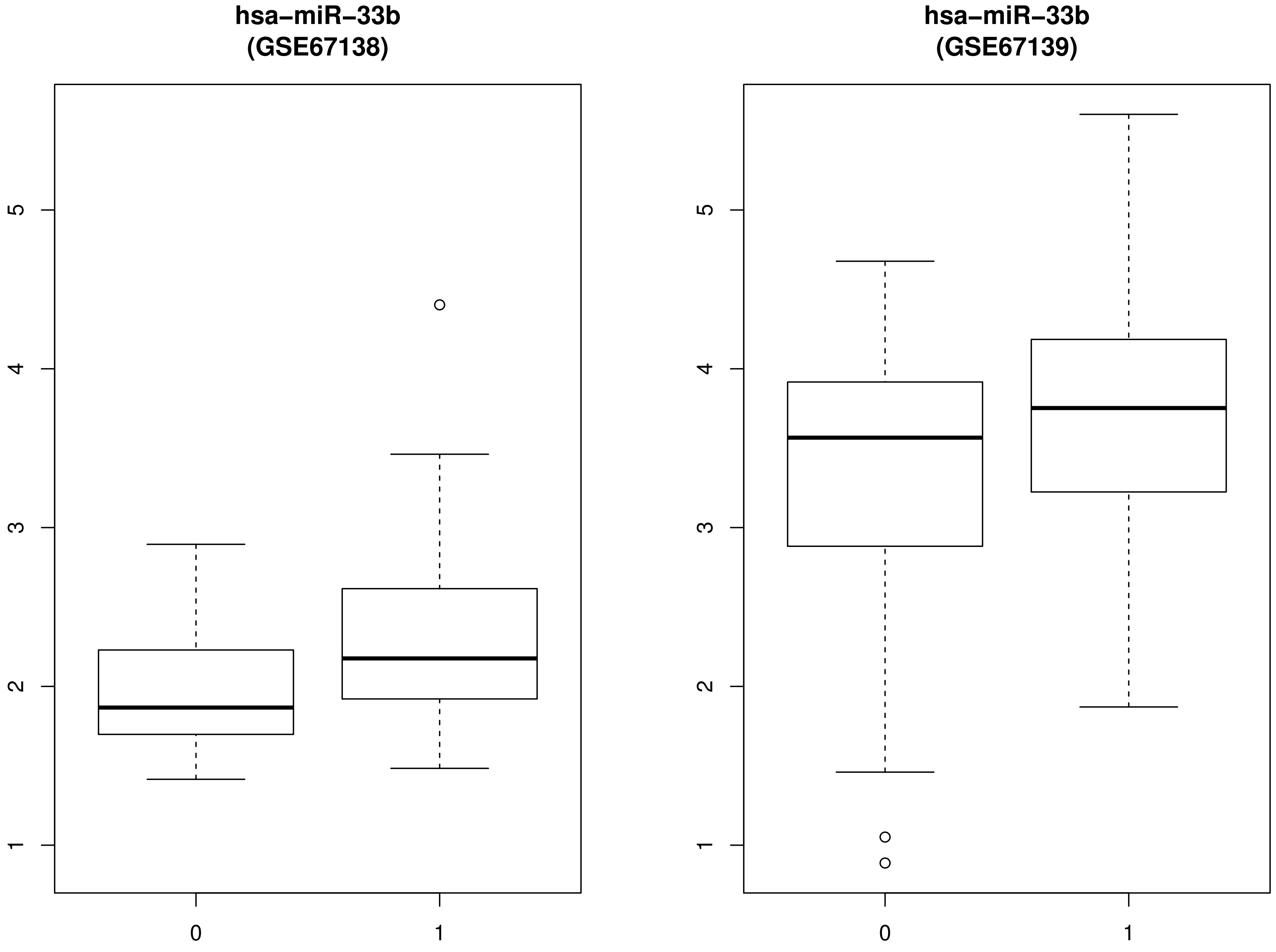
hsa−miR−33b
(GSE67138)
hsa−miR−33b
(GSE67139)
1
2
3
4
5
0
1

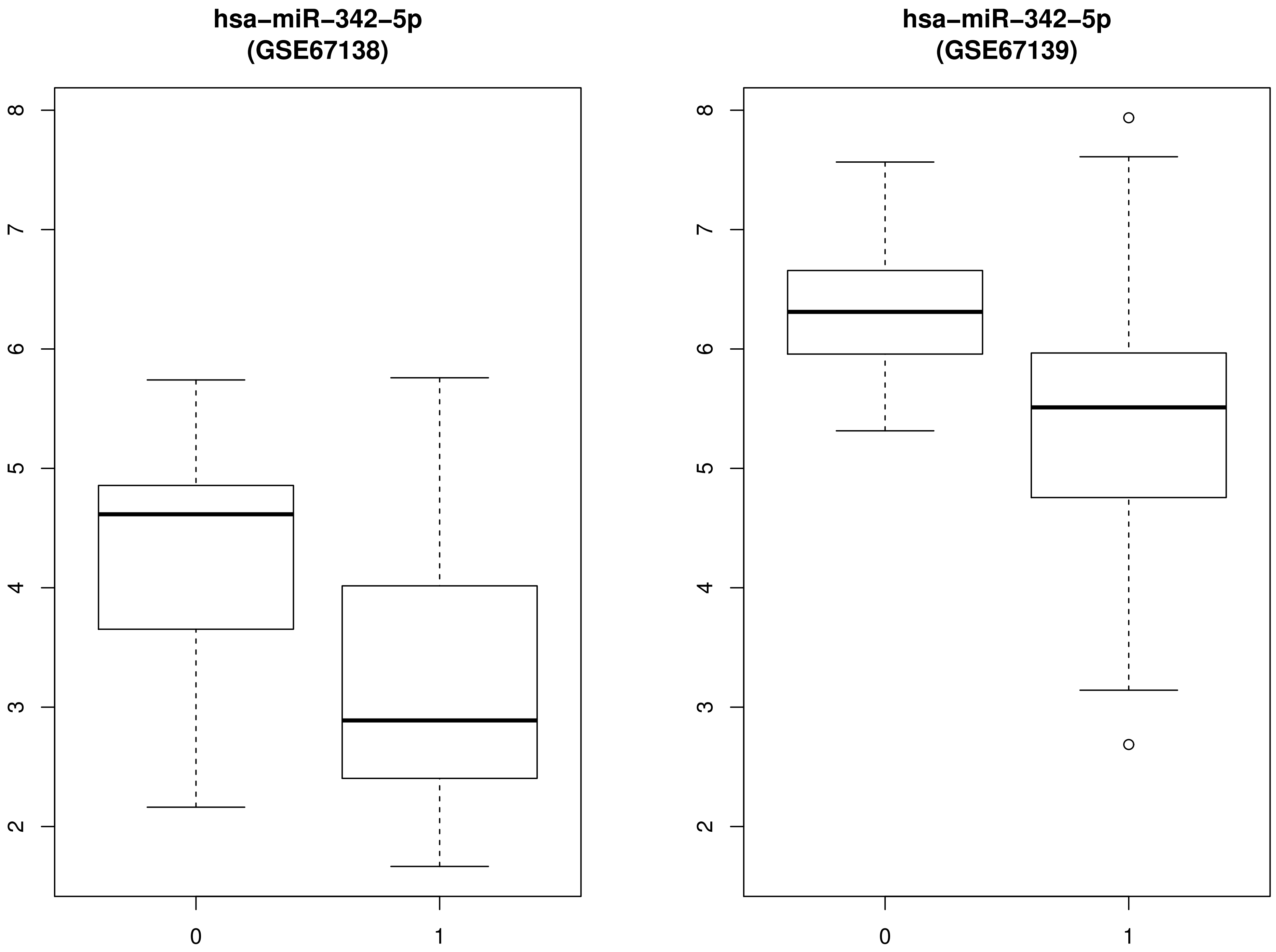
hsa−miR−342−5p
(GSE67138)
hsa−miR−342−5p
(GSE67139)
2
3
4
5
6
7
8
0
1

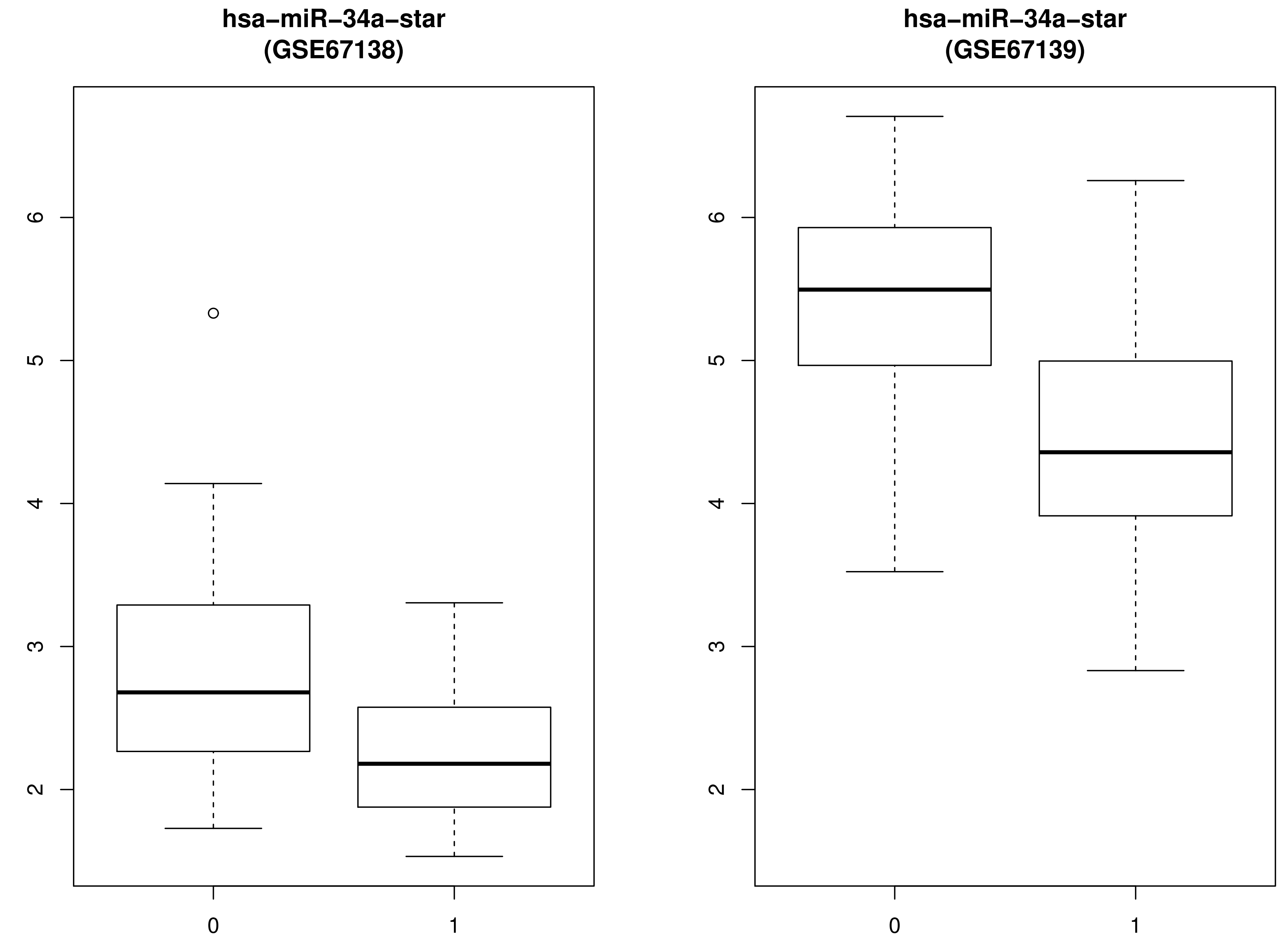
hsa−miR−34a−star
(GSE67138)
hsa−miR−34a−star
(GSE67139)
2
3
4
5
6
0
1

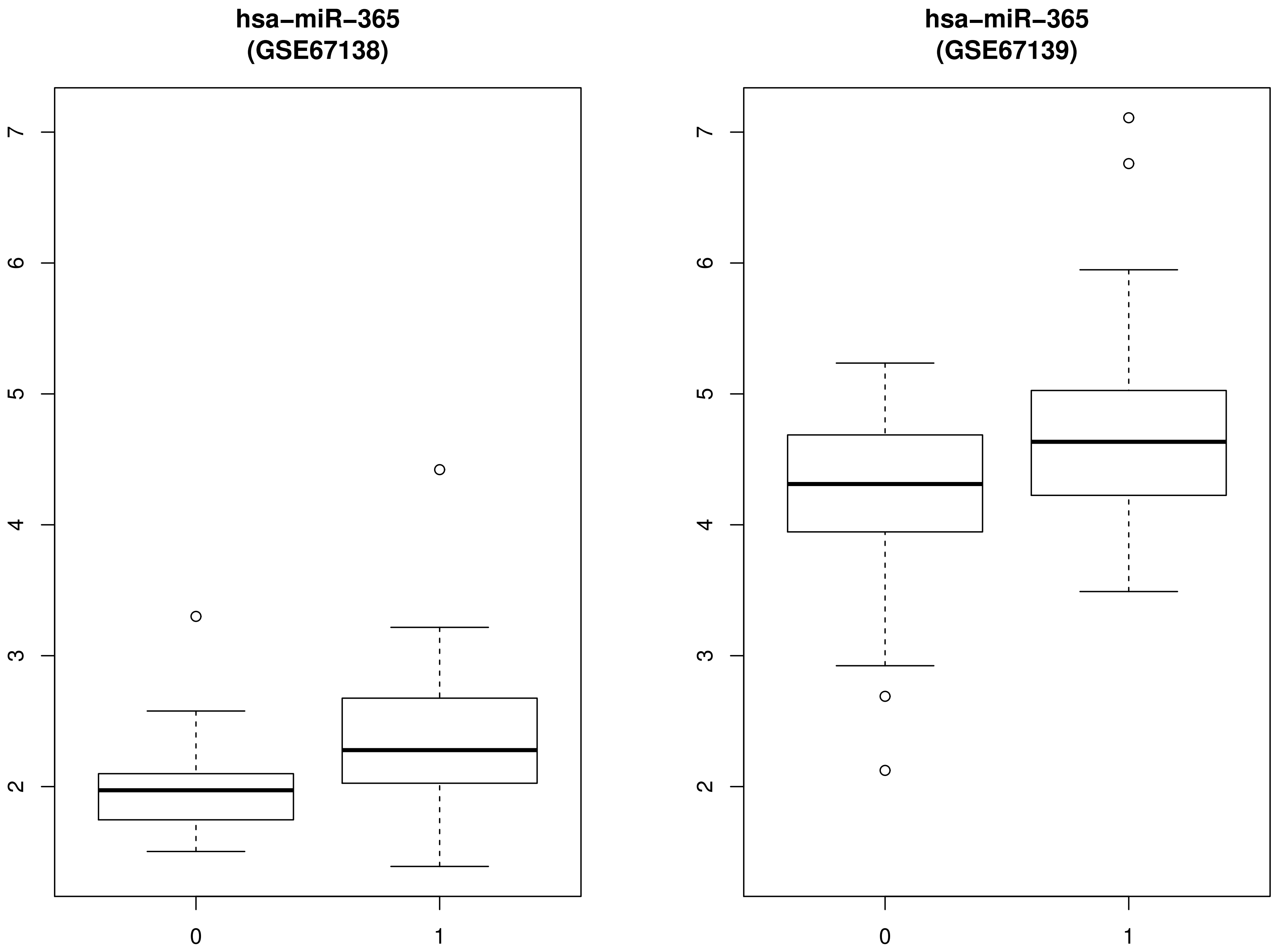
hsa−miR−365
(GSE67138)
hsa−miR−365
(GSE67139)
2
3
4
5
6
7
0
1

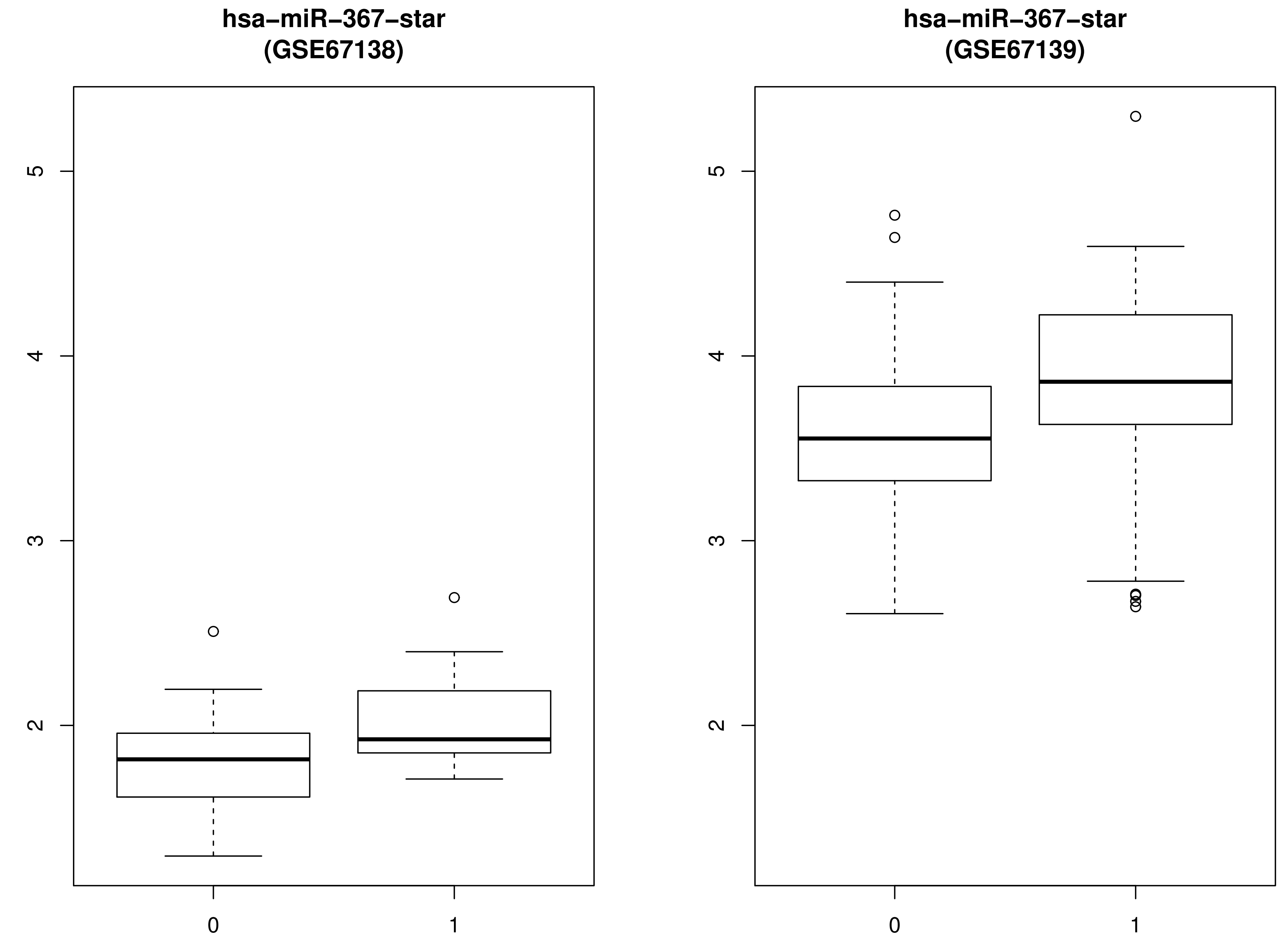
hsa−miR−367−star
(GSE67138)
hsa−miR−367−star
(GSE67139)
2
3
4
5
0
1

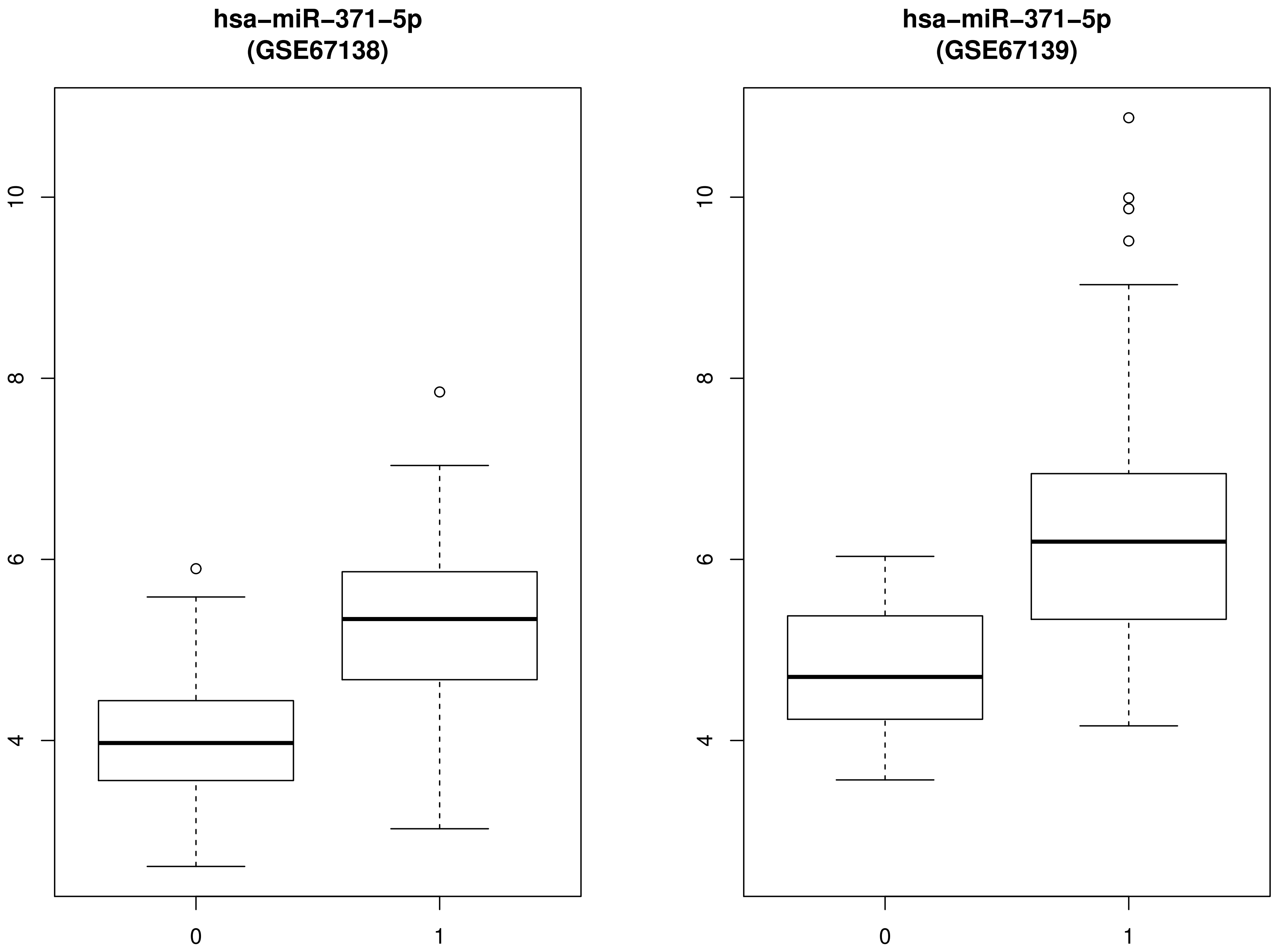
hsa−miR−371−5p
(GSE67138)
hsa−miR−371−5p
(GSE67139)
4
6
8
10
0
1

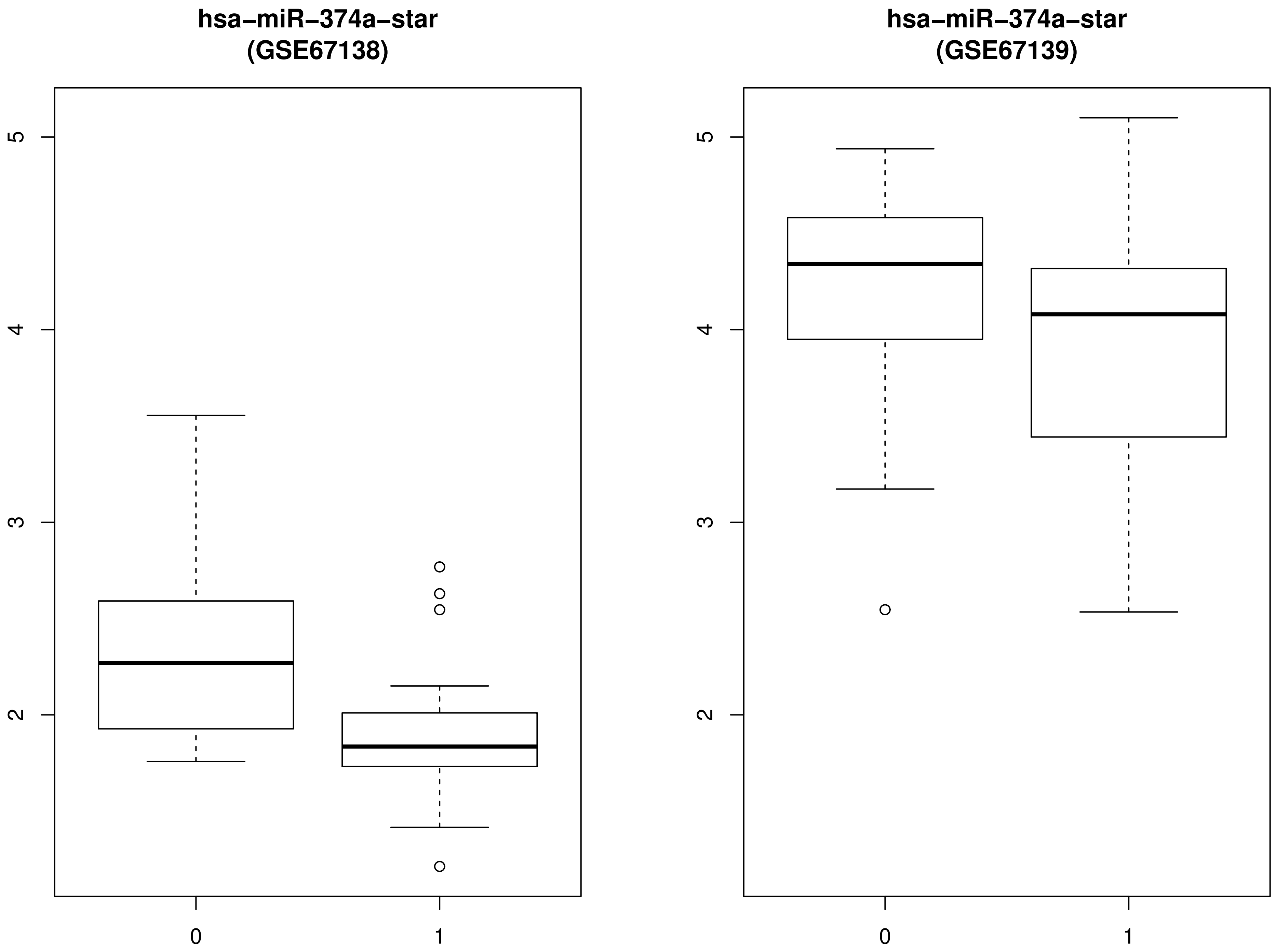
hsa−miR−374a−star
(GSE67138)
hsa−miR−374a−star
(GSE67139)
2
3
4
5
0
1

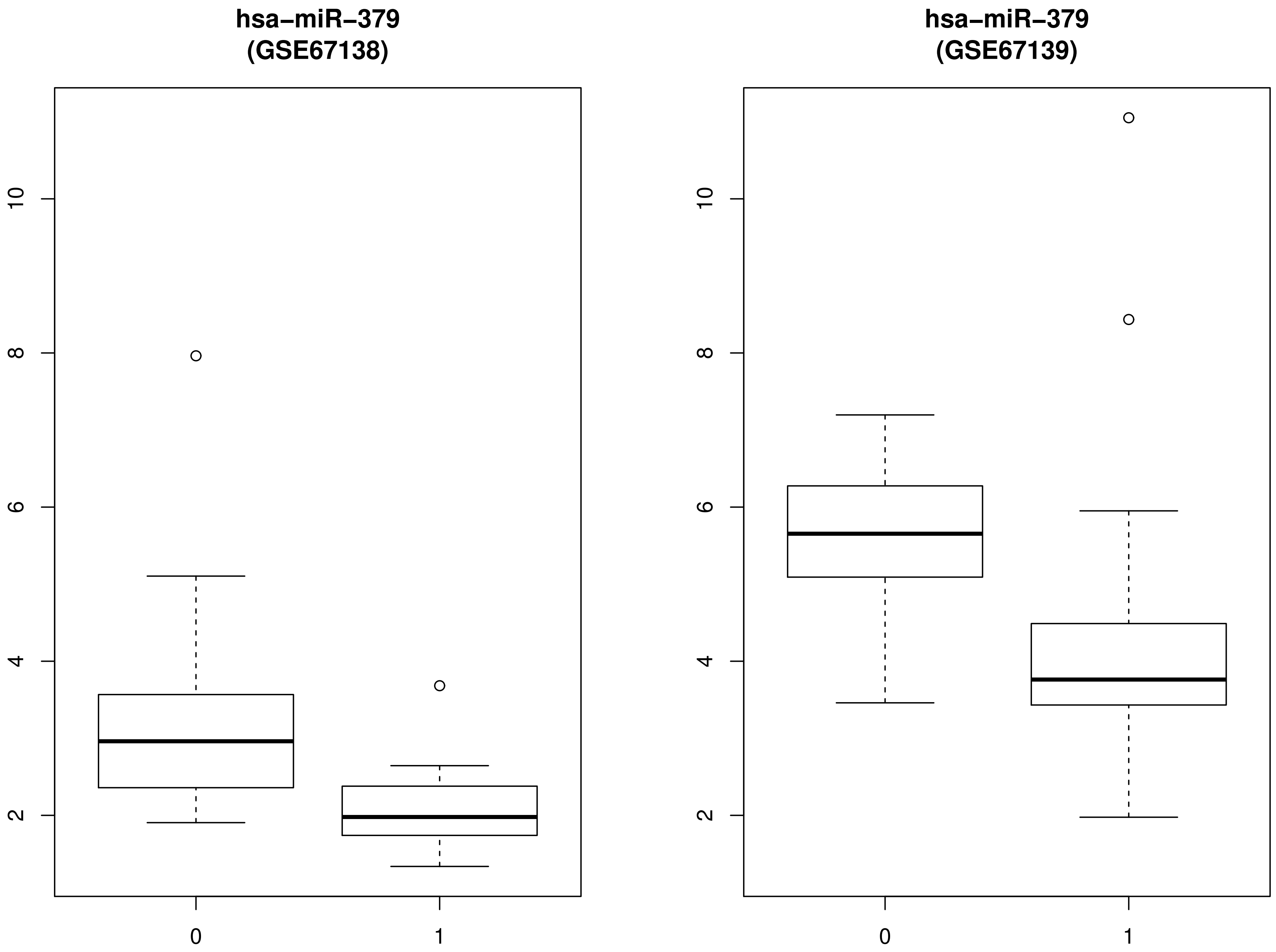
hsa−miR−379
(GSE67138)
hsa−miR−379
(GSE67139)
2
4
6
8
10
0
1

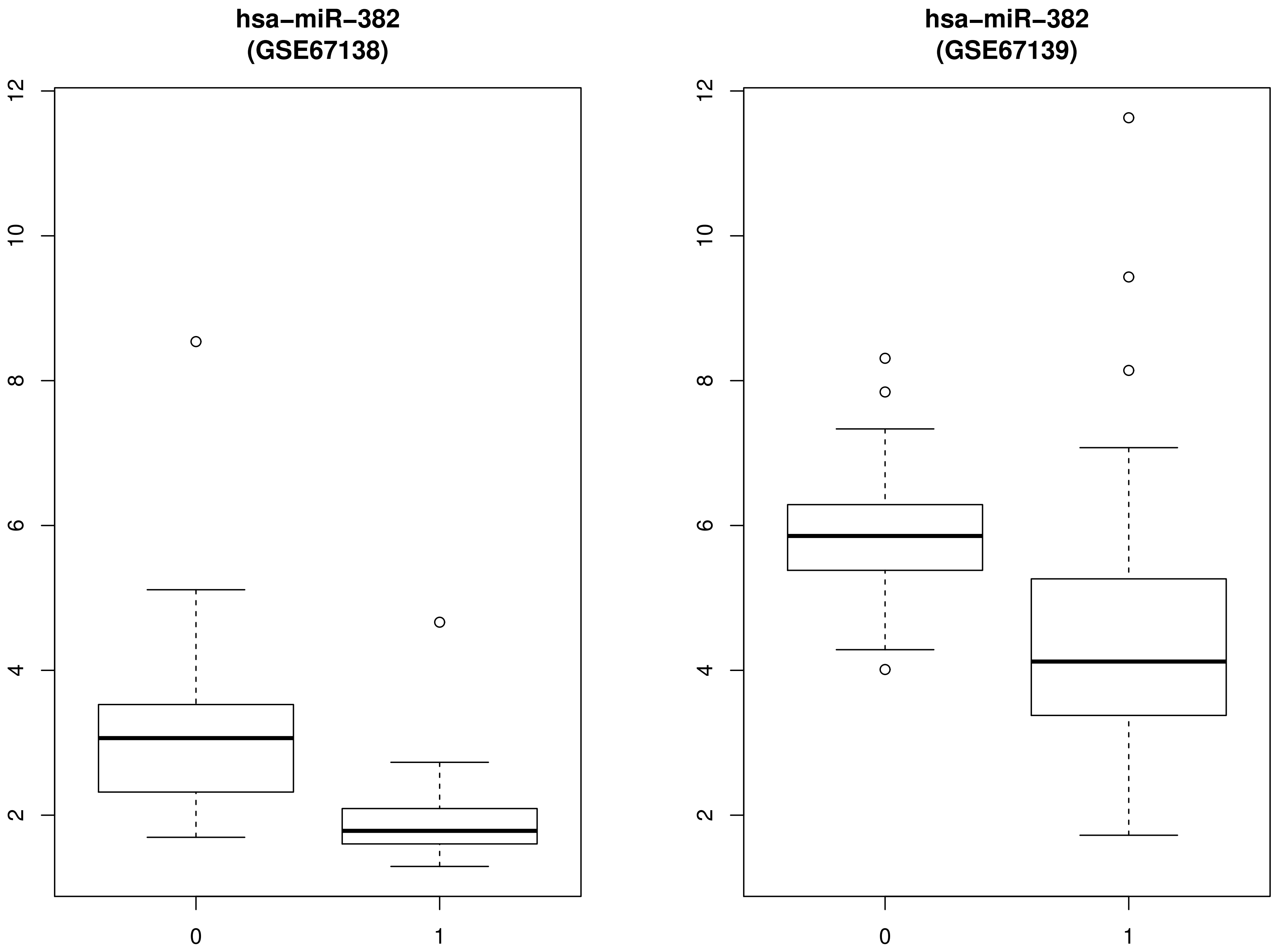

hsa−miR−382
(GSE67138)
hsa−miR−382
(GSE67139)
2
4
6
8
10
12
0
1

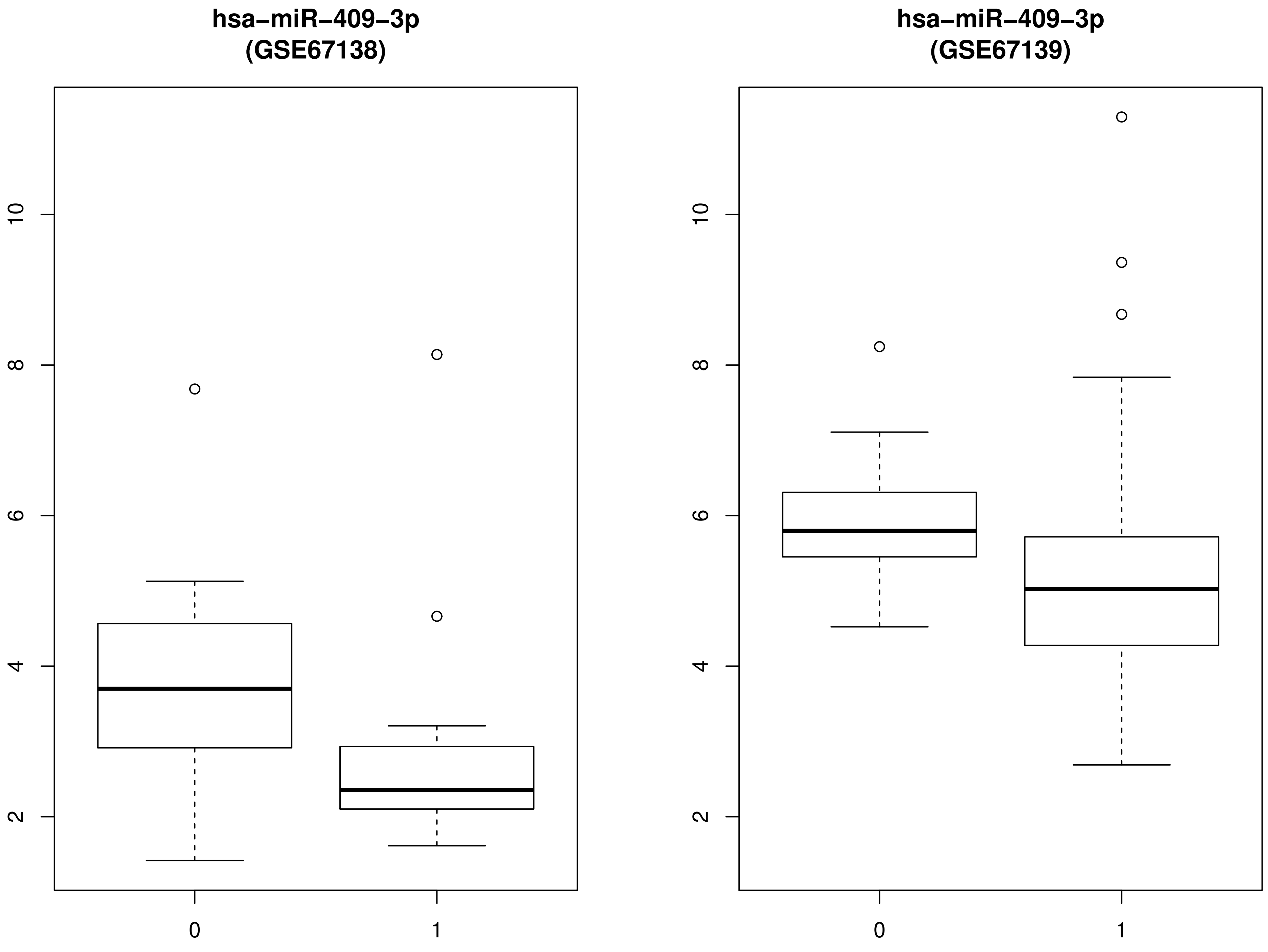

hsa−miR−409−3p
(GSE67138)
hsa−miR−409−3p
(GSE67139)
2
4
6
8
10
0
1

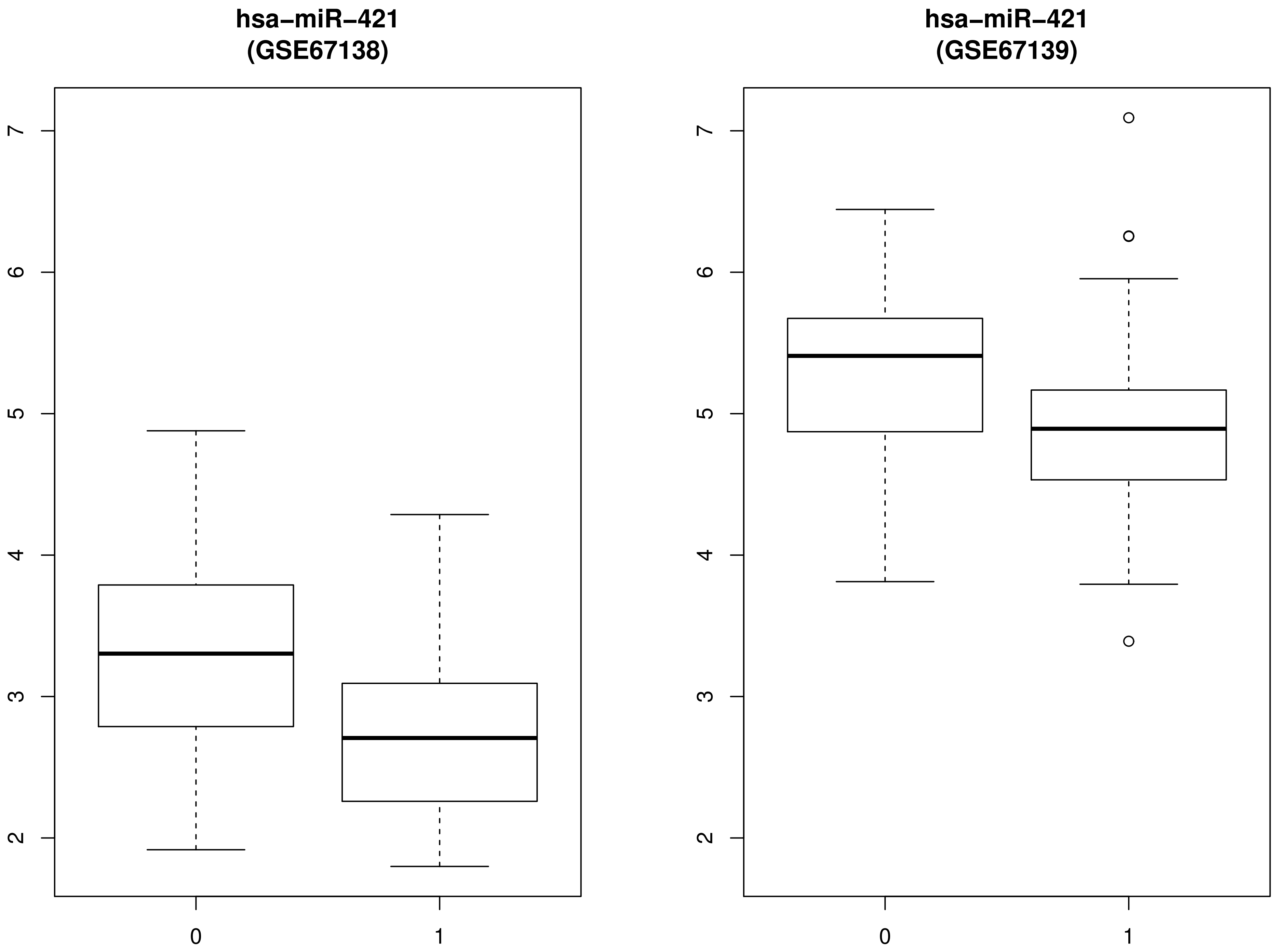
hsa−miR−421
(GSE67138)
hsa−miR−421
(GSE67139)
2
3
4
5
6
7
0
1

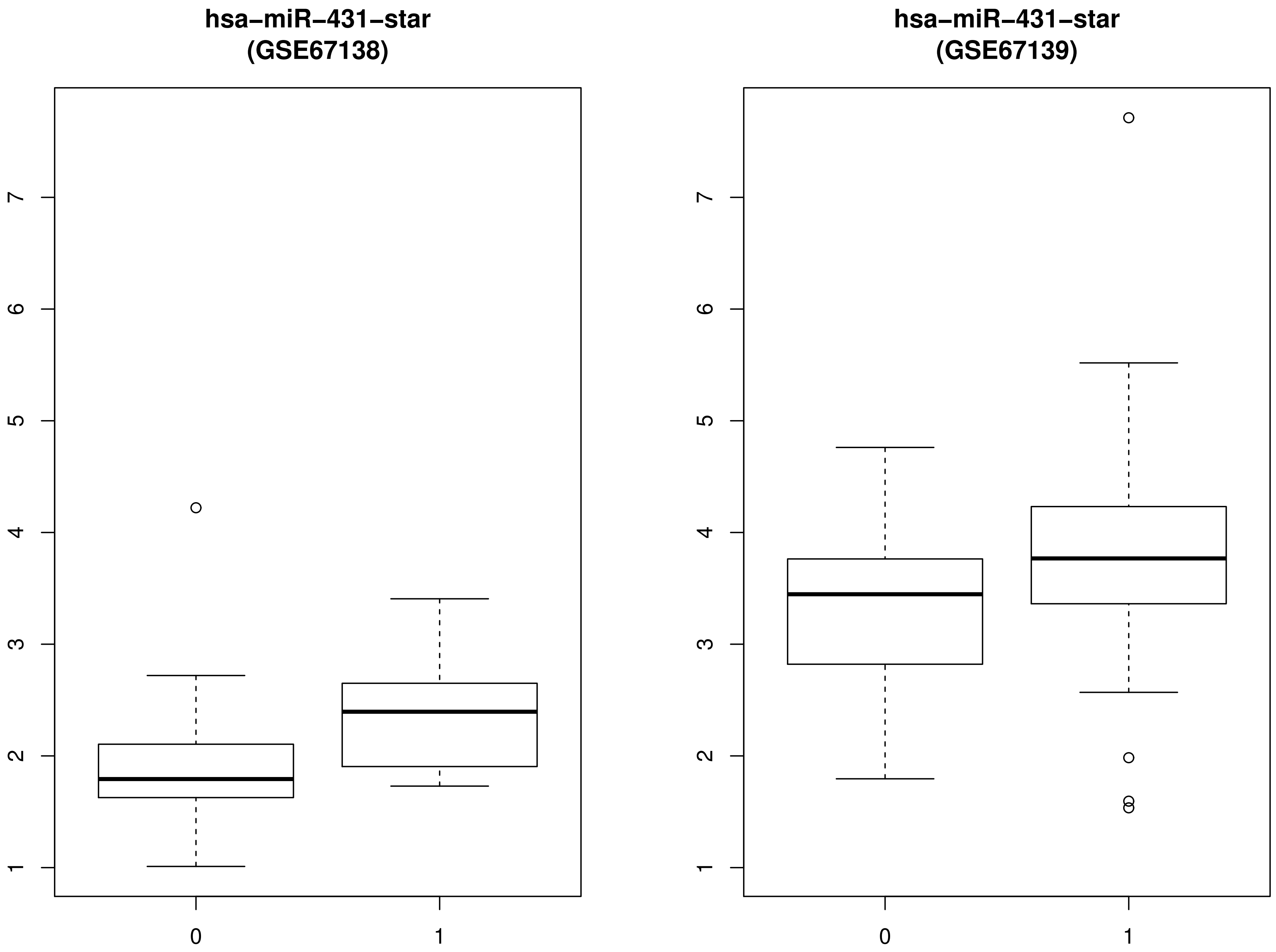
hsa−miR−431−star
(GSE67138)
hsa−miR−431−star
(GSE67139)
1
2
3
4
5
6
7
0
1

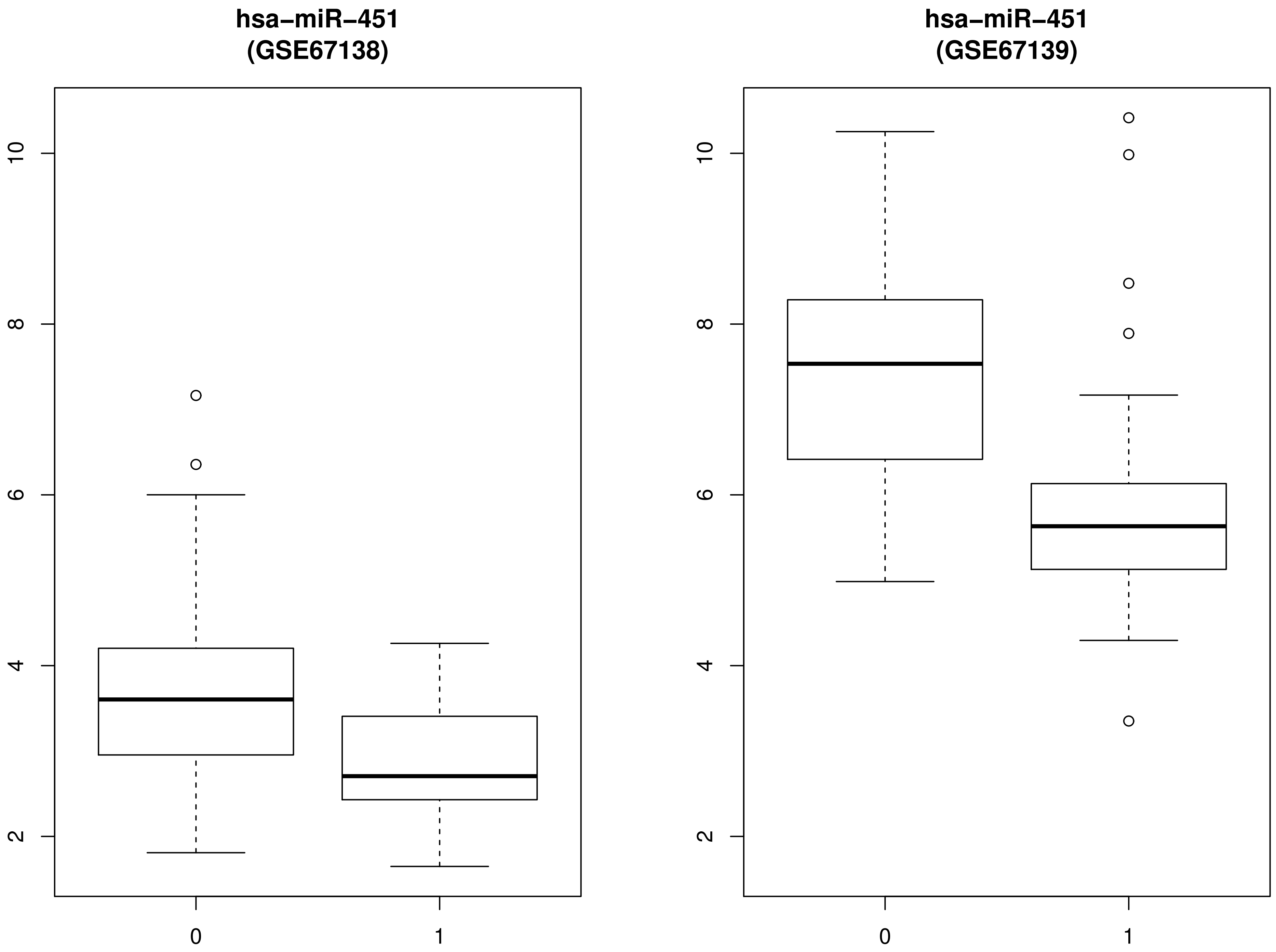
hsa−miR−451
(GSE67138)
hsa−miR−451
(GSE67139)
2
4
6
8
10
0
1

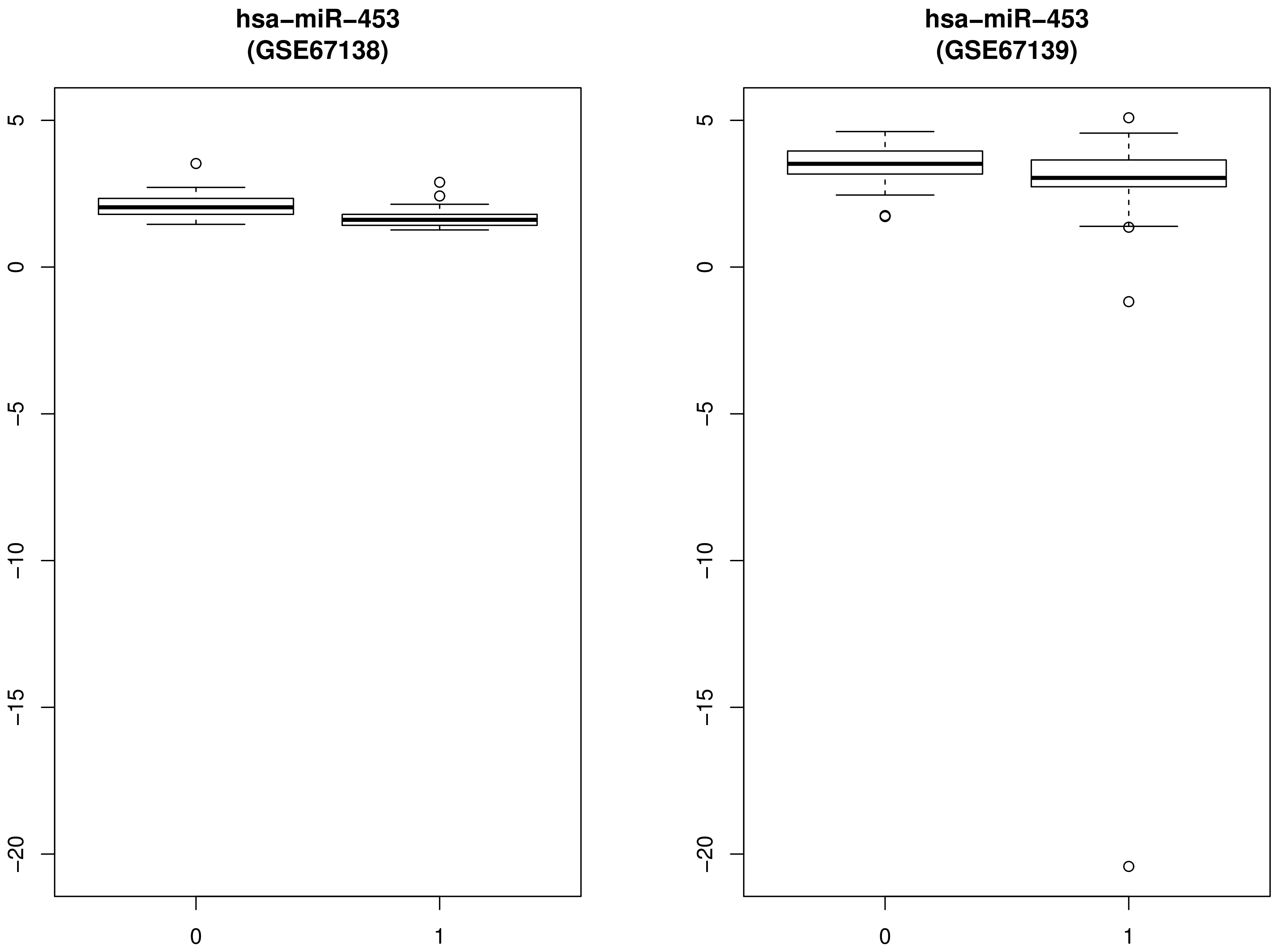

hsa−miR−453
(GSE67138)
hsa−miR−453
(GSE67139)
5
0
−5
−10
−15
−20
0
1

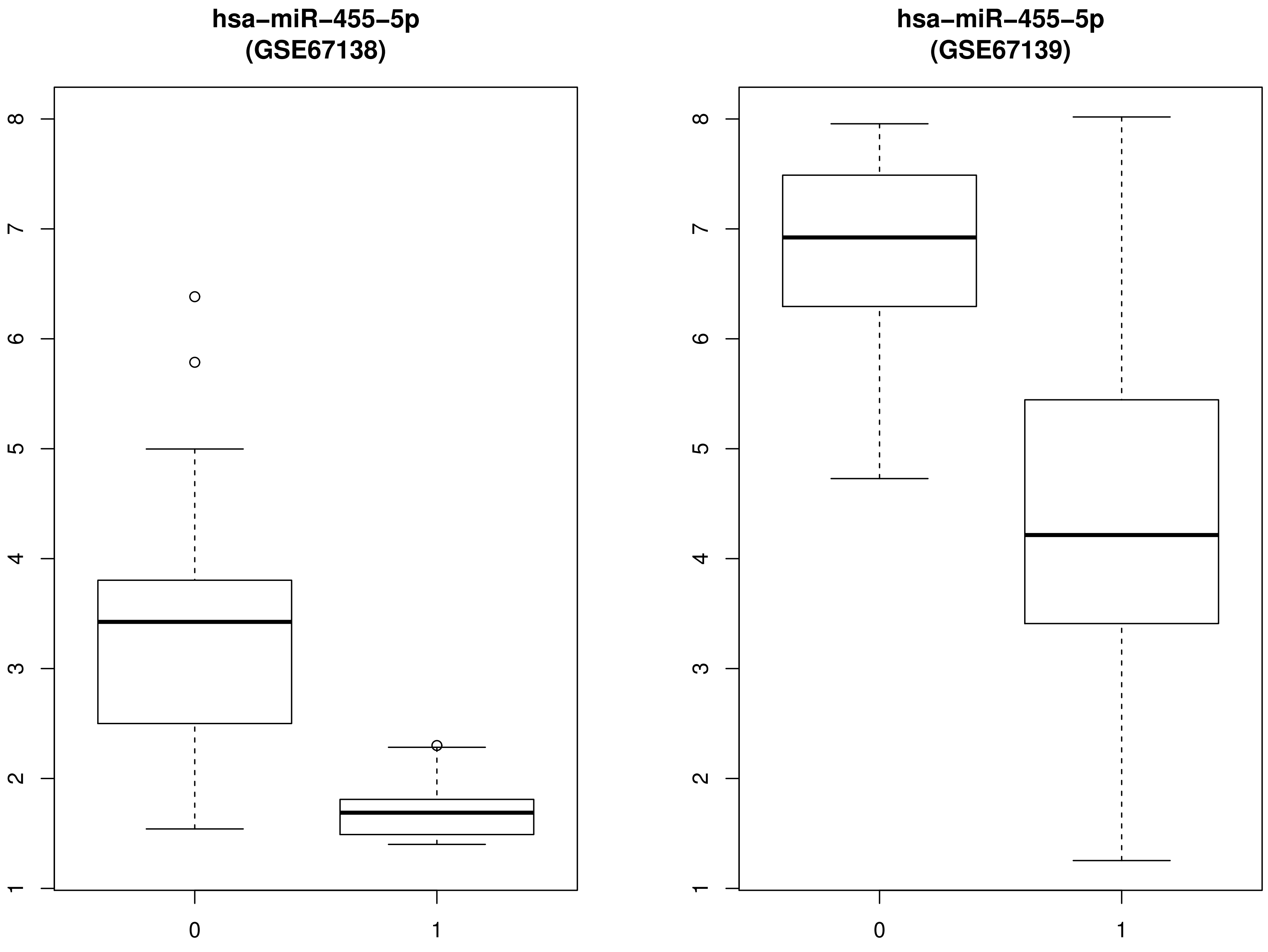
hsa−miR−455−5p
(GSE67138)
hsa−miR−455−5p
(GSE67139)
1
2
3
4
5
6
7
8
0
1

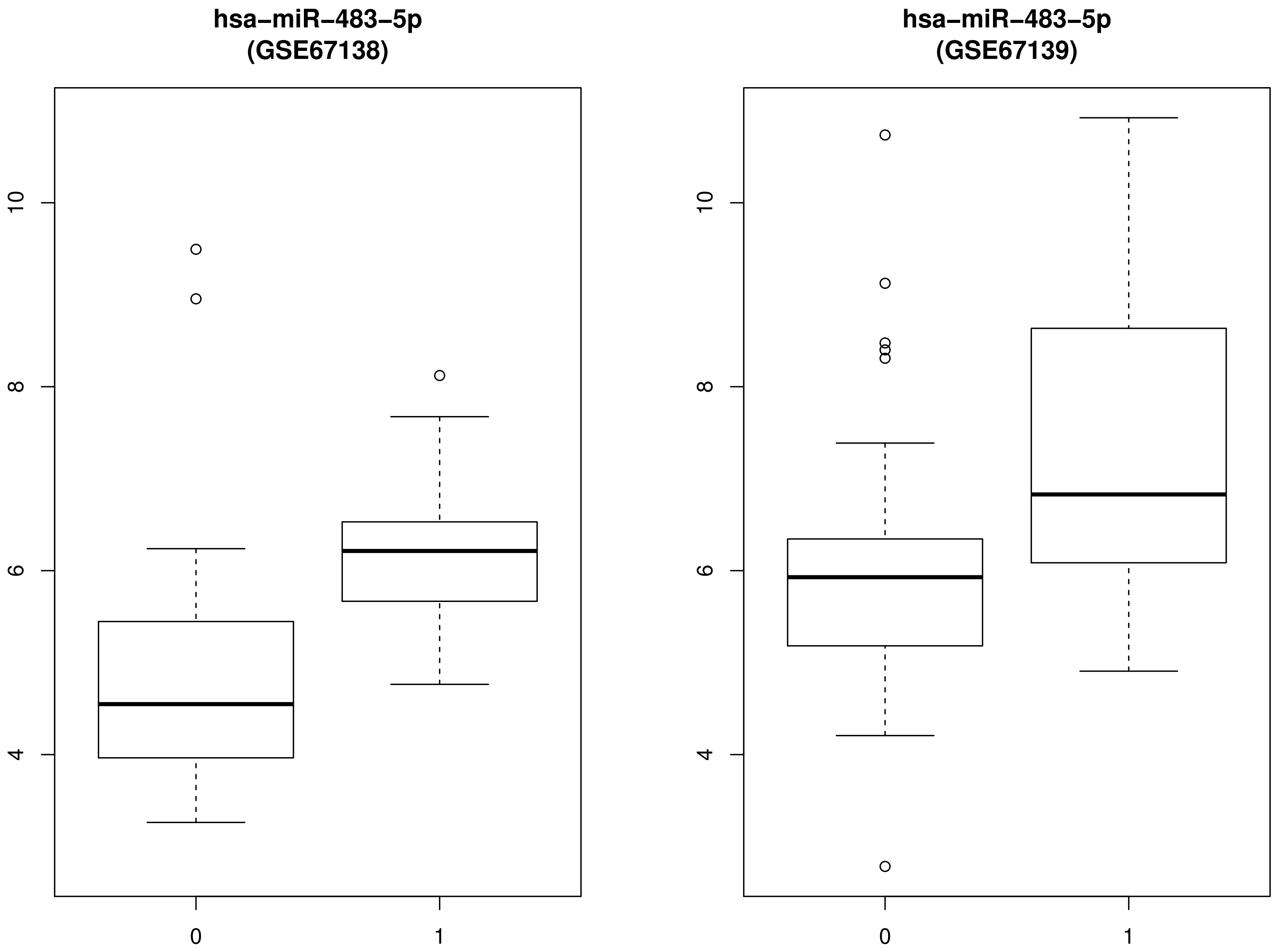
hsa−miR−483−5p
(GSE67138)
hsa−miR−483−5p
(GSE67139)
10
8
6
4
0
1

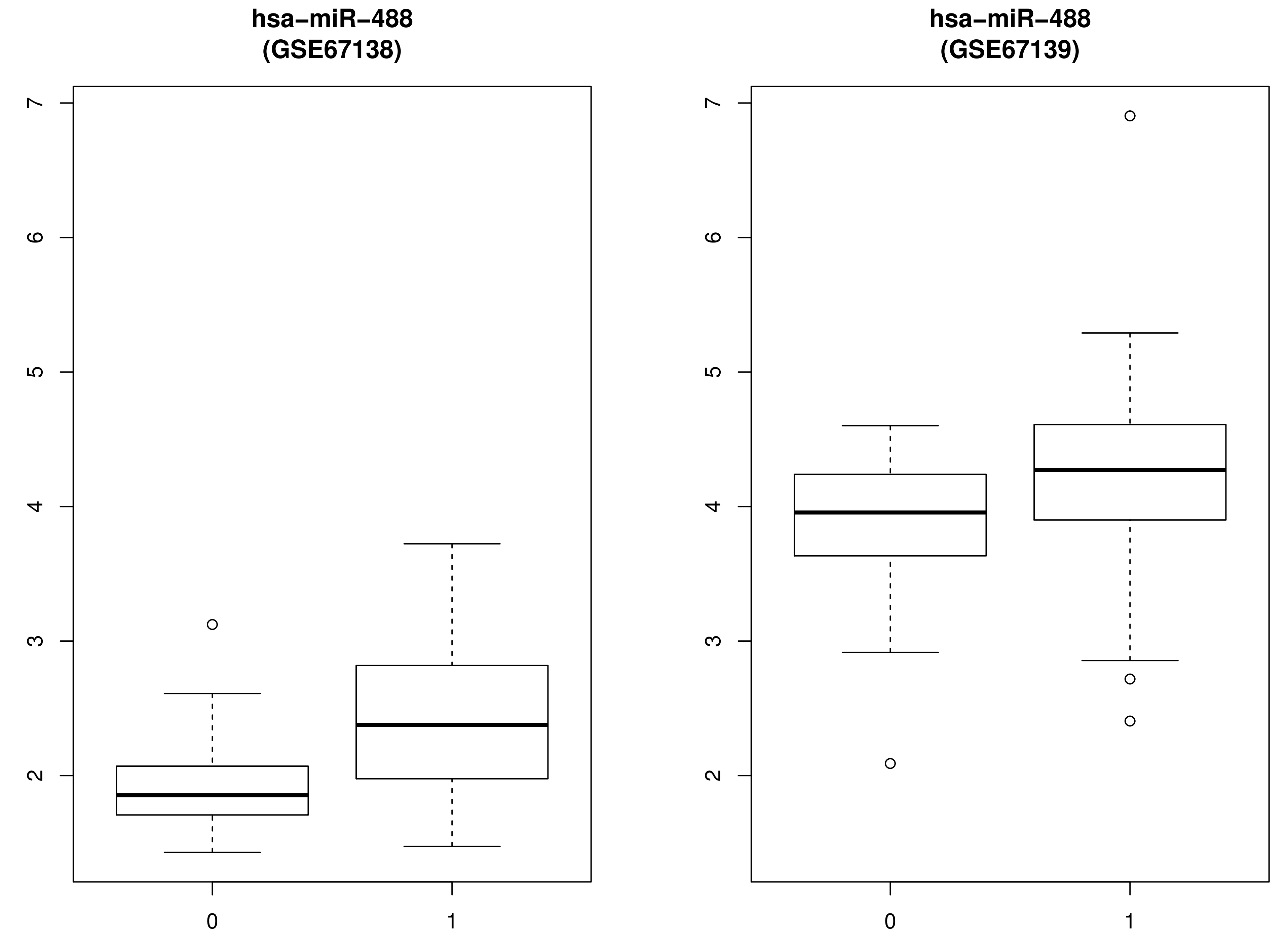
hsa−miR−488
(GSE67138)
hsa−miR−488
(GSE67139)
2
3
4
5
6
7
0
1
2
3
4
5
6
7
0
1

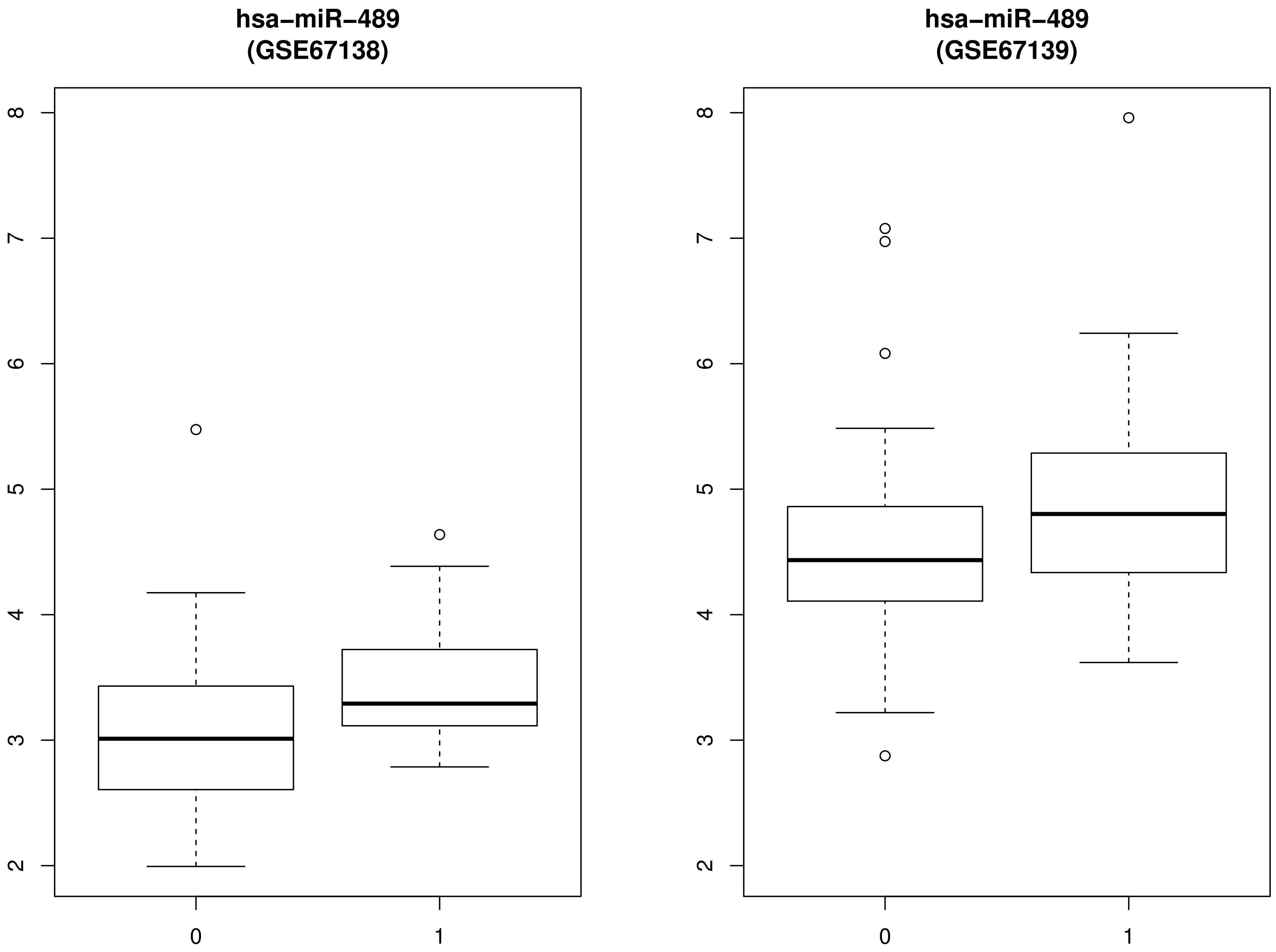
hsa−miR−489
(GSE67138)
hsa−miR−489
(GSE67139)
2
3
4
5
6
7
8
0
1

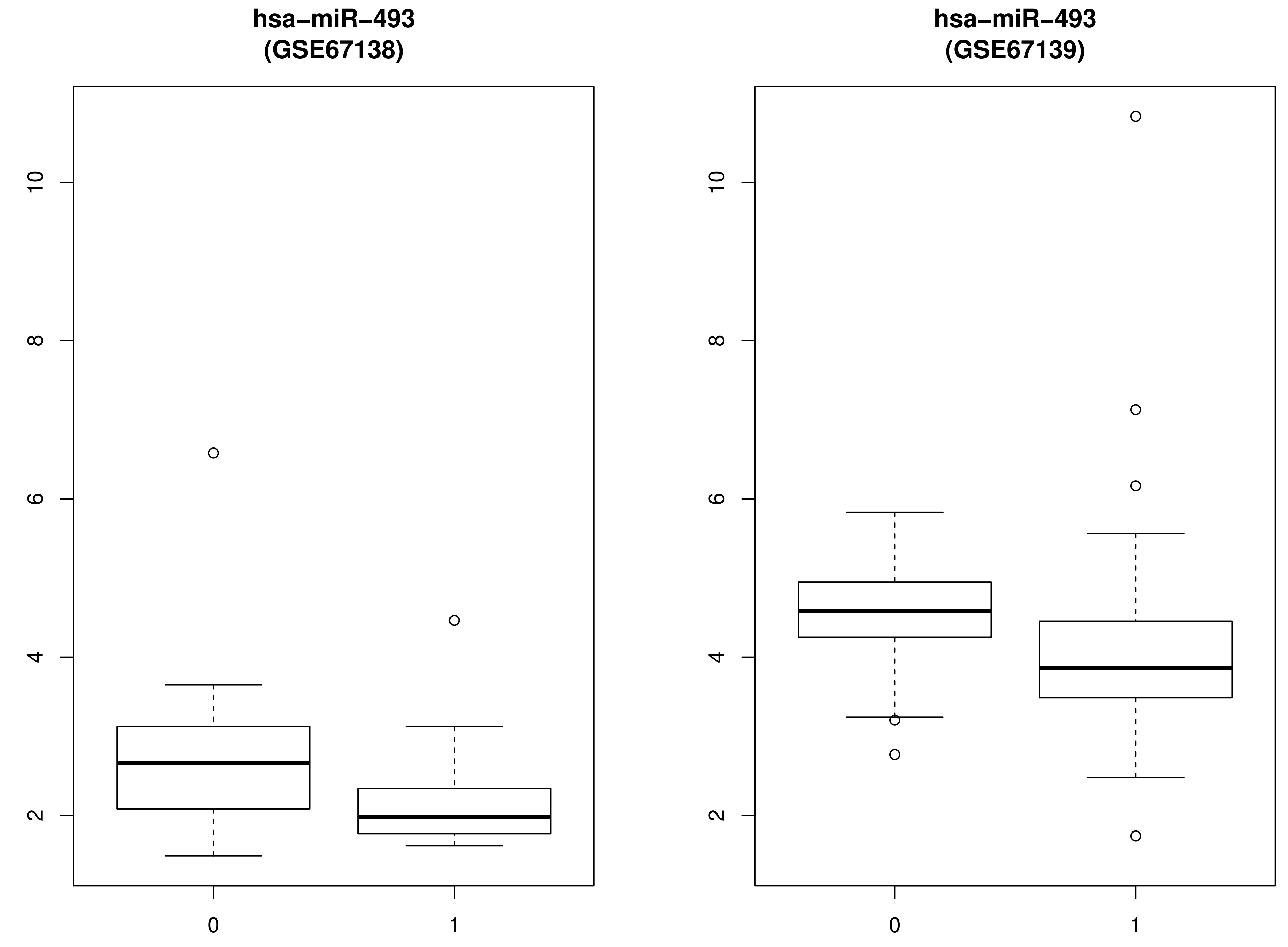
hsa−miR−493
(GSE67138)
hsa−miR−493
(GSE67139)
2
4
6
8
10
0
1

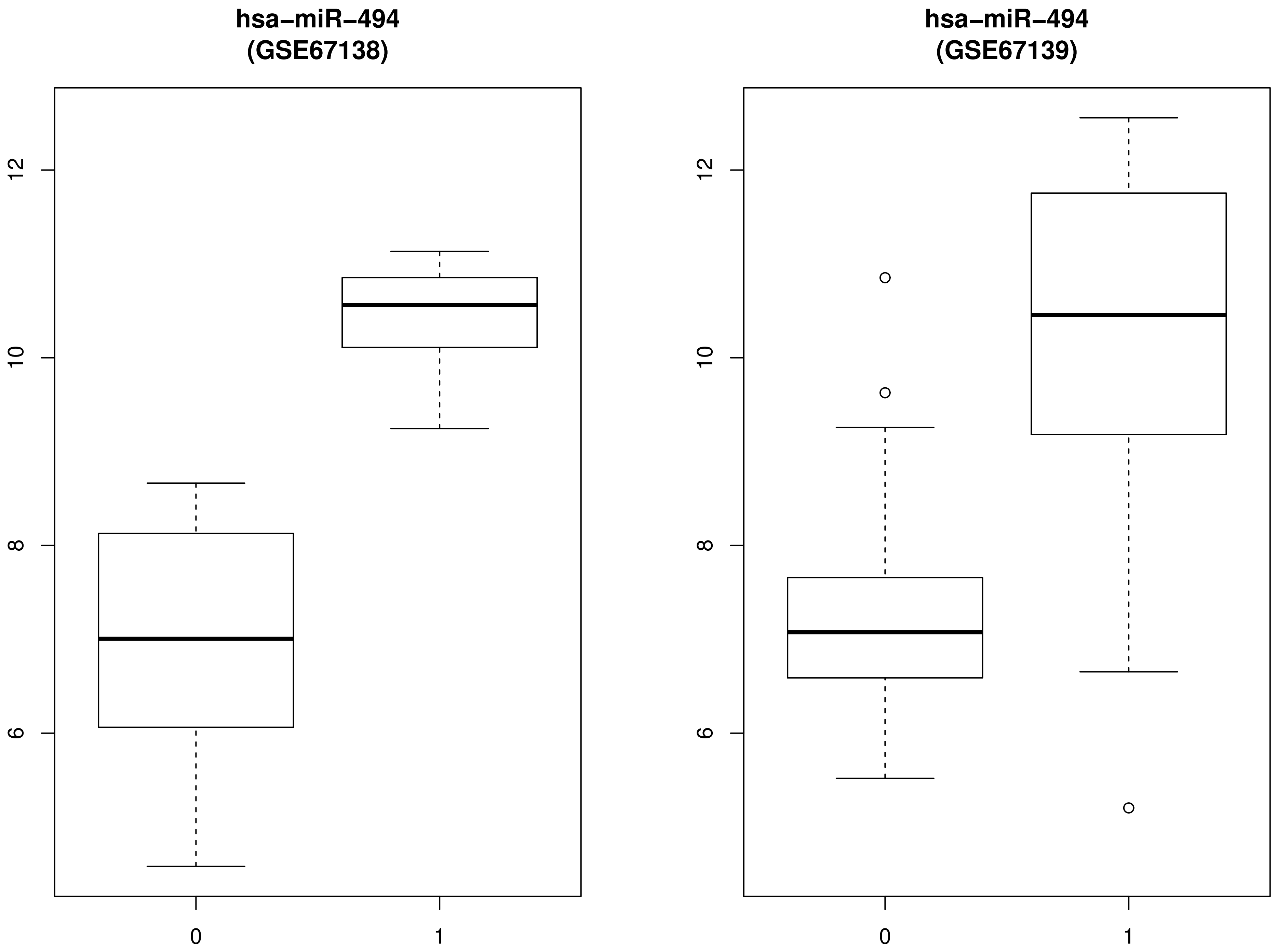
hsa−miR−494
(GSE67138)
hsa−miR−494
(GSE67139)
6
8
10
12
0
1
6
8
10
12
0
1

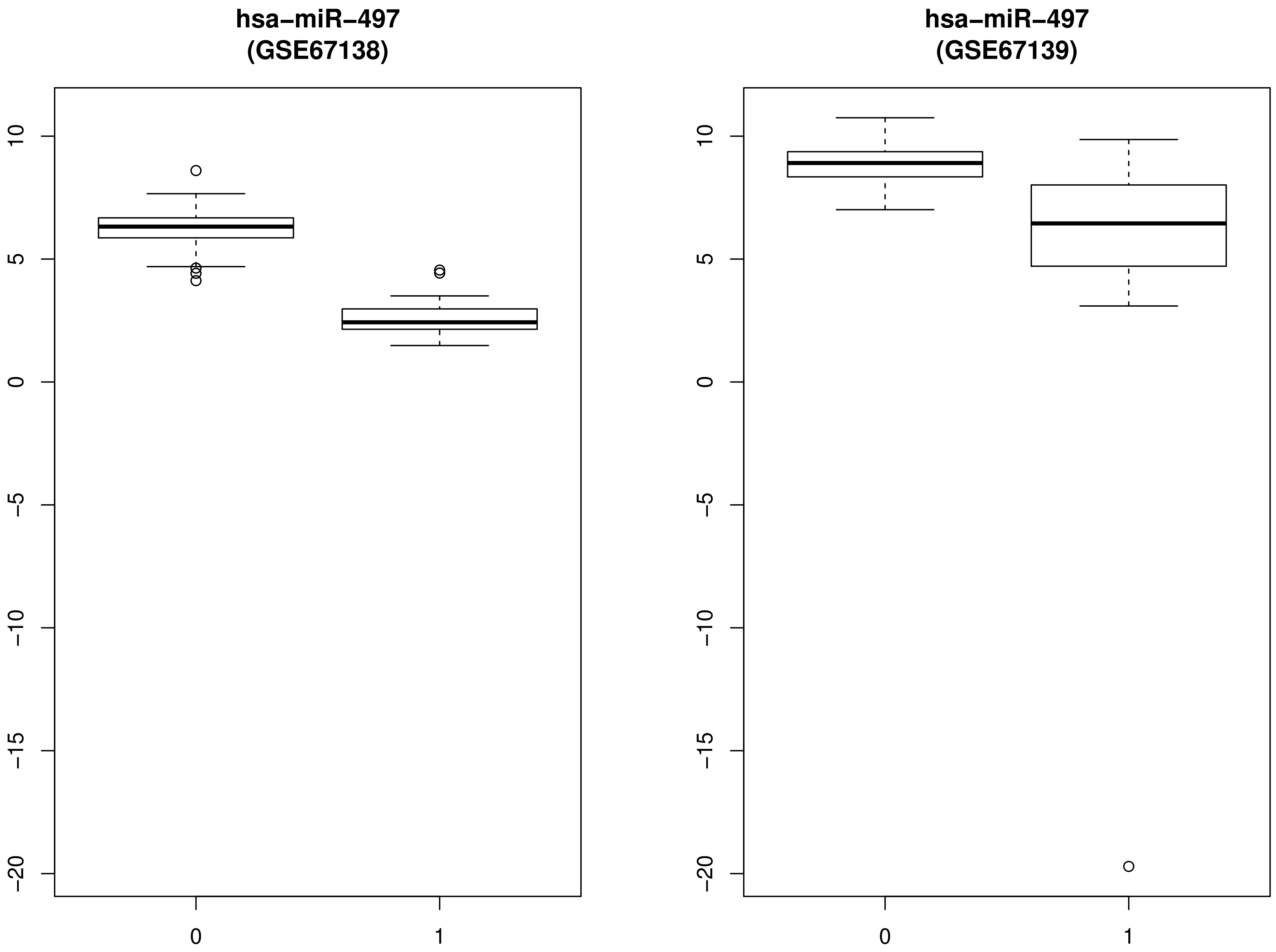
hsa−miR−497
(GSE67138)
hsa−miR−497
(GSE67139)
10
5
0
−5
−10
−15
−20
0
1

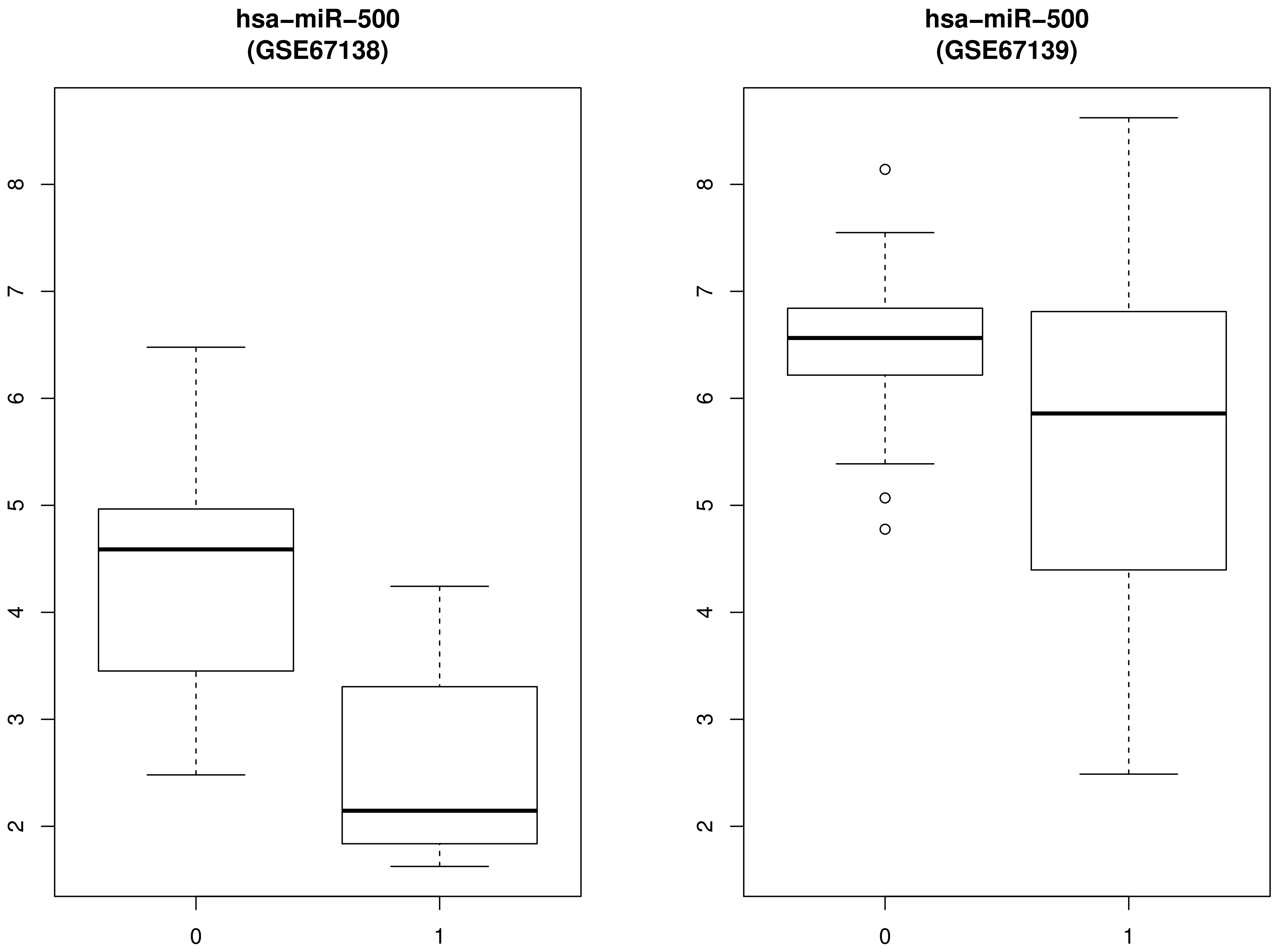

hsa−miR−500
(GSE67138)
hsa−miR−500
(GSE67139)
2
3
4
5
6
7
8
0
1

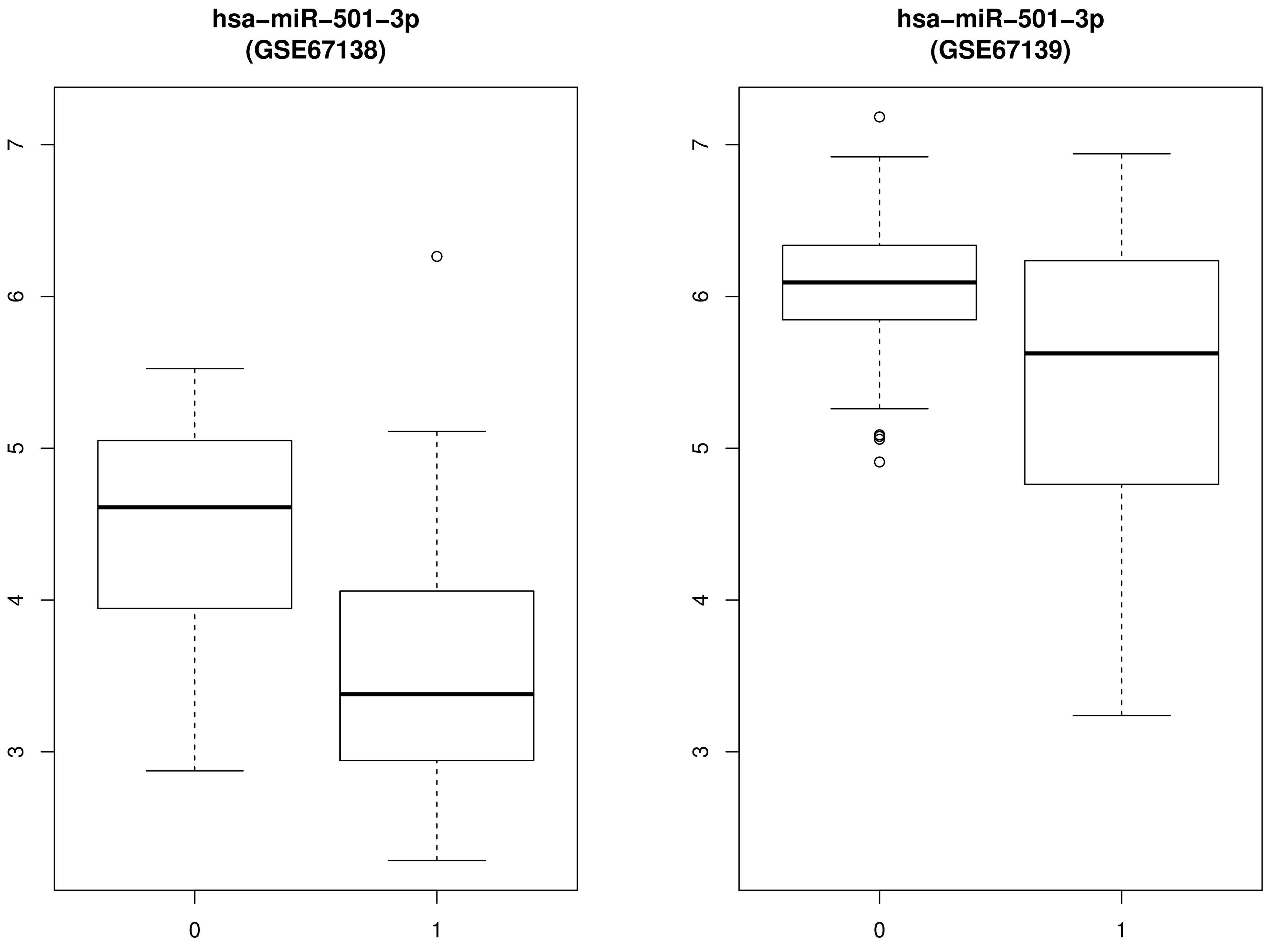
hsa−miR−501−3p
(GSE67138)
hsa−miR−501−3p
(GSE67139)
3
4
5
6
7
0
1

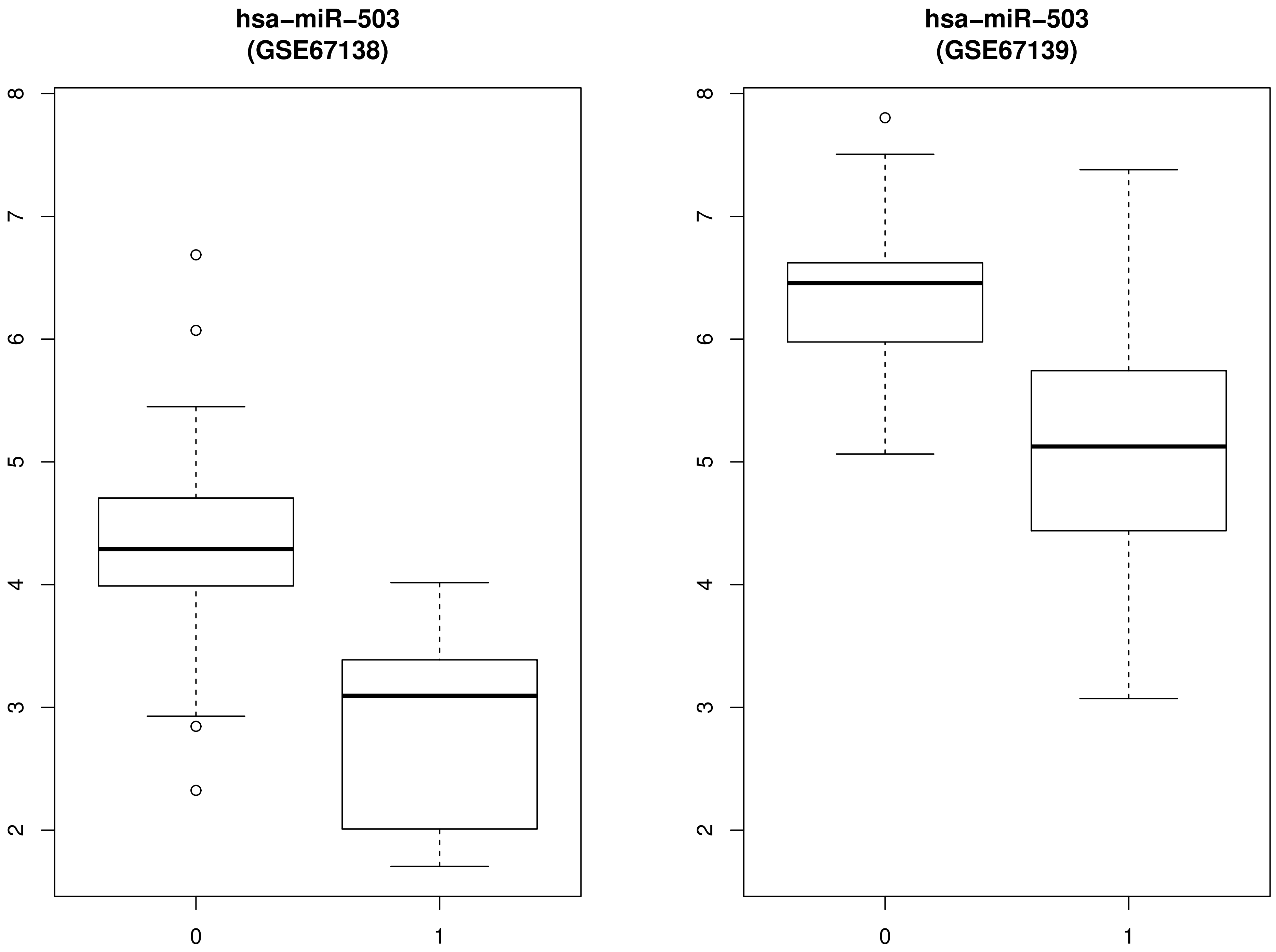

hsa−miR−503
(GSE67138)
hsa−miR−503
(GSE67139)
2
3
4
5
6
7
8
0
1

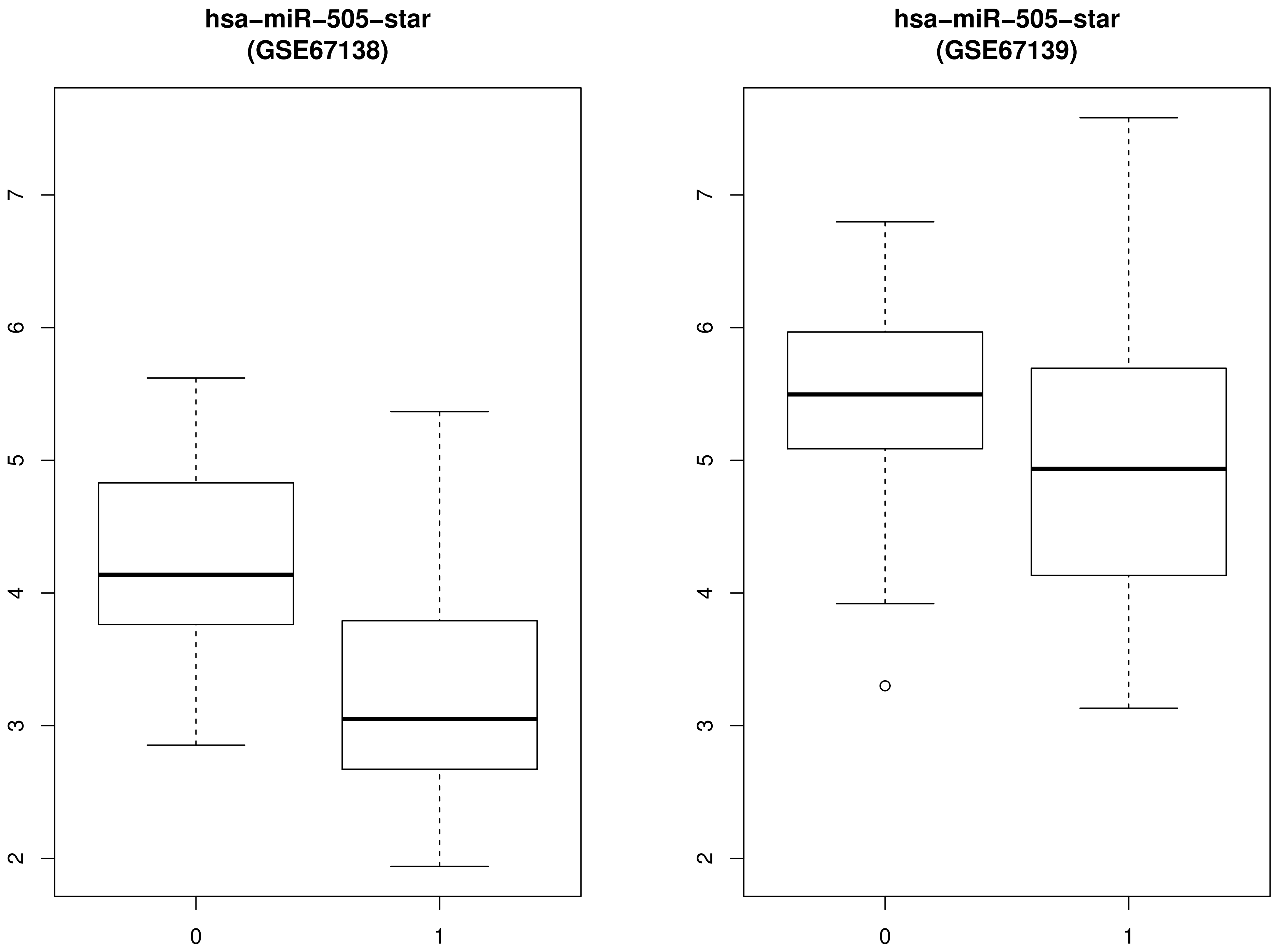
hsa−miR−505−star
(GSE67138)
hsa−miR−505−star
(GSE67139)
2
3
4
5
6
7
0
1

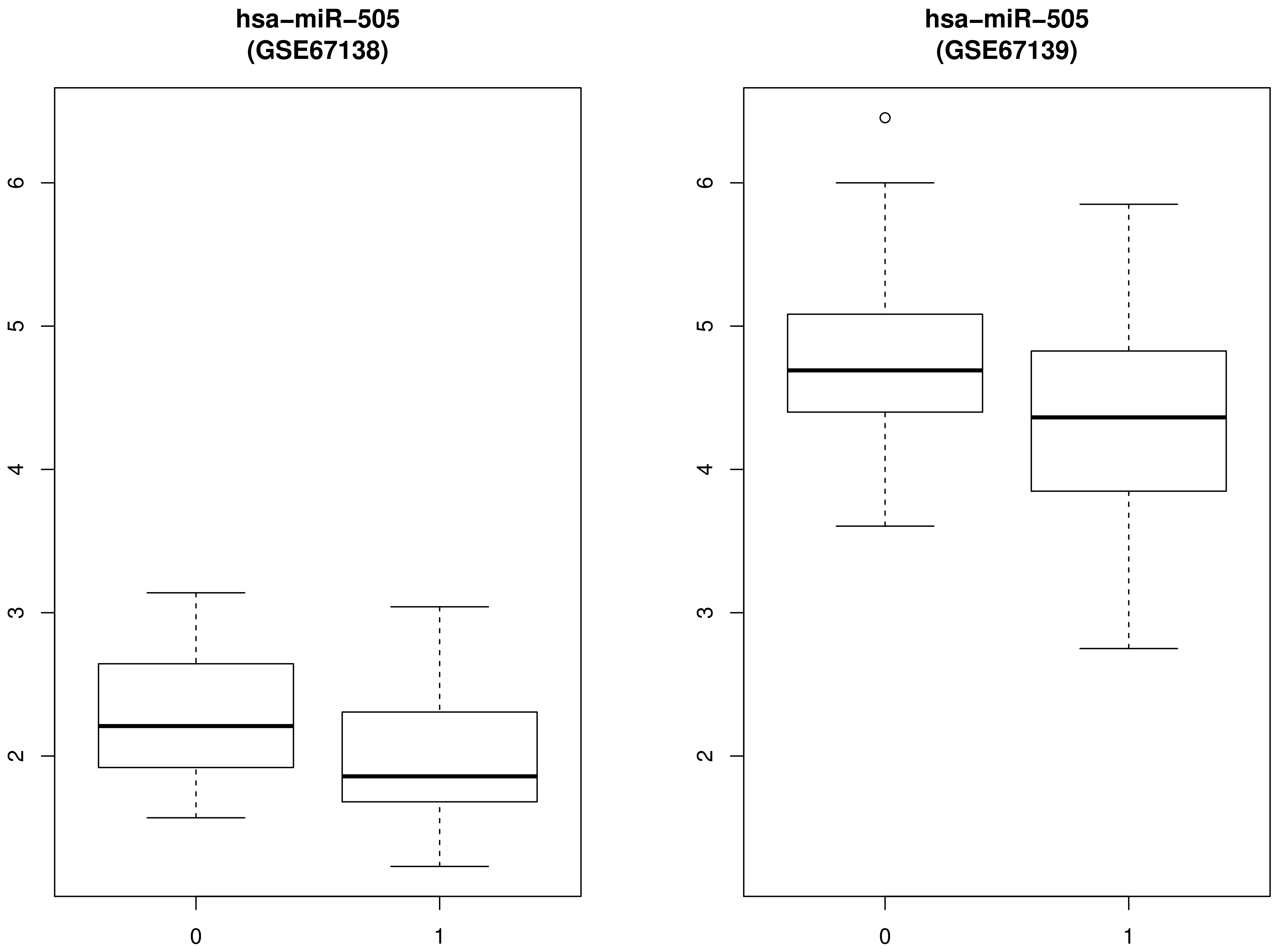
hsa−miR−505
(GSE67138)
hsa−miR−505
(GSE67139)
2
3
4
5
6
0
1

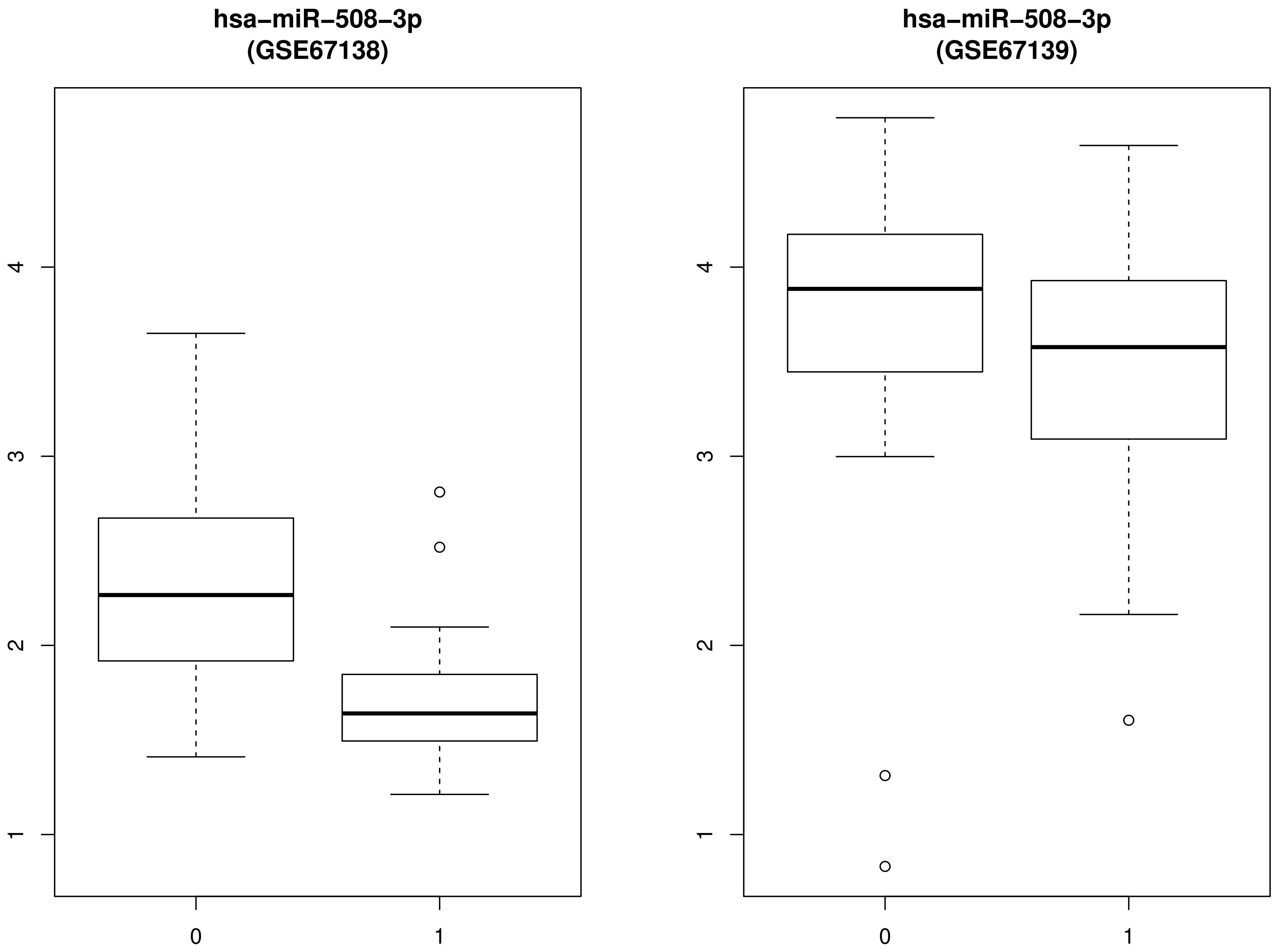
hsa−miR−508−3p
(GSE67138)
1
2
3
4
0
1
hsa−miR−508−3p
(GSE67139)
1
2
3
4
0
1

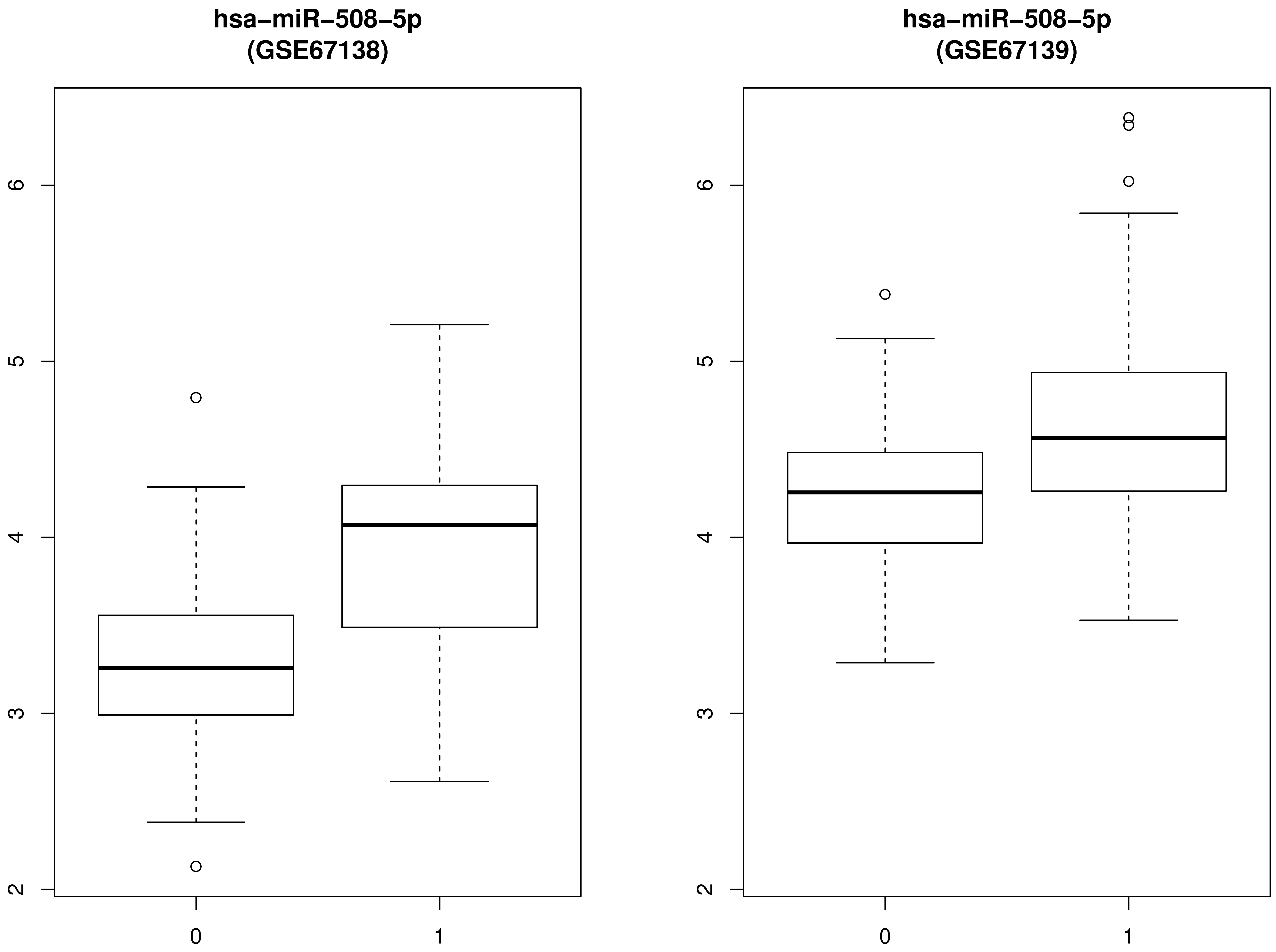
hsa−miR−508−5p
(GSE67138)
hsa−miR−508−5p
(GSE67139)
2
3
4
5
6
0
1

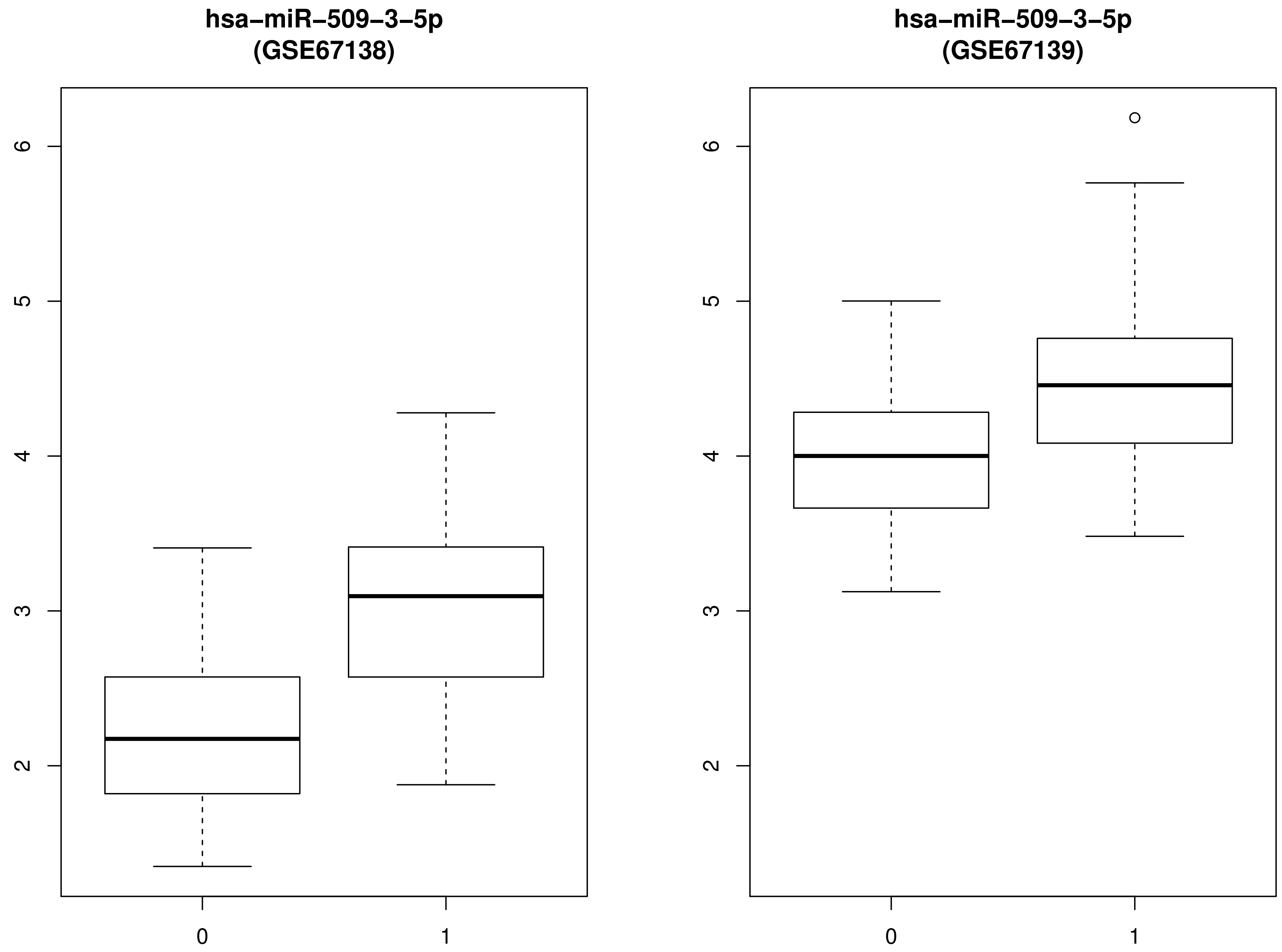
hsa–miR–509–3–5p
(GSE67138)
hsa–miR–509–3–5p
(GSE67139)
2
3
4
5
6
0
1

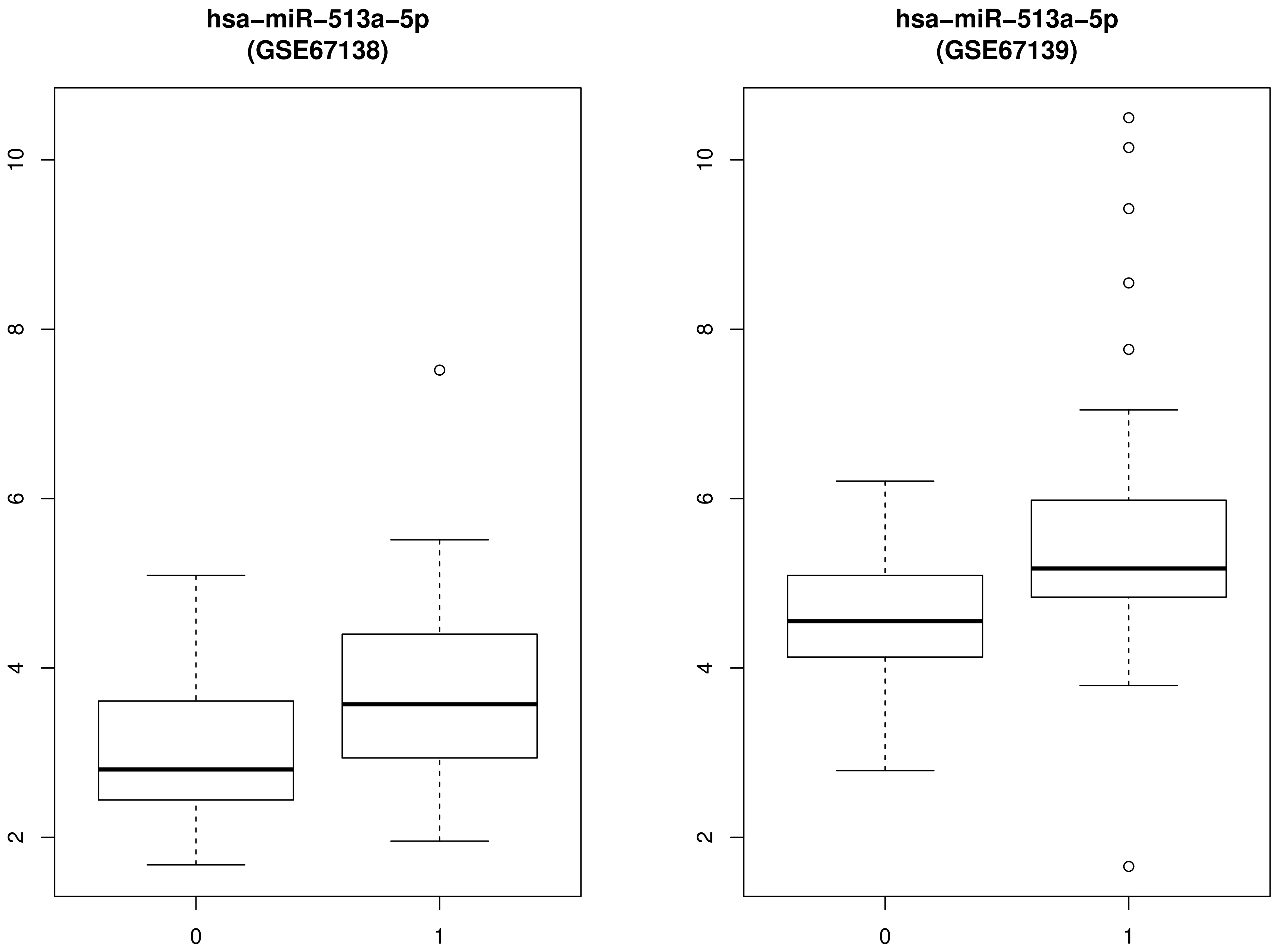
hsa−miR−513a−5p
(GSE67138)
hsa−miR−513a−5p
(GSE67139)
10
8
6
4
2
0
1
10
8
6
4
2
0
1

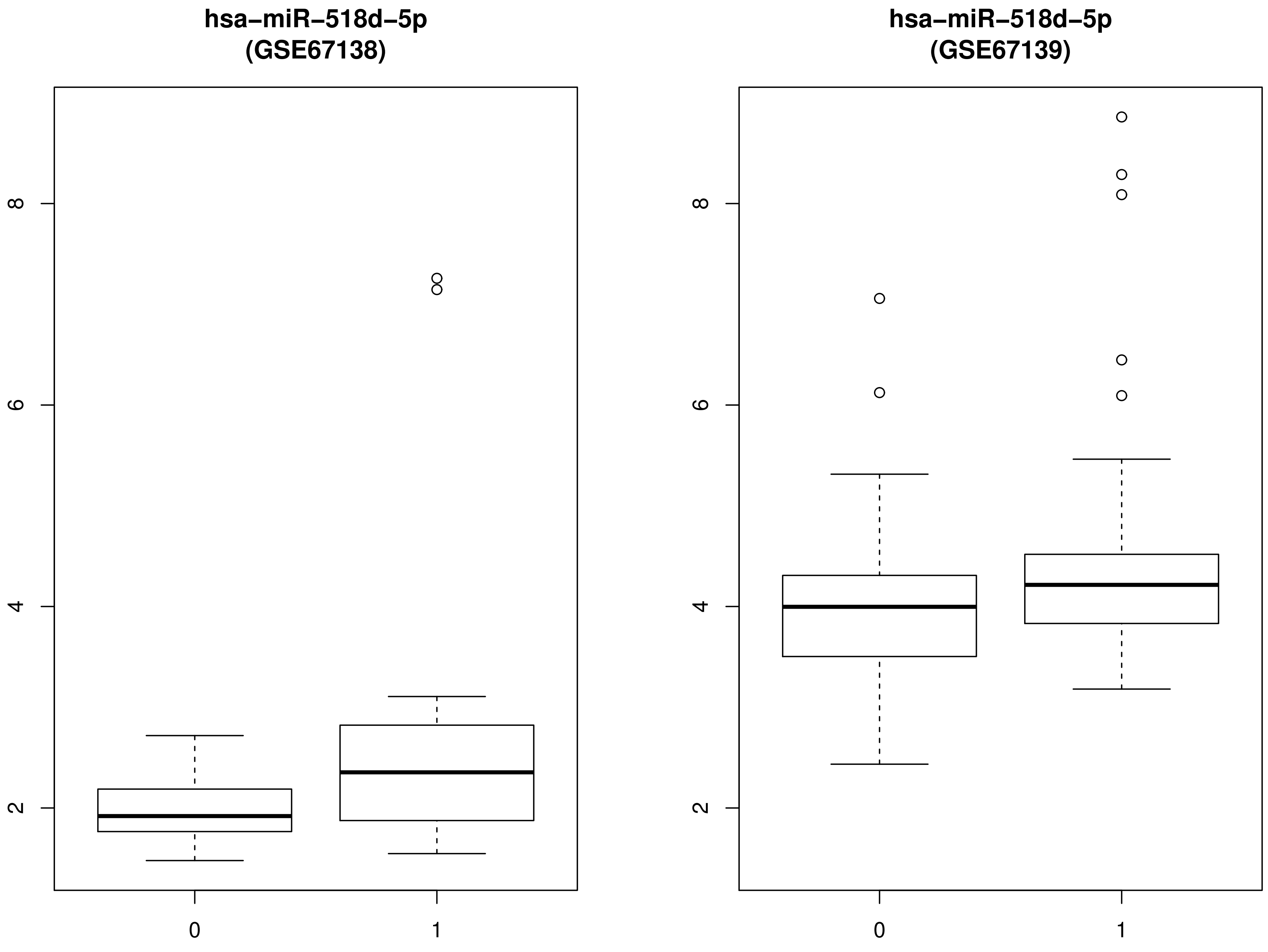
hsa−miR−518d−5p
(GSE67138)
hsa−miR−518d−5p
(GSE67139)
2
4
6
8
0
1

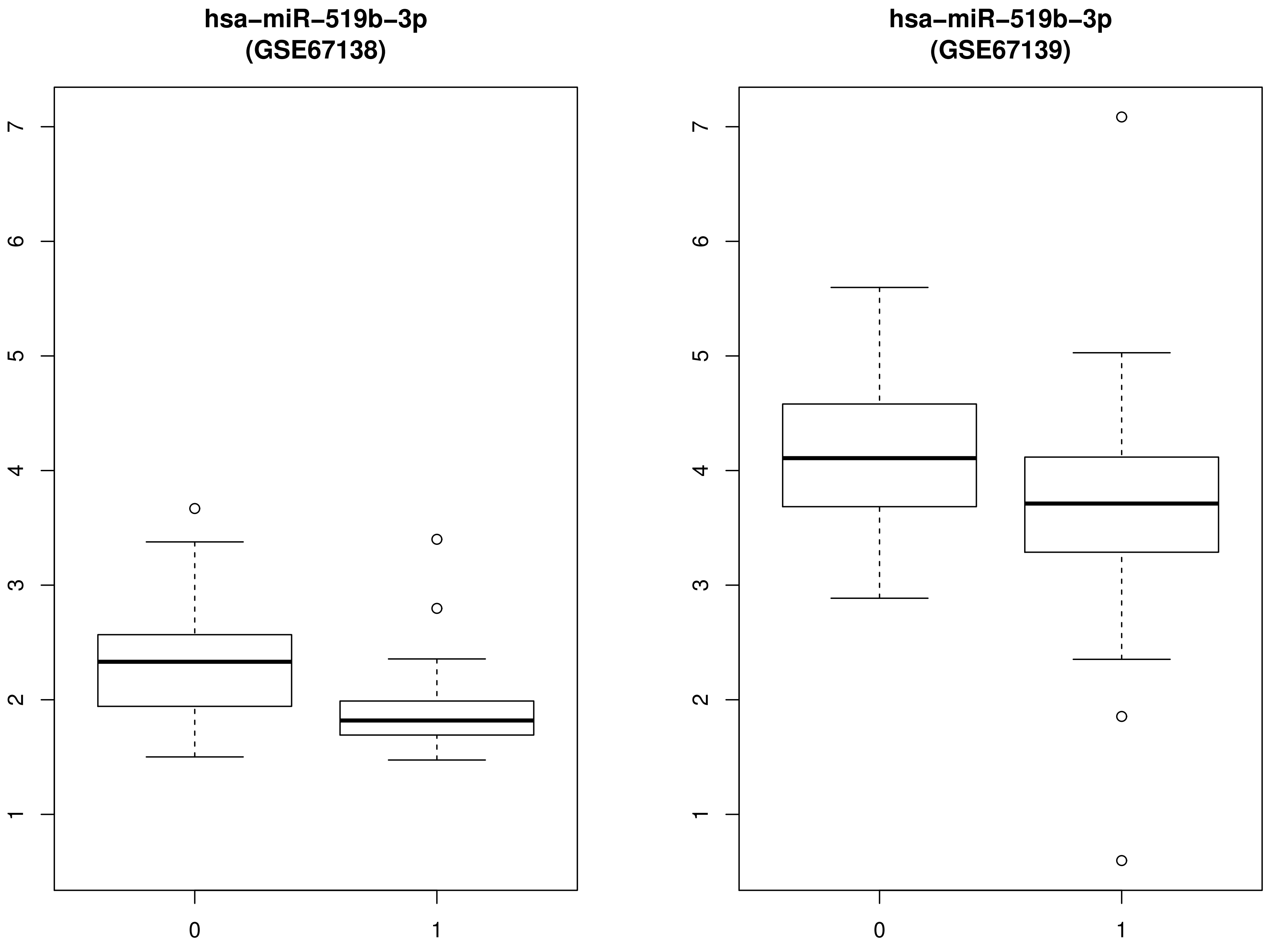

hsa−miR−519b−3p
(GSE67138)
hsa−miR−519b−3p
(GSE67139)
1
2
3
4
5
6
7
0
1

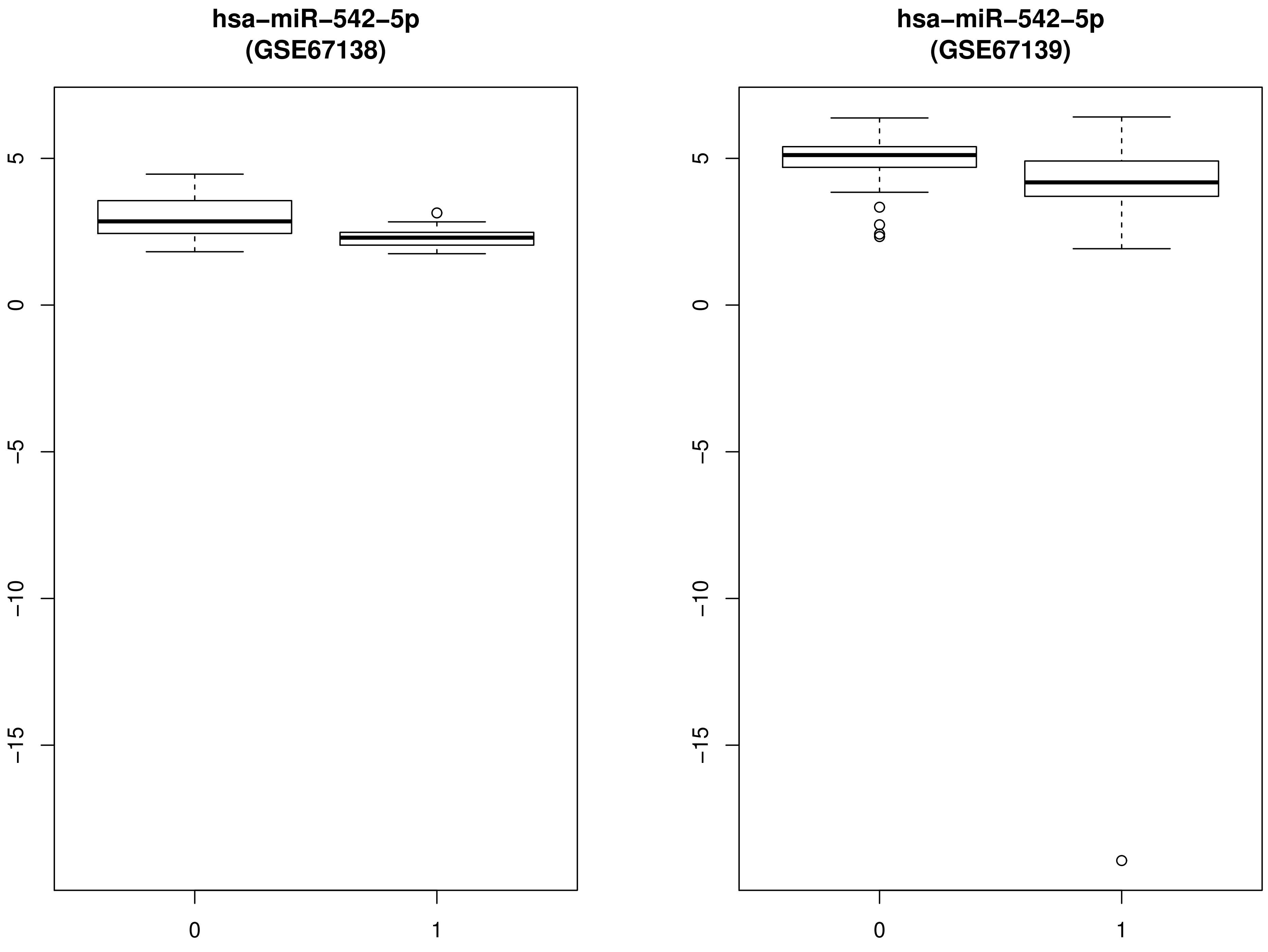
hsa−miR−542−5p
(GSE67138)
hsa−miR−542−5p
(GSE67139)
5
0
−5
−10
−15
0
1

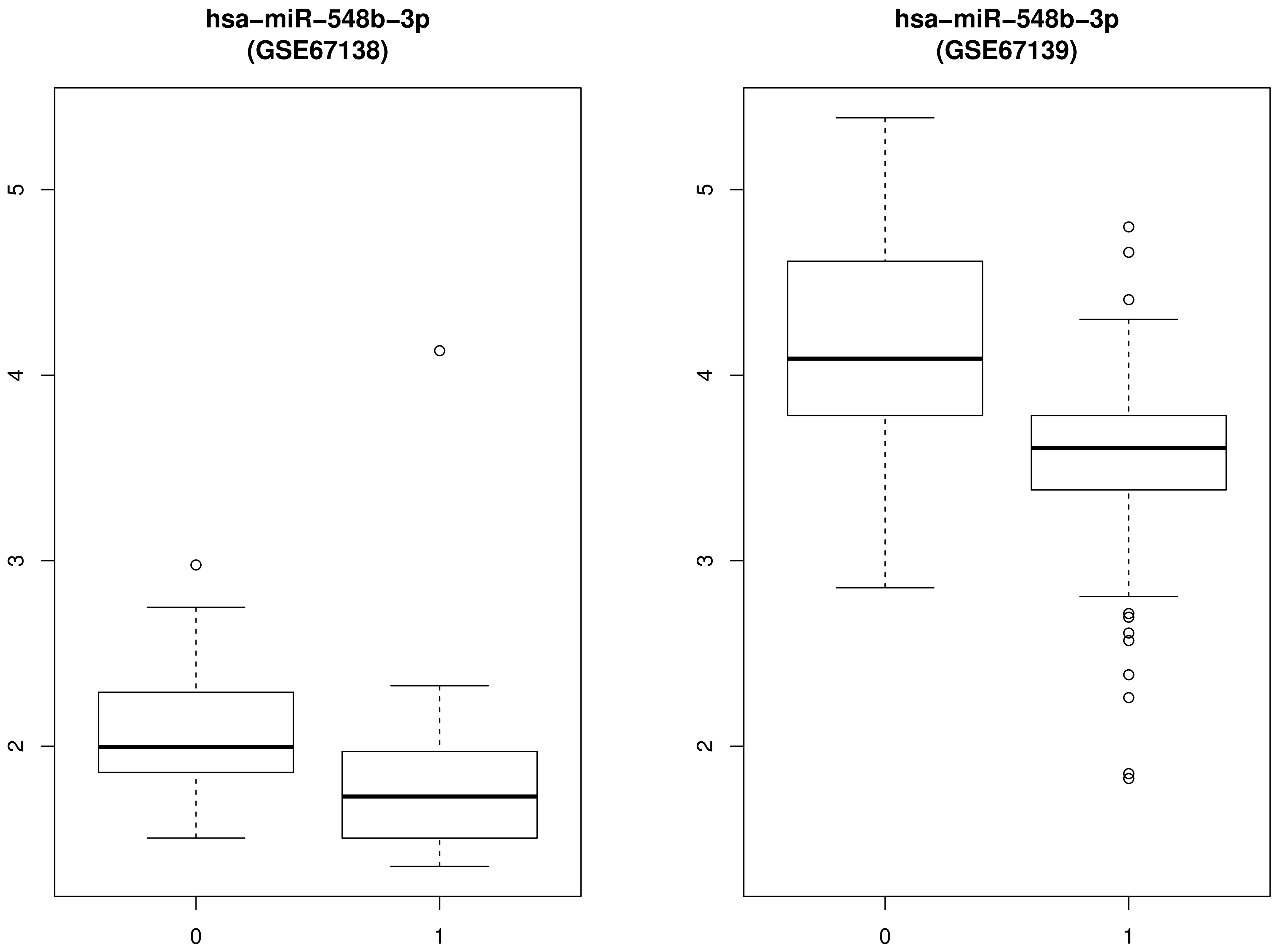

hsa−miR−548b−3p
(GSE67138)
hsa−miR−548b−3p
(GSE67139)
2
3
4
5
0
1

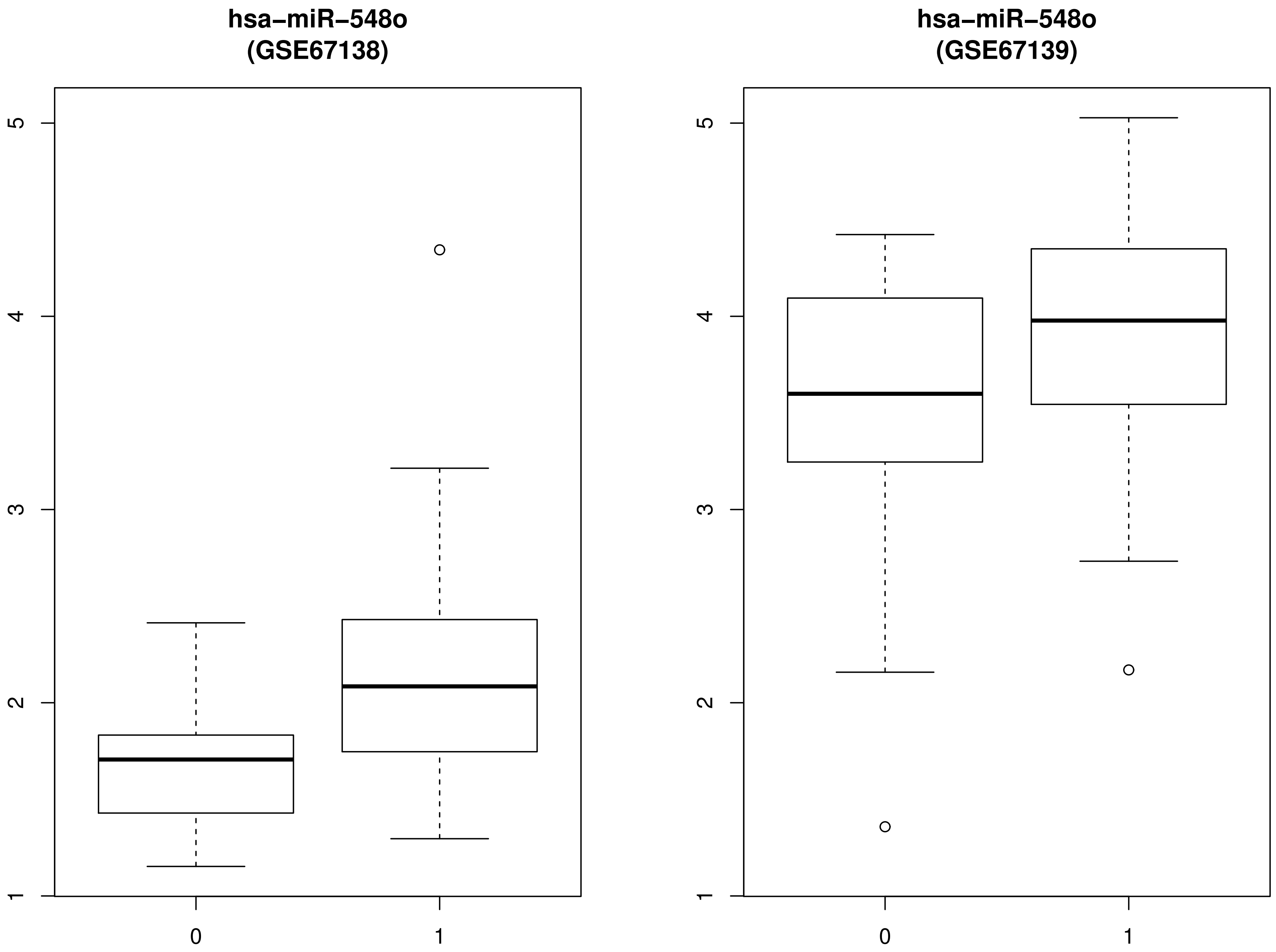
hsa−miR−548o
(GSE67138)
hsa−miR−548o
(GSE67139)
1
2
3
4
5
0
1

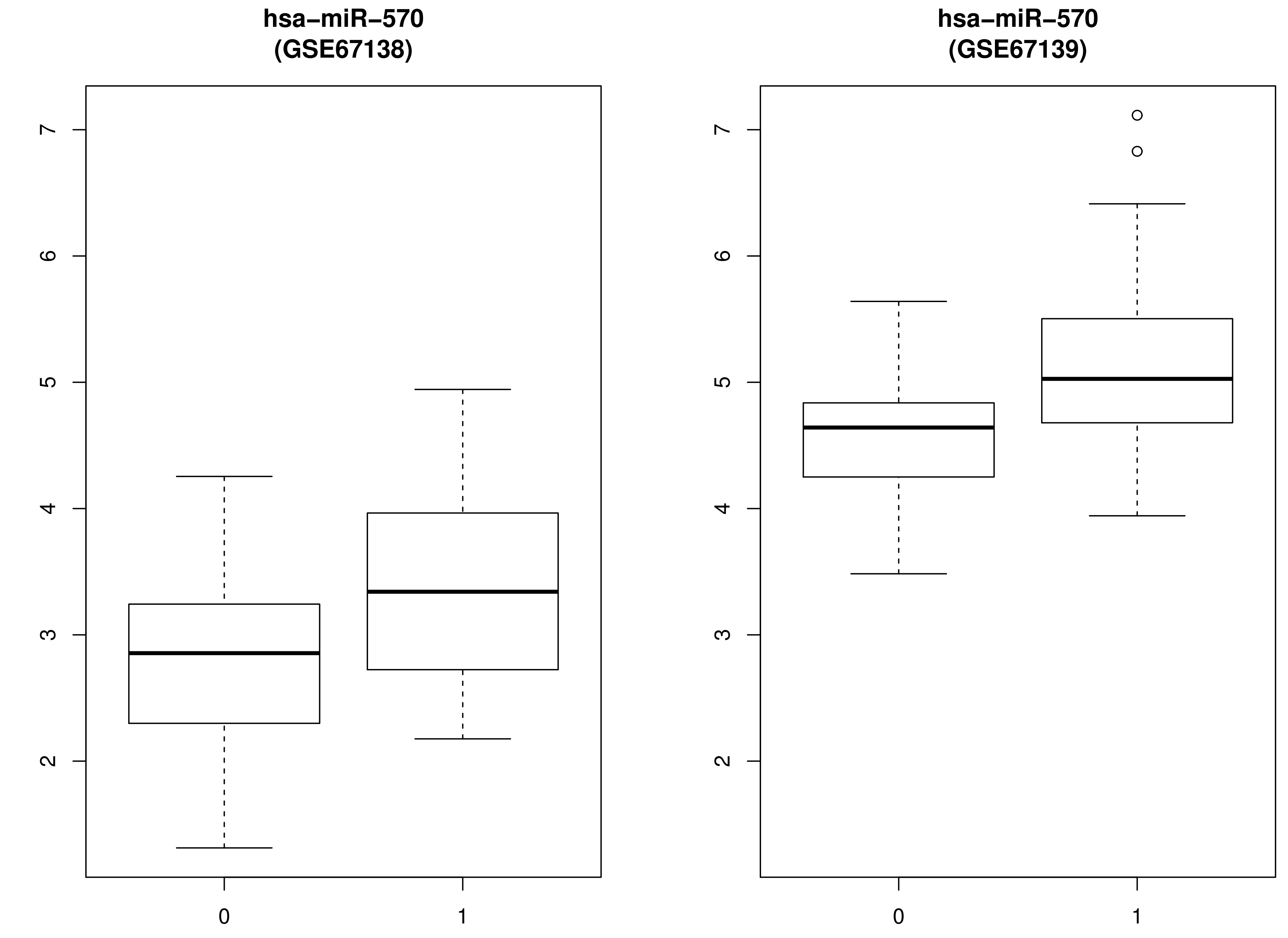
hsa−miR−570
(GSE67138)
hsa−miR−570
(GSE67139)
2
3
4
5
6
7
0
1

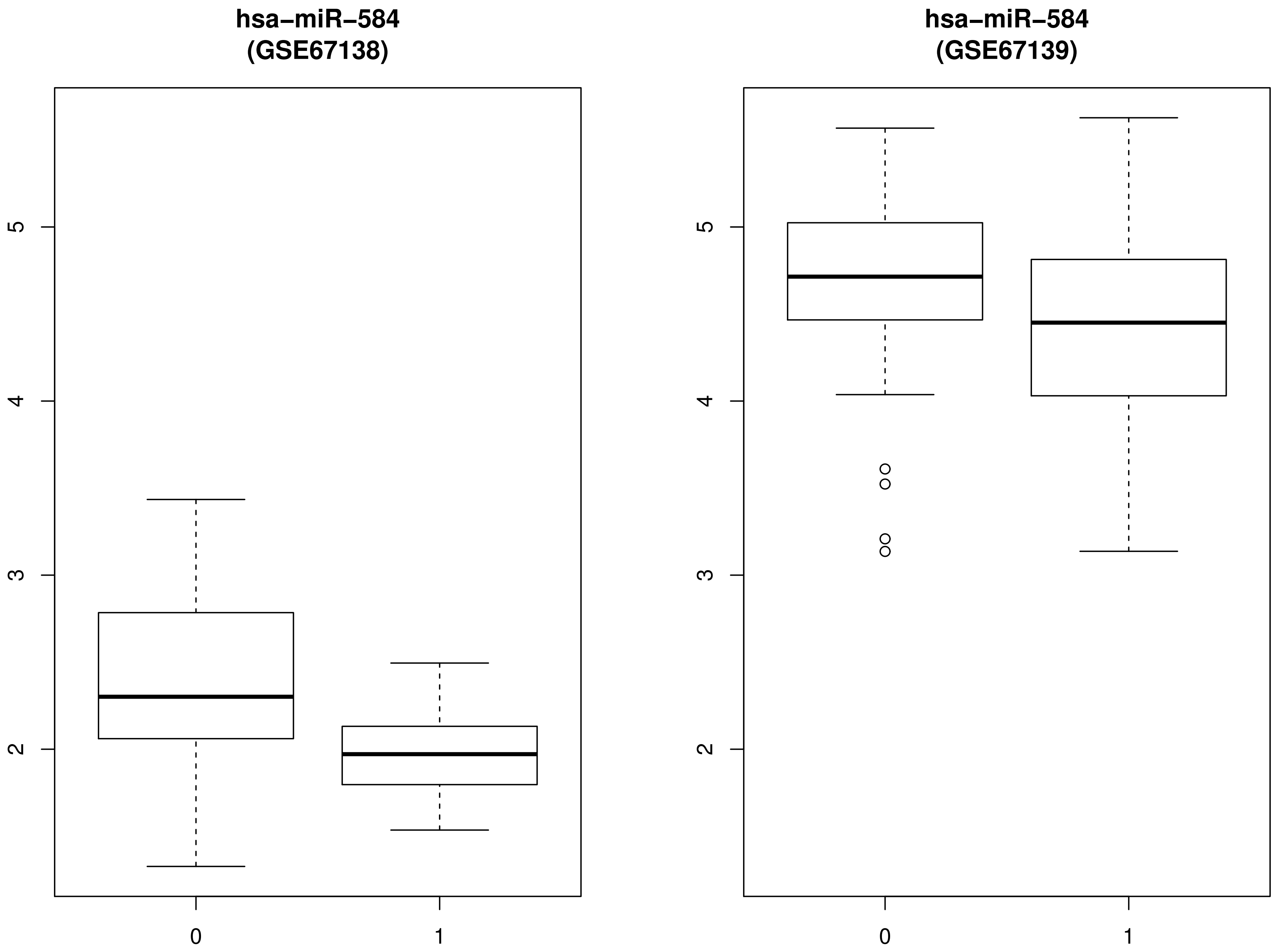
hsa−miR−584
(GSE67138)
5
4
3
2
0
1
hsa−miR−584
(GSE67139)
5
4
3
2
0
1

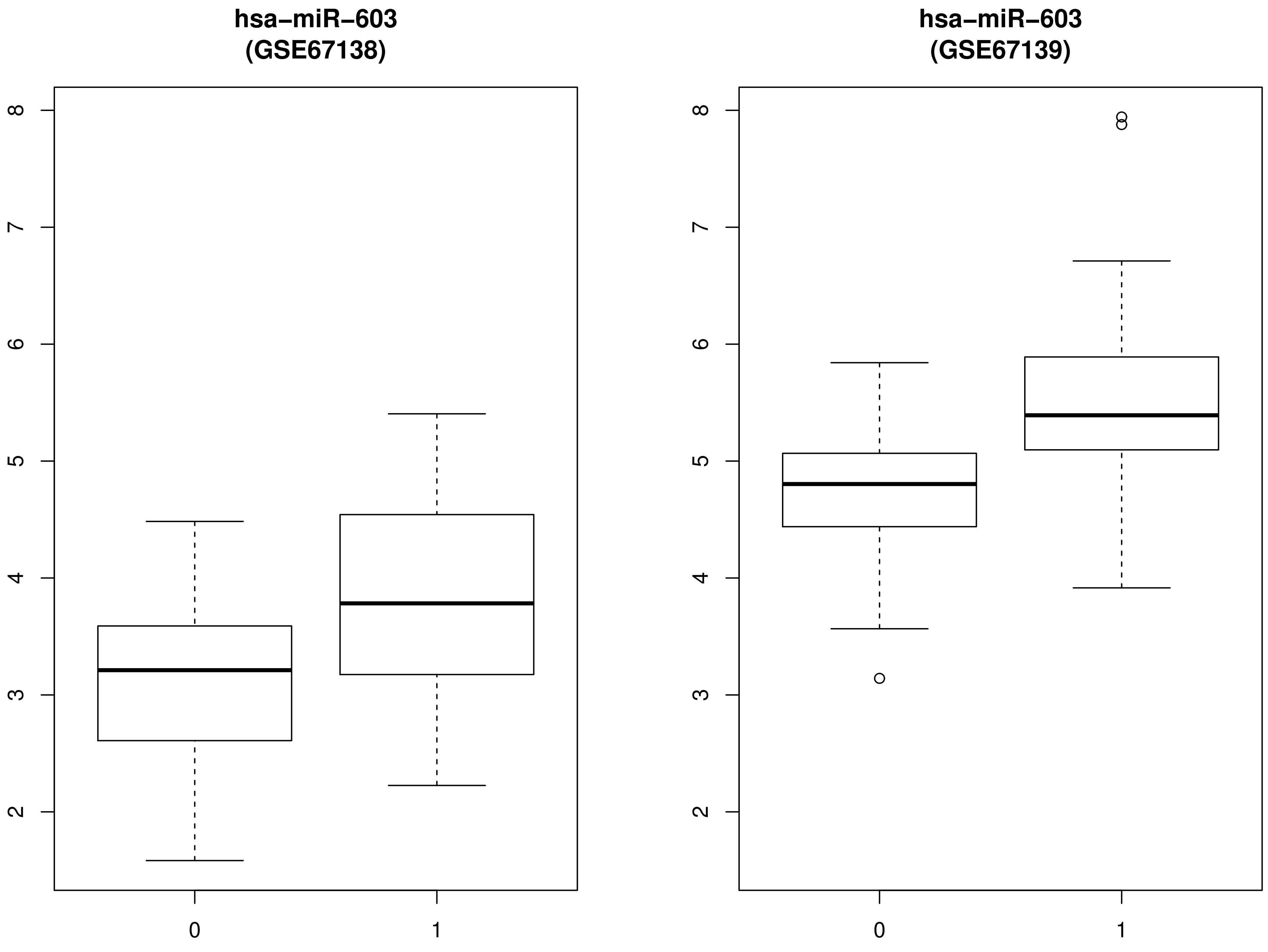
hsa−miR−603
(GSE67138)
2
3
4
5
6
7
8
0
1
hsa−miR−603
(GSE67139)
2
3
4
5
6
7
8
0
1

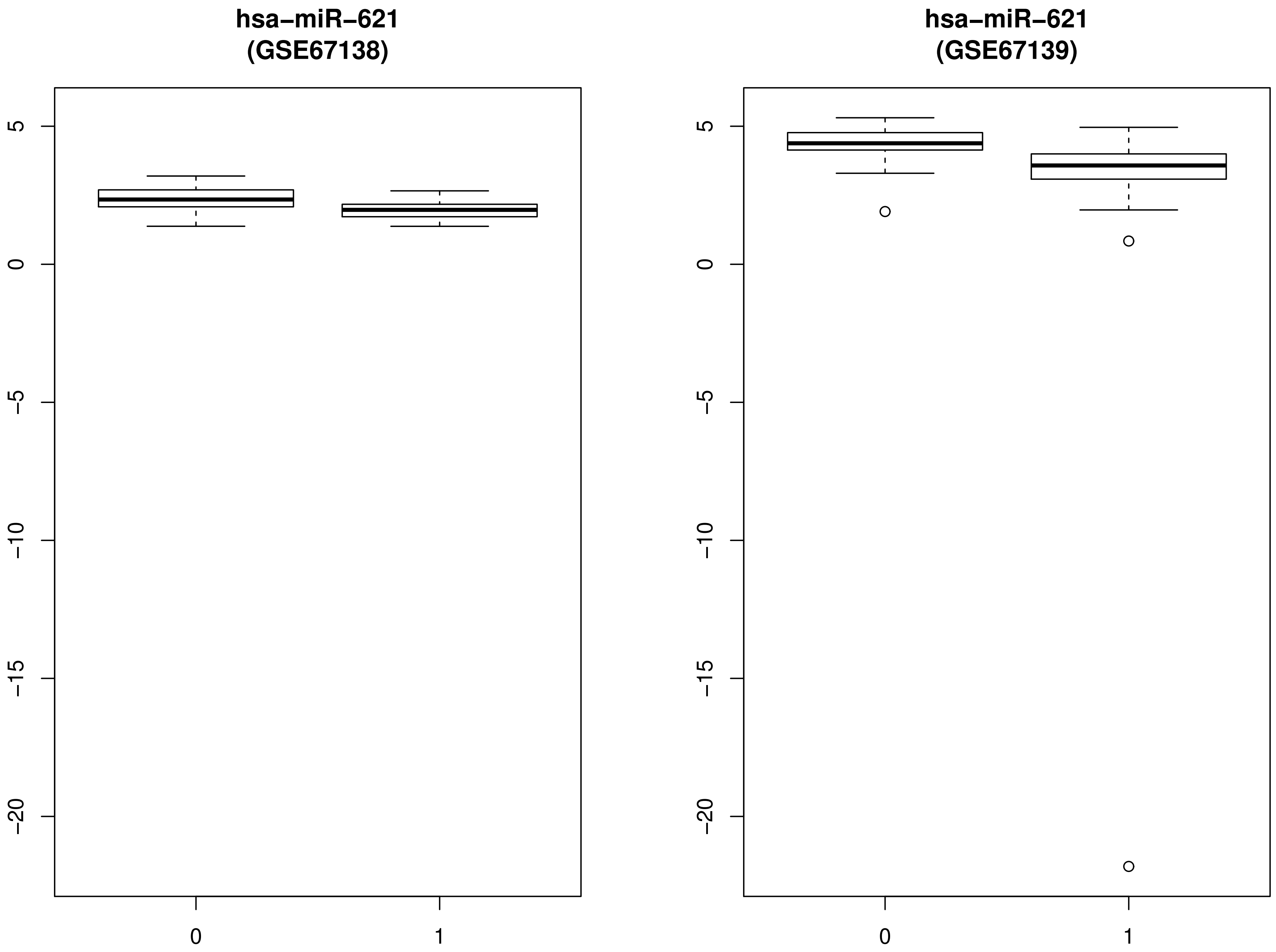

hsa−miR−621
(GSE67138)
hsa−miR−621
(GSE67139)
5
0
−5
−10
−15
−20
0
1

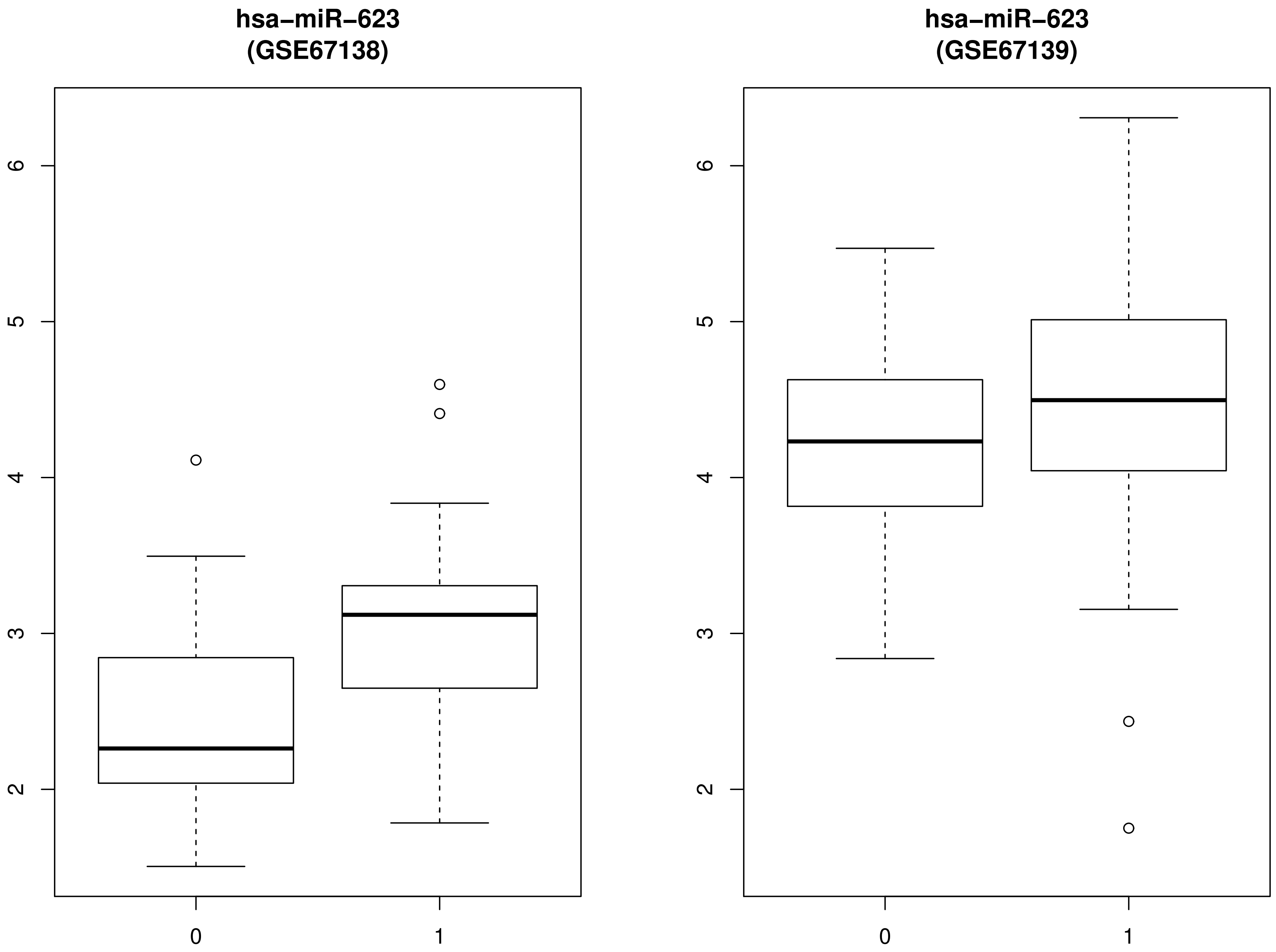
hsa−miR−623
(GSE67138)
hsa−miR−623
(GSE67139)
2
3
4
5
6
0
1

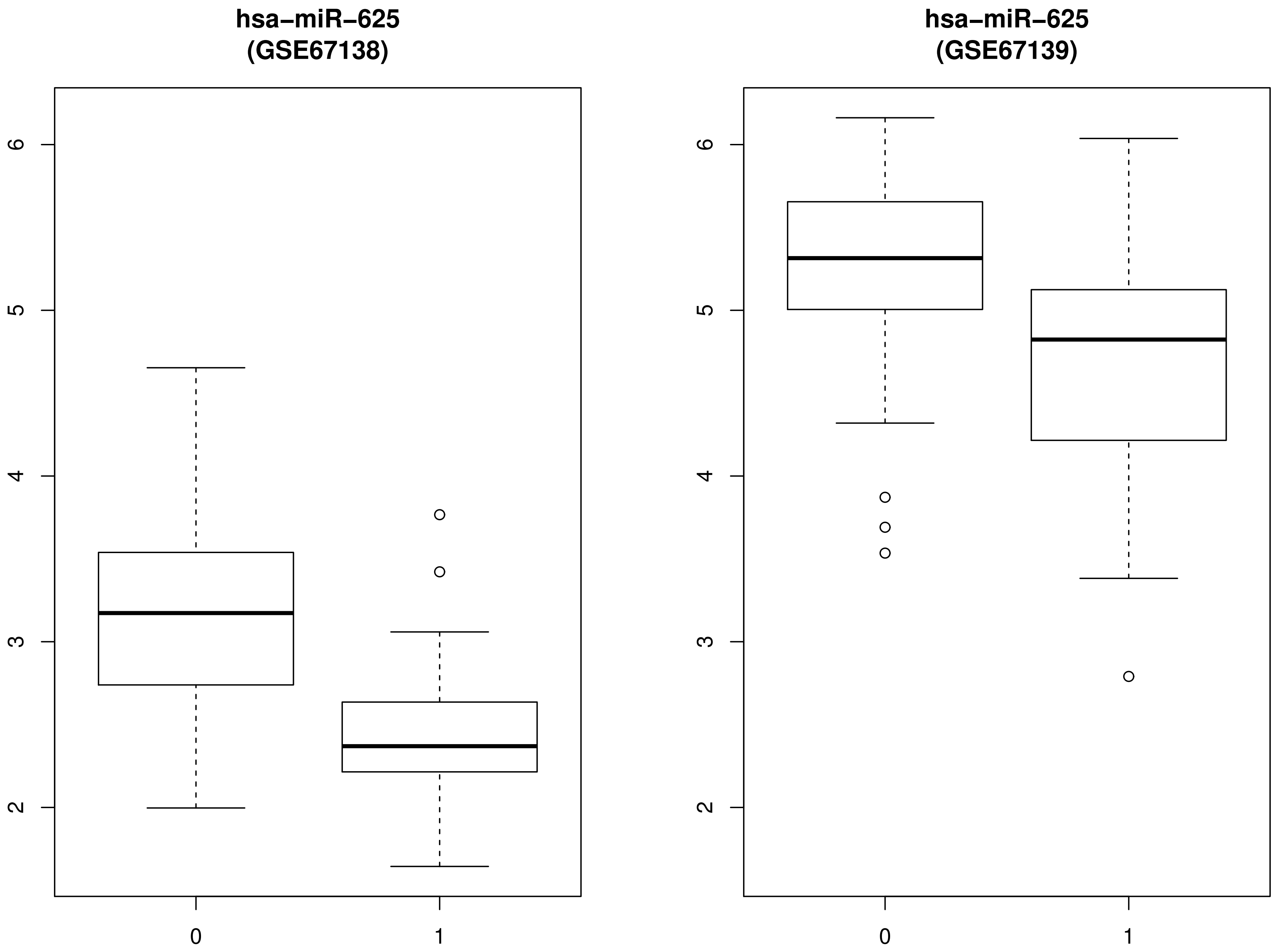

hsa−miR−625
(GSE67138)
hsa−miR−625
(GSE67139)
2
3
4
5
6
0
1

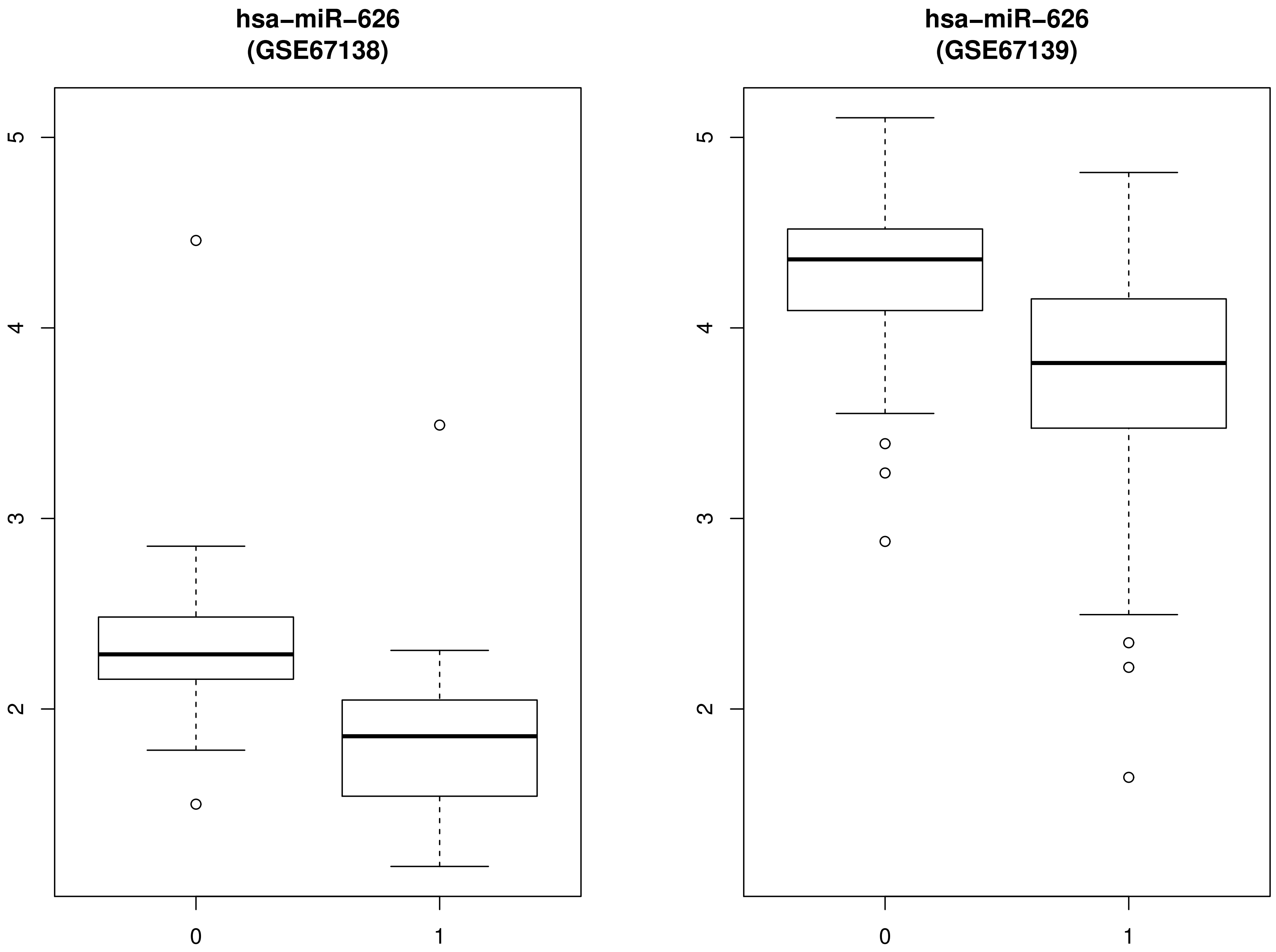
hsa−miR−626
(GSE67138)
hsa−miR−626
(GSE67139)
5
4
3
2
0
1

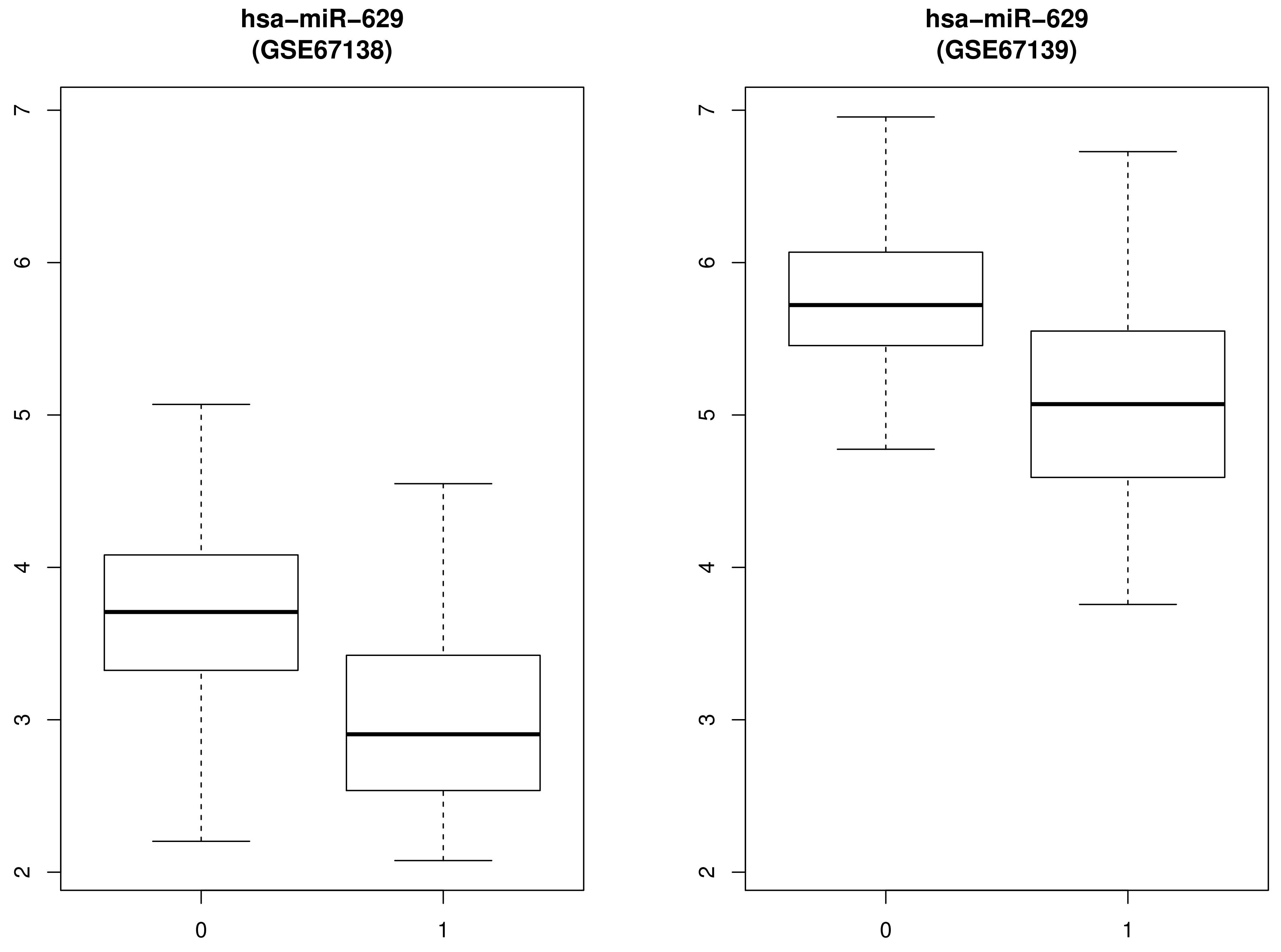
hsa−miR−629
(GSE67138)
hsa−miR−629
(GSE67139)
2
3
4
5
6
7
0
1

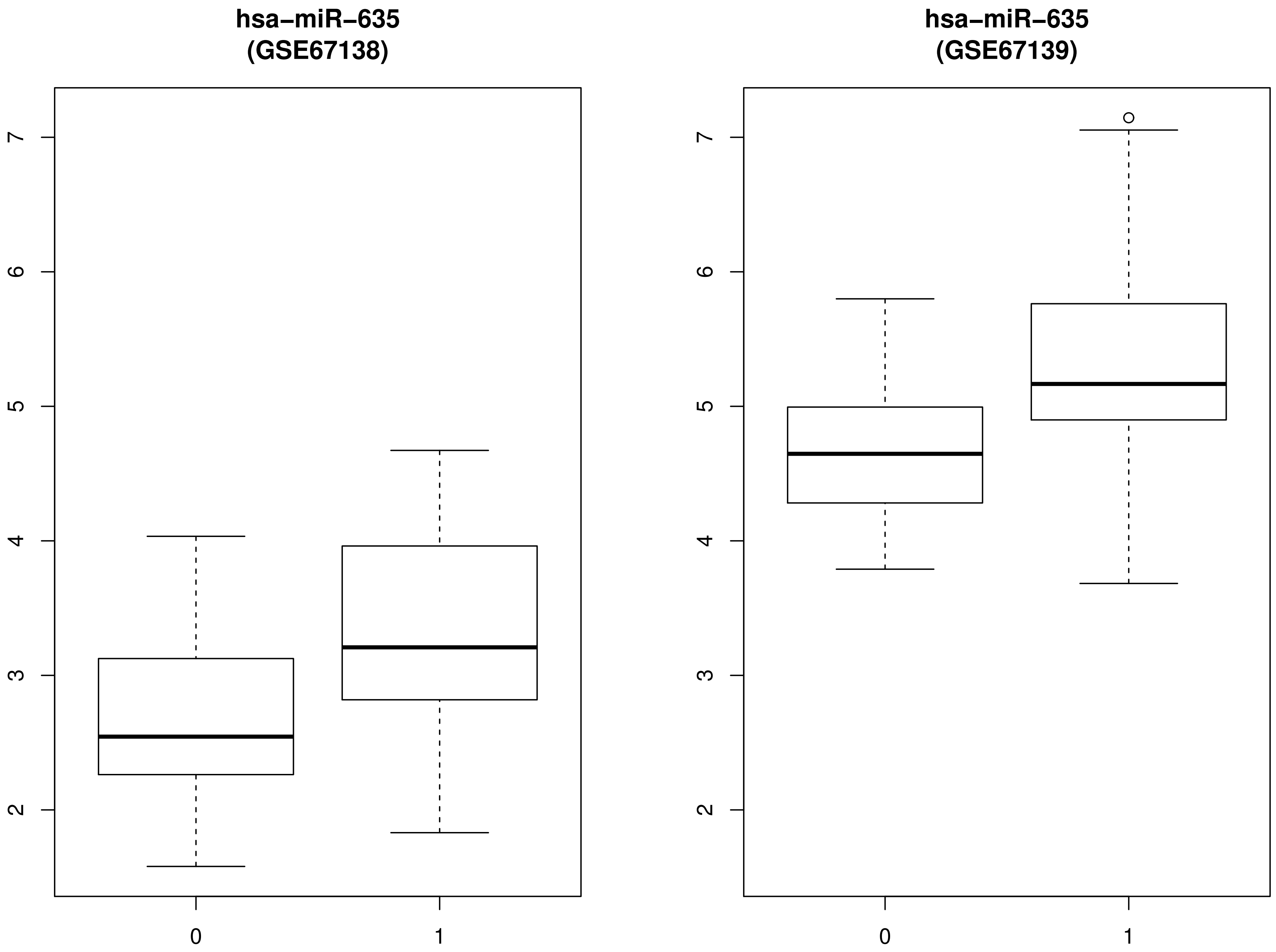
hsa−miR−635
(GSE67138)
hsa−miR−635
(GSE67139)
2
3
4
5
6
7
0
1

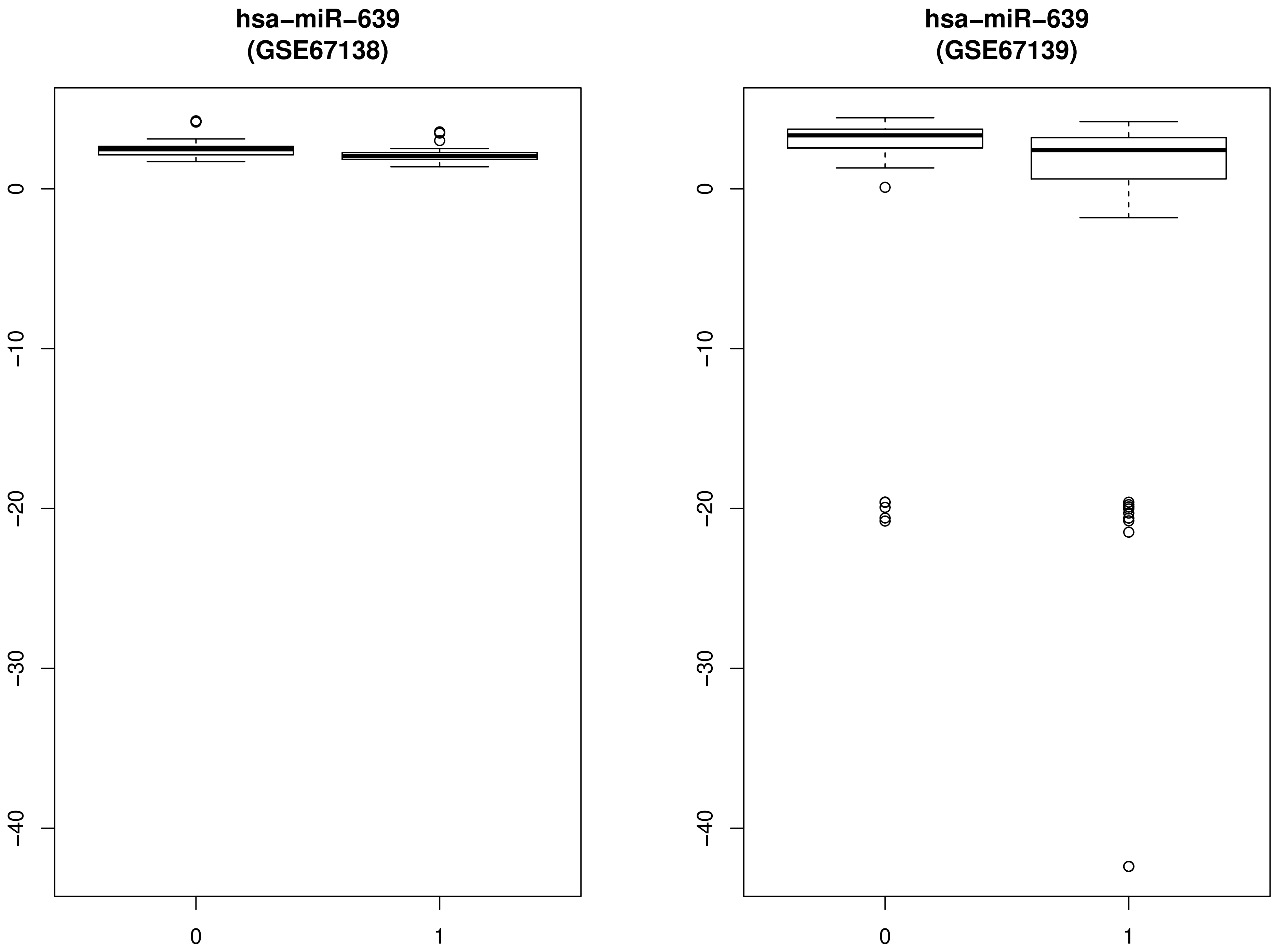
hsa−miR−639
(GSE67138)
hsa−miR−639
(GSE67139)
0
−10
−20
−30
−40
0
1

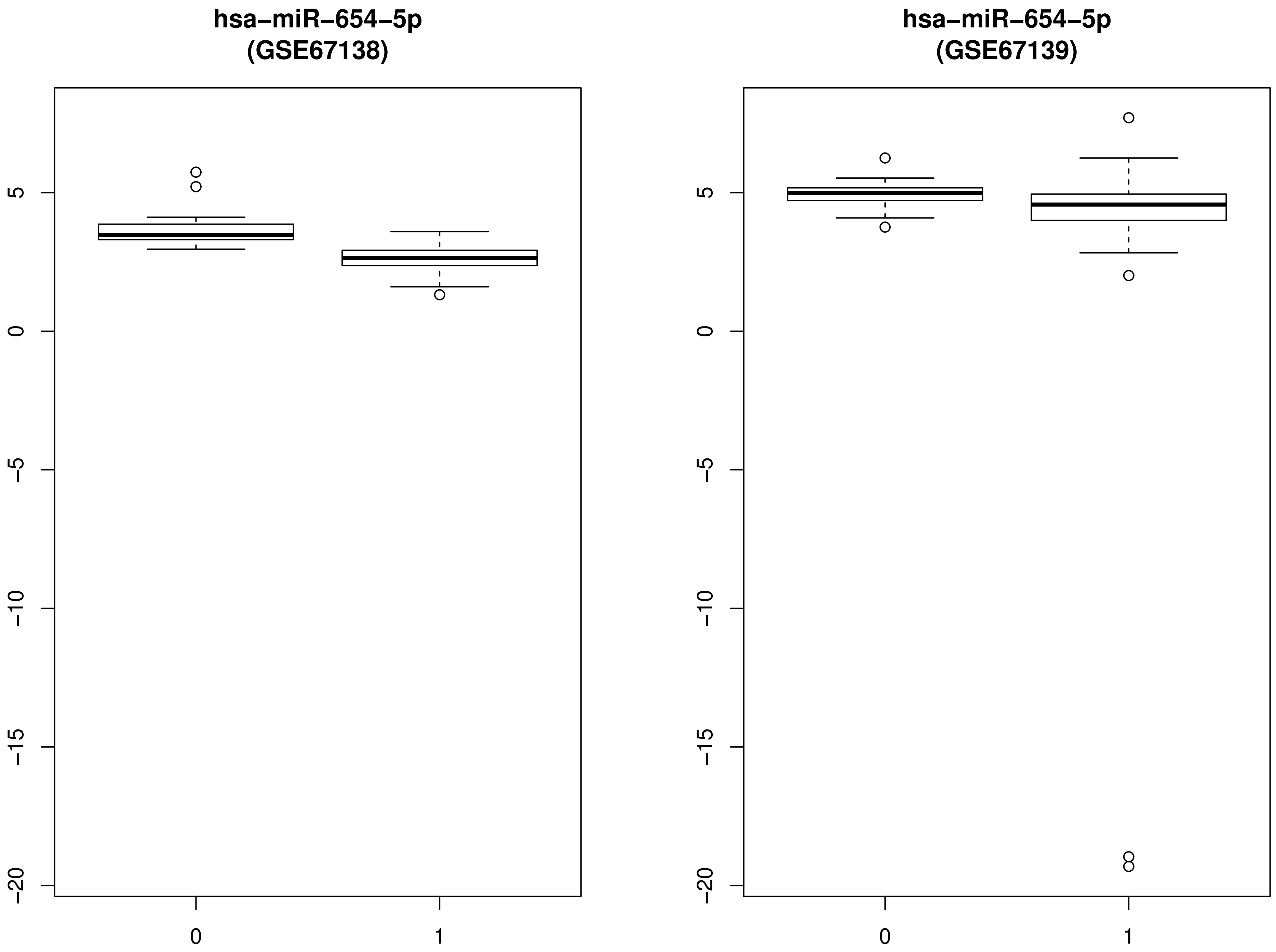
hsa−miR−654−5p
(GSE67138)
hsa−miR−654−5p
(GSE67139)
5
0
−5
−10
−15
−20
0
1

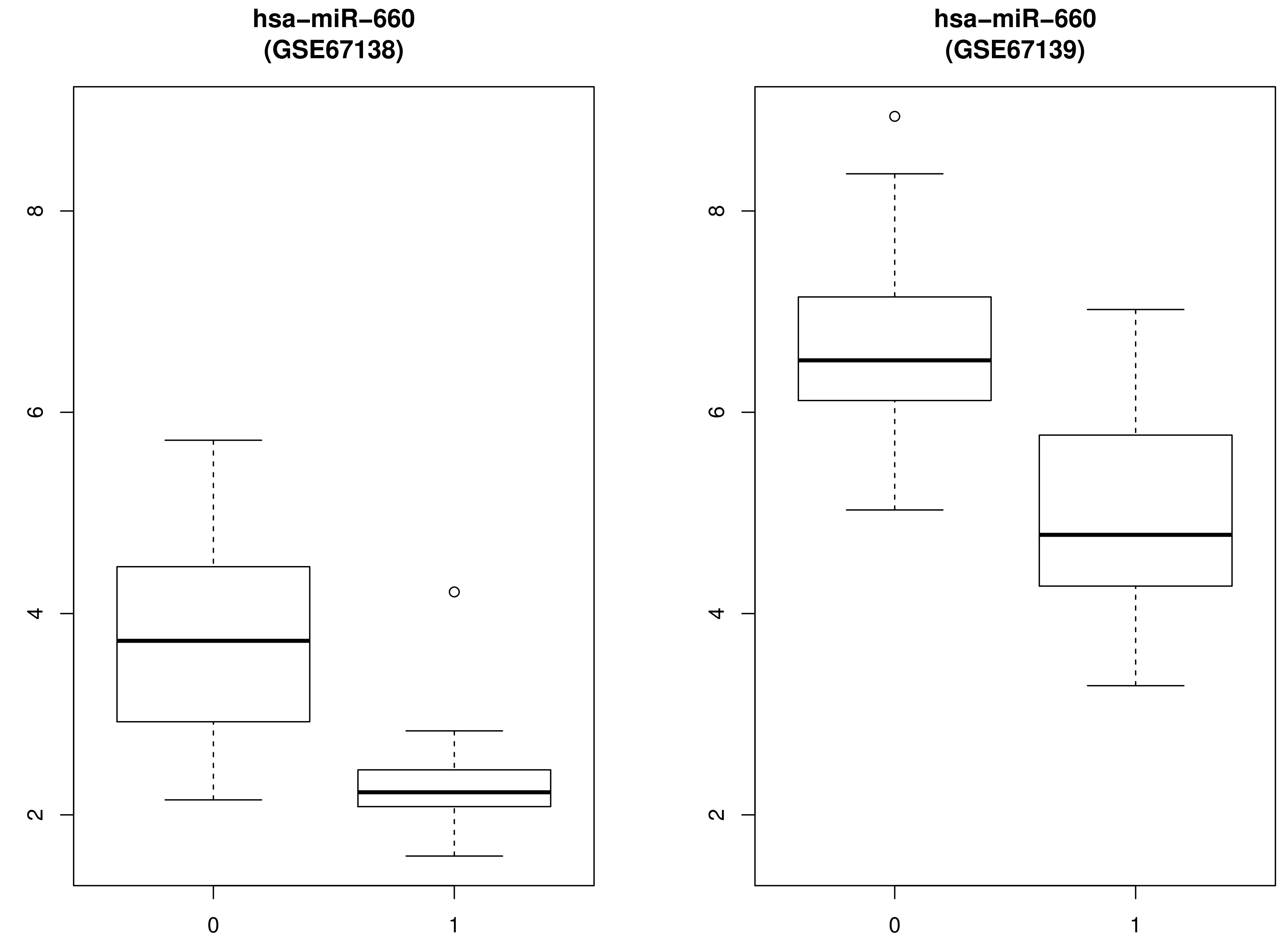
hsa−miR−660
(GSE67138)
hsa−miR−660
(GSE67139)
2
4
6
8
0
1

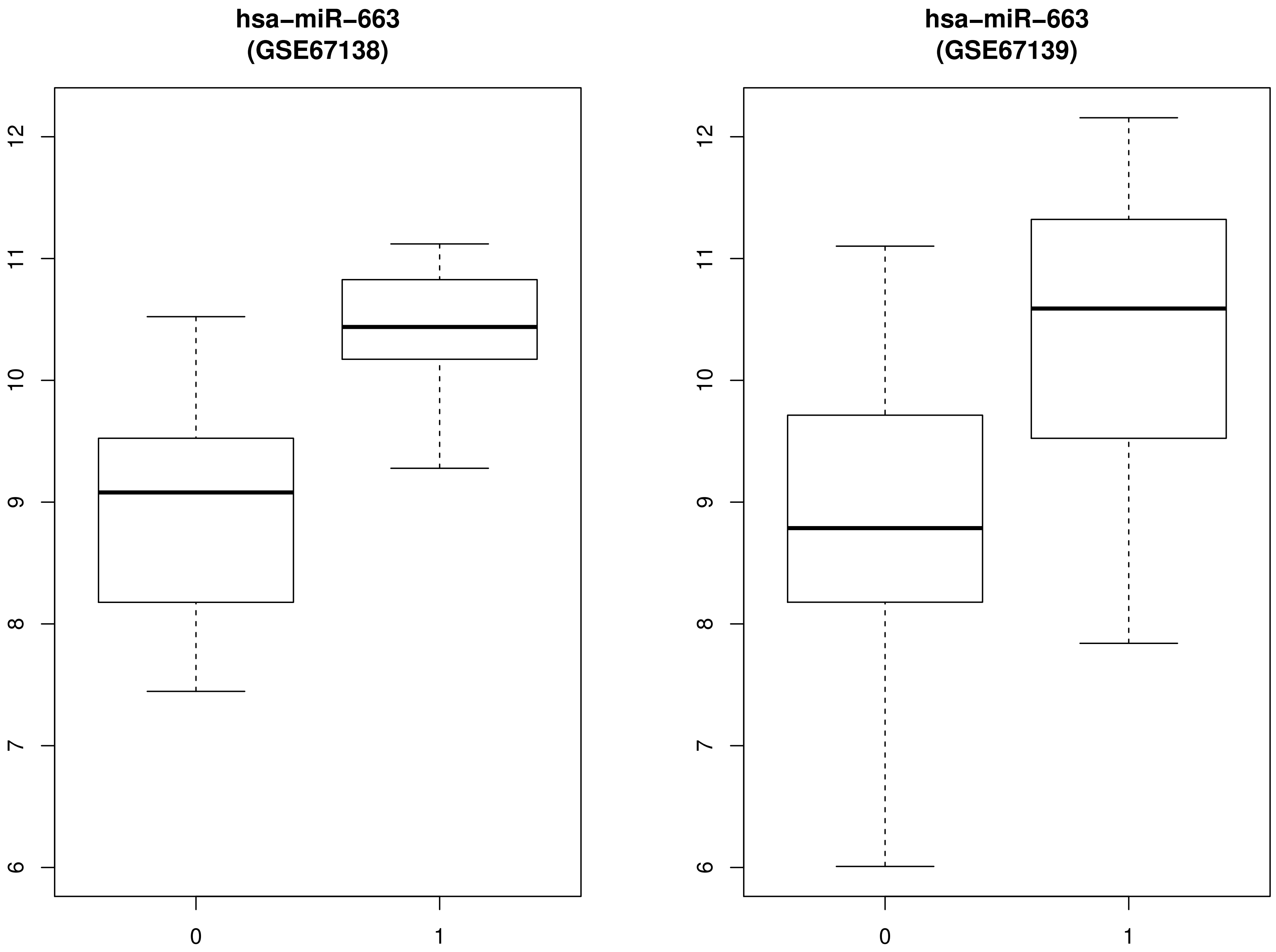
hsa−miR−663
(GSE67138)
hsa−miR−663
(GSE67139)
6
7
8
9
10
11
12
0
1

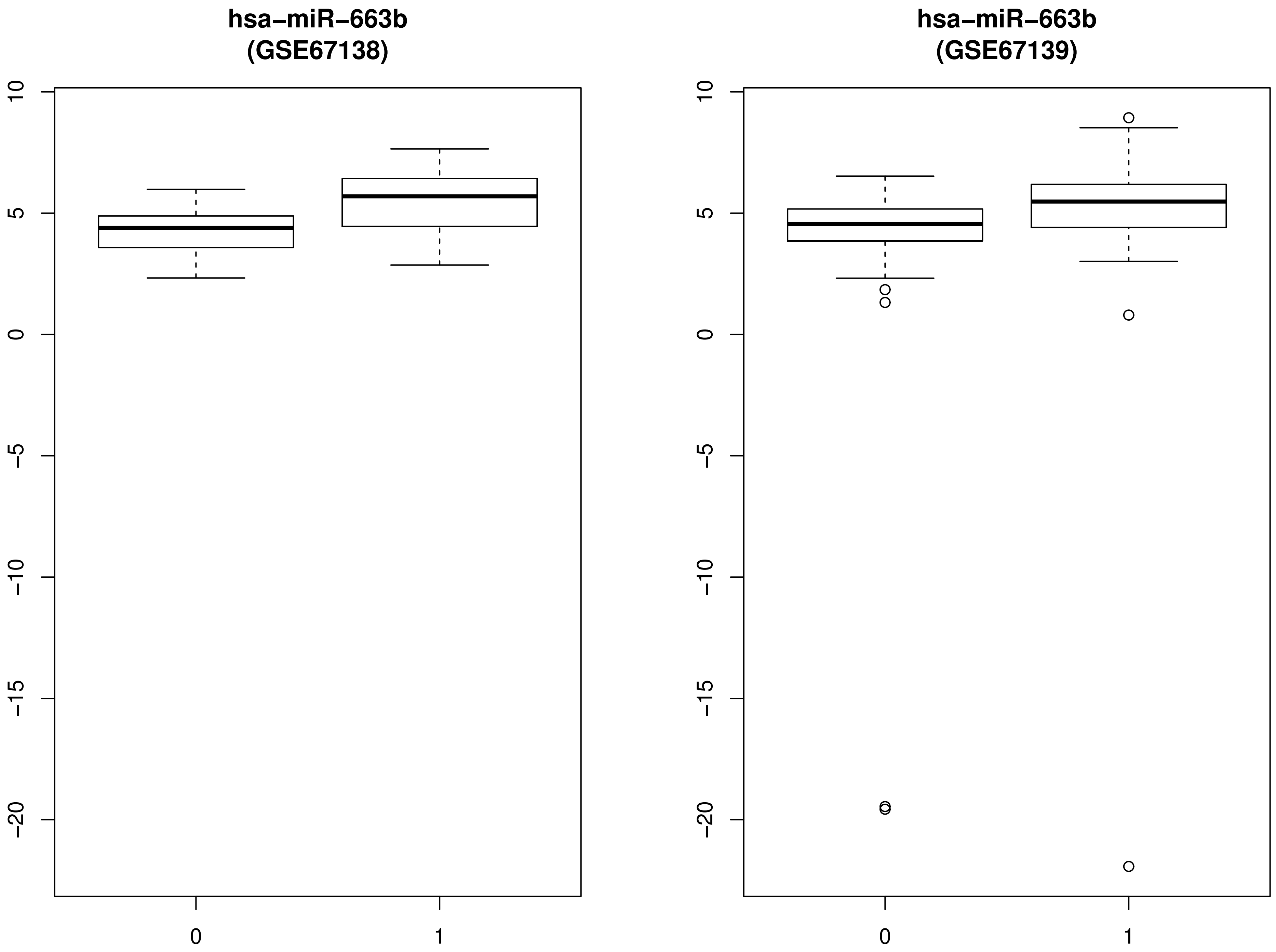
hsa−miR−663b
(GSE67138)
hsa−miR−663b
(GSE67139)
10
5
0
−5
−10
−15
−20
0
1

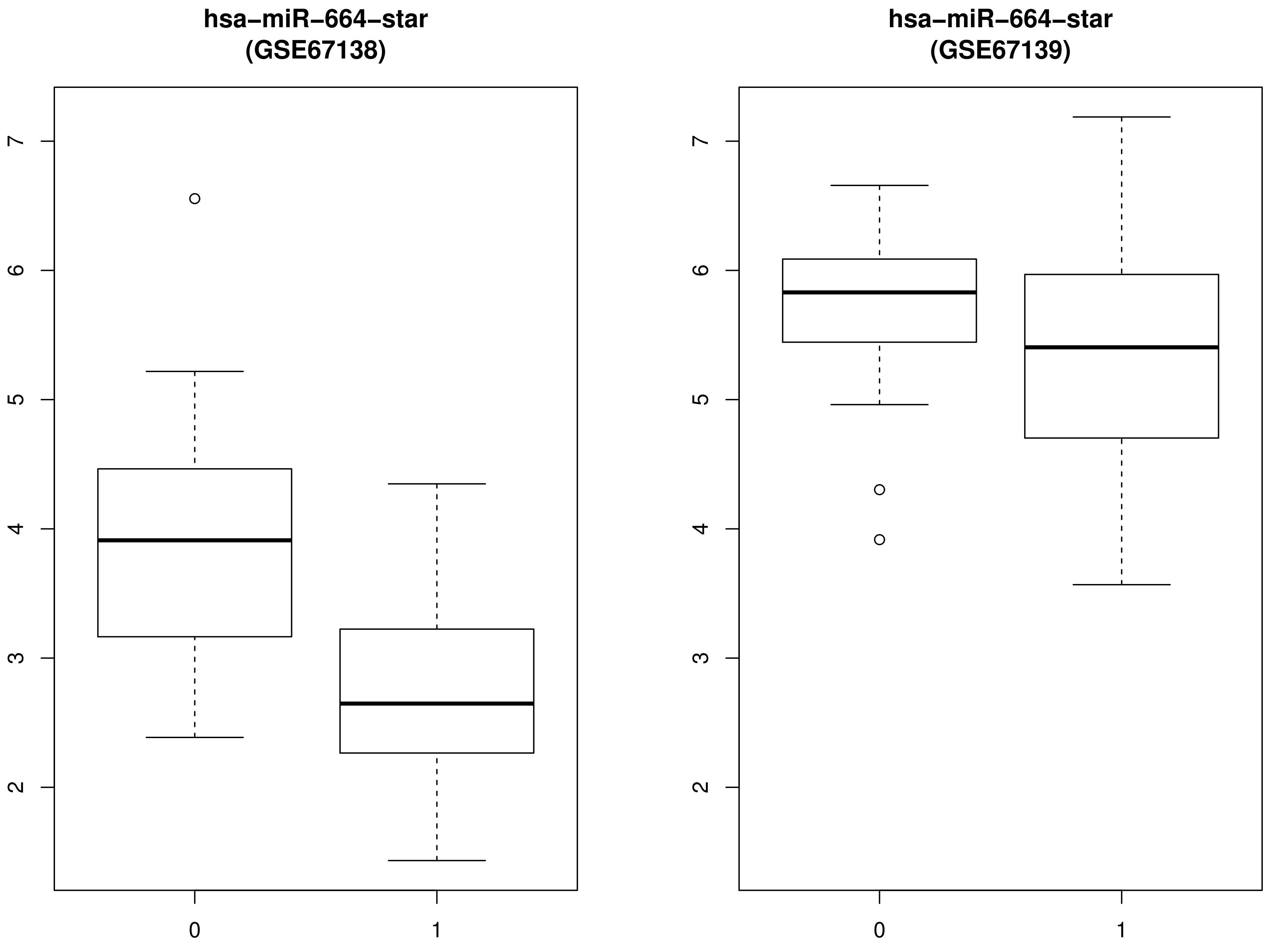
hsa−miR−664−star
(GSE67138)
hsa−miR−664−star
(GSE67139)
2
3
4
5
6
7
0
1

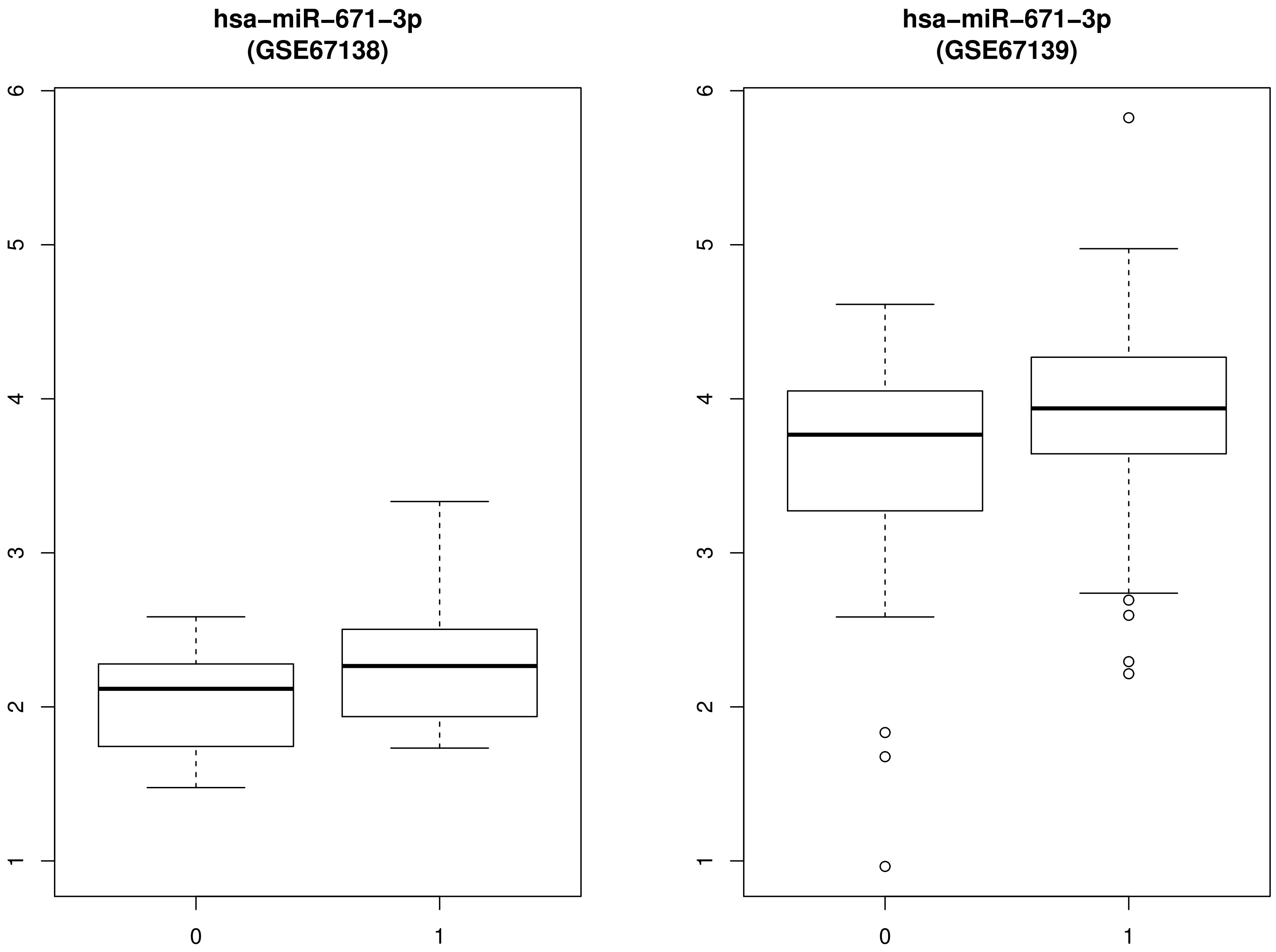
hsa−miR−671−3p
(GSE67138)
hsa−miR−671−3p
(GSE67139)
1
2
3
4
5
6
0
1

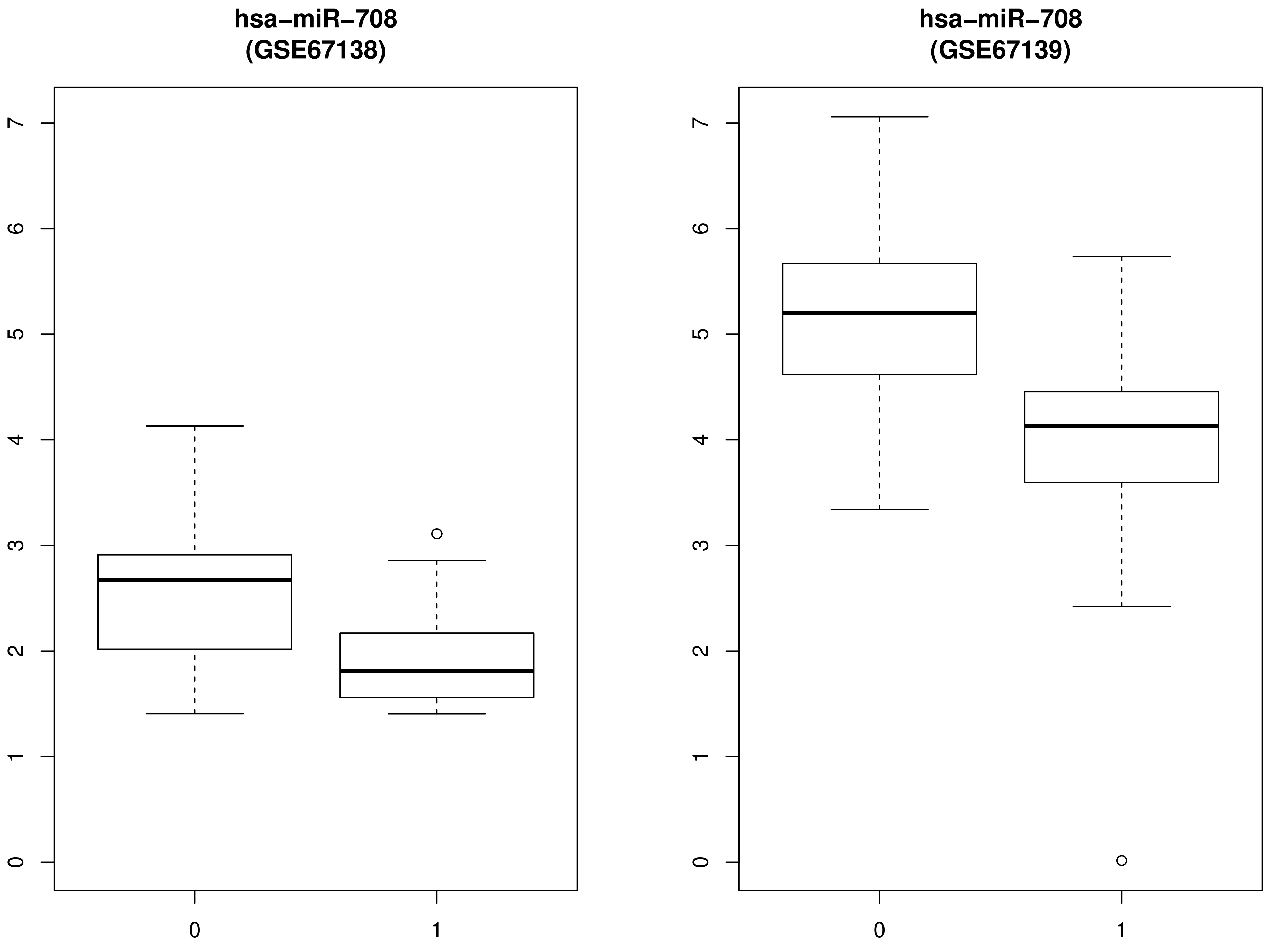
hsa−miR−708
(GSE67138)
hsa−miR−708
(GSE67139)
0
1
2
3
4
5
6
7
0
1

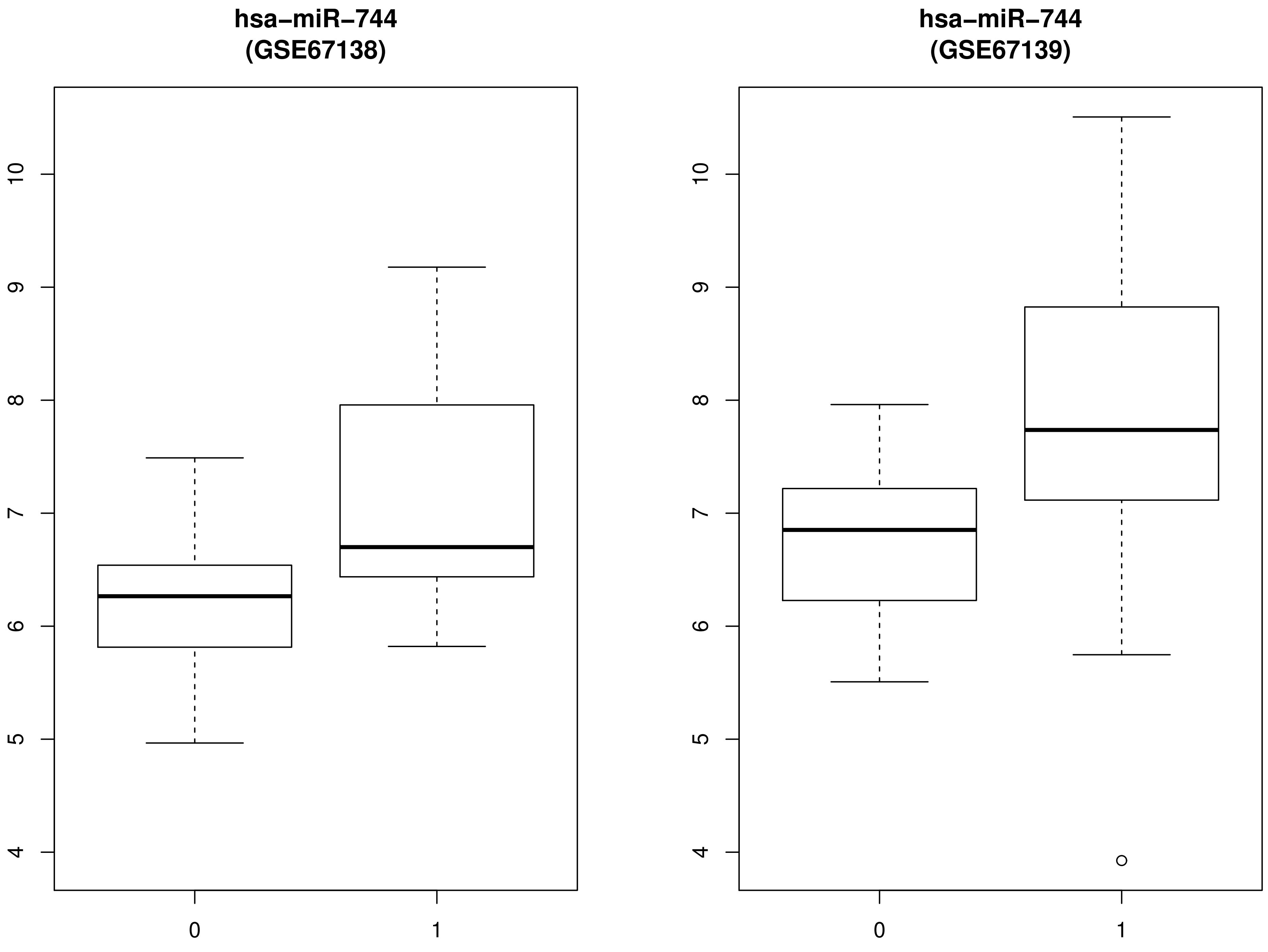
hsa−miR−744
(GSE67138)
hsa−miR−744
(GSE67139)
4
5
6
7
8
9
10
0
1

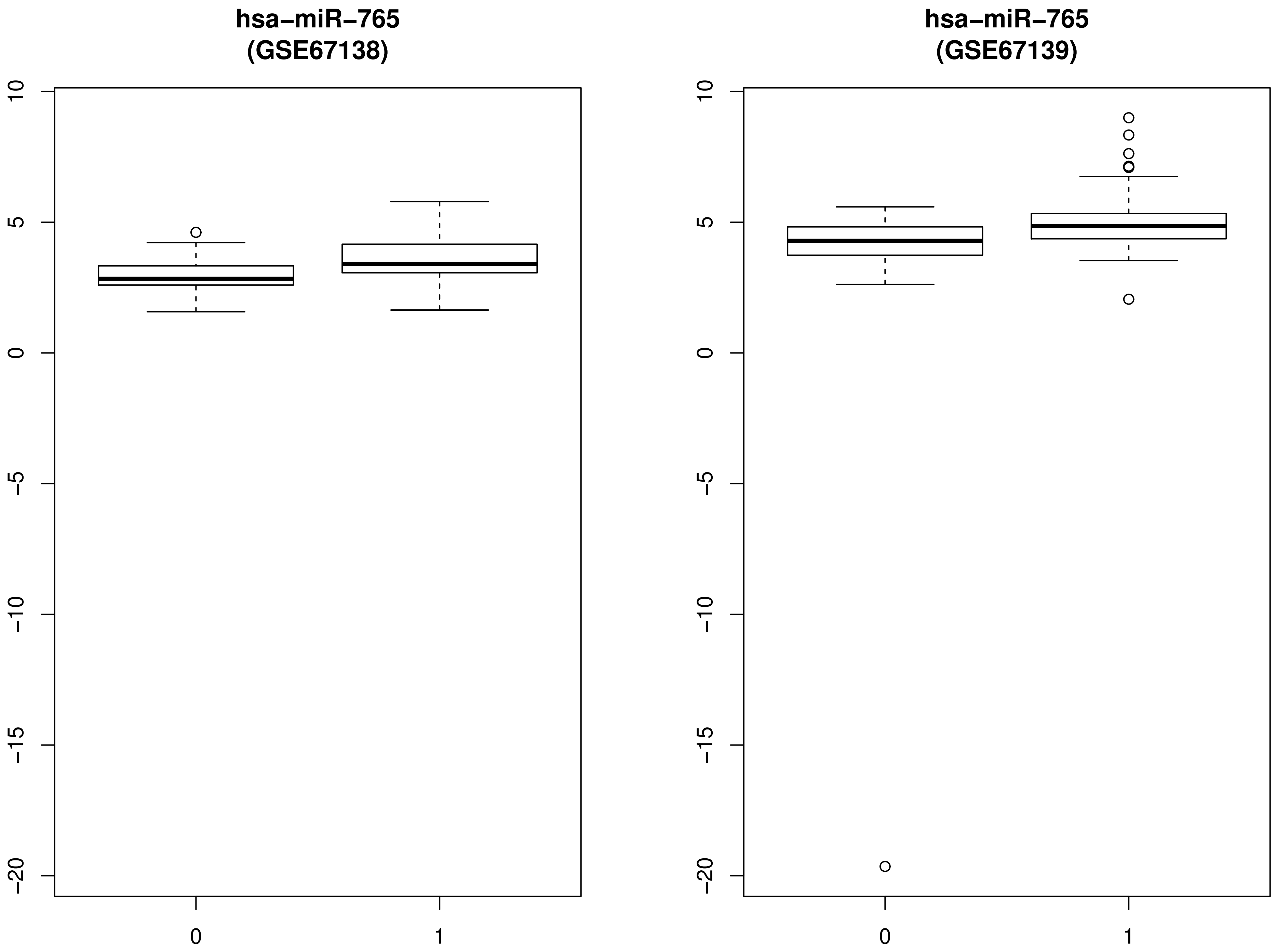

hsa−miR−765
(GSE67138)
hsa−miR−765
(GSE67139)
10
5
0
−5
−10
−15
−20
0
1

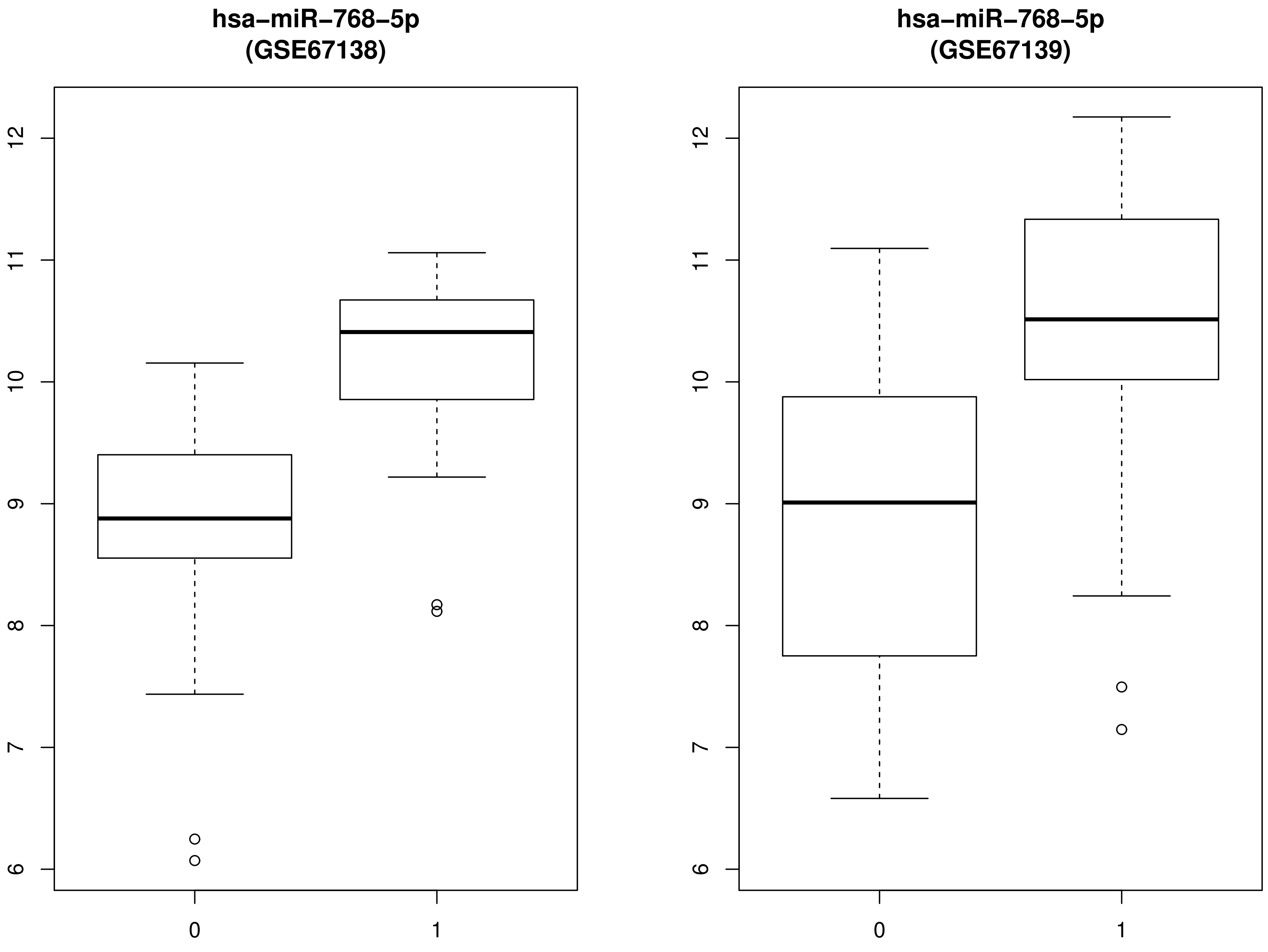
hsa−miR−768−5p
(GSE67138)
hsa−miR−768−5p
(GSE67139)
6
7
8
9
10
11
12
0
1

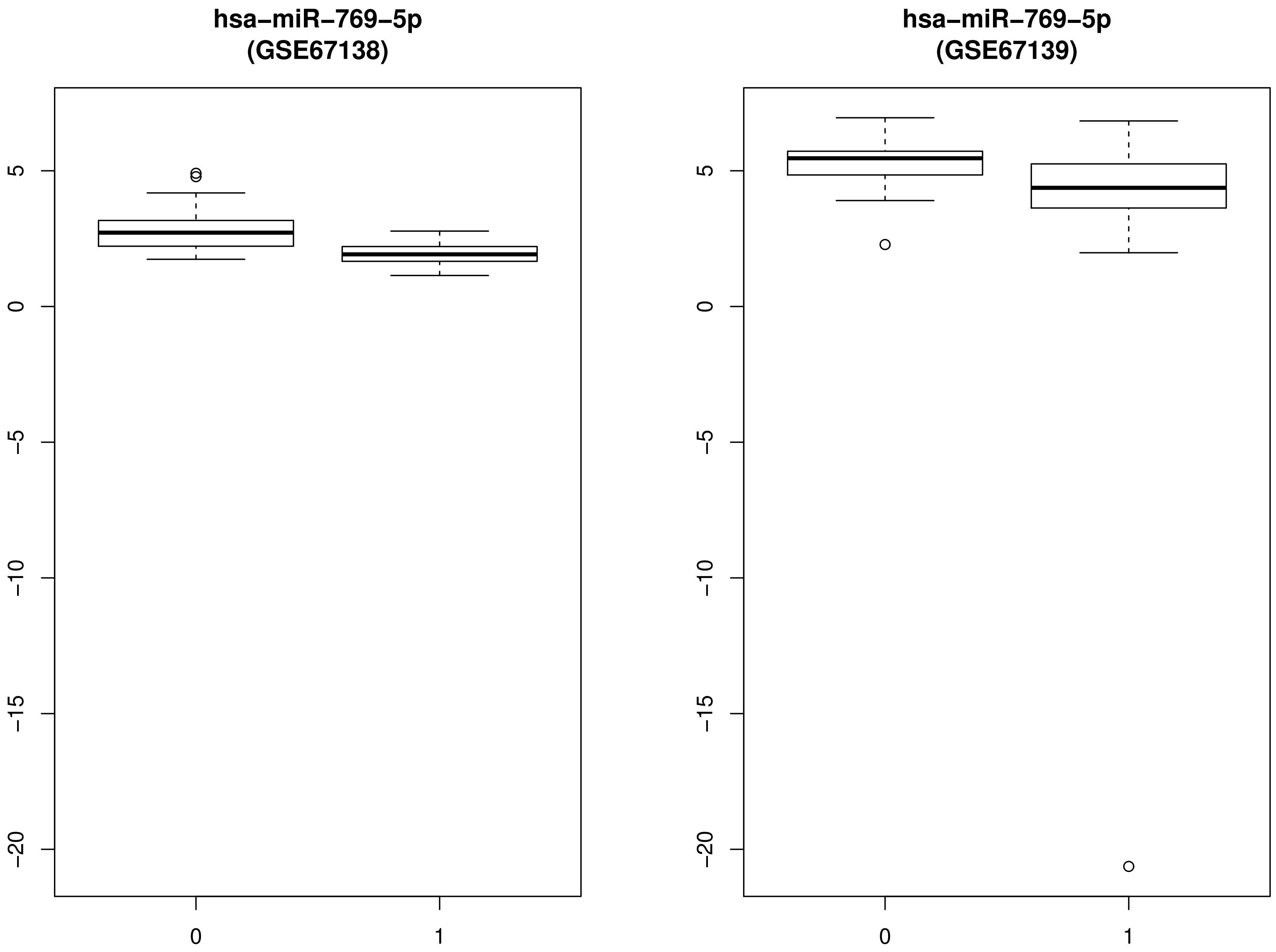
hsa−miR−769−5p
(GSE67138)
hsa−miR−769−5p
(GSE67139)
5
0
−5
−10
−15
−20
0
1

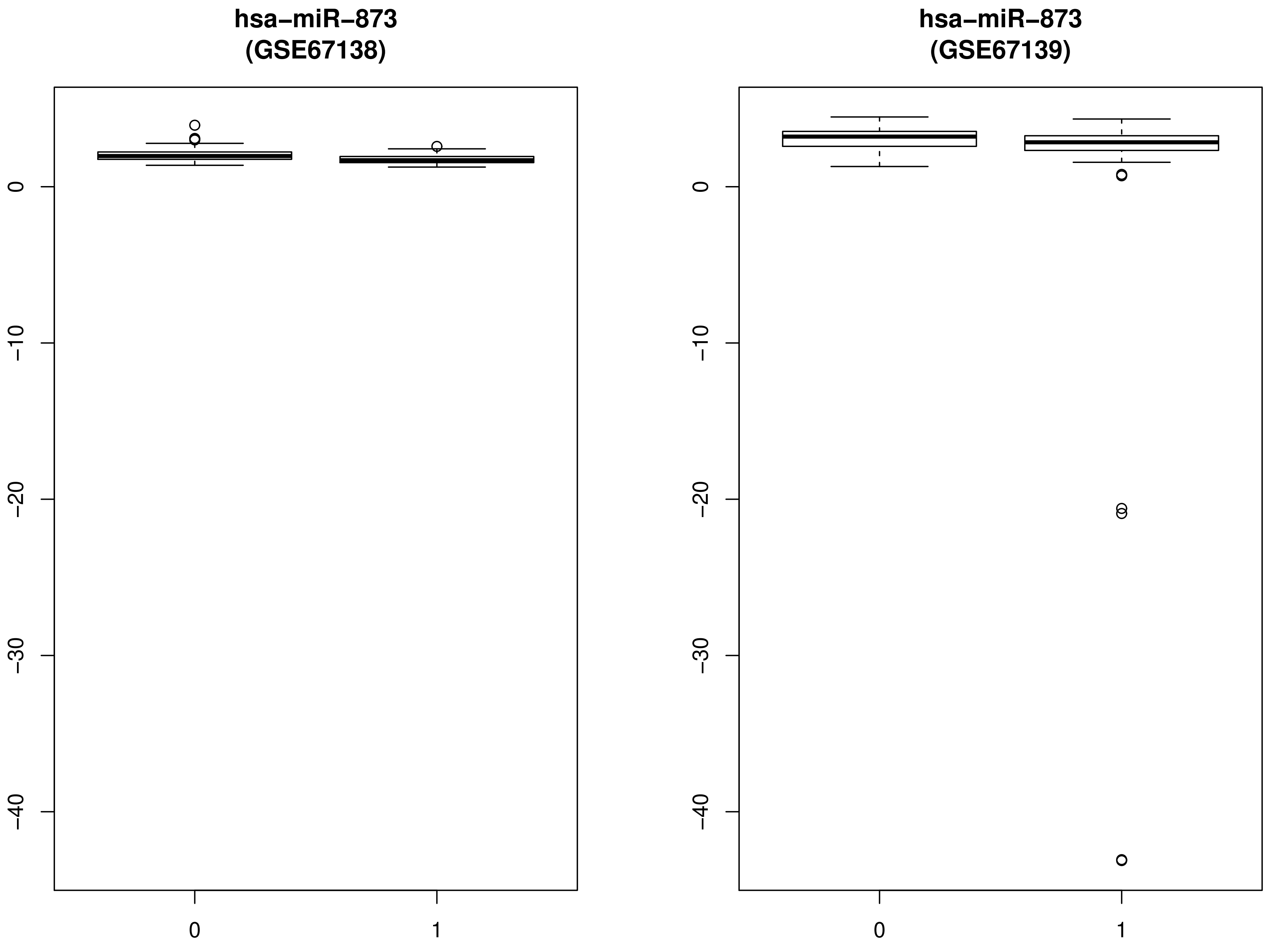
hsa−miR−873
(GSE67138)
hsa−miR−873
(GSE67139)
0
−10
−20
−30
−40
0
1

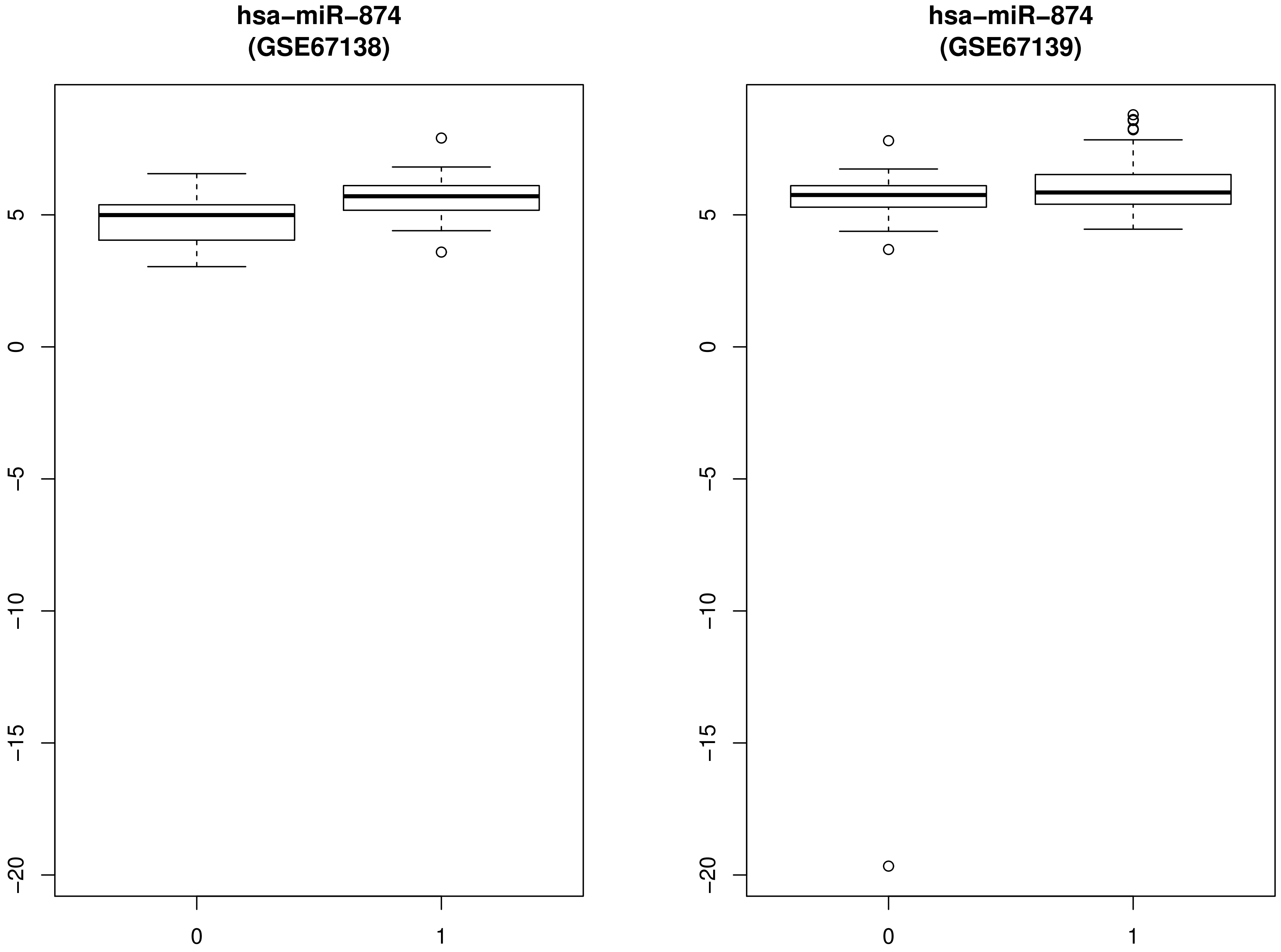
hsa−miR−874
(GSE67138)
hsa−miR−874
(GSE67139)
5
0
−5
−10
−15
−20
0
1

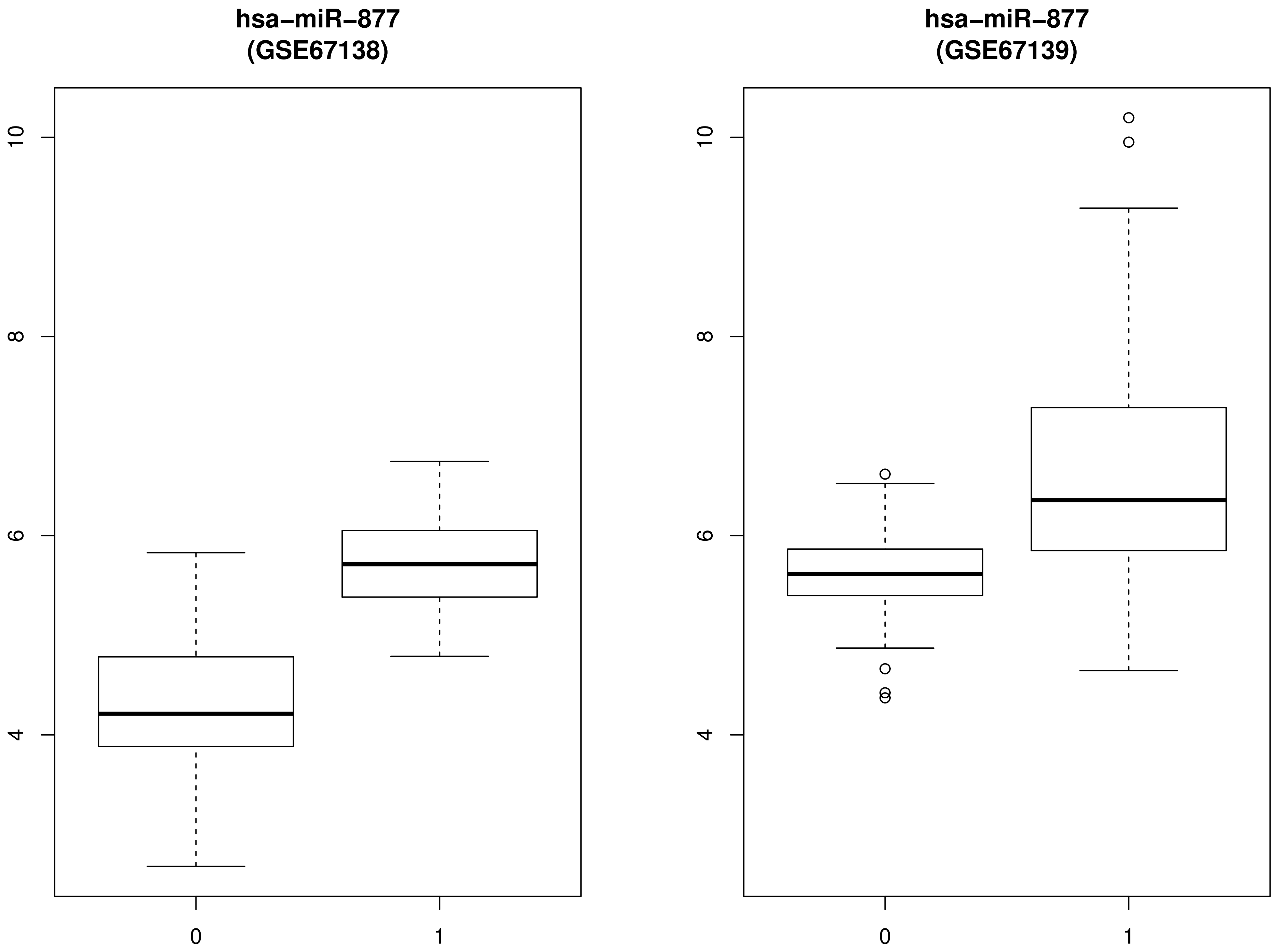
hsa−miR−877
(GSE67138)
hsa−miR−877
(GSE67139)
10
8
6
4
0
1

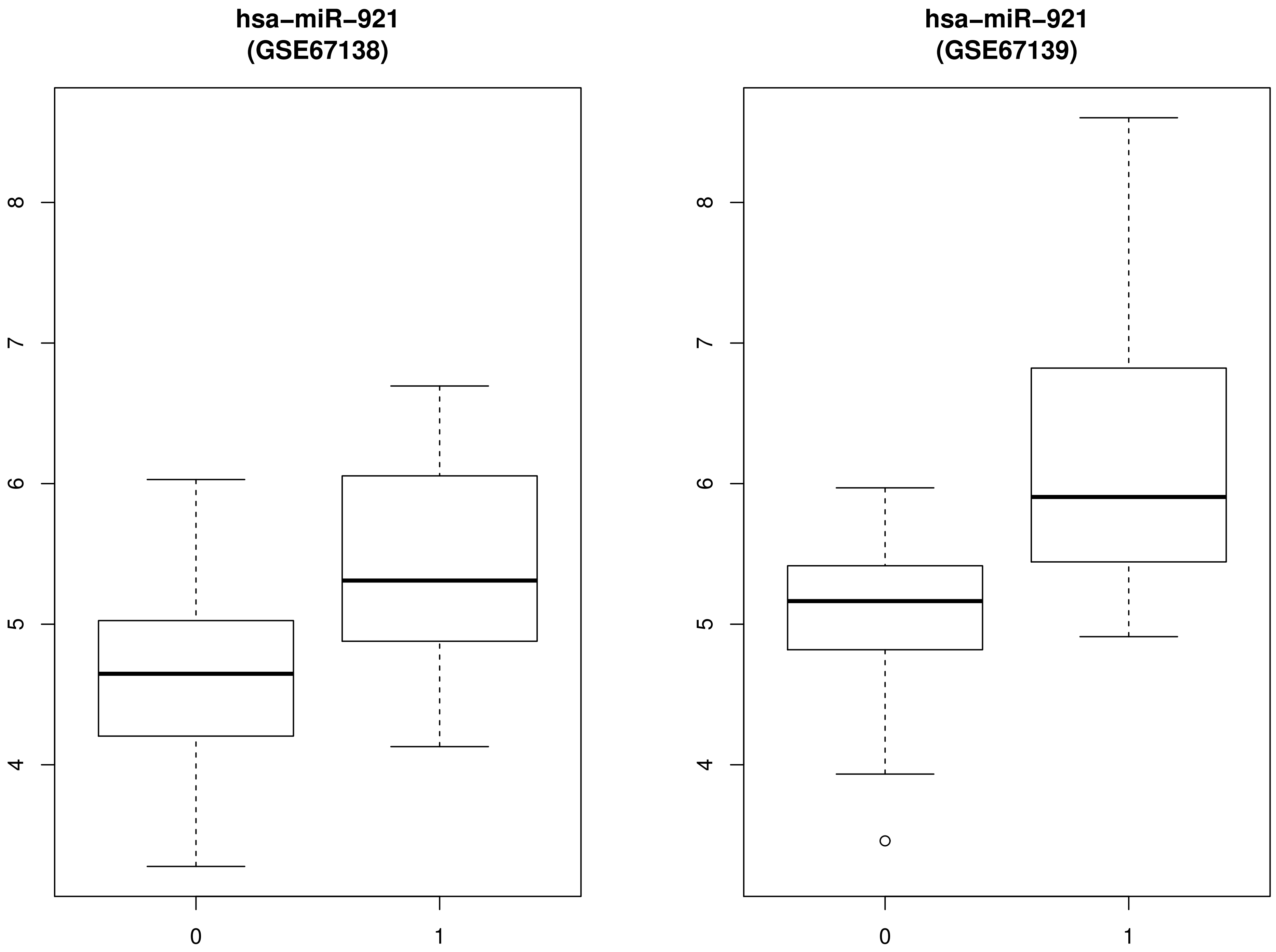
hsa−miR−921
(GSE67138)
hsa−miR−921
(GSE67139)
4
5
6
7
8
0
1
4
5
6
7
8
0
1

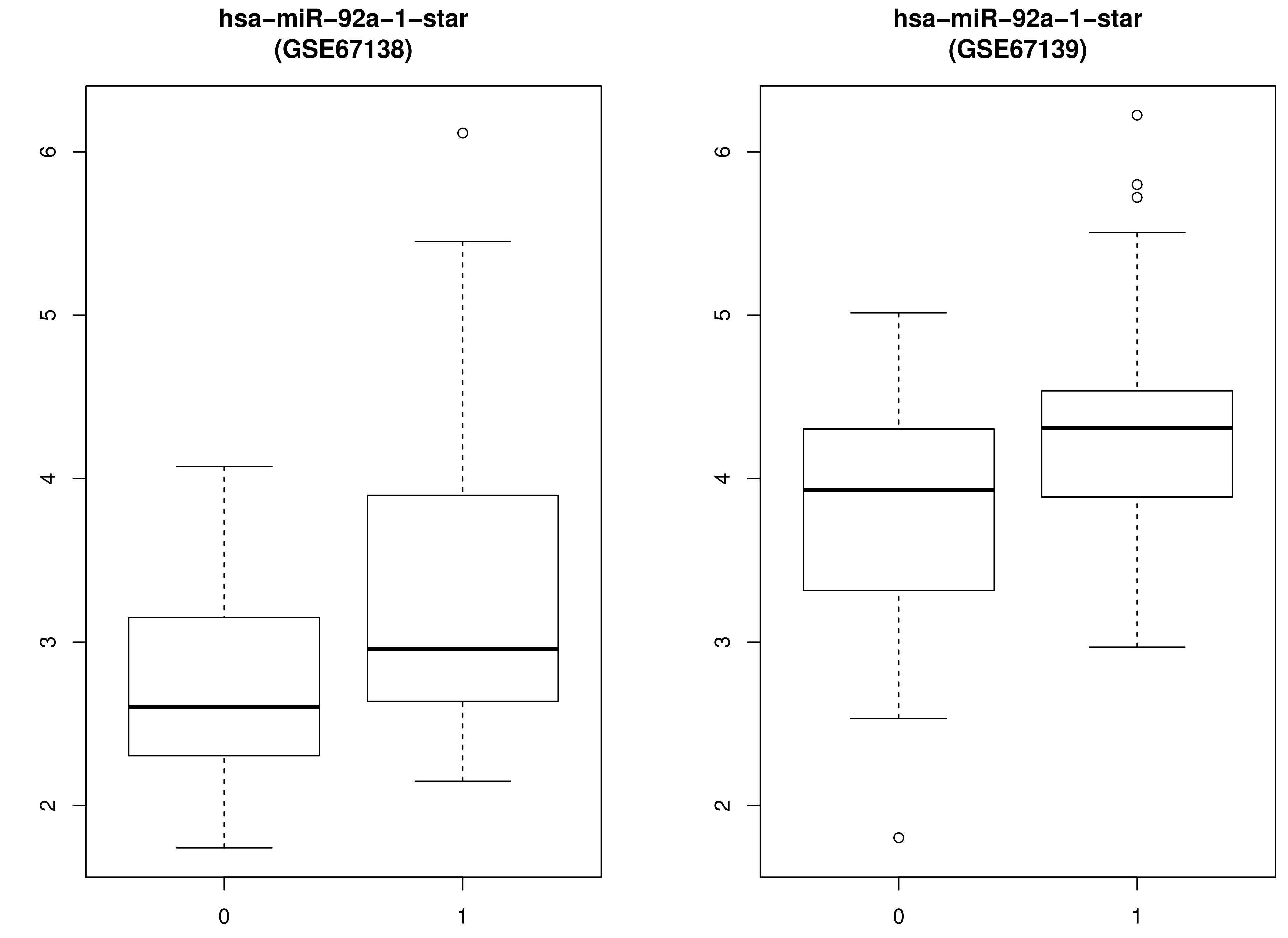
hsa−miR−92a−1−star
(GSE67138)
hsa−miR−92a−1−star
(GSE67139)
2
3
4
5
6
0
1

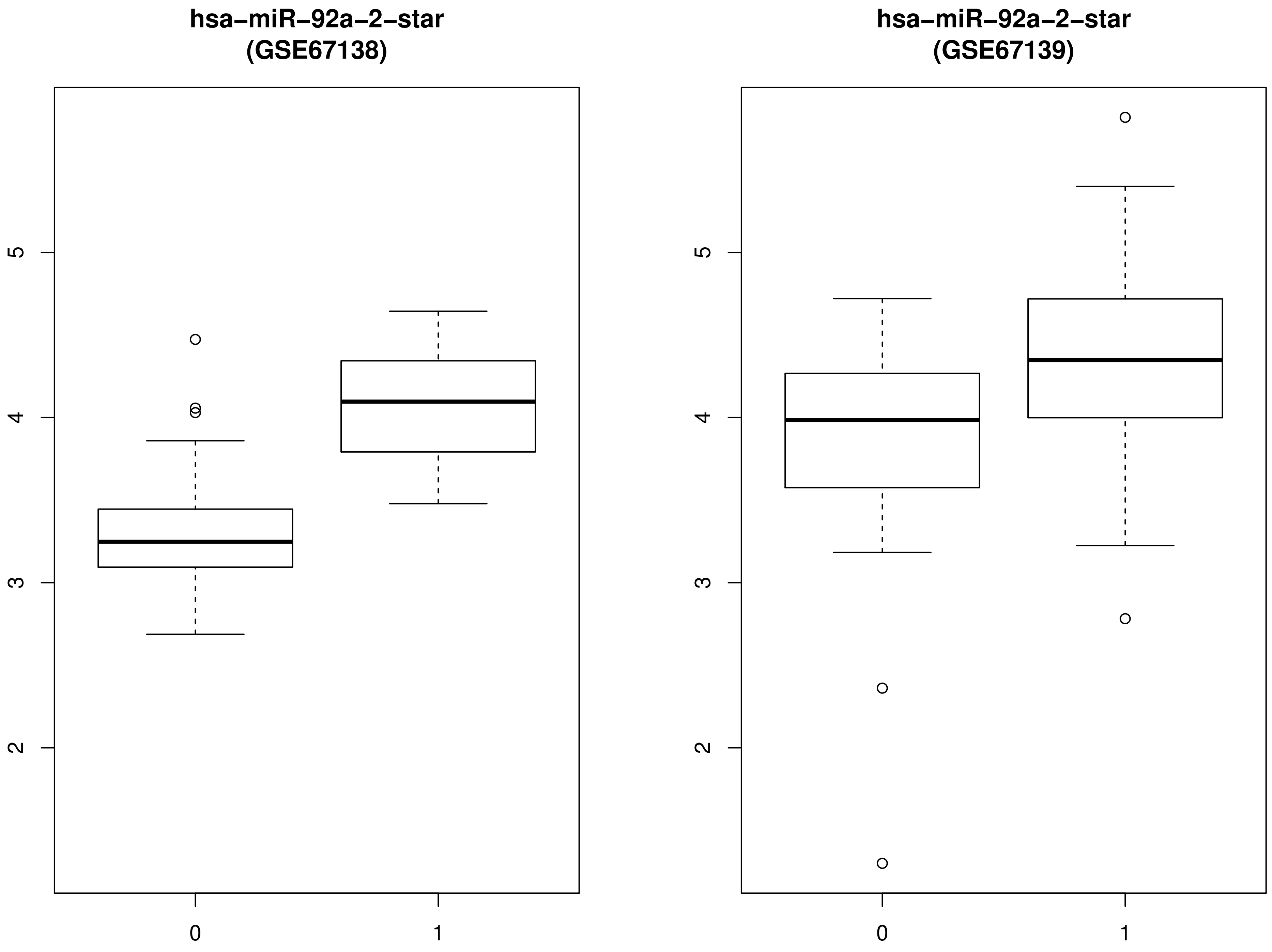
hsa−miR−92a−2−star
(GSE67138)
5
4
3
2
0
1
hsa−miR−92a−2−star
(GSE67139)
5
4
3
2
0
1

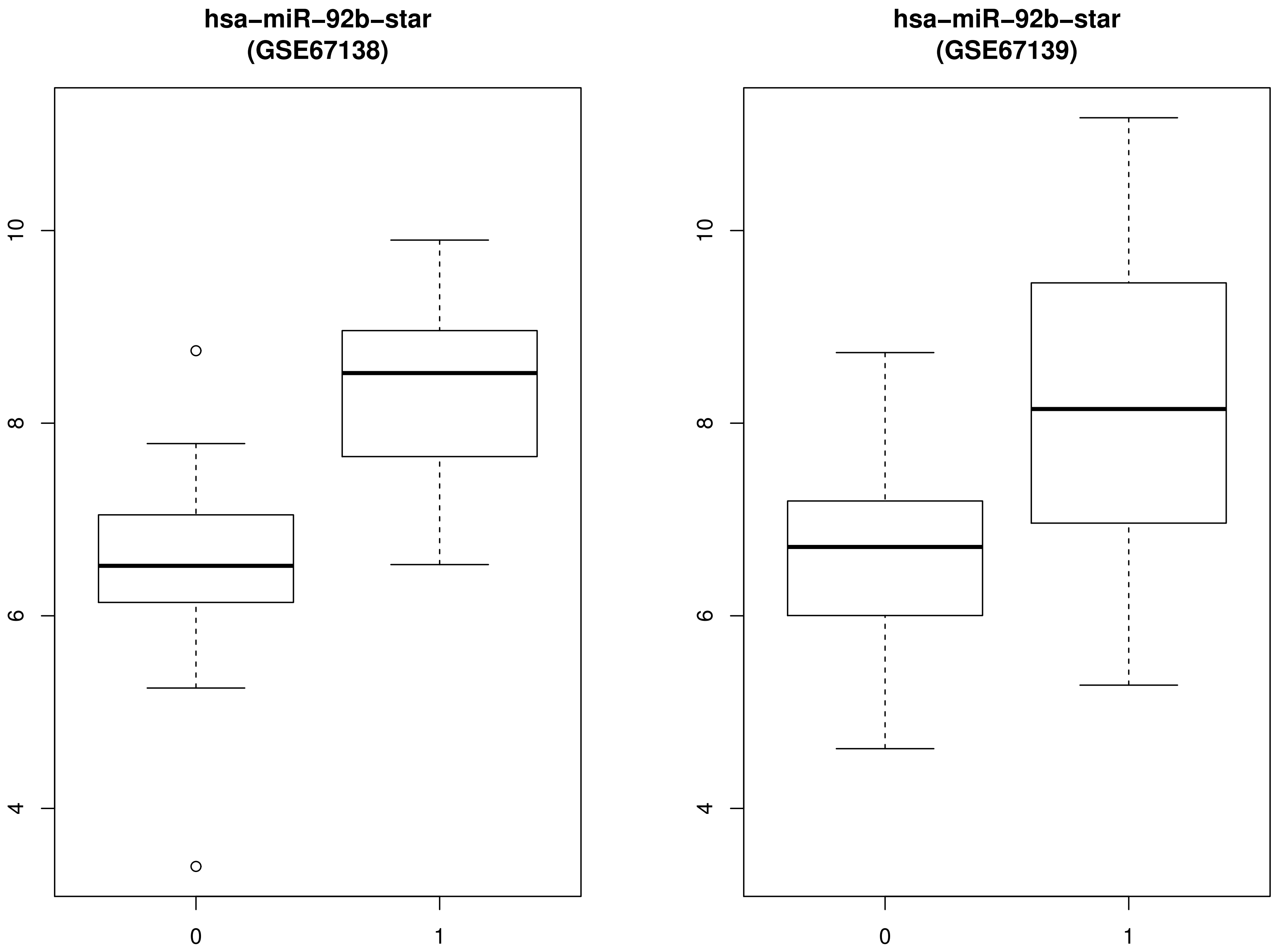
hsa–miR–92b–star
(GSE67138)
hsa–miR–92b–star
(GSE67139)
4
6
8
10
0
1

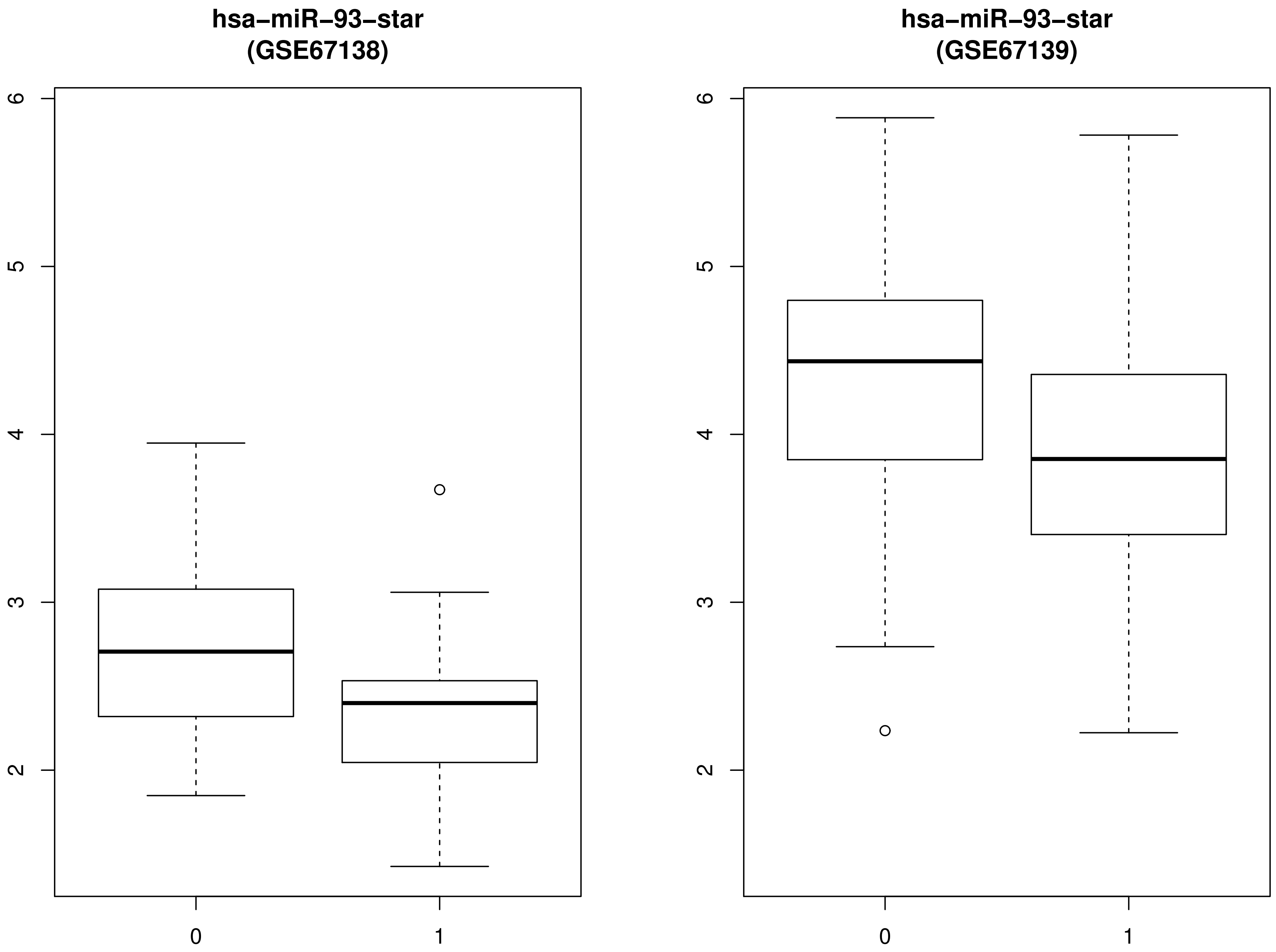

hsa−miR−93−star
(GSE67138)
6
5
4
3
2
0
1
hsa−miR−93−star
(GSE67139)
6
5
4
3
2
0
1

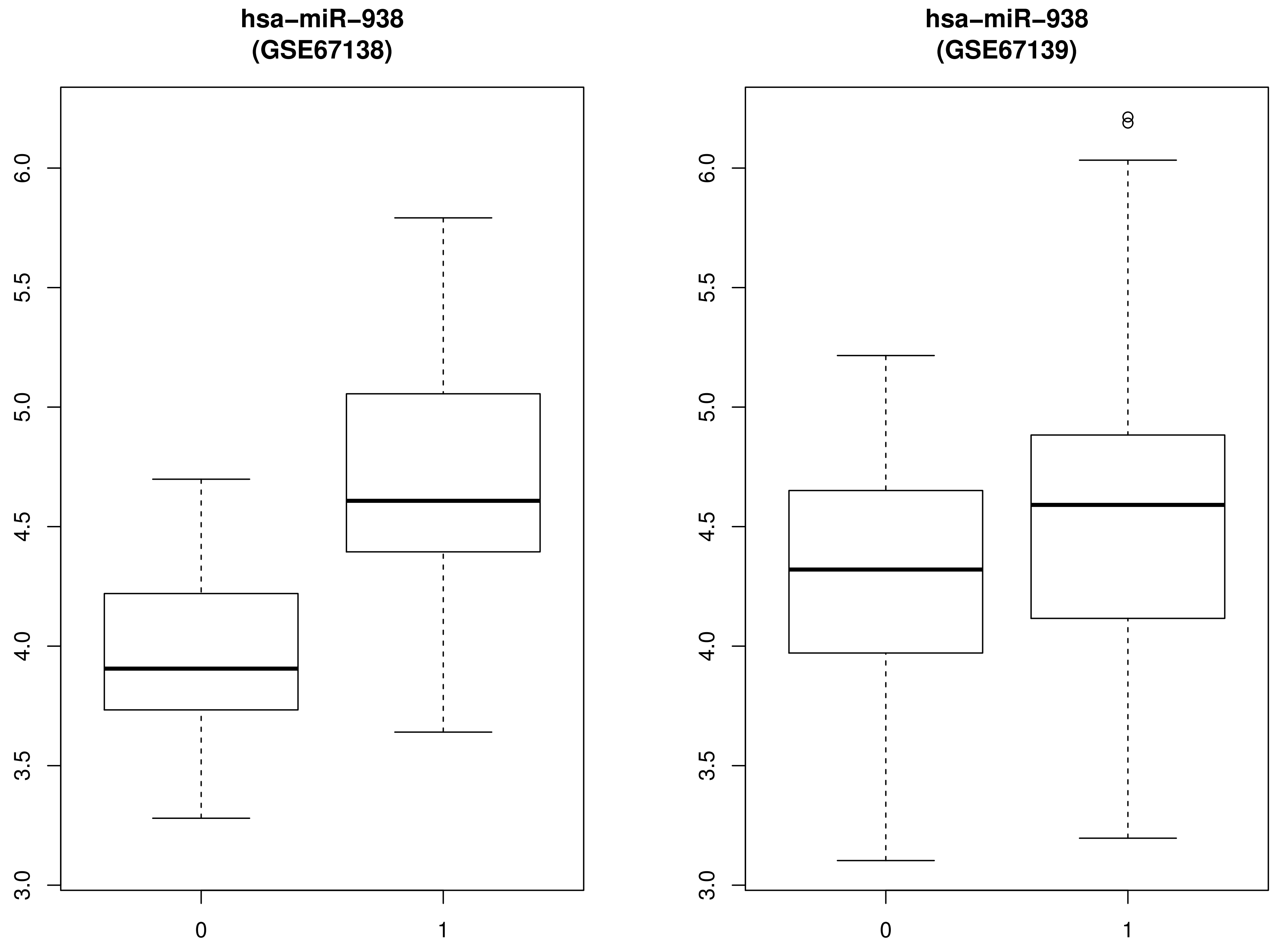
hsa−miR−938
(GSE67138)
hsa−miR−938
(GSE67139)
3.0
3.5
4.0
4.5
5.0
5.5
6.0
0
1

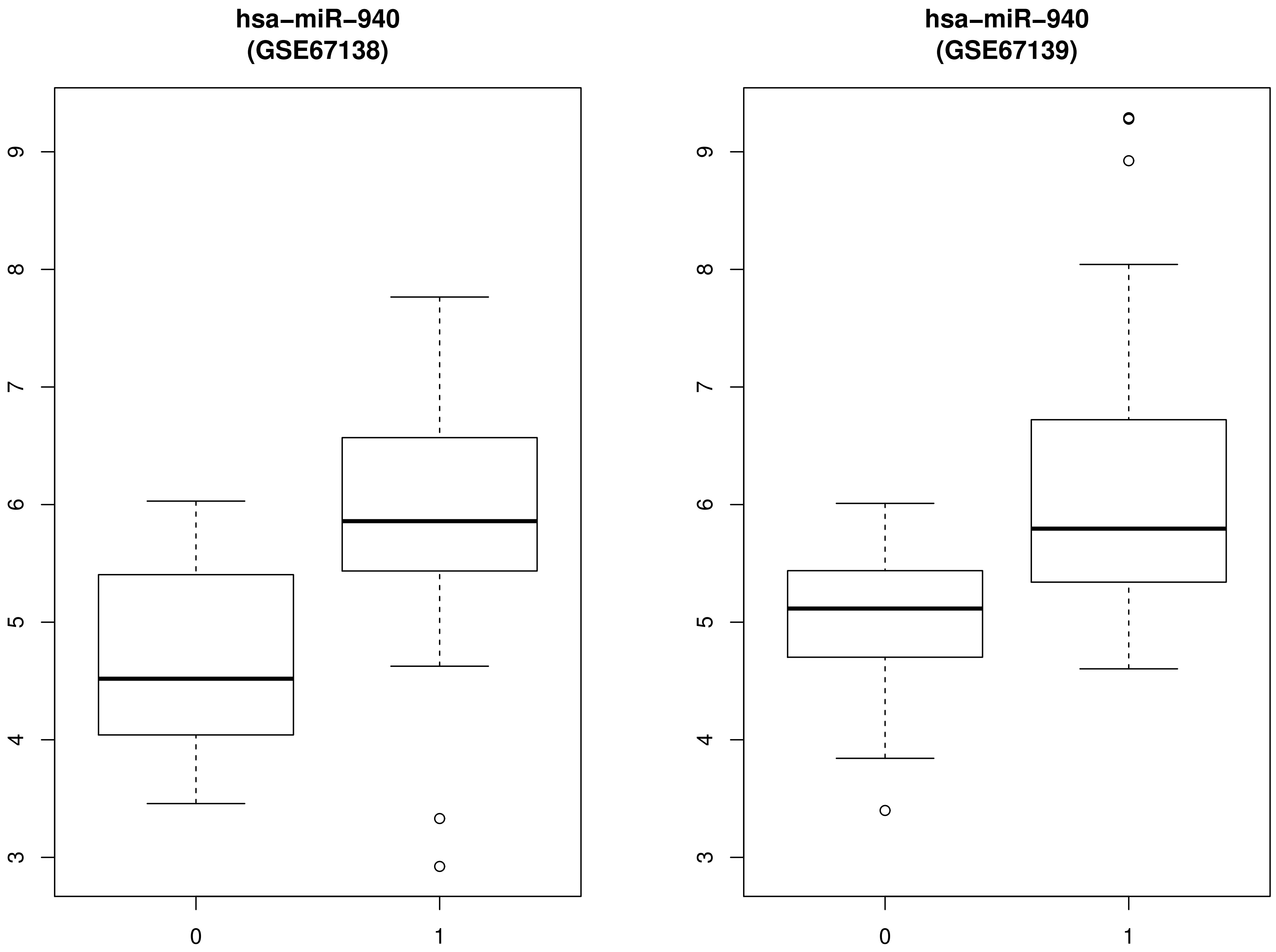
hsa−miR−940
(GSE67138)
hsa−miR−940
(GSE67139)
3
4
5
6
7
8
9
0
1

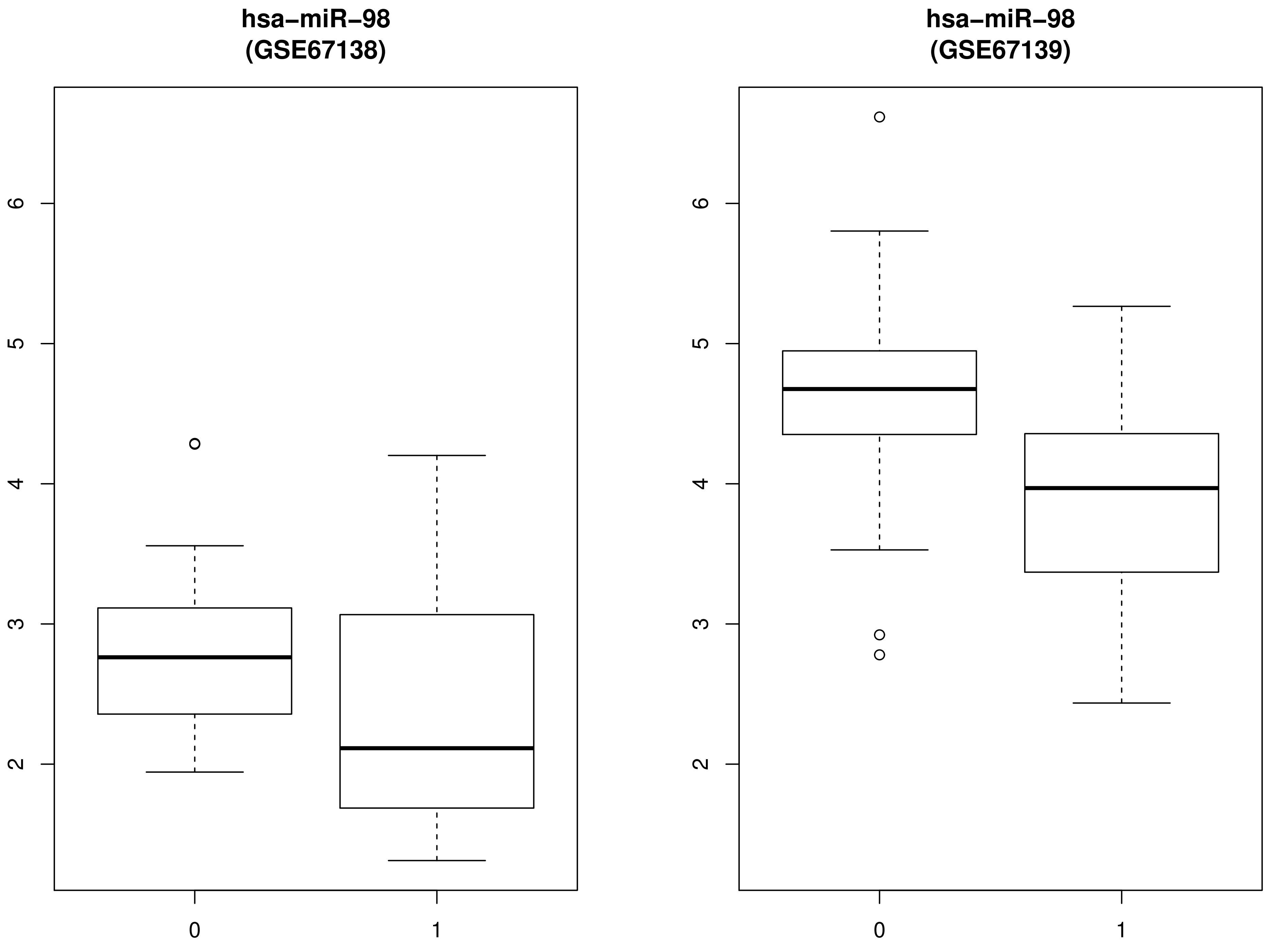
hsa−miR−98
(GSE67138)
hsa−miR−98
(GSE67139)
6
5
4
3
2
0
1
