# Supplementary material for: Detecting Differentially Variable MicroRNAs via Model-Based Clustering
Source: Int J Genomics. 2018 Jul 12;2018:6591634. doi: 10.1155/2018/6591634 (PMC6079429; doi:10.1155/2018/6591634)
Supplement: Supplementary Materials — Details about the gs model and its parameter estimation via the EM algorithm. Supplemental Table A1: the list of the 157 DE-only miRNAs. Supplemental Table A2: the list of the 60 DV-and-DE miRNAs. Supplemental Table A3: the list of the 1639 genes targeted by the 7 miRNAs in S DVonly. Supplemental Table A4: the list of the 8141 genes targeted by the 157 miRNAs in S DEonly. Supplemental Table A5: the list of the 6893 genes targeted by the 60 miRNAs in S both. Supplemental Table A6: the list of the 6 KEGG pathways enriched by the 1639 genes targeted by the 7 miRNAs in S DVonly. Supplemental Table A7: the list of the 1 KEGG pathway enriched by the 8141 genes targeted by the 157 miRNAs in S DEonly. Supplemental Table A8: the list of the 2 KEGG pathways enriched by the 6893 genes targeted by the 60 miRNAs in S both. Supplemental Table A9: p values of two-sided Wilcoxon signed rank tests to test if the median Jaccard index obtained by the gs method is the same as that obtained by each of the other 16 methods. Supplemental Table A10: p values of two-sided Wilcoxon signed rank tests to test if the median FPR obtained by the gs method is the same as that obtained by each of the other 16 methods. Supplemental Table A11: p values of two-sided Wilcoxon signed rank tests to test if the median FNR obtained by the gs method is the same as that obtained by each of the other 16 methods. Supplemental Table A12: p values of two-sided Wilcoxon signed rank tests to test if the median proportion of validation obtained by the gs method is the same as that obtained by each of the other 16 methods based on 100 bootstrap samples. Figure A1: the plot of percentiles of log2 expression levels across arrays. Left panel: GSE67138. Right panel: GSE67139. Figure A2: the plot of the first and second principal components. Left panel: GSE67138. Right panel: GSE67139. Supplemental Figure A3: parallel boxplots of the 7 validated DV-only miRNAs. Left panel: GSE67138. Right panel: GSE67139. Supplemental Fi [file 6591634.f1.zip › suppl_document_MMDvariance3cat_IJG_2364374.pdf]

# Supplementary Documents

---

# Contents


**A The gs model** ii

**B Re-parameterization of the correlation** iv

**C log density for genes in cluster 1** iv

**D log density for genes in cluster 2** v

**E log density for genes in cluster 3** v

**F Parameter Estimation by EM Algorithm** v

**G E-step of the EM algorithm** vii

**H The M-step of the E-M algorithm for 3-mixture distribution** ix

H.1 The first derivative of $\partial \log [f_1(\boldsymbol{x}_i|\boldsymbol{\theta}_1)] / \partial s_{c_1}$ . . . . . . . . . . x
H.2 The first derivative of $\partial \log [f_1(\boldsymbol{x}_i|\boldsymbol{\theta}_1)] / \partial \triangle_{n_1}$ . . . . . . . . . . x
H.3 The first derivative of $\partial \log [f_1(\boldsymbol{x}_i|\boldsymbol{\theta}_1)] / \partial r_{c_1}$ . . . . . . . . . . xi
H.4 The first derivative of $\partial \log [f_1(\boldsymbol{x}_i|\boldsymbol{\theta}_1)] / \partial r_{n_1}$ . . . . . . . . . . xi
H.5 The first derivative of $\partial \log [f_1(\boldsymbol{x}_i|\boldsymbol{\theta}_1)] / \partial \mu_{c_1}$ . . . . . . . . . . xii
H.6 The first derivative of $\partial \log [f_1(\boldsymbol{x}_i|\boldsymbol{\theta}_1)] / \partial \mu_{n_1}$ . . . . . . . . . . xii
H.7 The first derivative of $\partial \log [f_2(\boldsymbol{x}_i|\boldsymbol{\theta}_2)] / \partial s_2$ . . . . . . . . . . xii
H.8 The first derivative of $\partial \log [f_2(\boldsymbol{x}_i|\boldsymbol{\theta}_2)] / \partial r_{c_2}$ . . . . . . . . . . xii
H.9 The first derivative of $\partial \log [f_2(\boldsymbol{x}_i|\boldsymbol{\theta}_2)] / \partial r_{n_2}$ . . . . . . . . . . xiii
H.10 The first derivative of $\partial \log [f_2(\boldsymbol{x}_i|\boldsymbol{\theta}_2)] / \partial \mu_{c_2}$ . . . . . . . . . . xiii
H.11 The first derivative of $\partial \log [f_2(\boldsymbol{x}_i|\boldsymbol{\theta}_2)] / \partial \mu_{n_2}$ . . . . . . . . . . xiii
H.12 The first derivative of $\partial \log [f_3(\boldsymbol{x}_i|\boldsymbol{\theta}_3)] / \partial s_{c_3}$ . . . . . . . . . . xiv
H.13 The first derivative of $\partial \log [f_3(\boldsymbol{x}_i|\boldsymbol{\theta}_3)] / \partial \triangle_{n_3}$ . . . . . . . . . . xiv
H.14 The first derivative of $\partial \log [f_3(\boldsymbol{x}_i|\boldsymbol{\theta}_3)] / \partial r_{c_3}$ . . . . . . . . . . xv
H.15 The first derivative of $\partial \log [f_3(\boldsymbol{x}_i|\boldsymbol{\theta}_3)] / \partial r_{n_3}$ . . . . . . . . . . xv
H.16 The first derivative of $\partial \log [f_3(\boldsymbol{x}_i|\boldsymbol{\theta}_3)] / \partial \mu_{c_3}$ . . . . . . . . . . xv
H.17 The first derivative of $\partial \log [f_3(\boldsymbol{x}_i|\boldsymbol{\theta}_3)] / \partial \mu_{n_3}$ . . . . . . . . . . xv
H.18 The first derivatives of $Q_\ell$ . . . . . . . . . . . . . . . . . . . . . xvi
H.19 The first derivative of $\partial Q_1 / \partial s_{c_1}$ . . . . . . . . . . . . . . . . . . xvi

H.20 The first derivative of $\partial Q_1/\partial\triangle_{n_1}$ . . . . . . . . . . . . . . . . . . xvi
H.21 The first derivative of $\partial Q_1/\partial r_{c_1}$ . . . . . . . . . . . . . . . . . . . xvi
H.22 The first derivative of $\partial Q_1/\partial r_{n_1}$ . . . . . . . . . . . . . . . . . . . xvii
H.23 The first derivative of $\partial Q_1/\partial \mu_{c_1}$ . . . . . . . . . . . . . . . . . . . xvii
H.24 The first derivative of $\partial Q_1/\partial \mu_{n_1}$ . . . . . . . . . . . . . . . . . . . xvii
H.25 The first derivative of $\partial Q_2/\partial s_2$ . . . . . . . . . . . . . . . . . . . xvii
H.26 The first derivative of $\partial Q_2/\partial r_{c_2}$ . . . . . . . . . . . . . . . . . . . xviii
H.27 The first derivative of $\partial Q_2/\partial r_{n_2}$ . . . . . . . . . . . . . . . . . . . xviii
H.28 The first derivative of $\partial Q_2/\partial \mu_{c_2}$ . . . . . . . . . . . . . . . . . . . xviii
H.29 The first derivative of $\partial Q_2/\partial \mu_{n_2}$ . . . . . . . . . . . . . . . . . . . xviii
H.30 The first derivative of $\partial Q_3/\partial s_{c_3}$ . . . . . . . . . . . . . . . . . . . xix
H.31 The first derivative of $\partial Q_3/\partial\triangle_{n_3}$ . . . . . . . . . . . . . . . . . . xix
H.32 The first derivative of $\partial Q_3/\partial r_{c_3}$ . . . . . . . . . . . . . . . . . . . xix
H.33 The first derivative of $\partial Q_3/\partial r_{n_3}$ . . . . . . . . . . . . . . . . . . . xix
H.34 The first derivative of $\partial Q_3/\partial \mu_{c_3}$ . . . . . . . . . . . . . . . . . . . xx
H.35 The first derivative of $\partial Q_3/\partial \mu_{n_3}$ . . . . . . . . . . . . . . . . . . . xx

**I Log-density function for the multivariate normal distribution with special mean vector and covariance matrix xx**

# A The gs model

We can first consider a 3-cluster partition of gene probes via our marginal mixture distribution model:

- gene probes for cases have larger dispersion than those for controls
- gene probes for cases have the same dispersion as those for controls
- gene probes for cases have smaller dispersion than those for controls

Let $\boldsymbol{X} = (X_1, X_2, \ldots, X_{m_c}, X_{m_c+1}, X_{m_c+2}, \ldots, X_{m_c+m_n})^T$, a $m \times 1$ vector, be the *transformed* gene profile for a randomly selected gene over $m$ tissue samples ($m = m_c + m_n$, where $m_c$ is the number of abnormal tissue samples and $m_n$ normal tissue samples). We assume that data have been normalized to remove the effects of confounding factors, such as dye effect, chip effect, batch effect, etc.. The distribution of $\boldsymbol{X}$ is assumed a three-component mixture of multivariate Normal distributions with marginal density:

$$\begin{aligned} &f(\boldsymbol{x}|\boldsymbol{\theta}_1, \boldsymbol{\theta}_2, \boldsymbol{\theta}_3) = \pi_1 f_1(\boldsymbol{x}|\boldsymbol{\theta}_1) + \pi_2 f_2(\boldsymbol{x}|\boldsymbol{\theta}_2) + \pi_3 f_3(\boldsymbol{x}|\boldsymbol{\theta}_3), \\ &\pi_1 + \pi_2 + \pi_3 = 1, \pi_i > 0, i = 1, 2, 3, \end{aligned} \tag{A1}$$

where $\pi_1$, $\pi_2$, $\pi_3$ are mixture proportions. The $m \times 1$ vector $\boldsymbol{x}$ is a realization of the random vector $\boldsymbol{X}$; $\boldsymbol{\theta}_k$, is the parameter set for the $k$-th component distribution $f_k$, $k = 1, 2, 3$; and $f_1$, $f_2$, and $f_3$ are the density functions for multivariate Normal distributions with the mean vectors

$$\boldsymbol{\mu}_1 = \begin{pmatrix} \mu_{c1}\mathbf{1}_{m_c} \\ \mu_{n1}\mathbf{1}_{m_n} \end{pmatrix}, \quad \boldsymbol{\mu}_2 = \begin{pmatrix} \mu_{c2}\mathbf{1}_{m_c} \\ \mu_{n2}\mathbf{1}_{m_n} \end{pmatrix}, \qquad \boldsymbol{\mu}_3 = \begin{pmatrix} \mu_{c3}\mathbf{1}_{m_c} \\ \mu_{n3}\mathbf{1}_{m_n} \end{pmatrix}. \tag{A2}$$

and covariance matrices

$$\begin{aligned} \boldsymbol{\Sigma}_1 &= \begin{pmatrix} \sigma_{c_1}^2 \boldsymbol{R}_{c_1} & \mathbf{0} \\ \mathbf{0} & \sigma_{n_1}^2 \boldsymbol{R}_{n_1} \end{pmatrix}, \quad \boldsymbol{\Sigma}_2 = \begin{pmatrix} \sigma_2^2 \boldsymbol{R}_{c_1} & \mathbf{0} \\ \mathbf{0} & \sigma_2^2 \boldsymbol{R}_{n_1} \end{pmatrix}, \\ \boldsymbol{\Sigma}_3 &= \begin{pmatrix} \sigma_{c_3}^2 \boldsymbol{R}_{c_3} & \mathbf{0} \\ \mathbf{0} & \sigma_{n_3}^2 \boldsymbol{R}_{n_3} \end{pmatrix}, \end{aligned} \tag{A3}$$

respectively, where correlation matrix

$$\boldsymbol{R}_t = (1 - \rho_t) \left[ \boldsymbol{I}_{n_t} + \frac{\rho_t}{(1 - \rho_t)} \mathbf{1}_{n_t} \mathbf{1}_{n_t}^T \right], \tag{A4}$$

$t = c_1$, $n_1$, 2, $c_3$, or $n_3$. $n_t = m_c$ if $t = c_1$ or $c_3$; $n_t = m$ if $t = 2$; $n_t = m_n$ if $t = n_1$, or $n_3$. Here we assume, without loss of generality, the first $m_c$ elements of the random vector $\mathbf{X}$ are for the abnormal tissue samples and the remaining $m_n$ elements are for the normal tissue samples. Let $\boldsymbol{\theta}_1 = (\mu_{c_1}, \sigma_{c_1}^2, \rho_{c_1}, \mu_{n_1}, \sigma_{n_1}^2, \rho_{n_1})^T$, $\boldsymbol{\theta}_2 = (\mu_{c2}, \mu_{n_2}, \sigma_2^2, \rho_{c_2}, \rho_{n_2})^T$, $\boldsymbol{\theta}_3 = (\mu_{c_3}, \sigma_{c_3}^2, \rho_{c_3}, \mu_{n_3}, \sigma_{n_3}^2, \rho_{n_3})^T$.

Note that $\sigma_{c_1}^2 > \sigma_{n_1}^2$ for component 1 in which genes are over-dispersed in abnormal tissue samples, and $\sigma_{c_3}^2 < \sigma_{n_3}^2$ for component 3 where genes are under-dispersed in abnormal samples. Our prior belief is that the majority of genes are usually non-differentially dispersed, so we assume $\pi_2 > \pi_1$ and $\pi_2 > \pi_3$.

We reparametrize variances as

$$s_k = \log(\sigma_k^2), \quad k = c_1, 2, c_3, n_1, n_3. \tag{A5}$$

We reparametrize variances again to make sure $\sigma_{c_1}^2 > \sigma_{n_1}^2$ and $\sigma_{c_3}^2 < \sigma_{n_3}^2$:

$$\begin{aligned} s_{n_1} =& s_{c_1} - \exp(\triangle_{n_1}) \\ s_{n_3} =& s_{c_3} + \exp(\triangle_{n_3}) \end{aligned} \tag{A6}$$

# B Re-parameterization of the correlation

To make sure the covariance matrix is positive definite, the correlation $\rho$ should satisfies the condition

$$-\frac{1}{n-1} < \rho < 1.$$

So we reparameterize the correlation parameter

$$\rho = \frac{\exp(r) - 1/(n-1)}{1+\exp(r)}$$

# C log density for genes in cluster 1

$$\begin{aligned}
\log\left[f_1(\boldsymbol{x})|\boldsymbol{\theta}_1\right] = &-\frac{n_c}{2}\log(2\pi) - \frac{n_c}{2}s_{c_1} - \frac{n_c}{2}\log(n_c) + \frac{n_c-1}{2}\log(n_c-1) + \frac{n_c}{2}\log\left[1+\exp(r_{c_1})\right] - \frac{r_{c_1}}{2} \\
&- \frac{\left[\boldsymbol{a}\left(\boldsymbol{x}_{c_1},\mu_{c_1}\right)\right]^T\left[\boldsymbol{a}\left(\boldsymbol{x}_{c_1},\mu_{c_1}\right)\right]}{2\exp(s_{c_1})}\frac{n_c-1}{n_c}\left[1+\exp(r_{c_1})\right] \\
&+ \frac{\left(\left[\boldsymbol{a}\left(\boldsymbol{x}_{c_1},\mu_{c_1}\right)\right]^T\mathbf{1}\right)^2\left[(n_c-2)+\exp(r_{c_1})(n_c-1)-\frac{1}{\exp(r_{c_1})}\right]}{2\exp(s_{c_1})n_c^2} \\
&- \frac{n_n}{2}\log(2\pi) - \frac{n_n}{2}\left[s_{c_1}-\exp(\triangle_{n_1})\right] - \frac{n_n}{2}\log(n_n) + \frac{n_n-1}{2}\log(n_n-1) + \frac{n_n}{2}\log\left[1+\exp(r_{n_1})\right] - \frac{r_{n_1}}{2} \\
&- \frac{\left[\boldsymbol{a}\left(\boldsymbol{x}_{n_1},\mu_{n_1}\right)\right]^T\left[\boldsymbol{a}\left(\boldsymbol{x}_{n_1},\mu_{n_1}\right)\right]}{2\exp(\left[s_{c_1}-\exp(\triangle_{n_1})\right])}\frac{n_n-1}{n_n}\left[1+\exp(r_{n_1})\right] \\
&+ \frac{\left(\left[\boldsymbol{a}\left(\boldsymbol{x}_{n_1},\mu_{n_1}\right)\right]^T\mathbf{1}\right)^2\left[(n_n-2)+\exp(r_{n_1})(n_n-1)-\frac{1}{\exp(r_{n_1})}\right]}{2\exp(\left[s_{c_1}-\exp(\triangle_{n_1})\right])n_n^2}
\end{aligned} \tag{A7}$$

where

$$\begin{aligned}
\boldsymbol{a}(\boldsymbol{x},\mu)^T\boldsymbol{a}(\boldsymbol{x},\mu) =& \boldsymbol{x}^T\boldsymbol{x} - 2\mu\mathbf{1}^T\boldsymbol{x} + n\mu^2 \\
\left(\boldsymbol{a}(\boldsymbol{x},\mu)^T\mathbf{1}\right)^2 =& \left(\mathbf{1}^T\boldsymbol{x}\right)^2 + n^2\mu^2 - 2n\mu\boldsymbol{x}^T\mathbf{1}.
\end{aligned}$$

# D log density for genes in cluster 2

$$
\begin{aligned}
\log\left[f_2(\boldsymbol{x})|\boldsymbol{\theta}_2\right] = &-\frac{n_c}{2}\log(2\pi) - \frac{n_c}{2}s_2 - \frac{n_c}{2}\log(n_c) + \frac{n_c-1}{2}\log(n_c-1) + \frac{n_c}{2}\log\left[1+\exp(r_{c_2})\right] - \frac{r_{c_2}}{2} \\
&- \frac{\left[\boldsymbol{a}\left(\boldsymbol{x}_{c_1},\mu_{c_2}\right)\right]^T\left[\boldsymbol{a}\left(\boldsymbol{x}_{c_1},\mu_{c_2}\right)\right]}{2\exp(s_2)}\frac{n_c-1}{n_c}\left[1+\exp(r_{c_2})\right] \\
&+ \frac{\left(\left[\boldsymbol{a}\left(\boldsymbol{x}_{c_1},\mu_{c_2}\right)\right]^T\mathbf{1}\right)^2\left[(n_c-2)+\exp(r_{c_2})(n_c-1)-\frac{1}{\exp(r_{c_2})}\right]}{2\exp(s_2)n_c^2} \\
&- \frac{n_n}{2}\log(2\pi) - \frac{n_n}{2}\left[s_2\right] - \frac{n_n}{2}\log(n_n) + \frac{n_n-1}{2}\log(n_n-1) + \frac{n_n}{2}\log\left[1+\exp(r_{n_2})\right] - \frac{r_{n_2}}{2} \\
&- \frac{\left[\boldsymbol{a}\left(\boldsymbol{x}_{n_1},\mu_{n_2}\right)\right]^T\left[\boldsymbol{a}\left(\boldsymbol{x}_{n_1},\mu_{n_2}\right)\right]}{2\exp([s_2])}\frac{n_n-1}{n_n}\left[1+\exp(r_{n_2})\right] \\
&+ \frac{\left(\left[\boldsymbol{a}\left(\boldsymbol{x}_{n_1},\mu_{n_2}\right)\right]^T\mathbf{1}\right)^2\left[(n_n-2)+\exp(r_{n_2})(n_n-1)-\frac{1}{\exp(r_{n_2})}\right]}{2\exp([s_2])n_n^2}
\end{aligned}
\tag{A8}
$$

# E log density for genes in cluster 3

$$
\begin{aligned}
\log\left[f_3(\boldsymbol{x})|\boldsymbol{\theta}_3\right] = &-\frac{n_c}{2}\log(2\pi) - \frac{n_c}{2}s_{c_3} - \frac{n_c}{2}\log(n_c) + \frac{n_c-1}{2}\log(n_c-1) + \frac{n_c}{2}\log\left[1+\exp(r_{c_3})\right] - \frac{r_{c_3}}{2} \\
&- \frac{\left[\boldsymbol{a}\left(\boldsymbol{x}_{c_3},\mu_{c_3}\right)\right]^T\left[\boldsymbol{a}\left(\boldsymbol{x}_{c_3},\mu_{c_3}\right)\right]}{2\exp(s_{c_3})}\frac{n_c-1}{n_c}\left[1+\exp(r_{c_3})\right] \\
&+ \frac{\left(\left[\boldsymbol{a}\left(\boldsymbol{x}_{c_3},\mu_{c_3}\right)\right]^T\mathbf{1}\right)^2\left[(n_c-2)+\exp(r_{c_3})(n_c-1)-\frac{1}{\exp(r_{c_3})}\right]}{2\exp(s_{c_3})n_c^2} \\
&- \frac{n_n}{2}\log(2\pi) - \frac{n_n}{2}\left[s_{c_3}+\exp(\triangle_{n_3})\right] - \frac{n_n}{2}\log(n_n) + \frac{n_n-1}{2}\log(n_n-1) + \frac{n_n}{2}\log\left[1+\exp(r_{n_3})\right] - \frac{r_{n_3}}{2} \\
&- \frac{\left[\boldsymbol{a}\left(\boldsymbol{x}_{n_3},\mu_{n_3}\right)\right]^T\left[\boldsymbol{a}\left(\boldsymbol{x}_{n_3},\mu_{n_3}\right)\right]}{2\exp([s_{c_3}+\exp(\triangle_{n_3})])}\frac{n_n-1}{n_n}\left[1+\exp(r_{n_3})\right] \\
&+ \frac{\left(\left[\boldsymbol{a}\left(\boldsymbol{x}_{n_3},\mu_{n_3}\right)\right]^T\mathbf{1}\right)^2\left[(n_n-2)+\exp(r_{n_3})(n_n-1)-\frac{1}{\exp(r_{n_3})}\right]}{2\exp([s_{c_3}+\exp(\triangle_{n_3})])n_n^2}
\end{aligned}
\tag{A9}
$$

# F Parameter Estimation by EM Algorithm

Based on Titterington et al. (1995, Section 4.3), the fully categorized data can be represented as

$$\{\boldsymbol{y}_i, i=1,\ldots,p\} = \{(\boldsymbol{x}_i^T, \boldsymbol{z}_i^T)^T : i=1,\ldots,p\},$$

where $p$ is the number of genes, $\boldsymbol{x}_i$ is a $n \times 1$ vector, $n = n_c + n_n$, $n_c$ is the number of cases, $n_n$ is the number of controls, $\boldsymbol{z}_i = (z_{i1}, z_{i2}, 1 - z_{i1} - z_{i2})$ and

$$z_{ij} = \begin{cases} 1 & \text{if } \boldsymbol{x}_i \text{ is in category j}, \\ 0 & \text{otherwise.} \end{cases}, \quad i = 1, \ldots, p, \quad j = 1, 2.$$

The likelihood corresponding to $(\boldsymbol{y}_1, \ldots, \boldsymbol{y}_p)$ can then be written in the form

$$\begin{aligned} g(\boldsymbol{y}_1, \ldots, \boldsymbol{y}_p | \boldsymbol{\Psi}) &= \prod_{i=1}^{p} f(\boldsymbol{x}_i, \boldsymbol{z}_i) \\ &= \prod_{i=1}^{p} f(\boldsymbol{x}_i | \boldsymbol{z}_i) f(\boldsymbol{z}_i) \\ &= \prod_{i=1}^{p} \left\{ \left[ f_1(\boldsymbol{x}_i)^{z_{i1}} f_2(\boldsymbol{x}_i)^{z_{i2}} f_3(\boldsymbol{x}_i)^{(1-z_{i1}-z_{i2})} \right] \left[ \pi_1^{z_{i1}} \pi_2^{z_{i2}} (1 - \pi_1 - \pi_2)^{(1-z_{i1}-z_{i2})} \right] \right\}, \end{aligned}$$

where

$$\boldsymbol{Z}_i \sim \text{Multinomial}\,(1, \pi_1, \pi_2, 1 - \pi_1 - \pi_2) \tag{A10}$$

and

$$\begin{aligned} f(\boldsymbol{z}_i) &= \begin{cases} \pi_1 & \text{if } z_{i1} = 1 \text{ and } z_{i2} = 0, \\ \pi_2 & \text{if } z_{i2} = 1 \text{ and } z_{i1} = 0, \\ 1 - \pi_1 - \pi_2 & \text{if } z_{i1} = z_{i2} = 0, \end{cases} \\ &= \pi_1^{z_{i1}} \pi_2^{z_{i2}} (1 - \pi_1 - \pi_2)^{(1-z_{i1}-z_{i2})} \end{aligned} \tag{A11}$$

and

$$\begin{aligned} f(\boldsymbol{x}_i | \boldsymbol{z}_i) &= \begin{cases} f_1(\boldsymbol{x}_i) & \text{if } z_{i1} = 1 \text{ and } z_{i2} 0, \\ f_2(\boldsymbol{x}_i) & \text{if } z_{i2} = 1 \text{ and } z_{i1} = 0, \\ f_3(\boldsymbol{x}_i) & \text{if } z_{i1} = z_{i2} = 0, \end{cases} \\ &= f_1(\boldsymbol{x}_i)^{z_{i1}} f_2(\boldsymbol{x}_i)^{z_{i2}} f_3(\boldsymbol{x}_i)^{(1-z_{i1}-z_{i2})} \end{aligned} \tag{A12}$$

The log complete likelihood function is

$$\ell_0(\boldsymbol{\Psi}) = \sum_{i=1}^{p} \boldsymbol{z}_i^T \boldsymbol{V}(\boldsymbol{\pi}) + \sum_{i=1}^{p} \boldsymbol{z}_i^T \boldsymbol{U}_i(\boldsymbol{\theta}),$$

where

$$\boldsymbol{z}_i = \begin{pmatrix} z_{i1} \\ z_{i2} \\ 1 - z_{i1} - z_{i2} \end{pmatrix}, \quad \boldsymbol{V}(\boldsymbol{\pi}) = \begin{pmatrix} \log(\pi_1) \\ \log(\pi_2) \\ \log(1 - \pi_1 - \pi_2) \end{pmatrix}, \quad \boldsymbol{U}_i(\boldsymbol{\theta}) = \begin{pmatrix} \log\left(f_1(\boldsymbol{x}_i | \boldsymbol{\theta}_1)\right) \\ \log\left(f_2(\boldsymbol{x}_i | \boldsymbol{\theta}_2)\right) \\ \log\left(f_3(\boldsymbol{x}_i | \boldsymbol{\theta}_3)\right) \end{pmatrix}$$

and

$$\boldsymbol{\theta}_1 = \begin{pmatrix} s_{c_1} \\ r_{c_1} \\ \mu_{c_1} \\ \triangle_{c_1} \\ r_{n_1} \\ \mu_{n_1} \end{pmatrix}, \quad \boldsymbol{\theta}_2 = \begin{pmatrix} s_2 \\ r_{c_2} \\ \mu_{c_2} \\ r_{n_2} \\ \mu_{n_2} \end{pmatrix}, \quad \boldsymbol{\theta}_3 = \begin{pmatrix} s_{c_3} \\ r_{c_3} \\ \mu_{c_3} \\ \triangle_{n_3} \\ r_{n_3} \\ \mu_{n_3} \end{pmatrix},$$

and

$$\boldsymbol{\Psi} = (\pi_1, \pi_2, s_{c_1}, r_{c_1}, \mu_{c_1}, \triangle_{c_1}, r_{n_1}, \mu_{n_1}, s_2, r_{c_2}, \mu_{c_2}, r_{n_2}, \mu_{n_2}, s_{c_3}, r_{c_3}, \mu_{c_3}, \triangle_{n_3}, r_{n_3}, \mu_{n_3})^T.$$

The EM algorithm generates, from some initial approximation, $\boldsymbol{\Psi}^{(0)}$, a sequence $\{\boldsymbol{\Psi}^{(m)}\}$ of estimates. Each iteration consists of the following double step:

**E step:** Evaluate $\mathrm{E}\left[\log(g(\boldsymbol{y}|\boldsymbol{\Psi})|\boldsymbol{x}, \boldsymbol{\Psi}^{(m)}\right] = Q\left(\boldsymbol{\Psi}, \boldsymbol{\Psi}^{(m)}\right)$, say.

**M step:** Find $\boldsymbol{\Psi} = \boldsymbol{\Psi}^{(m+1)}$ to maximize $Q\left(\boldsymbol{\Psi}, \boldsymbol{\Psi}^{(m)}\right)$.

To stablize the estimates of $\pi_\ell$, $\ell = 1, 2, 3$, we assume that the vector of mixing proportions $(\pi_1, \pi_2, \pi_3)^T$ are Dirichlet distributed with parameters $b_1 = b_2 = b_3 = 3$.

# G E-step of the EM algorithm

We can obtain

$$Q\left(\boldsymbol{\Psi}, \boldsymbol{\Psi}^{(m)}\right) = \sum_{i=1}^{p}\left[\mathrm{E}\left(\boldsymbol{z}_i|\boldsymbol{x}, \boldsymbol{\Psi}^{(m)}\right)\right]^T \boldsymbol{V}(\boldsymbol{\pi}) + \sum_{i=1}^{p}\left[\mathrm{E}\left(\boldsymbol{z}_i|\boldsymbol{x}, \boldsymbol{\Psi}^{(m)}\right)\right]^T \boldsymbol{U}(\boldsymbol{\theta}).$$

Denote

$$\boldsymbol{w}_i\left(\boldsymbol{\Psi}^{(m)}\right) = \mathrm{E}\left(\boldsymbol{z}_i|\boldsymbol{x}, \boldsymbol{\Psi}^{(m)}\right) = \mathrm{E}\left(\boldsymbol{z}_i|\boldsymbol{x}_i, \boldsymbol{\Psi}^{(m)}\right).$$

The last equality is because of the independence of data points.

The $j$-th element of $\boldsymbol{w}_i\left(\boldsymbol{\Psi}^{(m)}\right)$ is

$$\begin{aligned}
w_{ij}\left(\boldsymbol{\Psi}^{(m)}\right) =& \mathrm{E}\left(z_{ij}|\boldsymbol{x}_i, \boldsymbol{\Psi}^{(m)}\right) \\
=& Pr\left(z_{ij}=1|\boldsymbol{x}_i, \boldsymbol{\Psi}^{(m)}\right) \\
=& \frac{Pr\left(\boldsymbol{x}_i|z_{ij}=1, \boldsymbol{\Psi}^{(m)}\right) Pr\left(z_{ij}=1|\boldsymbol{\Psi}^{(m)}\right)}{f\left(\boldsymbol{x}_i, \boldsymbol{\Psi}^{(m)}\right)} \\
=& \frac{f_j\left(\boldsymbol{x}_i|\boldsymbol{\theta}_j^{(m)}\right)\pi_j^{(m)}}{f\left(\boldsymbol{x}_i|\boldsymbol{\Psi}^{(m)}\right)},
\end{aligned}$$

where

$$f\left(\boldsymbol{x}_i|\boldsymbol{\Psi}^{(m)}\right) = f_1\left(\boldsymbol{x}_i|\boldsymbol{\theta}_1^{(m)}\right)\pi_1^{(m)} + f_2\left(\boldsymbol{x}_i|\boldsymbol{\theta}_2^{(m)}\right)\pi_2^{(m)} + f_3\left(\boldsymbol{x}_i|\boldsymbol{\theta}_3^{(m)}\right)\left[1-\pi_1^{(m)}-\pi_2^{(m)}\right].$$

These "weights" ($w_{ij}\left(\boldsymbol{\Psi}^{(m)}\right)$, $i=1,\ldots,p$, $j=1,2$) are therefore the probabilities of category membership for the $i$-th observation, conditional on $x_i$ and given that the parameter is $\boldsymbol{\Psi}^{(m)}$.

$$\begin{aligned}
Q(\boldsymbol{\Psi}, \boldsymbol{\Psi}^{(m)}) =& \sum_{i=1}^{p}\Big\{\mathrm{E}\left(z_{i1}|\boldsymbol{x}_i, \boldsymbol{\Psi}^{(m)}\right)\log(\pi_1) + \mathrm{E}\left(z_{i2}|\boldsymbol{x}_i, \boldsymbol{\Psi}^{(m)}\right)\log(\pi_2) \\
&+\mathrm{E}\left(1-z_{i1}-z_{i2}|\boldsymbol{x}_i, \boldsymbol{\Psi}^{(m)}\right)\log(\pi_3) \\
&+\mathrm{E}\left(z_{i1}|\boldsymbol{x}_i, \boldsymbol{\Psi}^{(m)}\right)\log(f_1(\boldsymbol{x}_i)) + \mathrm{E}\left(z_{i2}|\boldsymbol{x}_i, \boldsymbol{\Psi}^{(m)}\right)\log(f_2(\boldsymbol{x}_i)) \\
&+\mathrm{E}\left(1-z_{i1}-z_{i2}|\boldsymbol{x}_i, \boldsymbol{\Psi}^{(m)}\right)\log(f_3(\boldsymbol{x}_i))\Big\} \\
=& \sum_{i=1}^{p}\Big\{w_{i1}\left(\boldsymbol{\Psi}^{(m)}\right)\log(\pi_1) + w_{i2}\left(\boldsymbol{\Psi}^{(m)}\right)\log(\pi_2) \\
&+\left[1-w_{i1}\left(\boldsymbol{\Psi}^{(m)}\right)-w_{i2}\left(\boldsymbol{\Psi}^{(m)}\right)\right]\log(1-\pi_1-\pi_2) \\
&+w_{i1}\left(\boldsymbol{\Psi}^{(m)}\right)\log(f_1(\boldsymbol{x}_i)) + w_{i2}\left(\boldsymbol{\Psi}^{(m)}\right)\log(f_2(\boldsymbol{x}_i)) \\
&+\left[1-w_{i1}\left(\boldsymbol{\Psi}^{(m)}\right)-w_{i2}\left(\boldsymbol{\Psi}^{(m)}\right)\right]\log(f_3(\boldsymbol{x}_i))\Big\}.
\end{aligned} \tag{A13}$$

# H The M-step of the E-M algorithm for 3-mixture distribution

In the M step, we need to maximize the following function

$$\begin{aligned} Q\left(\boldsymbol{\Psi}|\boldsymbol{\Psi}^{(m)}\right) =& \log\left[\frac{\Gamma\left(b_1+b_2+b_3\right)}{\Gamma(b_1)\Gamma(b_2)\Gamma(b_3)}\right] + \left[w_1^{(m)} + (b_1-1)\right]\log(\pi_1) \\ &+ \left[w_2^{(m)} + (b_2-1)\right]\log(\pi_2) + \left[w_3^{(m)} + (b_3-1)\right]\log\left(1-\pi_1-\pi_2\right) \\ &+ \sum_{\ell=1}^{3}\sum_{i=1}^{p} w_{i\ell}^{(m)}\log\left[f_\ell\left(\boldsymbol{x}_i|\boldsymbol{\theta}_\ell\right)\right], \end{aligned}$$

where

$$\begin{aligned} w_\ell^{(m)} =& \sum_{i=1}^{p} w_{i\ell}^{(m)}, \\ w_{i\ell}^{(m)} =& \frac{\pi_\ell f_\ell\left(\boldsymbol{x}_i|\boldsymbol{\theta}_\ell^{(m)}\right)}{\sum_{t=1}^{3}\pi_t f_t\left(\boldsymbol{x}_i|\boldsymbol{\theta}_t^{(m)}\right)} \end{aligned}$$

and the superscript $(m)$ indicates the number of iterations.

By letting the first derivative to zero, we can get

$$\pi_\ell^{(m+1)} = \frac{\left[w_\ell^{(m)} + (b_\ell - 1)\right]}{(p+b_1+b_2+b_3-3)}. \tag{A14}$$

Denote

$$Q_\ell\left(\boldsymbol{\Psi}|\boldsymbol{\Psi}^{(m)}\right) = \sum_{i=1}^{p} w_{i\ell}^{(m)}\log\left[f_\ell\left(\boldsymbol{x}_i|\boldsymbol{\theta}_\ell\right)\right]$$

The first derivatives are:

$$\frac{\partial Q_\ell}{\partial \boldsymbol{\theta}_\ell} = \sum_{i=1}^{p} w_{i\ell}^{(m)}\frac{\partial\log\left[f_\ell\left(\boldsymbol{x}_i|\boldsymbol{\theta}_\ell\right)\right]}{\partial\boldsymbol{\theta}_\ell}$$

## H.1 The first derivative of $\partial \log \left[ f_1 \left( \boldsymbol{x}_i | \boldsymbol{\theta}_1 \right) \right] / \partial s_{c_1}$

$$
\begin{aligned}
\frac{\partial \log \left[ f_1 \left( \boldsymbol{x}_i | \boldsymbol{\theta}_1 \right) \right]}{\partial s_{c_1}} =& - \frac{n_c}{2} - \frac{(n_c - 1)}{2 n_c} \left[ 1 + \exp(r_{c_1}) \right] \left[ a \left( \boldsymbol{x}_{ci}, \mu_{c_1} \right) \right]^T \left[ a \left( \boldsymbol{x}_{ci}, \mu_{c_1} \right) \right] (-1) \left[ \exp(s_{c_1}) \right]^{-2} \exp(s_{c_1}) \\
&+ \frac{\left[ a \left( \boldsymbol{x}_{ci}, \mu_{c_1} \right)^T \mathbf{1} \right]^2 \left[ (n_c - 2) + \exp(r_{c_1})(n_c - 1) - \frac{1}{\exp(r_{c_1})} \right]}{2 n_c^2} (-1) \left[ \exp(s_{c_1}) \right]^2 \exp\left( s_{c_1} \right) \\
&- \frac{n_n}{2} - \frac{(n_n - 1)}{2 n_n} \left[ 1 + \exp(r_{n_1}) \right] \left[ a \left( \boldsymbol{x}_{ni}, \mu_{n_1} \right) \right]^T \left[ a \left( \boldsymbol{x}_{ni}, \mu_{n_1} \right) \right] (-1) \left[ \exp\left( s_{c_1} - \exp(\triangle_{n_1}) \right) \right]^{-2} \\
&\cdot \exp\left( s_{c_1} - \exp(\triangle_{n_1}) \right) \\
&+ \frac{\left[ a \left( \boldsymbol{x}_{ni}, \mu_{n_1} \right)^T \mathbf{1} \right]^2 \left[ (n_n - 2) + \exp(r_{n_1})(n_n - 1) - \frac{1}{\exp(r_{n_1})} \right]}{2 n_n^2} (-1) \left[ \exp\left( s_{c_1} - \exp(\triangle_{n_1}) \right] ) \right]^{-2} \\
&\cdot \exp\left( s_{c_1} - \exp(\triangle_{n_1}) \right) \\
=& - \frac{n}{2} + \frac{(n_c - 1)}{2 n_c \exp\left( s_{c_1} \right)} \left[ 1 + \exp(r_{c_1}) \right] \left[ a \left( \boldsymbol{x}_{ci}, \mu_{c_1} \right) \right]^T \left[ a \left( \boldsymbol{x}_{ci}, \mu_{c_1} \right) \right] \\
&- \frac{\left[ a \left( \boldsymbol{x}_{ci}, \mu_{c_1} \right)^T \mathbf{1} \right]^2 \left[ (n_c - 2) + \exp(r_{c_1})(n_c - 1) - \frac{1}{\exp(r_{c_1})} \right]}{2 n_c^2 \exp\left( s_{c_1} \right)} \\
&+ \frac{(n_n - 1)}{2 n_n \exp\left[ s_{c_1} - \exp(\triangle_{n_1}) \right]} \left[ 1 + \exp(r_{n_1}) \right] \left[ a \left( \boldsymbol{x}_{ni}, \mu_{n_1} \right) \right]^T \left[ a \left( \boldsymbol{x}_{ni}, \mu_{n_1} \right) \right] \\
&- \frac{\left[ a \left( \boldsymbol{x}_{ni}, \mu_{n_1} \right)^T \mathbf{1} \right]^2 \left[ (n_n - 2) + \exp(r_{n_1})(n_n - 1) - \frac{1}{\exp(r_{n_1})} \right]}{2 n_n^2 \exp\left[ s_{c_1} - \exp(\triangle_{n_1}) \right]}
\end{aligned}
$$

## H.2 The first derivative of $\partial \log \left[ f_1 \left( \boldsymbol{x}_i | \boldsymbol{\theta}_1 \right) \right] / \partial \triangle_{n_1}$

$$
\begin{aligned}
\frac{\partial \log \left[ f_1 \left( \boldsymbol{x}_i | \boldsymbol{\theta}_1 \right) \right]}{\partial \triangle_{n_1}} =& - \frac{n_n}{2} (-1) \exp\left( \triangle_{n_1} \right) - \frac{\left[ a \left( \boldsymbol{x}_{ni}, \mu_{n_1} \right) \right]^T \left[ a \left( \boldsymbol{x}_{ni}, \mu_{n_1} \right) \right]}{2} \frac{n_n - 1}{n_n} \left[ 1 + \exp\left( r_{n_1} \right) \right] \frac{\exp\left( \triangle_{n_1} \right)}{\exp\left[ s_{c_1} - \exp\left( \triangle_{n_1} \right) \right]} \\
&+ \left[ a \left( \boldsymbol{x}_{ni}, \mu_{n_1} \right)^T \mathbf{1} \right]^2 \frac{\left[ (n_n - 2) + \exp\left( r_{n_1} \right) (n_n - 1) - \frac{1}{\exp(r_{n_1})} \right]}{2 n_n^2} (-1) \frac{(-1) \exp\left( \triangle_{n_1} \right)}{\exp\left[ s_{c_1} - \exp\left( \triangle_{n_1} \right) \right]} \\
=& \frac{n_n}{2} \exp\left( \triangle_{n_1} \right) - \frac{\left[ a \left( \boldsymbol{x}_{ni}, \mu_{n_1} \right) \right]^T \left[ a \left( \boldsymbol{x}_{ni}, \mu_{n_1} \right) \right]}{2} \frac{n_n - 1}{n_n} \left[ 1 + \exp\left( r_{n_1} \right) \right] \frac{\exp\left( \triangle_{n_1} \right)}{\exp\left[ s_{c_1} - \exp\left( \triangle_{n_1} \right) \right]} \\
&+ \left[ a \left( \boldsymbol{x}_{ni}, \mu_{n_1} \right)^T \mathbf{1} \right]^2 \frac{\left[ (n_n - 2) + \exp\left( r_{n_1} \right) (n_n - 1) - \frac{1}{\exp(r_{n_1})} \right]}{2 n_n^2} \frac{\exp\left( \triangle_{n_1} \right)}{\exp\left[ s_{c_1} - \exp\left( \triangle_{n_1} \right) \right]}
\end{aligned}
$$

## H.3 The first derivative of $\partial \log\left[f_1\left(\boldsymbol{x}_i|\boldsymbol{\theta}_1\right)\right]/\partial r_{c_1}$

$$
\begin{aligned}
\frac{\partial \log\left[f_1(\boldsymbol{x}_i|\boldsymbol{\theta}_1)\right]}{\partial r_{c_1}} =& \frac{n_c}{2}\frac{\exp\left(r_{c_1}\right)}{1+\exp\left(r_{c_1}\right)} - \frac{1}{2} \\
&- \frac{\left[a\left(\boldsymbol{x}_{ci},\mu_{c_1}\right)\right]^T\left[a\left(\boldsymbol{x}_{ci},\mu_{c_1}\right)\right]}{2\exp\left(s_{c_1}\right)}\frac{n_c-1}{n_c}\exp(r_{c_1}) \\
&+ \frac{\left[a\left(\boldsymbol{x}_{ci},\mu_{c_1}\right)^T\mathbf{1}\right]^2\left[\exp\left(r_{c_1}\right)\left(n_c-1\right)-(-1)\left[\exp\left(r_{c_1}\right)\right]^2\exp\left(r_{c_1}\right)\right]}{2\exp\left(s_{c_1}\right)n_c^2} \\
=& \frac{n_c}{2}\frac{\exp\left(r_{c_1}\right)}{1+\exp\left(r_{c_1}\right)} - \frac{1}{2} \\
&- \frac{\left[a\left(\boldsymbol{x}_{ci},\mu_{c_1}\right)\right]^T\left[a\left(\boldsymbol{x}_{ci},\mu_{c_1}\right)\right]}{2\exp\left(s_{c_1}\right)}\frac{n_c-1}{n_c}\exp(r_{c_1}) \\
&+ \frac{\left[a\left(\boldsymbol{x}_{ci},\mu_{c_1}\right)^T\mathbf{1}\right]^2\left[\exp\left(r_{c_1}\right)\left(n_c-1\right)+\frac{1}{\exp\left(r_{c_1}\right)}\right]}{2\exp\left(s_{c_1}\right)n_c^2}
\end{aligned}
$$

## H.4 The first derivative of $\partial \log\left[f_1\left(\boldsymbol{x}_i|\boldsymbol{\theta}_1\right)\right]/\partial r_{n_1}$

$$
\begin{aligned}
\frac{\partial \log\left[f_1(\boldsymbol{x}_i|\boldsymbol{\theta}_1)\right]}{\partial r_{n_1}} =& \frac{n_n}{2}\frac{\exp\left(r_{n_1}\right)}{1+\exp\left(r_{n_1}\right)} - \frac{1}{2} \\
&- \frac{\left[a\left(\boldsymbol{x}_{ni},\mu_{n_1}\right)\right]^T\left[a\left(\boldsymbol{x}_{ni},\mu_{n_1}\right)\right]}{2\exp\left[s_{n_1}-\exp\left(\triangle_{n_1}\right)\right]}\frac{n_n-1}{n_n}\exp(r_{n_1}) \\
&+ \frac{\left[a\left(\boldsymbol{x}_{ni},\mu_{n_1}\right)^T\mathbf{1}\right]^2\left[\exp\left(r_{n_1}\right)\left(n_n-1\right)-(-1)\left[\exp\left(r_{n_1}\right)\right]^2\exp\left(r_{n_1}\right)\right]}{2\exp\left[s_{n_1}-\exp\left(\triangle_{n_1}\right)\right]n_n^2} \\
=& \frac{n_n}{2}\frac{\exp\left(r_{n_1}\right)}{1+\exp\left(r_{n_1}\right)} - \frac{1}{2} \\
&- \frac{\left[a\left(\boldsymbol{x}_{ni},\mu_{n_1}\right)\right]^T\left[a\left(\boldsymbol{x}_{ni},\mu_{n_1}\right)\right]}{2\exp\left[s_{n_1}-\exp\left(\triangle_{n_1}\right)\right]}\frac{n_n-1}{n_n}\exp(r_{n_1}) \\
&+ \frac{\left[a\left(\boldsymbol{x}_{ni},\mu_{n_1}\right)^T\mathbf{1}\right]^2\left[\exp\left(r_{n_1}\right)\left(n_n-1\right)+\frac{1}{\exp\left(r_{n_1}\right)}\right]}{2\exp\left[s_{n_1}-\exp\left(\triangle_{n_1}\right)\right]n_n^2}
\end{aligned}
$$

## H.5 The first derivative of $\partial \log\left[f_1\left(\boldsymbol{x}_i|\boldsymbol{\theta}_1\right)\right]/\partial \mu_{c_1}$

$$
\begin{aligned}
\frac{\partial \log\left[f_1\left(\boldsymbol{x}_i|\boldsymbol{\theta}_1\right)\right]}{\partial \mu_{c_1}} =& -\frac{1}{2\exp\left(s_{c_1}\right)}\frac{n_c-1}{n_c}\left[1+\exp\left(r_{c_1}\right)\right]2\left(n_c\mu_{c_1}-\mathbf{1}^T\boldsymbol{x}_c\right) \\
&+\frac{\left[(n_c-2)+\exp(r_{c_1})(n_c-1)-\frac{1}{\exp(r_{c_1})}\right]}{2\exp\left(s_{c_1}\right)n_c^2}2n_c\left(n_c\mu_{c_1}-\mathbf{1}^T\boldsymbol{x}_c\right) \\
=& -\frac{\left(n_c\mu_{c_1}-\mathbf{1}^T\boldsymbol{x}_c\right)}{\exp\left(s_{c_1}\right)}\frac{n_c-1}{n_c}\left[1+\exp\left(r_{c_1}\right)\right] \\
&+\frac{\left(n_c\mu_{c_1}-\mathbf{1}^T\boldsymbol{x}_c\right)\left[(n_c-2)+\exp(r_{c_1})(n_c-1)-\frac{1}{\exp(r_{c_1})}\right]}{\exp\left(s_{c_1}\right)n_c}
\end{aligned}
$$

## H.6 The first derivative of $\partial \log\left[f_1\left(\boldsymbol{x}_i|\boldsymbol{\theta}_1\right)\right]/\partial \mu_{n_1}$

$$
\begin{aligned}
\frac{\partial \log\left[f_1\left(\boldsymbol{x}_i|\boldsymbol{\theta}_1\right)\right]}{\partial \mu_{n_1}} =& -\frac{\left(n_n\mu_{n_1}-\mathbf{1}^T\boldsymbol{x}_n\right)}{\exp\left[s_{c_1}-\exp\left(\triangle_{n_1}\right)\right]}\frac{n_n-1}{n_n}\left[1+\exp\left(r_{n_1}\right)\right] \\
&+\frac{\left(n_n\mu_{n_1}-\mathbf{1}^T\boldsymbol{x}_n\right)\left[(n_n-2)+\exp(r_{n_1})(n_n-1)-\frac{1}{\exp(r_{n_1})}\right]}{\exp\left[s_{c_1}-\exp\left(\triangle_{n_1}\right)\right]n_n}
\end{aligned}
$$

## H.7 The first derivative of $\partial \log\left[f_2\left(\boldsymbol{x}_i|\boldsymbol{\theta}_2\right)\right]/\partial s_2$

$$
\begin{aligned}
\frac{\partial \log\left[f_2\left(\boldsymbol{x}_i|\boldsymbol{\theta}_2\right)\right]}{\partial s_2} =& -\frac{n}{2}+\frac{\left[a\left(\boldsymbol{x}_{ci},\mu_{c_2}\right)\right]^T\left[a\left(\boldsymbol{x}_{ci},\mu_{c_2}\right)\right]}{2\exp(s_2)}\frac{n_c-1}{n_c}\left[1+\exp\left(r_{c_2}\right)\right] \\
&-\frac{\left[a\left(\boldsymbol{x}_{ci},\mu_{c_2}\right)^T\mathbf{1}\right]^2\left[(n_c-2)+\exp(r_{c_2})(n_c-1)-\frac{1}{\exp(r_{c_2})}\right]}{2\exp\left(s_2\right)n_c^2} \\
&+\frac{\left[a\left(\boldsymbol{x}_{ni},\mu_{n_2}\right)\right]^T\left[a\left(\boldsymbol{x}_{ni},\mu_{n_2}\right)\right]}{2\exp(s_2)}\frac{n_n-1}{n_n}\left[1+\exp\left(r_{n_2}\right)\right] \\
&-\frac{\left[a\left(\boldsymbol{x}_{ni},\mu_{n_2}\right)^T\mathbf{1}\right]^2\left[(n_n-2)+\exp(r_{n_2})(n_n-1)-\frac{1}{\exp(r_{n_2})}\right]}{2\exp\left(s_2\right)n_n^2}
\end{aligned}
$$

## H.8 The first derivative of $\partial \log\left[f_2\left(\boldsymbol{x}_i|\boldsymbol{\theta}_2\right)\right]/\partial r_{c_2}$

$$
\begin{aligned}
\frac{\partial \log\left[f_2(\boldsymbol{x}_i|\boldsymbol{\theta}_2)\right]}{\partial r_{c_2}} =& \frac{n_c}{2}\frac{\exp\left(r_{c_2}\right)}{1+\exp\left(r_{c_2}\right)}-\frac{1}{2} \\
&-\frac{\left[a\left(\boldsymbol{x}_{ci},\mu_{c_2}\right)\right]^T\left[a\left(\boldsymbol{x}_{ci},\mu_{c_2}\right)\right]}{2\exp\left(s_2\right)}\frac{n_c-1}{n_c}\exp(r_{c_2}) \\
&+\frac{\left[a\left(\boldsymbol{x}_{ci},\mu_{c_2}\right)^T\mathbf{1}\right]^2\left[\exp\left(r_{c_2}\right)\left(n_c-1\right)+\frac{1}{\exp(r_{c_2})}\right]}{2\exp\left(s_2\right)n_c^2}
\end{aligned}
$$

## H.9 The first derivative of $\partial \log \left[ f_2 \left( \boldsymbol{x}_i | \boldsymbol{\theta}_2 \right) \right] / \partial r_{n_2}$

$$\begin{aligned}\frac{\partial \log \left[f_2(\boldsymbol{x}_i|\boldsymbol{\theta}_2)\right]}{\partial r_{n_2}} =& \frac{n_n}{2} \frac{\exp\left(r_{n_2}\right)}{1+\exp\left(r_{n_2}\right)} - \frac{1}{2} \\ &- \frac{\left[a\left(\boldsymbol{x}_{ci}, \mu_{n_2}\right)\right]^T \left[a\left(\boldsymbol{x}_{ni}, \mu_{n_2}\right)\right]}{2\exp\left(s_2\right)} \frac{n_n - 1}{n_n} \exp(r_{n_2}) \\ &+ \frac{\left[a\left(\boldsymbol{x}_{ni}, \mu_{n_2}\right)^T \mathbf{1}\right]^2 \left[\exp\left(r_{n_2}\right)\left(n_n - 1\right) + \frac{1}{\exp\left(r_{n_2}\right)}\right]}{2\exp\left(s_2\right) n_n^2}\end{aligned}$$

## H.10 The first derivative of $\partial \log \left[ f_2 \left( \boldsymbol{x}_i | \boldsymbol{\theta}_2 \right) \right] / \partial \mu_{c_2}$

$$\begin{aligned}\frac{\partial \log \left[f_2\left(\boldsymbol{x}_i|\boldsymbol{\theta}_2\right)\right]}{\partial \mu_{c_2}} =& - \frac{1}{2\exp\left(s_2\right)} \frac{n_c - 1}{n_c} \left[1+\exp\left(r_{c_2}\right)\right] 2 \left(n_c \mu_{c_2} - \mathbf{1}^T \boldsymbol{x}_c\right) \\ &+ \frac{\left[(n_c - 2) + \exp(r_{c_2})(n_c - 1) - \frac{1}{\exp\left(r_{c_2}\right)}\right]}{2\exp\left(s_2\right) n_c^2} 2n_c \left(n_c \mu_{c_2} - \mathbf{1}^T \boldsymbol{x}_c\right) \\ =& - \frac{\left(n_c \mu_{c_2} - \mathbf{1}^T \boldsymbol{x}_c\right)}{\exp\left(s_2\right)} \frac{n_c - 1}{n_c} \left[1+\exp\left(r_{c_2}\right)\right] \\ &+ \frac{\left(n_c \mu_{c_2} - \mathbf{1}^T \boldsymbol{x}_c\right) \left[(n_c - 2) + \exp(r_{c_2})(n_c - 1) - \frac{1}{\exp\left(r_{c_2}\right)}\right]}{\exp\left(s_2\right) n_c}\end{aligned}$$

## H.11 The first derivative of $\partial \log \left[ f_2 \left( \boldsymbol{x}_i | \boldsymbol{\theta}_2 \right) \right] / \partial \mu_{n_2}$

$$\begin{aligned}\frac{\partial \log \left[f_2\left(\boldsymbol{x}_i|\boldsymbol{\theta}_2\right)\right]}{\partial \mu_{n_2}} =& - \frac{1}{2\exp\left(s_2\right)} \frac{n_n - 1}{n_n} \left[1+\exp\left(r_{n_2}\right)\right] 2 \left(n_n \mu_{n_2} - \mathbf{1}^T \boldsymbol{x}_n\right) \\ &+ \frac{\left[(n_n - 2) + \exp(r_{n_2})(n_n - 1) - \frac{1}{\exp\left(r_{n_2}\right)}\right]}{2\exp\left(s_2\right) n_n^2} 2n_n \left(n_n \mu_{n_2} - \mathbf{1}^T \boldsymbol{x}_n\right) \\ =& - \frac{\left(n_n \mu_{n_2} - \mathbf{1}^T \boldsymbol{x}_n\right)}{\exp\left(s_2\right)} \frac{n_n - 1}{n_n} \left[1+\exp\left(r_{n_2}\right)\right] \\ &+ \frac{\left(n_n \mu_{n_2} - \mathbf{1}^T \boldsymbol{x}_n\right) \left[(n_n - 2) + \exp(r_{n_2})(n_n - 1) - \frac{1}{\exp\left(r_{n_2}\right)}\right]}{\exp\left(s_2\right) n_n}\end{aligned}$$

## H.12 The first derivative of $\partial \log\left[f_3\left(\boldsymbol{x}_i|\boldsymbol{\theta}_3\right)\right]/\partial s_{c_3}$

$$\begin{aligned}
\frac{\partial \log\left[f_3\left(\boldsymbol{x}_i|\boldsymbol{\theta}_3\right)\right]}{\partial s_{c_3}} =& -\frac{n_c}{2} - \frac{(n_c-1)}{2n_c}\left[1+\exp(r_{c_3})\right]\left[a\left(\boldsymbol{x}_{ci},\mu_{c_3}\right)\right]^T\left[a\left(\boldsymbol{x}_{ci},\mu_{c_3}\right)\right](-1)\left[\exp(s_{c_3})\right]^{-2}\exp(s_{c_3}) \\
&+ \frac{\left[a\left(\boldsymbol{x}_{ci},\mu_{c_3}\right)^T\mathbf{1}\right]^2\left[(n_c-2)+\exp(r_{c_3})(n_c-1)-\frac{1}{\exp(r_{c_3})}\right]}{2n_c^2}(-1)\left[\exp(s_{c_3})\right]^2\exp\left(s_{c_3}\right) \\
&- \frac{n_n}{2} - \frac{(n_n-1)}{2n_n}\left[1+\exp(r_{n_3})\right]\left[a\left(\boldsymbol{x}_{ni},\mu_{n_3}\right)\right]^T\left[a\left(\boldsymbol{x}_{ni},\mu_{n_3}\right)\right](-1)\left[\exp\left(s_{c_3}+\exp(\triangle_{n_3})\right)\right]^{-2} \\
&\cdot\exp\left(s_{c_3}+\exp(\triangle_{n_3})\right) \\
&+ \frac{\left[a\left(\boldsymbol{x}_{ni},\mu_{n_3}\right)^T\mathbf{1}\right]^2\left[(n_n-2)+\exp(r_{n_3})(n_n-1)-\frac{1}{\exp(r_{n_3})}\right]}{2n_n^2}(-1)\left[\exp\left(s_{c_3}+\exp(\triangle_{n_3})\right]\right)^{-2} \\
&\cdot\exp\left(s_{c_3}+\exp(\triangle_{n_3})\right) \\
=& -\frac{n}{2} + \frac{(n_c-1)}{2n_c\exp\left(s_{c_3}\right)}\left[1+\exp(r_{c_3})\right]\left[a\left(\boldsymbol{x}_{ci},\mu_{c_3}\right)\right]^T\left[a\left(\boldsymbol{x}_{ci},\mu_{c_3}\right)\right] \\
&- \frac{\left[a\left(\boldsymbol{x}_{ci},\mu_{c_3}\right)^T\mathbf{1}\right]^2\left[(n_c-2)+\exp(r_{c_3})(n_c-1)-\frac{1}{\exp(r_{c_3})}\right]}{2n_c^2\exp\left(s_{c_3}\right)} \\
&+ \frac{(n_n-1)}{2n_n\exp\left[s_{c_3}+\exp(\triangle_{n_3})\right]}\left[1+\exp(r_{n_3})\right]\left[a\left(\boldsymbol{x}_{ni},\mu_{n_3}\right)\right]^T\left[a\left(\boldsymbol{x}_{ni},\mu_{n_3}\right)\right] \\
&- \frac{\left[a\left(\boldsymbol{x}_{ni},\mu_{n_3}\right)^T\mathbf{1}\right]^2\left[(n_n-2)+\exp(r_{n_3})(n_n-1)-\frac{1}{\exp(r_{n_3})}\right]}{2n_n^2\exp\left[s_{c_3}+\exp(\triangle_{n_3})\right]}
\end{aligned}$$

## H.13 The first derivative of $\partial \log\left[f_3\left(\boldsymbol{x}_i|\boldsymbol{\theta}_3\right)\right]/\partial \triangle_{n_3}$

$$\begin{aligned}
\frac{\partial \log\left[f_3\left(\boldsymbol{x}_i|\boldsymbol{\theta}_3\right)\right]}{\partial \triangle_{n_3}} =& -\frac{n_n}{2}\exp\left(\triangle_{n_3}\right) - \frac{\left[a\left(\boldsymbol{x}_{ni},\mu_{n_3}\right)\right]^T\left[a\left(\boldsymbol{x}_{ni},\mu_{n_3}\right)\right]}{2}\frac{n_n-1}{n_n}\left[1+\exp\left(r_{n_3}\right)\right]\frac{(-1)\exp\left(\triangle_{n_3}\right)}{\exp\left[s_{c_3}+\exp\left(\triangle_{n_3}\right)\right]} \\
&+ \left[a\left(\boldsymbol{x}_{ni},\mu_{n_3}\right)^T\mathbf{1}\right]^2\frac{\left[(n_n-2)+\exp\left(r_{n_3}\right)(n_n-1)-\frac{1}{\exp(r_{n_3})}\right]}{2n_n^2}(-1)\frac{\exp\left(\triangle_{n_3}\right)}{\exp\left[s_{c_3}+\exp\left(\triangle_{n_3}\right)\right]} \\
=& -\frac{n_n}{2}\exp\left(\triangle_{n_3}\right) - \frac{\left[a\left(\boldsymbol{x}_{ni},\mu_{n_3}\right)\right]^T\left[a\left(\boldsymbol{x}_{ni},\mu_{n_3}\right)\right]}{2}\frac{n_n-1}{n_n}\left[1+\exp\left(r_{n_3}\right)\right]\frac{\exp\left(\triangle_{n_3}\right)}{\exp\left[s_{c_3}+\exp\left(\triangle_{n_3}\right)\right]} \\
&- \left[a\left(\boldsymbol{x}_{ni},\mu_{n_3}\right)^T\mathbf{1}\right]^2\frac{\left[(n_n-2)+\exp\left(r_{n_3}\right)(n_n-1)-\frac{1}{\exp(r_{n_3})}\right]}{2n_n^2}\frac{\exp\left(\triangle_{n_3}\right)}{\exp\left[s_{c_3}+\exp\left(\triangle_{n_3}\right)\right]}
\end{aligned}$$

## H.14 The first derivative of $\partial \log\left[f_3\left(\boldsymbol{x}_i|\boldsymbol{\theta}_3\right)\right]/\partial r_{c_3}$

$$\begin{aligned}\frac{\partial \log\left[f_3(\boldsymbol{x}_i|\boldsymbol{\theta}_3)\right]}{\partial r_{c_3}} =& \frac{n_c}{2}\frac{\exp\left(r_{c_3}\right)}{1+\exp\left(r_{c_3}\right)}-\frac{1}{2}\\&-\frac{\left[a\left(\boldsymbol{x}_{ci},\mu_{c_3}\right)\right]^T\left[a\left(\boldsymbol{x}_{ci},\mu_{c_3}\right)\right]}{2\exp\left(s_{c_3}\right)}\frac{n_c-1}{n_c}\exp(r_{c_3})\\&+\frac{\left[a\left(\boldsymbol{x}_{ci},\mu_{c_3}\right)^T\mathbf{1}\right]^2\left[\exp\left(r_{c_3}\right)\left(n_c-1\right)+\frac{1}{\exp\left(r_{c_3}\right)}\right]}{2\exp\left(s_{c_3}\right)n_c^2}\end{aligned}$$

## H.15 The first derivative of $\partial \log\left[f_3\left(\boldsymbol{x}_i|\boldsymbol{\theta}_3\right)\right]/\partial r_{n_3}$

$$\begin{aligned}\frac{\partial \log\left[f_3(\boldsymbol{x}_i|\boldsymbol{\theta}_3)\right]}{\partial r_{n_3}} =& \frac{n_n}{2}\frac{\exp\left(r_{n_3}\right)}{1+\exp\left(r_{n_3}\right)}-\frac{1}{2}\\&-\frac{\left[a\left(\boldsymbol{x}_{ni},\mu_{n_3}\right)\right]^T\left[a\left(\boldsymbol{x}_{ni},\mu_{n_3}\right)\right]}{2\exp\left[s_{n_3}+\exp\left(\triangle_{n_3}\right)\right]}\frac{n_n-1}{n_n}\exp(r_{n_3})\\&+\frac{\left[a\left(\boldsymbol{x}_{ni},\mu_{n_3}\right)^T\mathbf{1}\right]^2\left[\exp\left(r_{n_3}\right)\left(n_n-1\right)+\frac{1}{\exp\left(r_{n_3}\right)}\right]}{2\exp\left[s_{n_3}+\exp(\triangle_{n_3})\right]n_n^2}\end{aligned}$$

## H.16 The first derivative of $\partial \log\left[f_3\left(\boldsymbol{x}_i|\boldsymbol{\theta}_3\right)\right]/\partial \mu_{c_3}$

$$\begin{aligned}\frac{\partial \log\left[f_3\left(\boldsymbol{x}_i|\boldsymbol{\theta}_3\right)\right]}{\partial \mu_{c_3}} =& -\frac{1}{2\exp\left(s_{c_3}\right)}\frac{n_c-1}{n_c}\left[1+\exp\left(r_{c_3}\right)\right]2\left(n_c\mu_{c_3}-\mathbf{1}^T\boldsymbol{x}_c\right)\\&+\frac{\left[(n_c-2)+\exp(r_{c_3})(n_c-1)-\frac{1}{\exp\left(r_{c_3}\right)}\right]}{2\exp\left(s_{c_3}\right)n_c^2}2n_c\left(n_c\mu_{c_3}-\mathbf{1}^T\boldsymbol{x}_c\right)\\=& -\frac{\left(n_c\mu_{c_3}-\mathbf{1}^T\boldsymbol{x}_c\right)}{\exp\left(s_{c_3}\right)}\frac{n_c-1}{n_c}\left[1+\exp\left(r_{c_3}\right)\right]\\&+\frac{\left(n_c\mu_{c_3}-\mathbf{1}^T\boldsymbol{x}_c\right)\left[(n_c-2)+\exp(r_{c_3})(n_c-1)-\frac{1}{\exp\left(r_{c_3}\right)}\right]}{\exp\left(s_{c_3}\right)n_c}\end{aligned}$$

## H.17 The first derivative of $\partial \log\left[f_3\left(\boldsymbol{x}_i|\boldsymbol{\theta}_3\right)\right]/\partial \mu_{n_3}$

$$\begin{aligned}\frac{\partial \log\left[f_3\left(\boldsymbol{x}_i|\boldsymbol{\theta}_3\right)\right]}{\partial \mu_{n_3}} =& -\frac{\left(n_n\mu_{n_3}-\mathbf{1}^T\boldsymbol{x}_n\right)}{\exp\left[s_{c_3}+\exp\left(\triangle_{n_3}\right)\right]}\frac{n_n-1}{n_n}\left[1+\exp\left(r_{n_3}\right)\right]\\&+\frac{\left(n_n\mu_{n_3}-\mathbf{1}^T\boldsymbol{x}_n\right)\left[(n_n-2)+\exp(r_{n_3})(n_n-1)-\frac{1}{\exp\left(r_{n_3}\right)}\right]}{\exp\left[s_{c_3}+\exp\left(\triangle_{n_3}\right)\right]n_n}\end{aligned}$$

## H.18 The first derivatives of $Q_\ell$

The first derivatives are:

$$\frac{\partial Q_\ell}{\partial \boldsymbol{\theta}_\ell} = \sum_{i=1}^{p} w_{i\ell}^{(m)} \frac{\partial \log\left[f_\ell\left(\boldsymbol{x}_i|\boldsymbol{\theta}_\ell\right)\right]}{\partial \boldsymbol{\theta}_\ell}$$

## H.19 The first derivative of $\partial Q_1/\partial s_{c_1}$

$$\begin{aligned}\frac{\partial Q_1}{\partial s_{c_1}} =& -\frac{n}{2}\sum_{i=1}^{p} w_{i1}^{(m)} + \frac{(n_c-1)}{2n_c\exp\left(s_{c_1}\right)}\left[1+\exp(r_{c_1})\right]\sum_{i=1}^{p} w_{i1}^{(m)}\left[a\left(\boldsymbol{x}_{ci},\mu_{c_1}\right)\right]^T\left[a\left(\boldsymbol{x}_{ci},\mu_{c_1}\right)\right]\\ &- \frac{\left[(n_c-2)+\exp(r_{c_1})(n_c-1)-\frac{1}{\exp(r_{c_1})}\right]}{2n_c^2\exp\left(s_{c_1}\right)}\sum_{i=1}^{p} w_{i1}^{(m)}\left[a\left(\boldsymbol{x}_{ci},\mu_{c_1}\right)^T\mathbf{1}\right]^2\\ &+ \frac{(n_n-1)}{2n_n\exp\left[s_{c_1}-\exp(\triangle_{n_1})\right]}\left[1+\exp(r_{n_1})\right]\sum_{i=1}^{p} w_{i1}^{(m)}\left[a\left(\boldsymbol{x}_{ni},\mu_{n_1}\right)\right]^T\left[a\left(\boldsymbol{x}_{ni},\mu_{n_1}\right)\right]\\ &- \frac{\left[(n_n-2)+\exp(r_{n_1})(n_n-1)-\frac{1}{\exp(r_{n_1})}\right]}{2n_n^2\exp\left[s_{c_1}-\exp(\triangle_{n_1})\right]}\sum_{i=1}^{p} w_{i1}^{(m)}\left[a\left(\boldsymbol{x}_{ni},\mu_{n_1}\right)^T\mathbf{1}\right]^2\end{aligned}$$

## H.20 The first derivative of $\partial Q_1/\partial \triangle_{n_1}$

$$\begin{aligned}\frac{\partial Q_1}{\partial \triangle_{n_1}} =& \frac{n_n}{2}\exp\left(\triangle_{n_1}\right)\sum_{i=1}^{p} w_{i1}^{(m)} - \frac{n_n-1}{2n_n}\left[1+\exp\left(r_{n_1}\right)\right]\frac{\exp\left(\triangle_{n_1}\right)}{\exp\left[s_{c_1}-\exp\left(\triangle_{n_1}\right)\right]}\sum_{i=1}^{p} w_{i1}^{(m)}\left[a\left(\boldsymbol{x}_{ni},\mu_{n_1}\right)\right]^T\left[a\left(\boldsymbol{x}_{ni},\mu_{n_1}\right)\right]\\ &+ \frac{\left[(n_n-2)+\exp\left(r_{n_1}\right)(n_n-1)-\frac{1}{\exp\left(r_{n_1}\right)}\right]}{2n_n^2}\frac{\exp\left(\triangle_{n_1}\right)}{\exp\left[s_{c_1}-\exp\left(\triangle_{n_1}\right)\right]}\sum_{i=1}^{p} w_{i1}^{(m)}\left[a\left(\boldsymbol{x}_{ni},\mu_{n_1}\right)^T\mathbf{1}\right]^2\end{aligned}$$

## H.21 The first derivative of $\partial Q_1/\partial r_{c_1}$

$$\begin{aligned}\frac{\partial Q_1}{\partial r_{c_1}} =& \left[\frac{n_c}{2}\frac{\exp\left(r_{c_1}\right)}{1+\exp\left(r_{c_1}\right)}-\frac{1}{2}\right]\sum_{i=1}^{p} w_{i1}^{(m)}\\ &- \frac{1}{2\exp\left(s_{c_1}\right)}\frac{n_c-1}{n_c}\exp(r_{c_1})\sum_{i=1}^{p} w_{i1}^{(m)}\left[a\left(\boldsymbol{x}_{ci},\mu_{c_1}\right)\right]^T\left[a\left(\boldsymbol{x}_{ci},\mu_{c_1}\right)\right]\\ &+ \frac{\left[\exp\left(r_{c_1}\right)(n_c-1)+\frac{1}{\exp(r_{c_1})}\right]}{2\exp\left(s_{c_1}\right)n_c^2}\sum_{i=1}^{p} w_{i1}^{(m)}\left[a\left(\boldsymbol{x}_{ci},\mu_{c_1}\right)^T\mathbf{1}\right]^2\end{aligned}$$

## H.22 The first derivative of $\partial Q_1/\partial r_{n_1}$

$$\frac{\partial Q_1}{\partial r_{n_1}} = \left[\frac{n_n}{2}\frac{\exp\left(r_{n_1}\right)}{1+\exp\left(r_{n_1}\right)} - \frac{1}{2}\right]\sum_{i=1}^{p} w_{i1}^{(m)}$$
$$- \frac{1}{2\exp\left[s_{n_1} - \exp(\triangle_{n_1})\right]}\frac{n_n - 1}{n_n}\exp(r_{n_1})\sum_{i=1}^{p} w_{i1}^{(m)}\left[a\left(\boldsymbol{x}_{ni}, \mu_{n_1}\right)\right]^T\left[a\left(\boldsymbol{x}_{ni}, \mu_{n_1}\right)\right]$$
$$+ \frac{\left[\exp\left(r_{n_1}\right)\left(n_n - 1\right) + \frac{1}{\exp\left(r_{n_1}\right)}\right]}{2\exp\left[s_{n_1} - \exp\left(\triangle_{n_1}\right)\right] n_n^2}\sum_{i=1}^{p} w_{i1}^{(m)}\left[a\left(\boldsymbol{x}_{ni}, \mu_{n_1}\right)^T \mathbf{1}\right]^2$$

## H.23 The first derivative of $\partial Q_1/\partial \mu_{c_1}$

$$\frac{\partial Q_1}{\partial \mu_{c_1}} = -\frac{1}{\exp\left(s_{c_1}\right)}\frac{n_c - 1}{n_c}\left[1+\exp\left(r_{c_1}\right)\right]\sum_{i=1}^{p} w_{i1}^{(m)}\left(n_c\mu_{c_1} - \mathbf{1}^T\boldsymbol{x}_c\right)$$
$$+ \frac{\left[(n_c - 2) + \exp(r_{c_1})(n_c - 1) - \frac{1}{\exp\left(r_{c_1}\right)}\right]}{\exp\left(s_{c_1}\right) n_c}\sum_{i=1}^{p} w_{i1}^{(m)}\left(n_c\mu_{c_1} - \mathbf{1}^T\boldsymbol{x}_c\right)$$

## H.24 The first derivative of $\partial Q_1/\partial \mu_{n_1}$

$$\frac{\partial Q_1}{\partial \mu_{n_1}} = -\frac{1}{\exp\left[s_{c_1} - \exp\left(\triangle_{n_1}\right)\right]}\frac{n_n - 1}{n_n}\left[1+\exp\left(r_{n_1}\right)\right]\sum_{i=1}^{p} w_{i1}^{(m)}\left(n_n\mu_{n_1} - \mathbf{1}^T\boldsymbol{x}_n\right)$$
$$+ \frac{\left[(n_n - 2) + \exp(r_{n_1})(n_n - 1) - \frac{1}{\exp\left(r_{n_1}\right)}\right]}{\exp\left[s_{c_1} - \exp\left(\triangle_{n_1}\right)\right] n_n}\sum_{i=1}^{p} w_{i1}^{(m)}\left(n_n\mu_{n_1} - \mathbf{1}^T\boldsymbol{x}_n\right)$$

## H.25 The first derivative of $\partial Q_2/\partial s_2$

$$\frac{\partial Q_2}{\partial s_2} = -\frac{n}{2}\sum_{i=1}^{p} w_{i2}^{(m)} + \frac{1}{2\exp(s_2)}\frac{n_c - 1}{n_c}\left[1+\exp\left(r_{c_2}\right)\right]\sum_{i=1}^{p} w_{i2}^{(m)}\left[a\left(\boldsymbol{x}_{ci}, \mu_{c_2}\right)\right]^T\left[a\left(\boldsymbol{x}_{ci}, \mu_{c_2}\right)\right]$$
$$- \frac{\left[(n_c - 2) + \exp(r_{c_2})(n_c - 1) - \frac{1}{\exp\left(r_{c_2}\right)}\right]}{2\exp\left(s_2\right) n_c^2}\sum_{i=1}^{p} w_{i2}^{(m)}\left[a\left(\boldsymbol{x}_{ci}, \mu_{c_2}\right)^T \mathbf{1}\right]^2$$
$$+ \frac{1}{2\exp(s_2)}\frac{n_n - 1}{n_n}\left[1+\exp\left(r_{n_2}\right)\right]\sum_{i=1}^{p} w_{i2}^{(m)}\left[a\left(\boldsymbol{x}_{ni}, \mu_{n_2}\right)\right]^T\left[a\left(\boldsymbol{x}_{ni}, \mu_{n_2}\right)\right]$$
$$- \frac{\left[(n_n - 2) + \exp(r_{n_2})(n_n - 1) - \frac{1}{\exp\left(r_{n_2}\right)}\right]}{2\exp\left(s_2\right) n_n^2}\sum_{i=1}^{p} w_{i2}^{(m)}\left[a\left(\boldsymbol{x}_{ni}, \mu_{n_2}\right)^T \mathbf{1}\right]^2$$

## H.26 The first derivative of $\partial Q_2/\partial r_{c_2}$

$$\begin{aligned}\frac{\partial Q_2}{\partial r_{c_2}} =& \left[\frac{n_c}{2}\frac{\exp\left(r_{c_2}\right)}{1+\exp\left(r_{c_2}\right)}-\frac{1}{2}\right]\sum_{i=1}^{p} w_{i2}^{(m)} \\ &- \frac{1}{2\exp\left(s_2\right)}\frac{n_c-1}{n_c}\exp(r_{c_2})\sum_{i=1}^{p} w_{i2}^{(m)}\left[a\left(\boldsymbol{x}_{ci},\mu_{c_2}\right)\right]^T\left[a\left(\boldsymbol{x}_{ci},\mu_{c_2}\right)\right] \\ &+ \frac{\left[\exp\left(r_{c_2}\right)\left(n_c-1\right)+\frac{1}{\exp\left(r_{c_2}\right)}\right]}{2\exp\left(s_2\right)n_c^2}\sum_{i=1}^{p} w_{i2}^{(m)}\left[a\left(\boldsymbol{x}_{ci},\mu_{c_2}\right)^T\mathbf{1}\right]^2\end{aligned}$$

## H.27 The first derivative of $\partial Q_2/\partial r_{n_2}$

$$\begin{aligned}\frac{\partial Q_2}{\partial r_{n_2}} =& \left[\frac{n_n}{2}\frac{\exp\left(r_{n_2}\right)}{1+\exp\left(r_{n_2}\right)}-\frac{1}{2}\right]\sum_{i=1}^{p} w_{i2}^{(m)} \\ &- \frac{1}{2\exp\left(s_2\right)}\frac{n_n-1}{n_n}\exp(r_{n_2})\sum_{i=1}^{p} w_{i2}^{(m)}\left[a\left(\boldsymbol{x}_{ci},\mu_{n_2}\right)\right]^T\left[a\left(\boldsymbol{x}_{ni},\mu_{n_2}\right)\right] \\ &+ \frac{\left[\exp\left(r_{n_2}\right)\left(n_n-1\right)+\frac{1}{\exp\left(r_{n_2}\right)}\right]}{2\exp\left(s_2\right)n_n^2}\sum_{i=1}^{p} w_{i2}^{(m)}\left[a\left(\boldsymbol{x}_{ni},\mu_{n_2}\right)^T\mathbf{1}\right]^2\end{aligned}$$

## H.28 The first derivative of $\partial Q_2/\partial \mu_{c_2}$

$$\begin{aligned}\frac{\partial Q_2}{\partial \mu_{c_2}} =& -\frac{1}{\exp\left(s_2\right)}\frac{n_c-1}{n_c}\left[1+\exp\left(r_{c_2}\right)\right]\sum_{i=1}^{p} w_{i2}^{(m)}\left(n_c\mu_{c_2}-\mathbf{1}^T\boldsymbol{x}_c\right) \\ &+ \frac{\left[(n_c-2)+\exp(r_{c_2})(n_c-1)-\frac{1}{\exp\left(r_{c_2}\right)}\right]}{\exp\left(s_2\right)n_c}\sum_{i=1}^{p} w_{i2}^{(m)}\left(n_c\mu_{c_2}-\mathbf{1}^T\boldsymbol{x}_c\right)\end{aligned}$$

## H.29 The first derivative of $\partial Q_2/\partial \mu_{n_2}$

$$\begin{aligned}\frac{\partial Q_2}{\partial \mu_{n_2}} =& -\frac{1}{\exp\left(s_2\right)}\frac{n_n-1}{n_n}\left[1+\exp\left(r_{n_2}\right)\right]\sum_{i=1}^{p} w_{i2}^{(m)}\left(n_n\mu_{n_2}-\mathbf{1}^T\boldsymbol{x}_n\right) \\ &+ \frac{\left[(n_n-2)+\exp(r_{n_2})(n_n-1)-\frac{1}{\exp\left(r_{n_2}\right)}\right]}{\exp\left(s_2\right)n_n}\sum_{i=1}^{p} w_{i2}^{(m)}\left(n_n\mu_{n_2}-\mathbf{1}^T\boldsymbol{x}_n\right)\end{aligned}$$

## H.30 The first derivative of $\partial Q_3/\partial s_{c_3}$

$$\frac{\partial Q_3}{\partial s_{c_3}} = -\frac{n}{2}\sum_{i=1}^{p} w_{i3}^{(m)} + \frac{(n_c-1)}{2n_c \exp\left(s_{c_3}\right)}\left[1+\exp(r_{c_3})\right]\sum_{i=1}^{p} w_{i3}^{(m)}\left[a\left(\boldsymbol{x}_{ci},\mu_{c_3}\right)\right]^T\left[a\left(\boldsymbol{x}_{ci},\mu_{c_3}\right)\right]$$
$$-\frac{\left[(n_c-2)+\exp(r_{c_3})(n_c-1)-\frac{1}{\exp(r_{c_3})}\right]}{2n_c^2\exp\left(s_{c_3}\right)}\sum_{i=1}^{p} w_{i3}^{(m)}\left[a\left(\boldsymbol{x}_{ci},\mu_{c_3}\right)^T\mathbf{1}\right]^2$$
$$+\frac{(n_n-1)}{2n_n\exp\left[s_{c_3}+\exp(\triangle_{n_3})\right]}\left[1+\exp(r_{n_3})\right]\sum_{i=1}^{p} w_{i3}^{(m)}\left[a\left(\boldsymbol{x}_{ni},\mu_{n_3}\right)\right]^T\left[a\left(\boldsymbol{x}_{ni},\mu_{n_3}\right)\right]$$
$$-\frac{\left[(n_n-2)+\exp(r_{n_3})(n_n-1)-\frac{1}{\exp(r_{n_3})}\right]}{2n_n^2\exp\left[s_{c_3}+\exp(\triangle_{n_3})\right]}\sum_{i=1}^{p} w_{i3}^{(m)}\left[a\left(\boldsymbol{x}_{ni},\mu_{n_3}\right)^T\mathbf{1}\right]^2$$

## H.31 The first derivative of $\partial Q_3/\partial \triangle_{n_3}$

$$\frac{\partial Q_3}{\partial \triangle_{n_3}} = -\frac{n_n}{2}\exp\left(\triangle_{n_3}\right)\sum_{i=1}^{p} w_{i3}^{(m)} - \frac{1}{2}\frac{n_n-1}{n_n}\left[1+\exp\left(r_{n_3}\right)\right]\frac{\exp\left(\triangle_{n_3}\right)}{\exp\left[s_{c_3}+\exp\left(\triangle_{n_3}\right)\right]}\sum_{i=1}^{p} w_{i3}^{(m)}\left[a\left(\boldsymbol{x}_{ni},\mu_{n_3}\right)\right]^T\left[a\left(\boldsymbol{x}_{ni},\mu_{n_3}\right)\right]$$
$$-\frac{\left[(n_n-2)+\exp\left(r_{n_3}\right)(n_n-1)-\frac{1}{\exp(r_{n_3})}\right]}{2n_n^2}\frac{\exp\left(\triangle_{n_3}\right)}{\exp\left[s_{c_3}+\exp\left(\triangle_{n_3}\right)\right]}\sum_{i=1}^{p} w_{i3}^{(m)}\left[a\left(\boldsymbol{x}_{ni},\mu_{n_3}\right)^T\mathbf{1}\right]^2$$

## H.32 The first derivative of $\partial Q_3/\partial r_{c_3}$

$$\frac{\partial Q_3}{\partial r_{c_3}} = \left[\frac{n_c}{2}\frac{\exp\left(r_{c_3}\right)}{1+\exp\left(r_{c_3}\right)}-\frac{1}{2}\right]\sum_{i=1}^{p} w_{i3}^{(m)}$$
$$-\frac{1}{2\exp\left(s_{c_3}\right)}\frac{n_c-1}{n_c}\exp(r_{c_3})\sum_{i=1}^{p} w_{i3}^{(m)}\left[a\left(\boldsymbol{x}_{ci},\mu_{c_3}\right)\right]^T\left[a\left(\boldsymbol{x}_{ci},\mu_{c_3}\right)\right]$$
$$+\frac{\left[\exp\left(r_{c_3}\right)(n_c-1)+\frac{1}{\exp(r_{c_3})}\right]}{2\exp\left(s_{c_3}\right)n_c^2}\sum_{i=1}^{p} w_{i3}^{(m)}\left[a\left(\boldsymbol{x}_{ci},\mu_{c_3}\right)^T\mathbf{1}\right]^2$$

## H.33 The first derivative of $\partial Q_3/\partial r_{n_3}$

$$\frac{\partial Q_3}{\partial r_{n_3}} = \left[\frac{n_n}{2}\frac{\exp\left(r_{n_3}\right)}{1+\exp\left(r_{n_3}\right)}-\frac{1}{2}\right]\sum_{i=1}^{p} w_{i3}^{(m)}$$
$$-\frac{1}{2\exp\left[s_{n_3}+\exp\left(\triangle_{n_3}\right)\right]}\frac{n_n-1}{n_n}\exp(r_{n_3})\sum_{i=1}^{p} w_{i3}^{(m)}\left[a\left(\boldsymbol{x}_{ni},\mu_{n_3}\right)\right]^T\left[a\left(\boldsymbol{x}_{ni},\mu_{n_3}\right)\right]$$
$$+\frac{\left[\exp\left(r_{n_3}\right)(n_n-1)+\frac{1}{\exp(r_{n_3})}\right]}{2\exp\left[s_{n_3}+\exp(\triangle_{n_3})\right]n_n^2}\sum_{i=1}^{p} w_{i3}^{(m)}\left[a\left(\boldsymbol{x}_{ni},\mu_{n_3}\right)^T\mathbf{1}\right]^2$$

### H.34 The first derivative of $\partial Q_3/\partial\mu_{c_3}$

$$\begin{aligned}\frac{\partial Q_3}{\partial\mu_{c_3}} =& -\frac{1}{\exp\left(s_{c_3}\right)}\frac{n_c-1}{n_c}\left[1+\exp\left(r_{c_3}\right)\right]\sum_{i=1}^{p}w_{i3}^{(m)}\left(n_c\mu_{c_3}-\mathbf{1}^T\boldsymbol{x}_c\right)\\ &+\frac{\left[(n_c-2)+\exp(r_{c_3})(n_c-1)-\frac{1}{\exp(r_{c_3})}\right]}{\exp\left(s_{c_3}\right)n_c}\sum_{i=1}^{p}w_{i3}^{(m)}\left(n_c\mu_{c_3}-\mathbf{1}^T\boldsymbol{x}_c\right)\end{aligned}$$

### H.35 The first derivative of $\partial Q_3/\partial\mu_{n_3}$

$$\begin{aligned}\frac{\partial Q_3}{\partial\mu_{n_3}} =& -\frac{1}{\exp\left[s_{c_3}+\exp\left(\triangle_{n_3}\right)\right]}\frac{n_n-1}{n_n}\left[1+\exp\left(r_{n_3}\right)\right]\sum_{i=1}^{p}w_{i3}^{(m)}\left(n_n\mu_{n_3}-\mathbf{1}^T\boldsymbol{x}_n\right)\\ &+\frac{\left[(n_n-2)+\exp(r_{n_3})(n_n-1)-\frac{1}{\exp(r_{n_3})}\right]}{\exp\left[s_{c_3}+\exp\left(\triangle_{n_3}\right)\right]n_n}\sum_{i=1}^{p}w_{i3}^{(m)}\left(n_n\mu_{n_3}-\mathbf{1}^T\boldsymbol{x}_n\right)\end{aligned}$$

# I Log-density function for the multivariate normal distribution with special mean vector and covariance matrix

Suppose a $n\times 1$ random vector $\boldsymbol{X}$ has multivariate normal distribution $\mathrm{N}\left(\boldsymbol{\mu},\ \boldsymbol{\Sigma}\right)$, where

$$\boldsymbol{\mu}=\mu_0\mathbf{1}_n,\quad \boldsymbol{\Sigma}=\sigma^2(1-\rho)\boldsymbol{R}_0,\quad \boldsymbol{R}_0=\boldsymbol{I}_n+\frac{\rho}{(1-\rho)}\mathbf{1}_n\mathbf{1}_n^T. \tag{A15}$$

The density function is

$$\begin{aligned}f(\boldsymbol{x}) &= (2\pi)^{-n/2}\left|\boldsymbol{\Sigma}\right|^{-1/2}\exp\left[-\frac{(\boldsymbol{x}-\boldsymbol{\mu})^T\boldsymbol{\Sigma}^{-1}(\boldsymbol{x}-\boldsymbol{\mu})}{2}\right]\\ &= (2\pi)^{-n/2}\left|\sigma^2(1-\rho)\boldsymbol{R}_0\right|^{-1/2}\exp\left[-\frac{(\boldsymbol{x}-\mu_0\mathbf{1}_n)^T\boldsymbol{R}_0^{-1}(\boldsymbol{x}-\mu_0\mathbf{1}_n)}{2[\sigma^2(1-\rho)]}\right]\\ &= \left[2\pi\sigma^2(1-\rho)\right]^{-n/2}\left|\boldsymbol{R}_0\right|^{-1/2}\exp\left[-\frac{(\boldsymbol{x}-\mu_0\mathbf{1}_n)^T\boldsymbol{R}_0^{-1}(\boldsymbol{x}-\mu_0\mathbf{1}_n)}{2[\sigma^2(1-\rho)]}\right]\end{aligned} \tag{A16}$$

The matrix cookbook[1] shows the following result:

$$(\boldsymbol{A}+\boldsymbol{BC})^{-1} = \boldsymbol{A}^{-1} - \boldsymbol{A}^{-1}\boldsymbol{B}\left[\boldsymbol{I}+\boldsymbol{C}\boldsymbol{A}^{-1}\boldsymbol{B}\right]^{-1}\boldsymbol{C}\boldsymbol{A}^{-1} \tag{A17}$$

and

$$\left|\boldsymbol{I}+\boldsymbol{u}\boldsymbol{v}^T\right| = 1+\boldsymbol{u}^T\boldsymbol{v}. \tag{A18}$$

Let

$$\boldsymbol{A}=\boldsymbol{I}_n,\ \boldsymbol{B}=\frac{\rho}{(1-\rho)}\mathbf{1}_n,\ \boldsymbol{C}=\mathbf{1}^T,\ \boldsymbol{u}=\frac{\rho}{(1-\rho)}\mathbf{1}_n,\ \boldsymbol{v}=\mathbf{1}_n.$$

We have

$$\begin{aligned}\boldsymbol{R}_0^{-1} &= \left[\boldsymbol{I}_n+\frac{\rho}{(1-\rho)}\mathbf{1}_n\mathbf{1}_n^T\right]^{-1} = \boldsymbol{I}_n - \frac{\rho}{[1+(n-1)\rho]}\mathbf{1}_n\mathbf{1}_n^T \\ |\boldsymbol{R}_0| &= 1+\frac{\rho}{(1-\rho)}\mathbf{1}_n^T\mathbf{1}_n = \frac{[1+(n-1)\rho]}{(1-\rho)}.\end{aligned} \tag{A19}$$

Denote

$$\boldsymbol{a}(\boldsymbol{x},\mu_0) = (\boldsymbol{x}-\mu_0\mathbf{1}_n). \tag{A20}$$

Therefore the density function can be simplified into

$$\begin{aligned}f(\boldsymbol{x}) &= \left[2\pi\sigma^2\right]^{-n/2}\left\{(1-\rho)^{n-1}[1+(n-1)\rho]\right\}^{-1/2} \\ &\cdot \exp\left[-\frac{[\boldsymbol{a}(\boldsymbol{x},\mu_0)]^T[\boldsymbol{a}(\boldsymbol{x},\mu_0)] - \frac{\rho}{[1+(n-1)\rho]}\left[\boldsymbol{a}(\boldsymbol{x},\mu_0)^T\mathbf{1}_n\right]^2}{2[\sigma^2(1-\rho)]}\right]\end{aligned} \tag{A21}$$

Log-density function is

$$\begin{aligned}\log[f(\boldsymbol{x})] = &-\frac{n}{2}\log(2\pi) - \frac{n}{2}\log(\sigma^2) - \frac{1}{2}\left\{(n-1)\log[(1-\rho)] + \log[1+(n-1)\rho]\right\} \\ &- \frac{[\boldsymbol{a}(\boldsymbol{x},\mu_0)]^T[\boldsymbol{a}(\boldsymbol{x},\mu_0)] - \frac{\rho}{[1+(n-1)\rho]}\left[\boldsymbol{a}(\boldsymbol{x},\mu_0)^T\mathbf{1}_n\right]^2}{2[\sigma^2(1-\rho)]}.\end{aligned} \tag{A22}$$

# References

[1] http://matrixcookbook.com/
